# Supplementary material for: Regio- and Stereoselective Switchable Synthesis of (E)- and (Z)-N-Carbonylvinylated Pyrazoles
Source: Molecules. 2023 May 25;28(11):4347. doi: 10.3390/molecules28114347 (PMC10254332; doi:10.3390/molecules28114347)
Supplement: Supplementary file 1 [file molecules-28-04347-s001.zip › molecules-2395418-supplementary.pdf]

Supporting Information for:

Regio- and Stereoselective Switchable Synthesis of (*E*)- and (*Z*)-*N*- acylvinylated Pyrazoles

Xue Zhang, Zheyu Zhang, Haifeng Yu*, Guangbo Che*

(College of Chemistry, Baicheng Normal University, Baicheng, Jilin 137000)

Table of contents

I. General information ……………………………………………………….…...S2

II. Synthetic procedures and analytical data of compounds **3**, **4** ……………………S2

III. Synthetic procedures and analytical data of compounds **5**, **6**…………………..S16

IV. General procedures for a gram scale synthesis of **3a** and **4a** …………………..S34

V. Copies of ^{1}H NMR and ^{13}C NMR spectra of compounds ………………………S35

VI. Crystal Data of **3l**, **4o**, **5c**, **5k'**, **6c** ……………………………………………S127

I. General Information

All silver-catalyzed reactions were carried out without any particular precautions to extrude moisture or oxygen.

All reagents were purchased from commercial sources and used without further purification, unless otherwise indicated. All reactions were monitored by TLC, which was performed on precoated aluminum sheets of silica gel 60 (F254). The products were purified by flash column chromatography on silica gel (300−400 mesh). Melting points were uncorrected. ^{1}H NMR and ^{13}C NMR spectra were recorded at 25 ºC on a Varian 400 MHz. ^{1}H NMR and ^{13}C NMR were determined with TMS as the internal standard. All chemical shifts are given in ppm. High-resolution mass spectra (HRMS) were obtained using a microTOF II focus spectrometer (ESI).

II. Synthetic procedures and analytical data of compounds 3, 4

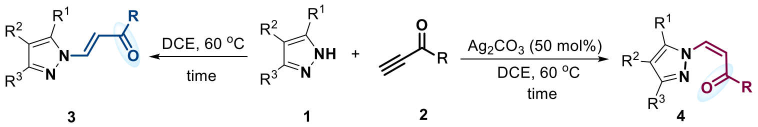

3 1 2 4

General procedure for the synthesis of 3 (taking 3a as an example): To a solution of 3,5-dimethyl-1H-pyrazole (**1aa**) (24.0 mg, 0.25 mmol) and ethyl propionate (**2a**) (30.4 µL, 0.3 mmol) in DCE (1 ml) at 60 ºC. After the reaction was finished as indicated by TLC (reaction time, 8 h), the resulting mixture was poured into water (10 mL) and extracted with DCM (CH_2Cl_2, 10 mL×3). The combined organic layer was dried over anhydrous Na_2SO_4 and concentrated *in vacuo*. Purification of the crude product with flash column chromatography (petroleum ether (60-90 ºC)/ ethyl acetate = 10:1, v/v) to give **3a** (43.7 mg, 90%).

General procedure for the synthesis of 4 (taking 4a as an example): To a solution of 3,5-dimethyl-1H-pyrazole (**1aa**) (24.0 mg, 0.25 mmol) and ethyl propionate (**2a**) (30.4 µL, 0.3 mmol) in DCE (1 ml) at 60 ºC was added Ag_2CO_3 (34.5 mg, 0.125 mmol). After the reaction was finished as indicated by TLC (reaction time, 12 h), the resulting mixture was poured into water (10 mL) and extracted with DCM (CH_2Cl_2, 10 mL×3). The combined organic layer was dried over anhydrous Na_2SO_4 and concentrated *in vacuo*. Purification of the crude product with flash column chromatography (petroleum ether (60-90 ºC)/ ethyl acetate = 8:1, v/v) to give **4a** (43.2 mg, 89%).

Analytical data of compounds 3

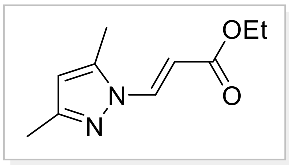

Ethyl (E)-3-(3,5-dimethyl-1H-pyrazol-1-yl)acrylate (3a). Eluent: petroleum ether (60-90 °C)/ ethyl acetate = 10:1 (v/v). Colourless oil, 43.7 mg, yield 90%. NMR Spectroscopy: ^{1}H NMR ($CDCl_3$, 400 MHz) δ 1.31 (t, J = 7.1 Hz, 3H), 2.25 (s, 3H), 2.34 (s, 3H), 4.24 (q, J = 7.1 Hz, 2H), 5.95 (s, 1H), 6.39 (d, J = 13.6 Hz, 1H), 7.82 (d, J = 13.5 Hz, 1H). ^{13}C NMR ($CDCl_3$, 100 MHz) δ 10.9, 13.9, 14.4, 60.4, 104.6, 109.0, 136.1, 141.6, 152.9, 167.6. Mass Spectrometry: HRMS (ESI-TOF) (m/z): Calcd for $C_{10}H_{15}N_2O_2$ ([M + H]$^+$) 195.1128, found 195.1126.

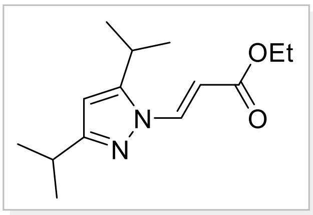

Ethyl (E)-3-(3,5-diisopropyl-1H-pyrazol-1-yl)acrylate (3b). Eluent: petroleum ether (60-90 °C)/ ethyl acetate = 10:1 (v/v). Colourless oil, 55 mg, yield 88%. NMR Spectroscopy: ^{1}H NMR ($CDCl_3$, 400 MHz) δ 1.26-1.32 (m, 15H), 2.91-2.98 (m, 1H), 3.03-3.09 (m, 1H), 4.26 (q, J = 7.1 Hz, 2H), 5.98 (s, 1H), 6.45 (d, J = 13.4 Hz, 1H), 7.89 (d, J = 13.4 Hz, 1H). ^{13}C NMR ($CDCl_3$, 100 MHz) δ 14.5, 22.4 (2C), 22.7 (2C), 25.1, 28.3, 60.4, 102.7, 104.8, 136.3, 152.5, 162.7, 167.7. Mass Spectrometry: HRMS (ESI-TOF) (m/z): Calcd for $C_{14}H_{23}N_2O_2$ ([M + H]$^+$) 251.1754, found 251.1758.

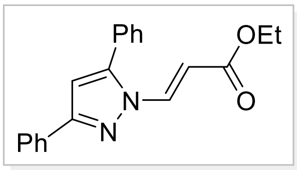

Ethyl (E)-3-(3,5-diphenyl-1H-pyrazol-1-yl)acrylate (3c). Eluent: petroleum ether (60-90 °C)/ ethyl acetate = 10:1 (v/v). White solid, 69.2 mg, yield 87%. m.p: 77.5 °C. NMR Spectroscopy: ^{1}H NMR ($CDCl_3$, 400 MHz) δ 1.29 (t, J = 7.1 Hz, 3H), 4.22 (q, J = 7.1 Hz, 2H), 6.70 (d, J = 13.5 Hz, 1H), 6.75 (s, 1H), 7.36-7.40 (m, 1H), 7.42-7.46 (m, 4H), 7.48-7.54 (m, 3H), 7.91-7.93 (m, 2H), 7.95 (d, J = 13.5 Hz, 1H). ^{13}C NMR ($CDCl_3$, 100 MHz) δ 14.4, 60.5, 106.6, 106.9, 126.3 (2C), 128.8, 128.9 (2C), 129.1, 129.2 (2C), 129.6 (2C), 129.7, 132.1, 137.4, 147.2, 154.1, 167.3. Mass Spectrometry: HRMS (ESI-TOF) (m/z): Calcd for $C_{20}H_{19}N_2O_2$ ([M + H]$^+$) 319.1441, found 319.1443.

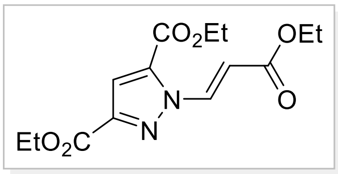

Diethyl (E)-1-(3-ethoxy-3-oxoprop-1-en-1-yl)-1H-pyrazole-3,5-dicarboxylate (3d). Eluent: petroleum ether (60-90 °C)/ ethyl acetate = 10:1 (v/v). Colourless oil, 63.6 mg, yield 82%. NMR Spectroscopy: ^{1}H NMR ($CDCl_3$, 400 MHz) δ 1.33 (t, *J* = 7.1 Hz, 3H), 1.40-1.44 (m, 6H), 4.28 (q, *J* = 7.1 Hz, 2H), 4.39-4.46 (m, 4H), 6.84 (d, *J* = 13.8 Hz, 1H), 7.44 (s, 1H), 8.95 (d, *J* = 13.8 Hz, 1H). ^{13}C NMR ($CDCl_3$, 100 MHz) δ 14.3, 14.4 (2C), 60.9, 61.9, 62.3, 112.4, 115.9, 134.8, 137.6, 145.4, 158.5, 161.1, 165.9. Mass Spectrometry: HRMS (ESI-TOF) (m/z): Calcd for $C_{14}H_{19}N_2O_6$ ($[M + H]^+$) 311.1238, found 311.1239.

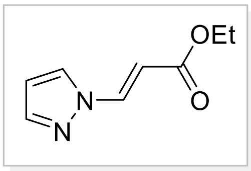

Ethyl (E)-3-(1H-pyrazol-1-yl)acrylate (3e). Eluent: petroleum ether (60-90 °C)/ ethyl acetate = 10:1 (v/v). White solid, 35.3 mg, yield 85%. m.p: 72.1 ºC. NMR Spectroscopy: ^{1}H NMR ($CDCl_3$, 400 MHz) δ 1.32 (t, *J* = 7.1 Hz, 3H), 4.26 (q, *J* = 7.1 Hz, 2H), 6.35 (d, *J* = 14.0 Hz, 1H), 6.44 (s, 1H), 7.65-7.72 (m, 2H), 7.99 (d, *J* = 14.0 Hz, 1H). ^{13}C NMR ($CDCl_3$, 100 MHz) δ 14.4, 60.7, 106.0, 109.2, 130.1, 139.7, 143.6, 166.7. Mass Spectrometry: HRMS (ESI-TOF) (m/z): Calcd for $C_8H_{11}N_2O_2$ ($[M + H]^+$) 167.0815, found 167.0815.

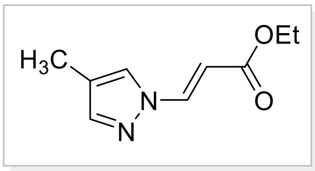

Ethyl (E)-3-(4-methyl-1H-pyrazol-1-yl)acrylate (3f). Eluent: petroleum ether (60-90 °C)/ ethyl acetate = 10:1 (v/v). White solid, 40.5 mg, yield 90%. m.p: 83.3 ºC. NMR Spectroscopy: ^{1}H NMR ($CDCl_3$, 400 MHz) δ 1.32 (t, *J* = 7.1 Hz, 3H), 2.11 (s, 3H), 4.25 (q, *J* = 7.1 Hz, 2H), 6.21 (d, *J* = 14.0 Hz, 1H), 7.42 (s, 1H), 7.53 (s, 1H), 7.92 (d, *J* = 14.0 Hz, 1H). ^{13}C NMR ($CDCl_3$, 100 MHz) δ 9.0, 14.4, 60.6, 104.5, 120.1, 128.0, 140.0, 144.8, 166.9. Mass Spectrometry: HRMS (ESI-TOF) (m/z): Calcd for $C_9H_{13}N_2O_2$ ($[M + H]^+$) 181.0972, found 181.0975.

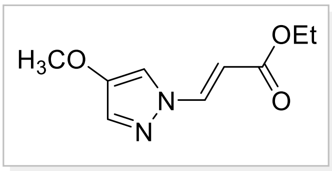

Ethyl (E)-3-(4-methoxy-1H-pyrazol-1-yl)acrylate (3g). Eluent: petroleum ether (60-90 °C)/ ethyl acetate = 10:1 (v/v). White solid, 42.6 mg, yield 87%. m.p: 82 ºC. NMR Spectroscopy: ^{1}H NMR ($CDCl_3$, 400 MHz) δ 1.31 (t, *J* = 7.1 Hz, 3H), 3.79 (s, 3H), 4.24 (q, *J* = 7.2 Hz, 2H), 6.07 (d, *J* = 14.1 Hz, 1H), 7.28 (s, 1H), 7.49 (s, 1H), 7.89 (d, *J* = 14.1 Hz, 1H). ^{13}C NMR ($CDCl_3$, 100 MHz) δ 14.4, 58.9, 60.6, 103.5, 111.2, 133.5, 140.8, 149.4, 166.7. Mass Spectrometry: HRMS (ESI-TOF) (m/z): Calcd for $C_9H_{13}N_2O_3$ ([M + H]$^+$) 197.0921, found 197.0926.

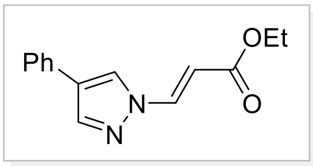

Ethyl (E)-3-(4-phenyl-1H-pyrazol-1-yl)acrylate (3h). Eluent: petroleum ether (60-90 °C)/ ethyl acetate = 10:1 (v/v). White solid, 47.8 mg, yield 79%. m.p: 109.7 ºC. NMR Spectroscopy: ^{1}H NMR ($CDCl_3$, 400 MHz) δ 1.34 (t, *J* = 7.1 Hz, 3H), 4.27 (q, *J* = 7.1 Hz, 2H), 6.36 (d, *J* = 14.0 Hz, 1H), 7.28-7.32 (m, 1H), 7.38-7.42 (m, 2H), 7.50-7.52 (m, 2H), 7.88 (s, 1H), 8.00 (t, *J* = 7.2 Hz, 2H). ^{13}C NMR ($CDCl_3$, 100 MHz) δ 14.5, 60.8, 106.0, 125.8, 126.0 (2C), 126.3, 127.7, 129.2 (2C), 131.0, 139.8, 141.6, 166.6. Mass Spectrometry: HRMS (ESI-TOF) (m/z): Calcd for $C_{14}H_{15}N_2O_2$ ([M + H]$^+$) 243.1128, found 243.1134.

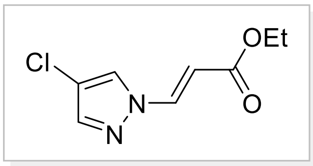

Ethyl (E)-3-(4-chloro-1H-pyrazol-1-yl)acrylate (3i). Eluent: petroleum ether (60-90 °C)/ ethyl acetate = 10:1 (v/v). White solid, 42 mg, yield 84%. m.p: 107.1 ºC. NMR Spectroscopy: ^{1}H NMR ($CDCl_3$, 400 MHz) δ 1.32 (t, *J* = 7.1 Hz, 3H), 4.26 (q, *J* = 7.1 Hz, 2H), 6.29 (d, *J* = 14.0 Hz, 1H), 7.63 (s, 1H), 7.66 (s, 1H), 7.87 (d, *J* = 14.0 Hz, 1H). ^{13}C NMR ($CDCl_3$, 100 MHz) δ 14.4, 61.0, 106.8, 114.3, 127.2, 139.3, 142.1, 166.2. Mass Spectrometry: HRMS (ESI-TOF) (m/z): Calcd for $C_8H_{10}ClN_2O_2$ ([M + H]$^+$) 201.0425, found 201.0428.

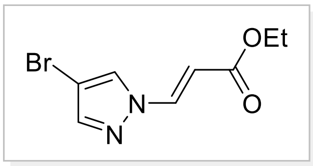

Ethyl (E)-3-(4-bromo-1H-pyrazol-1-yl)acrylate (3j). Eluent: petroleum ether (60-90 °C)/ ethyl acetate = 10:1 (v/v). White solid, 51.9 mg, yield 85%. m.p: 100.4 ºC. NMR Spectroscopy: ^{1}H NMR ($CDCl_3$, 400 MHz) δ 1.33 (t, *J* = 7.1 Hz, 3H), 4.26 (q, *J* = 7.1 Hz, 2H), 6.32 (d, *J* = 14.0 Hz, 1H), 7.66 (s, 1H), 7.67 (s, 1H), 7.89 (d, *J* = 14.0 Hz, 1H). ^{13}C NMR ($CDCl_3$, 100 MHz) δ 14.4,

61.0, 97.7, 106.8, 129.6, 139.1, 144.0, 166.2. Mass Spectrometry: HRMS (ESI-TOF) (m/z): Calcd for $C_8H_{10}BrN_2O_2$ ($[M + H]^+$) 244.9920, found 244.9920.

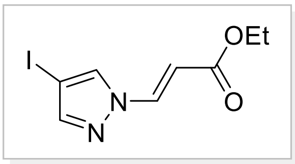

Ethyl (E)-3-(4-iodo-1H-pyrazol-1-yl)acrylate (3k). Eluent: petroleum ether (60-90 °C)/ ethyl acetate = 10:1 (v/v). White solid, 61.3 mg, yield 84%. m.p: 119 ºC. NMR Spectroscopy: ^{1}H NMR ($CDCl_3$, 400 MHz) δ 1.32 (t, J = 7.1 Hz, 3H), 4.26 (q, J = 7.1 Hz, 2H), 6.33 (d, J = 14.0 Hz, 1H), 7.69 (s, 1H), 7.73 (s, 1H), 7.91 (d, J = 14.0 Hz, 1H). ^{13}C NMR ($CDCl_3$, 100 MHz) δ 14.4, 60.9, 61.2, 106.8, 134.1, 138.7, 148.2, 166.3. Mass Spectrometry: HRMS (ESI-TOF) (m/z): Calcd for $C_8H_{10}IN_2O_2$ ($[M + H]^+$) 292.9781, found 292.9778.

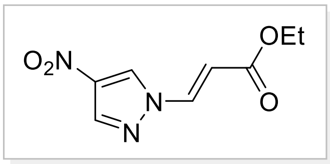

Ethyl (E)-3-(4-nitro-1H-pyrazol-1-yl)acrylate (3l). Eluent: petroleum ether (60-90 °C)/ ethyl acetate = 10:1 (v/v). White solid, 42.7 mg, yield 81%. m.p: 139.6 ºC. NMR Spectroscopy: ^{1}H NMR ($CDCl_3$, 400 MHz) δ 1.35 (t, J = 7.1 Hz, 3H), 4.30 (q, J = 7.1 Hz, 2H), 6.61 (d, J = 13.9 Hz, 1H), 7.90 (d, J = 13.9 Hz, 1H), 8.24 (s, 1H), 8.40 (s, 1H). ^{13}C NMR ($CDCl_3$, 100 MHz) δ 14.3, 61.4, 111.7, 128.8, 138.1, 138.3, 165.3. Mass Spectrometry: HRMS (ESI-TOF) (m/z): Calcd for $C_8H_{10}N_3O_4$ ($[M + H]^+$) 212.0666, found 212.0671.

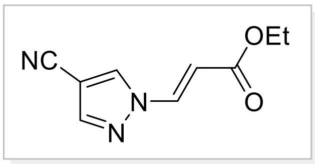

Ethyl (E)-3-(4-cyano-1H-pyrazol-1-yl)acrylate (3m). Eluent: petroleum ether (60-90 °C)/ ethyl acetate = 10:1 (v/v). Colourless oil, 22.0 mg, yield 46%. NMR Spectroscopy: ^{1}H NMR ($CDCl_3$, 400 MHz) δ 1.34 (t, J = 7.1 Hz, 3H), 4.29 (q, J = 7.1 Hz, 2H), 6.56 (d, J = 13.9 Hz, 1H), 7.91 (d, J = 13.9 Hz, 1H), 7.97 (s, 1H), 8.08 (s, 1H). ^{13}C NMR ($CDCl_3$, 100 MHz) δ 14.3, 61.3, 95.9, 110.8, 112.2, 135.1, 137.9, 144.6, 165.5. Mass Spectrometry: HRMS (ESI-TOF) (m/z): Calcd for $C_9H_{10}N_3O_2$ ($[M + H]^+$) 192.0768, found 192.0773.

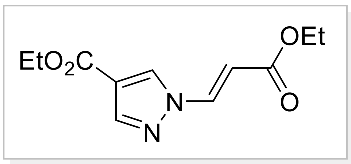

Ethyl (E)-1-(3-ethoxy-3-oxoprop-1-en-1-yl)-1H-pyrazole-4-carboxylate (3n). Eluent: petroleum ether (60-90 °C)/ ethyl acetate = 10:1 (v/v). White solid, 44.6 mg, yield 75%. m.p: 126.3 ºC. NMR Spectroscopy: ^{1}H NMR ($CDCl_3$, 400 MHz) δ 1.31-1.38 (m, 6H), 4.25-4.36 (m, 4H), 6.49 (d, J = 13.9 Hz, 1H), 7.93 (d, J = 13.9 Hz, 1H), 8.07 (s, 1H), 8.13 (s, 1H). ^{13}C NMR ($CDCl_3$, 100 MHz) δ 14.4, 14.5, 60.9, 61.1, 109.0, 118.2, 133.3, 138.9, 144.0, 162.2, 166.0. Mass Spectrometry: HRMS (ESI-TOF) (m/z): Calcd for $C_{11}H_{15}N_2O_4$ ([M + H]$^+$) 239.1026, found 239.1032.

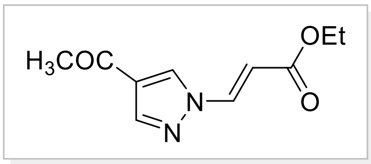

Ethyl (E)-3-(4-acetyl-1H-pyrazol-1-yl)acrylate (3o). Eluent: petroleum ether (60-90 °C)/ ethyl acetate = 10:1 (v/v). White solid, 46.8 mg, yield 90%. m.p: 117.5 ºC. NMR Spectroscopy: ^{1}H NMR ($CDCl_3$, 400 MHz) δ 1.34 (t, J = 7.2 Hz, 3H), 2.48 (s, 3H), 4.28 (q, J = 7.1 Hz, 2H), 6.52 (d, J = 13.9 Hz, 1H), 7.94 (d, J = 13.9 Hz, 1H), 8.08 (s, 1H), 8.13 (s, 1H). ^{13}C NMR ($CDCl_3$, 100 MHz) δ 14.4, 28.2, 61.1, 109.5, 126.3, 132.4, 138.8, 143.2, 165.9, 191.4. Mass Spectrometry: HRMS (ESI-TOF) (m/z): Calcd for $C_{10}H_{13}N_2O_3$ ([M + H]$^+$) 209.0921, found 209.0926.

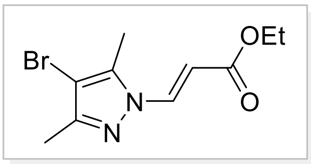

Ethyl (E)-3-(4-bromo-3,5-dimethyl-1H-pyrazol-1-yl)acrylate (3p). Eluent: petroleum ether (60-90 °C)/ ethyl acetate = 10:1 (v/v). White solid, 55.1 mg, yield 81%. m.p: 88 ºC. NMR Spectroscopy: ^{1}H NMR ($CDCl_3$, 400 MHz) δ 1.31 (t, J = 7.1 Hz, 3H), 2.26 (s, 3H), 2.36 (s, 3H), 4.24 (q, J = 7.1 Hz, 2H), 6.43 (d, J = 13.6 Hz, 1H), 7.82 (d, J = 13.6 Hz, 1H). ^{13}C NMR ($CDCl_3$, 100 MHz) δ 10.4, 12.7, 14.5, 60.6, 98.9, 105.6, 136.1, 139.3, 151.4, 167.2. Mass Spectrometry: HRMS (ESI-TOF) (m/z): Calcd for $C_{10}H_{14}BrN_2O_2$ ([M + H]$^+$) 273.0233, found 273.0237.

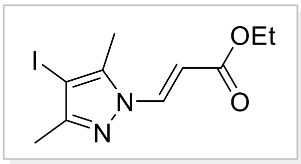

Ethyl (E)-3-(4-iodo-3,5-dimethyl-1H-pyrazol-1-yl)acrylate (3q). Eluent: petroleum ether (60-90 °C)/ ethyl acetate = 10:1 (v/v). White solid, 69.6 mg, yield 87%. m.p: 65 ºC. NMR Spectroscopy: ^{1}H NMR ($CDCl_3$, 400 MHz) δ 1.31 (t, J = 7.1 Hz, 3H), 2.27 (s, 3H), 2.40 (s, 3H), 4.24 (q, J = 7.1 Hz, 2H), 6.42 (d, J = 13.5 Hz, 1H), 7.84 (d, J = 13.6 Hz, 1H). ^{13}C NMR ($CDCl_3$, 100 MHz) δ 12.1, 14.5, 14.5, 60.6, 68.2, 105.7, 136.1, 143.0, 154.3, 167.2. Mass Spectrometry:

HRMS (ESI-TOF) (m/z): Calcd for $C_{10}H_{14}IN_2O_2$ ([M + H]$^+$) 321.0094, found 321.0096.

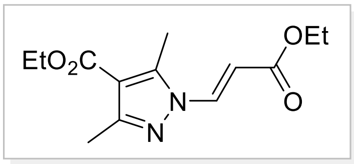

Ethyl (E)-1-(3-ethoxy-3-oxoprop-1-en-1-yl)-3,5-dimethyl-1H-pyrazole-4-carboxylate (3r). Eluent: petroleum ether (60-90 °C)/ ethyl acetate = 10:1 (v/v). White solid, 54.5 mg, yield 82%. m.p: 115 ºC. NMR Spectroscopy: ^{1}H NMR ($CDCl_3$, 400 MHz) δ 1.32 (t, *J* = 7.1 Hz, 3H), 1.37 (t, *J* = 7.1 Hz, 3H), 2.45 (s, 3H), 2.64 (s, 3H), 4.26 (q, *J* = 7.2 Hz, 2H), 4.31 (q, *J* = 7.1 Hz, 2H), 6.55 (d, *J* = 13.5 Hz, 1H), 7.88 (d, *J* = 13.5 Hz, 1H). ^{13}C NMR ($CDCl_3$, 100 MHz) δ 10.9, 14.4, 14.4, 14.7, 60.3, 60.8, 108.1, 112.9, 135.1, 146.2, 154.2, 163.9, 166.9. Mass Spectrometry: HRMS (ESI-TOF) (m/z): Calcd for $C_{13}H_{19}N_2O_4$ ([M + H]$^+$) 267.1339, found 267.1341.

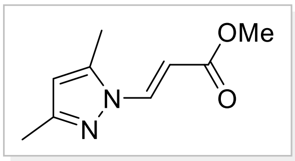

Methyl (E)-3-(3,5-dimethyl-1H-pyrazol-1-yl)acrylate (3s). Eluent: petroleum ether (60-90 °C)/ ethyl acetate = 10:1 (v/v). Colourless oil, 40.1 mg, yield 89%. NMR Spectroscopy: ^{1}H NMR ($CDCl_3$, 400 MHz) δ 2.26 (s, 3H), 2.34 (s, 3H), 3.78 (s, 3H), 5.95 (s, 1H), 6.39 (d, *J* = 13.6 Hz, 1H), 7.83 (d, *J* = 13.5 Hz, 1H). ^{13}C NMR ($CDCl_3$, 100 MHz) δ 10.9, 13.9, 51.7, 104.1, 109.1, 136.3, 141.7, 153.0, 168.0. Mass Spectrometry: HRMS (ESI-TOF) (m/z): Calcd for $C_9H_{13}N_2O_2$ ([M + H]$^+$) 181.0972, found 181.0977.

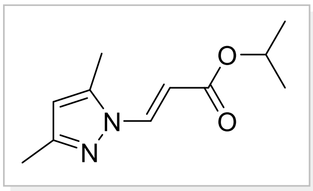

Isopropyl (E)-3-(3,5-dimethyl-1H-pyrazol-1-yl)acrylate (3t). Eluent: petroleum ether (60-90 °C)/ ethyl acetate = 10:1 (v/v). Colourless oil, 41.6 mg, yield 80%. NMR Spectroscopy: ^{1}H NMR ($CDCl_3$, 400 MHz) δ 1.28 (d, *J* = 6.3 Hz, 6H), 2.25 (s, 3H), 2.34 (s, 3H), 5.08-5.14 (m, 1H), 5.95 (s, 1H), 6.37 (d, *J* = 13.6 Hz, 1H), 7.81 (d, *J* = 13.6 Hz, 1H). ^{13}C NMR ($CDCl_3$, 100 MHz) δ 10.9, 13.9, 22.0 (2C), 67.7, 105.2, 108.9, 135.9, 141.6, 152.8, 167.1. Mass Spectrometry: HRMS (ESI-TOF) (m/z): Calcd for $C_{11}H_{17}N_2O_2$ ([M + H]$^+$) 209.1285, found 209.1289.

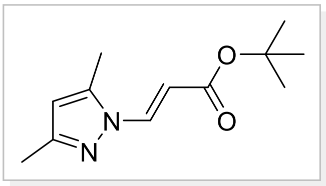

Tert-butyl (E)-3-(3,5-dimethyl-1H-pyrazol-1-yl)acrylate (3u). Eluent: petroleum ether (60-90 °C)/ ethyl acetate = 10:1 (v/v). Colourless oil, 47.2 mg, yield 85%. NMR Spectroscopy: ^{1}H NMR ($CDCl_3$, 400 MHz) δ 1.50 (s, 9H), 2.25 (s, 3H), 2.32 (s, 3H), 5.93 (s, 1H), 6.33 (d, *J* = 13.6 Hz, 1H), 7.75 (d, *J* = 13.6 Hz, 1H). ^{13}C NMR ($CDCl_3$, 100 MHz) δ 10.9, 13.9, 28.3 (3C), 80.4, 106.6, 108.8, 135.4, 141.4, 152.6, 167.0. Mass Spectrometry: HRMS (ESI-TOF) (m/z): Calcd for $C_{12}H_{19}N_2O_2$ ([M + H]$^+$) 223.1441, found 223.1445.

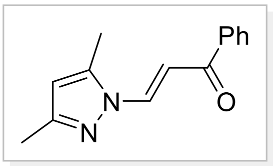

(E)-3-(3,5-dimethyl-1H-pyrazol-1-yl)-1-phenylprop-2-en-1-one (3v). Eluent: petroleum ether (60-90 °C)/ ethyl acetate = 10:1 (v/v). White solid, 52.0 mg, yield 92%. m.p: 103 ºC. NMR Spectroscopy: ^{1}H NMR ($CDCl_3$, 400 MHz) δ 2.30 (d, *J* = 1.6 Hz, 3H), 2.39 (s, 3H), 6.00 (s, 1H), 7.46-7.50 (m, 2H), 7.55-7.60 (m, 2H), 8.02-8.08 (m, 3H). ^{13}C NMR ($CDCl_3$, 100 MHz) δ 11.0, 14.0, 108.0, 109.6, 128.5 (2C), 128.7 (2C), 132.9, 136.3, 138.3, 142.5, 153.4, 189.8. Mass Spectrometry: HRMS (ESI-TOF) (m/z): Calcd for $C_{14}H_{15}N_2O$ ([M + H]$^+$) 227.1179, found 227.1184.

Analytical data of compounds 4

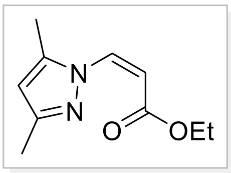

Ethyl (Z)-3-(3,5-dimethyl-1H-pyrazol-1-yl)acrylate (4a). Eluent: petroleum ether (60-90 °C)/ ethyl acetate = 8:1 (v/v). Colourless oil, 43.2 mg, yield 89%. NMR Spectroscopy: ^{1}H NMR ($CDCl_3$, 400 MHz) δ 1.31 (t, *J* = 7.2 Hz, 3H), 2.19 (s, 3H), 2.25 (s, 3H), 4.26 (q, *J* = 7.2 Hz, 2H), 5.52 (d, *J* = 9.7 Hz, 1H), 5.86 (s, 1H), 6.79 (d, *J* = 9.7 Hz, 1H). ^{13}C NMR ($CDCl_3$, 100 MHz) δ 11.1, 13.8, 14.1, 61.0, 107.4, 107.7, 127.8, 140.2, 150.7, 166.7. Mass Spectrometry: HRMS (ESI-TOF) (m/z): Calcd for $C_{10}H_{15}N_2O_2$ ([M + H]$^+$) 195.1128, found 195.1131.

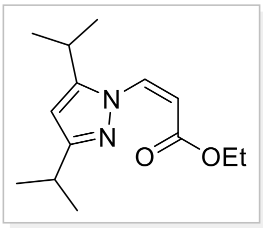

Ethyl (Z)-3-(3,5-diisopropyl-1H-pyrazol-1-yl)acrylate (4b). Eluent: petroleum ether (60-90 °C)/ ethyl acetate = 8:1 (v/v). Colourless oil, 50 mg, yield 80%. NMR Spectroscopy: ^{1}H NMR ($CDCl_3$, 400 MHz) δ 1.21 (s, 3H), 1.23 (s, 3H), 1.24 (s, 3H), 1.26 (s, 3H), 1.30 (t, *J* = 7.2 Hz, 3H), 2.85-2.96 (m, 2H), 4.26 (q, *J* = 7.2 Hz, 2H), 5.49 (d, *J* = 9.7 Hz, 1H), 5.89 (s, 1H), 6.82 (d, *J* = 9.7 Hz, 1H). ^{13}C NMR ($CDCl_3$, 100 MHz) δ 14.2, 22.3 (2C), 22.5 (2C), 25.2, 28.1, 60.9, 101.1, 107.2, 126.7, 150.6, 160.5, 167.2. Mass Spectrometry: HRMS (ESI-TOF) (m/z): Calcd for $C_{14}H_{23}N_2O_2$ ([M + H]$^+$) 251.1754, found 251.1757.

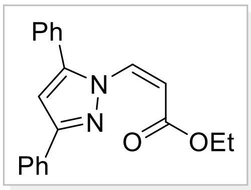

Ethyl (Z)-3-(3,5-diphenyl-1H-pyrazol-1-yl)acrylate (4c). Eluent: petroleum ether (60-90 °C)/ ethyl acetate = 8:1 (v/v). Colourless oil, 64.4 mg, yield 81%. NMR Spectroscopy: ^{1}H NMR ($CDCl_3$, 400 MHz) δ 1.25 (t, *J* = 7.2 Hz, 3H), 4.29 (q, *J* = 7.1 Hz, 2H), 5.59 (d, *J* = 9.7 Hz, 1H), 6.69 (s, 1H), 6.89 (d, *J* = 9.7 Hz, 1H), 7.32 (t, *J* = 7.4 Hz, 1H), 7.39 (t, *J* = 7.2 Hz, 2H), 7.44 (d, *J* = 2.6 Hz, 5H), 7.84 (d, *J* = 7.3 Hz, 2H). ^{13}C NMR ($CDCl_3$, 100 MHz) δ 14.2, 61.1, 104.9, 109.2, 126.0 (2C), 128.4 (2C), 128.6 (2C), 128.9 (2C), 129.1, 129.2 (2C), 129.4, 132.6, 145.6, 152.6, 166.7. Mass Spectrometry: HRMS (ESI-TOF) (m/z): Calcd for $C_{20}H_{19}N_2O_2$ ([M + H]$^+$) 319.1441, found 319.1445.

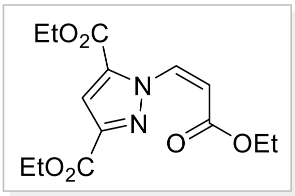

Diethyl (Z)-1-(3-ethoxy-3-oxoprop-1-en-1-yl)-1H-pyrazole-3,5-dicarboxylate (4d). Eluent: petroleum ether (60-90 °C)/ ethyl acetate = 8:1 (v/v). Colourless oil, 58.1 mg, yield 75%. NMR Spectroscopy: ^{1}H NMR ($CDCl_3$, 400 MHz) δ 1.26 (t, J = 7.1 Hz, 3H), 1.37-1.40 (m, 6H), 4.24 (q, J = 7.2 Hz, 2H), 4.34-4.42 (m, 4H), 5.93 (d, J = 9.6 Hz, 1H), 7.40 (s, 1H), 7.77 (d, J = 9.5 Hz, 1H). ^{13}C NMR ($CDCl_3$, 100 MHz) δ 14.1, 14.2, 14.3, 61.3, 61.5, 62.0, 114.6, 115.5, 130.7, 134.5, 144.1, 158.8, 161.2, 165.2. Mass Spectrometry: HRMS (ESI-TOF) (m/z): Calcd for $C_{14}H_{19}N_2O_6$ ($[M + H]^+$) 311.1238, found 311.1241.

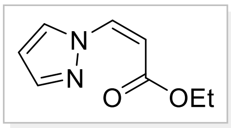

Ethyl (Z)-3-(1H-pyrazol-1-yl)acrylate (4e). Eluent: petroleum ether (60-90 °C)/ ethyl acetate = 8:1 (v/v). Colourless oil, 31.1 mg, yield 75%. NMR Spectroscopy: ^{1}H NMR ($CDCl_3$, 400 MHz) δ 1.32 (t, J = 7.2 Hz, 3H), 4.22 (q, J = 7.1 Hz, 2H), 5.42 (d, J = 11.2 Hz, 1H), 6.41 (t, J = 2.0 Hz, 1H), 7.30 (d, J = 11.1 Hz, 1H), 7.66 (d, J = 1.4 Hz, 1H), 9.11 (s, d, J = 2.7 Hz, 1H). ^{13}C NMR ($CDCl_3$, 100 MHz) δ 14.3, 60.7, 102.4, 108.8, 133.2, 137.9, 142.4, 165.1. Mass Spectrometry: HRMS (ESI-TOF) (m/z): Calcd for $C_8H_{11}N_2O_2$ ($[M + H]^+$) 167.0815, found 167.0817.

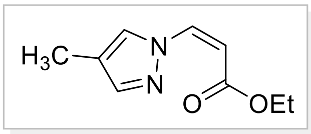

Ethyl (Z)-3-(4-methyl-1H-pyrazol-1-yl)acrylate (4f). Eluent: petroleum ether (60-90 °C)/ ethyl acetate = 8:1 (v/v). Colourless oil, 35.6 mg, yield 79%. NMR Spectroscopy: ^{1}H NMR ($CDCl_3$, 400 MHz) δ 1.32 (t, J = 7.1 Hz, 3H), 2.12 (s, 3H), 4.21 (q, J = 7.2 Hz, 2H), 5.32 (d, J = 11.2 Hz, 1H), 7.21 (d, J = 11.2 Hz, 1H), 7.48 (s, 1H), 8.90 (s, 1H). ^{13}C NMR ($CDCl_3$, 100 MHz) δ 9.1, 29.9, 60.6, 100.7, 119.7, 131.3, 138.2, 143.8, 165.3. Mass Spectrometry: HRMS (ESI-TOF) (m/z): Calcd for $C_9H_{13}N_2O_2$ ($[M + H]^+$) 181.0972, found 181.0975.

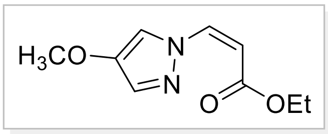

Ethyl (Z)-3-(4-methoxy-1H-pyrazol-1-yl)acrylate (4g). Eluent: petroleum ether (60-90 °C)/ ethyl acetate = 8:1 (v/v). Colourless oil, 39.2 mg, yield 80%. NMR Spectroscopy: ^{1}H NMR ($CDCl_3$, 400 MHz) δ 1.32 (t, J = 7.1 Hz, 3H), 3.81 (s, 3H), 4.21 (q, J = 7.1 Hz, 2H), 5.26 (d, J = 11.2 Hz, 1H),

7.13 (d, J = 11.2 Hz, 1H), 7.45 (s, 1H), 8.90 (s, 1H). ^{13}C NMR ($CDCl_3$, 100 MHz) δ 14.4, 58.9, 60.5, 99.4, 115.3, 133.2, 139.3, 148.8, 165.4. Mass Spectrometry: HRMS (ESI-TOF) (m/z): Calcd for $C_9H_{13}N_2O_3$ ([M + H]$^+$) 197.0921, found 197.0926.

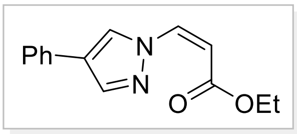

Ethyl (Z)-3-(4-phenyl-1H-pyrazol-1-yl)acrylate (4h). Eluent: petroleum ether (60-90 °C)/ ethyl acetate = 8:1 (v/v). White solid, 43.6 mg, yield 72%. m.p: 65 ºC. NMR Spectroscopy: ^{1}H NMR ($CDCl_3$, 400 MHz) δ 1.34 (t, J = 7.2 Hz, 3H), 4.25 (q, J = 7.2 Hz, 2H), 5.43 (d, J = 11.2 Hz, 1H), 7.28-7.29 (m, 2H), 7.39 (t, J = 7.4 Hz, 2H), 7.56-7.58 (m, 2H), 7.95 (s, 1H), 9.46 (s, 1H). ^{13}C NMR ($CDCl_3$, 100 MHz) δ 14.4, 60.8, 102.1, 125.9, 126.2 (2C), 127.4, 129.1 (2C), 129.4, 131.5, 138.0, 140.4, 165.2. Mass Spectrometry: HRMS (ESI-TOF) (m/z): Calcd for $C_{14}H_{15}N_2O_2$ ([M + H]$^+$) 243.1128, found 243.1134.

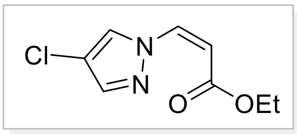

Ethyl (Z)-3-(4-chloro-1H-pyrazol-1-yl)acrylate (4i). Eluent: petroleum ether (60-90 °C)/ ethyl acetate = 8:1 (v/v). Colourless oil, 38.5 mg, yield 77%. NMR Spectroscopy: ^{1}H NMR ($CDCl_3$, 400 MHz) δ 1.32 (t, J = 7.1 Hz, 3H), 4.23 (q, J = 7.1 Hz, 2H), 5.43 (d, J = 11.1 Hz, 1H), 7.16 (d, J = 11.2 Hz, 1H), 7.58 (s, 1H), 9.21 (s, 1H). ^{13}C NMR ($CDCl_3$, 100 MHz) δ 14.3, 60.9, 103.0, 113.5, 130.9, 137.7, 141.0, 164.9. Mass Spectrometry: HRMS (ESI-TOF) (m/z): Calcd for $C_8H_{10}ClN_2O_2$ ([M + H]$^+$) 201.0425, found 201.0427.

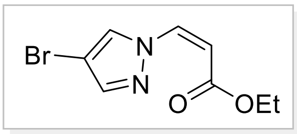

Ethyl (Z)-3-(4-bromo-1H-pyrazol-1-yl)acrylate (4j). Eluent: petroleum ether (60-90 °C)/ ethyl acetate = 8:1 (v/v). Colourless oil, 47.6 mg, yield 78%. NMR Spectroscopy: ^{1}H NMR ($CDCl_3$, 400 MHz) δ 1.32 (t, J = 7.1 Hz, 3H), 4.23 (q, J = 7.2 Hz, 2H), 5.44 (d, J = 11.1 Hz, 1H), 7.18 (d, J = 11.1 Hz, 1H), 7.61 (s, 1H), 9.25 (s, 1H). ^{13}C NMR ($CDCl_3$, 100 MHz) δ 14.3, 61.0, 97.2, 103.2, 133.1, 137.5, 142.9, 164.9. Mass Spectrometry: HRMS (ESI-TOF) (m/z): Calcd for $C_8H_{10}BrN_2O_2$ ([M + H]$^+$) 244.9920, found 244.9921.

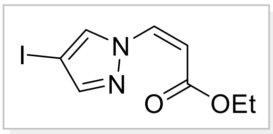

Ethyl (Z)-3-(4-iodo-1H-pyrazol-1-yl)acrylate (4k). Eluent: petroleum ether (60-90 °C)/ ethyl

acetate = 8:1 (v/v). Colourless oil, 59.9 mg, yield 82%. NMR Spectroscopy: ^{1}H NMR ($CDCl_3$, 400 MHz) δ 1.33 (t, *J* = 7.1 Hz, 3H), 4.23 (q, *J* = 7.2 Hz, 2H), 5.42 (d, *J* = 11.1 Hz, 1H), 7.19 (d, *J* = 11.1 Hz, 1H), 7.64 (s, 1H), 9.26 (s, 1H). ^{13}C NMR ($CDCl_3$, 100 MHz) δ 14.3, 61.0, 103.4 (2C), 137.0, 137.4, 147.0, 164.9. Mass Spectrometry: HRMS (ESI-TOF) (m/z): Calcd for $C_8H_{10}IN_2O_2$ ([M + H]$^+$) 292.9781, found 292.9786.

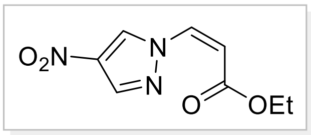

Ethyl (Z)-3-(4-nitro-1H-pyrazol-1-yl)acrylate (4l). Eluent: petroleum ether (60-90 °C)/ ethyl acetate = 8:1 (v/v). White solid, 38.5 mg, yield 73%. m.p: 139.6 ºC. NMR Spectroscopy: ^{1}H NMR ($CDCl_3$, 400 MHz) δ 1.33-1.36 (m, 3H), 4.25-4.31 (m, 2H), 5.72 (d, *J* = 11.0 Hz, 1H), 7.21-7.24 (m, 1H), 8.19 (s, 1H), 9.86 (s, 1H). ^{13}C NMR ($CDCl_3$, 100 MHz) δ 14.2, 61.6, 108.3 (2C), 132.4, 136.8, 137.2, 164.2. Mass Spectrometry: HRMS (ESI-TOF) (m/z): Calcd for $C_8H_{10}N_3O_4$ ([M + H]$^+$) 212.0666, found 212.0668.

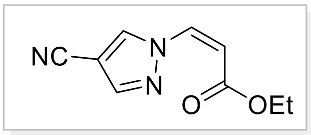

Ethyl (Z)-3-(4-cyano-1H-pyrazol-1-yl)acrylate (4m). Eluent: petroleum ether (60-90 °C)/ ethyl acetate = 8:1 (v/v). Colourless oil, 19.1 mg, yield 40%. NMR Spectroscopy: ^{1}H NMR ($CDCl_3$, 400 MHz) δ 1.34 (t, *J* = 7.2 Hz, 3H), 4.26 (q, *J* = 7.2 Hz, 2H), 5.65 (d, *J* = 11.0 Hz, 1H), 7.25 (d, *J* = 11.2 Hz, 1H), 7.89 (s, 1H), 9.63 (s, 1H). ^{13}C NMR ($CDCl_3$, 100 MHz) δ 14.2, 61.4, 95.4, 107.2, 112.7, 136.4, 138.8, 143.2, 164.4. Mass Spectrometry: HRMS (ESI-TOF) (m/z): Calcd for $C_9H_{10}N_3O_2$ ([M + H]$^+$) 192.0768, found 192.0769.

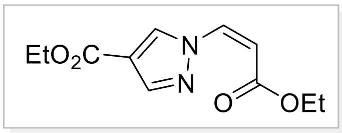

Ethyl (Z)-1-(3-ethoxy-3-oxoprop-1-en-1-yl)-1H-pyrazole-4-carboxylate (4n). Eluent: petroleum ether (60-90 °C)/ ethyl acetate = 8:1 (v/v). White solid, 51.2 mg, yield 86%. m.p: 66 ºC. NMR Spectroscopy: ^{1}H NMR ($CDCl_3$, 400 MHz) δ 1.31-1.38 (m, 6H), 4.26 (q, *J* = 7.1 Hz, 2H), 4.33 (q, *J* = 7.1 Hz, 2H), 5.57 (d, *J* = 11.0 Hz, 1H), 7.24 (d, *J* = 11.1 Hz, 1H), 8.03 (s, 1H), 9.47 (s, 1H). ^{13}C NMR ($CDCl_3$, 100 MHz) δ 14.2, 14.5, 60.7, 61.1, 105.7, 117.7, 136.2, 137.1, 142.9, 162.5, 164.6. Mass Spectrometry: HRMS (ESI-TOF) (m/z): Calcd for $C_{11}H_{15}N_2O_4$ ([M + H]$^+$) 239.1026, found 239.1032.

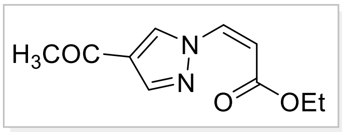

Ethyl (Z)-3-(4-acetyl-1H-pyrazol-1-yl)acrylate (4o). Eluent: petroleum ether (60-90 °C)/ ethyl acetate = 8:1 (v/v). White solid, 44.7 mg, yield 86%. m.p: 162.5 ºC. NMR Spectroscopy: ^{1}H NMR ($CDCl_3$, 400 MHz) δ 1.34 (t, *J* = 7.1 Hz, 3H), 2.49 (s, 3H), 4.26 (q, *J* = 7.1 Hz, 2H), 5.58 (d, *J* = 11.1 Hz, 1H), 7.27 (d, *J* = 11.1 Hz, 1H), 8.04 (s, 1H), 9.62 (s, 1H). ^{13}C NMR ($CDCl_3$, 100 MHz) δ 14.3, 28.3, 61.2, 105.6, 126.0, 136.0, 137.4, 141.9, 164.7, 192.0. Mass Spectrometry: HRMS (ESI-TOF) (m/z): Calcd for $C_{10}H_{13}N_2O_3$ ($[M + H]^+$) 209.0921, found 209.0925.

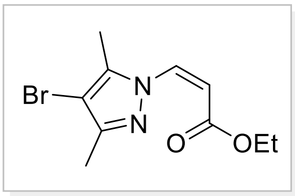

Ethyl (Z)-3-(4-bromo-3,5-dimethyl-1H-pyrazol-1-yl)acrylate (4p). Eluent: petroleum ether (60-90 °C)/ ethyl acetate = 8:1 (v/v). Colourless oil, 47.6 mg, yield 70%. NMR Spectroscopy: ^{1}H NMR ($CDCl_3$, 400 MHz) δ 1.29 (t, *J* = 7.2 Hz, 3H), 2.20 (s, 3H), 2.26 (s, 3H), 4.24 (q, *J* = 7.2 Hz, 2H), 5.59 (d, *J* = 9.6 Hz, 1H), 6.81 (d, *J* = 9.6 Hz, 1H). ^{13}C NMR ($CDCl_3$, 100 MHz) δ 10.5, 12.6, 14.1, 61.1, 97.3, 109.5, 128.3, 138.1, 149.1, 166.1. Mass Spectrometry: HRMS (ESI-TOF) (m/z): Calcd for $C_{10}H_{14}BrN_2O_2$ ($[M + H]^+$) 273.0233, found 273.0235.

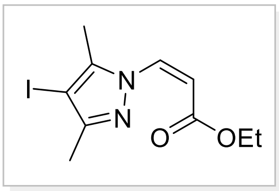

Ethyl (Z)-3-(4-iodo-3,5-dimethyl-1H-pyrazol-1-yl)acrylate (4q). Eluent: petroleum ether (60-90 °C)/ ethyl acetate = 8:1 (v/v). Colourless oil, 66.4 mg, yield 83%. NMR Spectroscopy: ^{1}H NMR ($CDCl_3$, 400 MHz) δ 1.29 (t, *J* = 7.1 Hz, 3H), 2.21 (s, 3H), 2.29 (s, 3H), 4.24 (q, *J* = 7.1 Hz, 2H), 5.57 (d, *J* = 9.6 Hz, 1H), 6.85 (d, *J* = 9.6 Hz, 1H). ^{13}C NMR ($CDCl_3$, 100 MHz) δ 12.2, 14.1, 14.3, 61.1, 66.3, 109.6, 128.4, 141.7, 152.1, 166.0. Mass Spectrometry: HRMS (ESI-TOF) (m/z): Calcd for $C_{10}H_{14}IN_2O_2$ ($[M + H]^+$) 321.0094, found 321.0094.

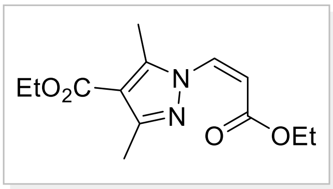

Ethyl (Z)-1-(3-ethoxy-3-oxoprop-1-en-1-yl)-3,5-dimethyl-1H-pyrazole-4-carboxylate (4r). Eluent: petroleum ether (60-90 °C)/ ethyl acetate = 8:1 (v/v). White solid, 52.5 mg, yield 79%. m.p: 68 ºC.

NMR Spectroscopy: ^{1}H NMR ($CDCl_3$, 400 MHz) δ 1.29 (t, *J* = 7.2 Hz, 3H), 1.36 (t, *J* = 7.1 Hz, 3H), 2.53 (s, 3H), 2.40 (s, 3H), 4.22-4.32 (m, 4H), 5.75 (d, *J* = 9.6 Hz, 1H), 6.88 (d, *J* = 9.6 Hz, 1H). ^{13}C NMR ($CDCl_3$, 100 MHz) δ 11.3, 14.1, 14.5, 14.6, 60.0, 61.2, 111.4, 112.4, 127.9, 145.1, 152.3, 164.2, 165.8. Mass Spectrometry: HRMS (ESI-TOF) (m/z): Calcd for $C_{13}H_{19}N_2O_4$ ([M + H]$^+$) 267.1339, found 267.1341.

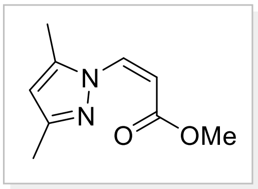

Methyl (Z)-3-(3,5-dimethyl-1H-pyrazol-1-yl)acrylate (4s). Eluent: petroleum ether (60-90 °C)/ ethyl acetate = 8:1 (v/v). Colourless oil, 36 mg, yield 80%. NMR Spectroscopy: ^{1}H NMR ($CDCl_3$, 400 MHz) δ 2.20 (s, 3H), 2.25 (s, 3H), 3.78 (s, 3H), 5.52 (d, *J* = 9.7 Hz, 1H), 5.87 (s, 1H), 6.80 (d, *J* = 9.7 Hz, 1H). ^{13}C NMR ($CDCl_3$, 100 MHz) δ 11.1, 13.9, 52.0, 107.0, 107.5, 128.0, 140.3, 150.9, 167.3. Mass Spectrometry: HRMS (ESI-TOF) (m/z): Calcd for $C_9H_{13}N_2O_2$ ([M + H]$^+$) 181.0972, found 181.0975.

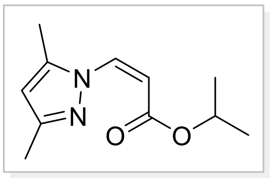

Isopropyl (Z)-3-(3,5-dimethyl-1H-pyrazol-1-yl)acrylate (4t). Eluent: petroleum ether (60-90 °C)/ ethyl acetate = 8:1 (v/v). Colourless oil, 36.4 mg, yield 70%. NMR Spectroscopy: ^{1}H NMR ($CDCl_3$, 400 MHz) δ 1.30 (s, 3H), 1.31 (s, 3H), 2.19 (s, 3H), 2.25 (s, 3H), 5.11-5.19 (m, 1H), 5.51 (d, *J* = 9.7 Hz, 1H), 5.86 (s, 1H), 6.76 (d, *J* = 9.7 Hz, 1H). ^{13}C NMR ($CDCl_3$, 100 MHz) δ 11.1, 13.8, 21.8 (2C), 68.5, 107.3, 108.2, 127.2, 140.0, 150.7, 166.3. Mass Spectrometry: HRMS (ESI-TOF) (m/z): Calcd for $C_{11}H_{17}N_2O_2$ ([M + H]$^+$) 209.1285, found 209.1289.

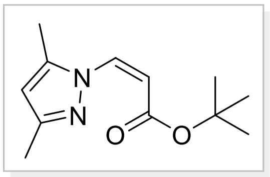

Tert-butyl (Z)-3-(3,5-dimethyl-1H-pyrazol-1-yl)acrylate (4u). Eluent: petroleum ether (60-90 °C)/ ethyl acetate = 8:1 (v/v). Colourless oil, 39.4 mg, yield 71%. NMR Spectroscopy: ^{1}H NMR ($CDCl_3$, 400 MHz) δ 1.52 (s, 9H), 2.20 (s, 3H), 2.34 (s, 3H), 5.50 (d, *J* = 9.7 Hz, 1H), 5.85 (s, 1H), 6.72 (d, *J* = 9.7 Hz, 1H). ^{13}C NMR ($CDCl_3$, 100 MHz) δ 11.1, 13.7, 28.1 (3C), 81.4, 107.1, 110.1, 127.0, 140.0, 150.4, 165.6. Mass Spectrometry: HRMS (ESI-TOF) (m/z): Calcd for $C_{12}H_{19}N_2O_2$ ([M + H]$^+$) 223.1441, found 223.1445.

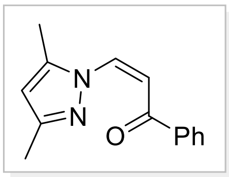

(Z)-3-(3,5-dimethyl-1H-pyrazol-1-yl)-1-phenylprop-2-en-1-one (4v). Eluent: petroleum ether (60-90 °C)/ ethyl acetate = 8:1 (v/v). White solid, 49.2 mg, yield 87%. m.p: 96.5 ºC. NMR Spectroscopy: ^{1}H NMR ($CDCl_3$, 400 MHz) δ 2.31 (s, 3H), 2.40 (s, 3H), 6.01 (s, 1H), 7.47-7.51 (m, 2H), 7.55-7.60 (m, 2H), 8.03-8.09 (m, 3H). ^{13}C NMR ($CDCl_3$, 100 MHz) δ 11.0, 14.0, 108.0, 109.6, 128.5 (2C), 128.7 (2C), 132.9, 136.3, 138.3, 142.5, 153.4, 189.8. Mass Spectrometry: HRMS (ESI-TOF) (m/z): Calcd for $C_{14}H_{15}N_2O$ ($[M + H]^+$) 227.1179, found 227.1184.

III. Synthetic procedures and analytical data of compounds 5, 6

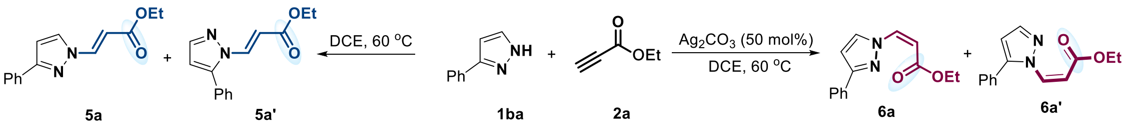

General procedure for the synthesis of 5 (taking 5a and 5a’ as an example): To a solution of 3-phenyl-1H-pyrazole (**1ba**) (72.0 mg, 0.5 mmol) and ethyl propionate (**2a**) (101.5 μL, 1.0 mmol) in DCE (1 ml) at 60 ºC. After the reaction was finished as indicated by TLC (reaction time, 8 h), the resulting mixture was poured into water (10 mL) and extracted with DCM (CH_2Cl_2, 10 mL×3). The combined organic layer was dried over anhydrous Na_2SO_4 and concentrated *in vacuo*. Purification of the crude product with flash column chromatography (petroleum ether (60-90 ºC)/ ethyl acetate = 10:1, v/v) to give **5a** (79.8 mg, 66%) and **5a’** (24.2 mg, 20%).

General procedure for the synthesis of 6 (taking 6a and 6a’ as an example): To a solution of 3-phenyl-1H-pyrazole (**1ba**) (72.0 mg, 0.5 mmol) and ethyl propionate (**2a**) (101.5 μL, 1.0 mmol) in DCE (1 ml) at 60 ºC was added Ag_2CO_3 (69.0 mg, 0.25 mmol). After the reaction was finished as indicated by TLC (reaction time, 12 h), the resulting mixture was poured into water (10 mL) and extracted with DCM (CH_2Cl_2, 10 mL×3). The combined organic layer was dried over anhydrous Na_2SO_4 and concentrated *in vacuo*. Purification of the crude product with flash column chromatography (petroleum ether (60-90 ºC)/ ethyl acetate = 8:1, v/v) to give **6a** (86.0 mg, 71%) and **6a’** (21.8 mg, 18%).

Analytical data of compounds 5

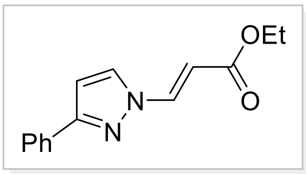

Ethyl (E)-3-(3-phenyl-1H-pyrazol-1-yl)acrylate (5a). Eluent: petroleum ether (60-90 °C)/ ethyl acetate = 10:1 (v/v). White solid, 39.9 mg, yield 66%. m.p: 101.7 ºC. NMR Spectroscopy: ^{1}H NMR ($CDCl_3$, 400 MHz) δ 1.33 (t, *J* = 7.2 Hz, 3H), 4.27 (q, *J* = 7.1 Hz, 2H), 6.45 (d, *J* = 13.9 Hz, 1H), 6.75 (d, *J* = 2.6 Hz, 1H), 7.35-7.39 (m, 1H), 7.41-7.45 (m, 2H), 7.65 (d, *J* = 2.6 Hz, 1H), 7.85-7.87 (m, 2H), 7.97 (d, *J* = 13.9 Hz, 1H). ^{13}C NMR ($CDCl_3$, 100 MHz) δ 14.4, 60.7, 105.7, 106.7, 126.2 (2C), 128.9 (2C), 129.0, 131.7, 132.1, 139.6, 155.2, 166.8. Mass Spectrometry: HRMS (ESI-TOF) (m/z): Calcd for $C_{14}H_{15}N_2O_2$ ([M + H]$^+$) 243.1128, found 243.1134.

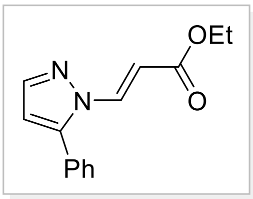

Ethyl (E)-3-(5-phenyl-1H-pyrazol-1-yl)acrylate (5a'). Eluent: petroleum ether (60-90 °C)/ ethyl acetate = 10:1 (v/v). White solid, 12.1 mg, yield 20%. m.p: 65.2 ºC. NMR Spectroscopy: ^{1}H NMR ($CDCl_3$, 400 MHz) δ 1.28 (t, *J* = 7.1 Hz, 3H), 4.21 (q, *J* = 7.1 Hz, 2H), 6.44 (d, *J* = 1.5 Hz, 1H), 6.59 (d, *J* = 13.6 Hz, 1H), 7.39-7.41 (m, 2H), 7.48-7.50 (m, 3H), 7.73 (s, 1H), 7.95 (d, *J* = 13.6 Hz, 1H). ^{13}C NMR ($CDCl_3$, 100 MHz) δ 14.4, 60.6, 107.3, 109.1, 128.9, 129.2 (2C), 129.5, 129.6 (2C), 137.5, 142.9, 145.9, 167.1. Mass Spectrometry: HRMS (ESI-TOF) (m/z): Calcd for $C_{14}H_{15}N_2O_2$ ([M + H]$^+$) 243.1128, found 243.1134.

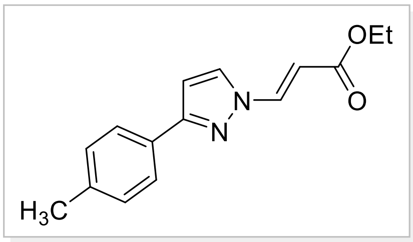

Ethyl (E)-3-(3-(p-tolyl)-1H-pyrazol-1-yl)acrylate (5b). Eluent: petroleum ether (60-90 °C)/ ethyl acetate = 10:1 (v/v). White solid, 41.6 mg, yield 65%. m.p: 129 ºC. NMR Spectroscopy: ^{1}H NMR ($CDCl_3$, 400 MHz) δ 1.33 (t, *J* = 7.1 Hz, 3H), 2.39 (s, 3H), 4.26 (q, *J* = 7.1 Hz, 2H), 6.43 (d, *J* = 13.8 Hz, 1H), 6.73 (d, *J* = 2.6 Hz, 1H), 7.24 (d, *J* = 8.0 Hz, 2H), 7.64 (d, *J* = 2.6 Hz, 1H), 7.75 (d, *J* = 8.1 Hz, 2H), 7.97 (d, *J* = 13.8 Hz, 1H). ^{13}C NMR ($CDCl_3$, 100 MHz) δ 14.5, 21.5, 60.7, 105.4, 106.7, 126.2 (2C), 129.3, 129.6 (2C), 131.7, 139.0, 139.7, 155.3, 166.9. Mass Spectrometry: HRMS (ESI-TOF) (m/z): Calcd for $C_{15}H_{17}N_2O_2$ ([M + H]$^+$) 257.1285, found 257.1290.

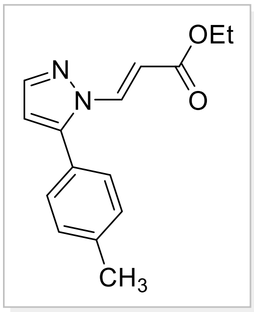

Ethyl (E)-3-(5-(p-tolyl)-1H-pyrazol-1-yl)acrylate (5b'). Eluent: petroleum ether (60-90 °C)/ ethyl acetate = 10:1 (v/v). White solid, 11.5 mg, yield 18%. m.p: 77.5 ºC. NMR Spectroscopy: ^{1}H NMR ($CDCl_3$, 400 MHz) δ 1.28 (t, *J* = 7.1 Hz, 3H), 2.43 (s, 3H), 4.21 (q, *J* = 7.2 Hz, 2H), 6.40 (d, *J* = 1.6 Hz, 1H), 6.57 (d, *J* = 13.6 Hz, 1H), 7.29 (d, *J* = 1.3 Hz, 4H), 7.71 (s, 1H), 7.95 (d, *J* = 13.6 Hz, 1H). ^{13}C NMR ($CDCl_3$, 100 MHz) δ 14.4, 21.5, 60.6, 107.1, 108.8, 126.0, 129.5 (2C), 129.9 (2C), 137.6, 140.0, 142.9, 146.0, 167.2. Mass Spectrometry: HRMS (ESI-TOF) (m/z): Calcd for $C_{15}H_{17}N_2O_2$ ([M + H]$^+$) 257.1285, found 257.1290.

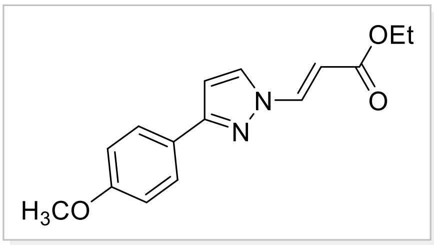

Ethyl (E)-3-(3-(4-methoxyphenyl)-1H-pyrazol-1-yl)acrylate (5c). Eluent: petroleum ether (60-90 °C)/ ethyl acetate = 10:1 (v/v). White solid, 41.5 mg, yield 61%. m.p: 100.1 ºC. NMR Spectroscopy: ^{1}H NMR ($CDCl_3$, 400 MHz) δ 1.29 (t, *J* = 7.1 Hz, 3H), 3.87 (s, 3H), 4.21 (q, *J* = 7.1 Hz, 2H), 6.38 (d, *J* = 1.5 Hz, 1H), 6.56 (d, *J* = 13.6 Hz, 1H), 7.01 (d, *J* = 8.6 Hz, 2H), 7.32 (d, *J* = 8.6 Hz, 2H), 7.70 (s, 1H), 7.93 (d, *J* = 13.6 Hz, 1H). ^{13}C NMR ($CDCl_3$, 100 MHz) δ 14.4, 55.6, 60.6, 107.0, 108.7, 114.6 (2C), 121.1, 130.9 (2C), 137.6, 142.9, 145.8, 160.6, 167.2. Mass Spectrometry: HRMS (ESI-TOF) (m/z): Calcd for $C_{15}H_{17}N_2O_3$ ([M + H]$^+$) 273.1234, found 273.1237.

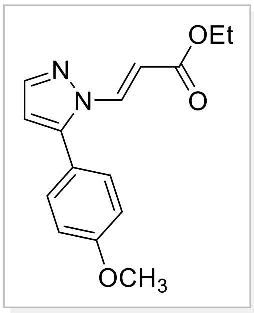

Ethyl (E)-3-(5-(4-methoxyphenyl)-1H-pyrazol-1-yl)acrylate (5c'). Eluent: petroleum ether (60-90 °C)/ ethyl acetate = 10:1 (v/v). White solid, 24.5 mg, yield 36%. m.p: 94.7 ºC. NMR

Spectroscopy: ^{1}H NMR ($CDCl_3$, 400 MHz) δ 1.33 (t, *J* = 7.1 Hz, 3H), 3.85 (s, 3H), 4.26 (q, *J* = 7.1 Hz, 2H), 6.42 (d, *J* = 13.8 Hz, 1H), 6.69 (d, *J* = 2.6 Hz, 1H), 6.94-6.97 (m, 2H), 7.62 (d, *J* = 2.6 Hz, 1H), 7.78-7.81 (m, 2H), 7.95 (d, *J* = 13.8 Hz, 1H). ^{13}C NMR ($CDCl_3$, 100 MHz) δ 14.4, 55.5, 60.6, 105.2, 106.4, 114.3 (2C), 124.8, 127.6 (2C), 131.7, 139.6, 155.1, 160.4, 167.0. Mass Spectrometry: HRMS (ESI-TOF) (m/z): Calcd for $C_{15}H_{17}N_2O_3$ ([M + H]$^+$) 273.1234, found 273.1239.

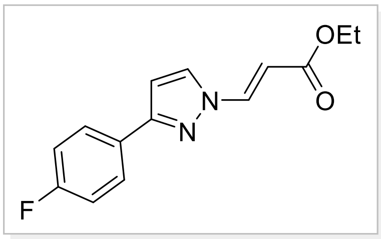

Ethyl (E)-3-(3-(4-fluorophenyl)-1H-pyrazol-1-yl)acrylate (5d). Eluent: petroleum ether (60-90 °C)/ ethyl acetate = 10:1 (v/v). White solid, 47.5 mg, yield 73%. m.p: 91.8 ºC. NMR Spectroscopy: ^{1}H NMR ($CDCl_3$, 400 MHz) δ 1.29 (t, *J* = 7.1 Hz, 3H), 4.22 (q, *J* = 7.1 Hz, 2H), 6.42 (d, *J* = 1.6 Hz, 1H), 6.58 (d, *J* = 13.6 Hz, 1H), 7.18-7.22 (m, 2H), 7.36-7.40 (m, 2H), 7.72 (d, *J* = 0.5 Hz, 1H), 7.87 (d, *J* = 13.6 Hz, 1H). ^{13}C NMR ($CDCl_3$, 100 MHz) δ 14.4, 60.7, 107.6, 109.2, 116.4 (d, *J* = 21.9 Hz), 125.0 (2C), 131.5 (d, *J* = 8.4 Hz), 137.1, 142.9, 144.7, 163.5 (d, *J* = 248.8 Hz, 2C), 167.4. Mass Spectrometry: HRMS (ESI-TOF) (m/z): Calcd for $C_{14}H_{14}FN_2O_2$ ([M + H]$^+$) 261.1034, found 261.1036. Mass Spectrometry: HRMS (ESI-TOF) (m/z): Calcd for $C_{14}H_{14}FN_2O_2$ ([M + H]$^+$) 261.1034, found 261.1036.

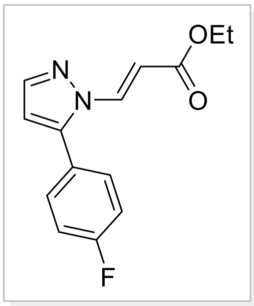

Ethyl (E)-3-(5-(4-fluorophenyl)-1H-pyrazol-1-yl)acrylate (5d'). Eluent: petroleum ether (60-90 °C)/ ethyl acetate = 10:1 (v/v). White solid, 15.6 mg, yield 24%. m.p: 99.4 ºC. NMR Spectroscopy: ^{1}H NMR ($CDCl_3$, 400 MHz) δ 1.34 (t, *J* = 7.1 Hz, 3H), 4.27 (q, *J* = 7.1 Hz, 2H), 6.44 (d, *J* = 13.8 Hz, 1H), 6.71 (d, *J* = 2.5 Hz, 1H), 7.12 (d, *J* = 8.6 Hz, 2H), 7.65 (d, *J* = 2.5 Hz, 1H), 7.82-7.86 (m, 2H), 7.96 (d, *J* = 13.8 Hz, 1H). ^{13}C NMR (CDCl3, 100 MHz) δ 14.5, 60.7, 105.9, 106.5, 115.9 (d, *J* = 21.6 Hz, 2C), 128.1 (d, *J* = 8.3 Hz), 128.4 (2C), 131.9, 139.5, 154.3, 163.4 (d, *J* = 246.7 Hz), 166.8. Mass Spectrometry: HRMS (ESI-TOF) (m/z): Calcd for $C_{14}H_{14}FN_2O_2$ ([M + H]$^+$) 261.1034, found 261.1037.

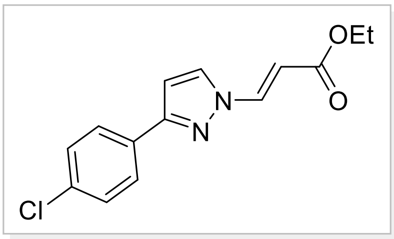

Ethyl (E)-3-(3-(4-chlorophenyl)-1H-pyrazol-1-yl)acrylate (5e). Eluent: petroleum ether (60-90 °C)/ ethyl acetate = 10:1 (v/v). White solid, 40.7 mg, yield 59%. m.p: 124 ºC. NMR Spectroscopy: ^{1}H NMR ($CDCl_3$, 400 MHz) δ 1.34 (t, *J* = 7.1 Hz, 3H), 4.27 (q, *J* = 7.2 Hz, 2H), 6.45 (d, *J* = 13.8 Hz, 1H), 6.73 (d, *J* = 2.6 Hz, 1H), 7.39-7.42 (m, 2H), 7.66 (d, *J* = 2.6 Hz, 1H), 7.79-7.81 (m, 2H), 7.96 (d, *J* = 13.8 Hz, 1H). ^{13}C NMR ($CDCl_3$, 100 MHz) δ 14.5, 60.8, 106.1, 106.6, 127.5 (2C), 129.1 (2C), 130.7, 131.9, 134.9, 139.5, 154.1, 166.8. Mass Spectrometry: HRMS (ESI-TOF) (m/z): Calcd for $C_{14}H_{14}ClN_2O_2$ ([M + H]$^+$) 277.0738, found 277.0730.

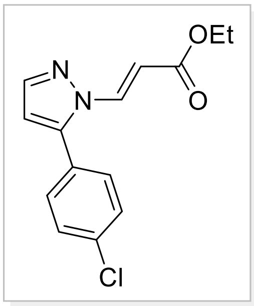

Ethyl (E)-3-(5-(4-chlorophenyl)-1H-pyrazol-1-yl)acrylate (5e'). Eluent: petroleum ether (60-90 °C)/ ethyl acetate = 10:1 (v/v). White solid, 9.7 mg, yield 14%. m.p: 124.7 ºC. NMR Spectroscopy: ^{1}H NMR ($CDCl_3$, 400 MHz) δ 1.29 (t, *J* = 7.1 Hz, 3H), 4.22 (q, *J* = 7.1 Hz, 2H), 6.43 (d, *J* = 1.7 Hz, 1H), 6.59 (d, *J* = 13.6 Hz, 1H), 7.33-7.35 (m, 2H), 7.47-7.49 (m, 2H), 7.73 (s, 1H), 7.85-7.89 (m, 1H). ^{13}C NMR ($CDCl_3$, 100 MHz) δ 14.4, 60.7, 107.7, 109.3, 127.4, 129.5 (2C), 130.9 (2C), 136.0, 137.0, 142.9, 144.6, 167.0. Mass Spectrometry: HRMS (ESI-TOF) (m/z): Calcd for $C_{14}H_{14}ClN_2O_2$ ([M + H]$^+$) 277.0738, found 277.0744.

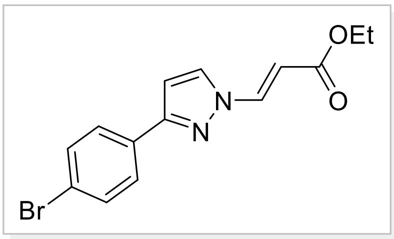

Ethyl (E)-3-(3-(4-bromophenyl)-1H-pyrazol-1-yl)acrylate (5f). Eluent: petroleum ether (60-90 °C)/ ethyl acetate = 10:1 (v/v). White solid, 57.6 mg, yield 72%. m.p: 147.1 ºC. NMR Spectroscopy: ^{1}H NMR ($CDCl_3$, 400 MHz) δ 1.29 (t, *J* = 7.1 Hz, 3H), 4.22 (q, *J* = 7.1 Hz, 2H), 6.43 (d, *J* = 1.7 Hz, 1H), 6.59 (d, *J* = 13.6 Hz, 1H), 7.26-7.28 (m, 2H), 7.63-7.65 (m, 2H), 7.72 (s, 1H), 7.87 (d, *J* = 13.6 Hz, 1H). ^{13}C NMR ($CDCl_3$, 100 MHz) δ 14.4, 60.7, 107.8, 109.2, 124.2,

127.8, 131.1 (2C), 132.4 (2C), 137.0, 142.9, 144.6, 167.0. Mass Spectrometry: HRMS (ESI-TOF) (m/z): Calcd for $C_{14}H_{14}BrN_2O_2$ ($[M + H]^+$) 321.0233, found 321.0238.

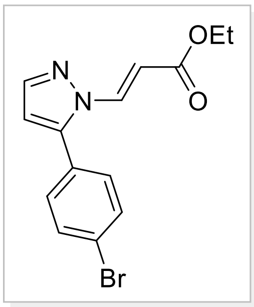

Ethyl (E)-3-(5-(4-bromophenyl)-1H-pyrazol-1-yl)acrylate (5f'). Eluent: petroleum ether (60-90 °C)/ ethyl acetate = 10:1 (v/v). White solid, 19.2 mg, yield 24%. m.p: 136.9 ºC. NMR Spectroscopy: ^{1}H NMR ($CDCl_3$, 400 MHz) δ 1.29 (t, *J* = 7.1 Hz, 3H), 4.22 (q, *J* = 7.1 Hz, 2H), 6.43 (d, *J* = 1.7 Hz, 1H), 6.59 (d, *J* = 13.6 Hz, 1H), 7.26-7.28 (m, 2H), 7.63-7.65 (m, 2H), 7.72 (s, 1H), 7.87 (d, *J* = 13.6 Hz, 1H). ^{13}C NMR ($CDCl_3$, 100 MHz) δ 14.4, 60.7, 107.8, 109.2, 124.2, 127.8, 131.1 (2C), 132.5 (2C), 137.0, 142.9, 144.6, 167.0. Mass Spectrometry: HRMS (ESI-TOF) (m/z): Calcd for $C_{14}H_{14}BrN_2O_2$ ($[M + H]^+$) 321.0233, found 321.0235.

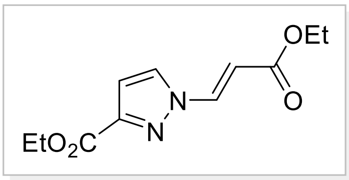

Ethyl (E)-1-(3-ethoxy-3-oxoprop-1-en-1-yl)-1H-pyrazole-3-carboxylate (5g). Eluent: petroleum ether (60-90 °C)/ ethyl acetate = 10:1 (v/v). White solid, 37.5 mg, yield 63%. m.p: 142.0 ºC. NMR Spectroscopy: ^{1}H NMR ($CDCl_3$, 400 MHz) δ 1.32 (t, *J* = 7.1 Hz, 3H), 1.42 (t, *J* = 7.1 Hz, 3H), 4.27 (q, *J* = 7.1 Hz, 2H), 4.44 (q, *J* = 7.2 Hz, 2H), 6.49 (d, *J* = 14.1 Hz, 1H), 6.95 (d, *J* = 2.6 Hz, 1H), 7.71 (d, *J* = 2.6 Hz, 1H), 8.02 (d, *J* = 14.1 Hz, 1H). ^{13}C NMR ($CDCl_3$, 100 MHz) δ 14.3, 14.4, 61.1, 61.7, 109.0, 111.3, 130.8, 139.4, 147.2, 161.7, 165.9. Mass Spectrometry: HRMS (ESI-TOF) (m/z): Calcd for $C_{11}H_{15}N_2O_4$ ($[M + H]^+$) 239.1026, found 239.1021.

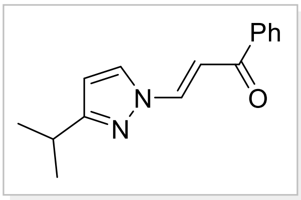

(E)-3-(3-isopropyl-1H-pyrazol-1-yl)-1-phenylprop-2-en-1-one (5h). Eluent: petroleum ether (60-90 °C)/ ethyl acetate = 10:1 (v/v). Colourless oil, 39.5 mg, yield 76% (**5h**+**5h'**). NMR Spectroscopy: ^{1}H NMR ($CDCl_3$, 400 MHz) δ 1.27 (s, 3H), 1.29 (t, *J* = 7.3 Hz, 3H), 1.31 (s, 3H), 2.98-3.06 (m, 1H), 4.24 (q, *J* = 7.1 Hz, 2H), 6.26 (d, *J* = 4.3 Hz, 1H), 6.28 (d, *J* = 7.0 Hz, 1H),

7.54 (d, *J* = 2.6 Hz, 1H), 7.91 (d, *J* = 13.9 Hz, 1H). ^{13}C NMR ($CDCl_3$, 100 MHz) δ 14.4, 22.4 (2C), 28.2, 60.5, 104.4, 106.7, 130.7, 139.8, 163.6, 167.0. Mass Spectrometry: HRMS (ESI-TOF) (m/z): Calcd for $C_{11}H_{17}N_2O_2$ ([M + H]$^+$) 209.1285, found 209.1289.

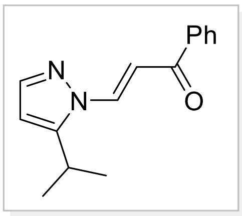

(E)-3-(5-isopropyl-1H-pyrazol-1-yl)-1-phenylprop-2-en-1-one (5h'). Eluent: petroleum ether (60-90 °C)/ ethyl acetate = 10:1 (v/v). Colourless oil, 39.5 mg, yield 76% (**5h**+**5h'**). NMR Spectroscopy: ^{1}H NMR ($CDCl_3$, 400 MHz) δ 1.30 (s, 3H), 1.32 (s, 3H), 1.33 (t, *J* = 4.9 Hz, 3H), 3.08-3.15 (m, 1H), 4.26 (q, *J* = 7.1 Hz, 2H), 6.14 (d, *J* = 1.3 Hz, 1H), 6.53 (d, *J* = 13.5 Hz, 1H), 7.58 (s, 1H), 7.95 (d, *J* = 13.6 Hz, 1H). ^{13}C NMR ($CDCl_3$, 100 MHz) δ 14.4, 22.7 (2C), 25.0, 60.5, 105.0, 106.4, 136.1, 142.9, 151.8, 167.3. Mass Spectrometry: HRMS (ESI-TOF) (m/z): Calcd for $C_{11}H_{17}N_2O_2$ ([M + H]$^+$) 209.1285, found 209.1287.

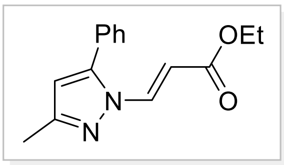

Ethyl (E)-3-(3-methyl-5-phenyl-1H-pyrazol-1-yl)acrylate (5i). Eluent: petroleum ether (60-90 °C)/ ethyl acetate = 10:1 (v/v). White solid, 32 mg, yield 50%. m.p: 121.7 ºC. NMR Spectroscopy: ^{1}H NMR ($CDCl_3$, 400 MHz) δ 1.27 (t, *J* = 6.9 Hz, 3H), 2.36 (s, 3H), 4.19 (q, *J* = 7.1 Hz, 2H), 6.24 (s, 1H), 6.49 (d, *J* = 13.6 Hz, 1H), 7.36-7.39 (m, 2H), 7.46-7.48 (m, 3H), 7.87 (d, *J* = 13.6 Hz, 1H). ^{13}C NMR ($CDCl_3$, 100 MHz) δ 14.0, 14.4, 60.4, 105.8, 109.5, 129.0, 129.1 (2C), 129.4, 129.5 (2C), 137.4, 146.7, 152.8, 167.4. Mass Spectrometry: HRMS (ESI-TOF) (m/z): Calcd for $C_{15}H_{17}N_2O_2$ ([M + H]$^+$) 257.1285, found 257.1288.

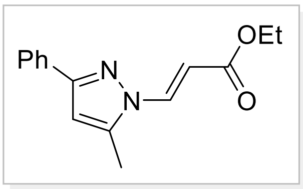

Ethyl (E)-3-(5-methyl-3-phenyl-1H-pyrazol-1-yl)acrylate (5i'). Eluent: petroleum ether (60-90 °C)/ ethyl acetate = 10:1 (v/v). White solid, 16 mg, yield 25%. m.p: 124.7 ºC. NMR Spectroscopy: ^{1}H NMR ($CDCl_3$, 400 MHz) δ 1.33 (t, *J* = 7.2 Hz, 3H), 2.43 (d, *J* = 0.6 Hz, 3H), 4.27 (q, *J* = 7.1 Hz, 2H), 6.47 (d, *J* = 0.6 Hz, 1H), 6.59 (d, *J* = 13.5 Hz, 1H), 7.33-7.37 (m, 1H), 7.39-7.43 (m, 2H), 7.82-7.85 (m, 2H), 7.90 (d, *J* = 13.5 Hz, 1H). ^{13}C NMR ($CDCl_3$, 100 MHz) δ 11.1, 14.5, 60.6, 105.9, 106.2, 126.2 (2C), 128.8 (2C), 128.9, 132.3, 136.2, 142.2, 154.2, 167.4.

Mass Spectrometry: HRMS (ESI-TOF) (m/z): Calcd for $C_{15}H_{17}N_2O_2$ ($[M + H]^+$) 257.1285, found 257.1289.

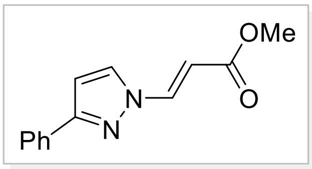

Methyl (E)-3-(3-phenyl-1H-pyrazol-1-yl)acrylate (5j). Eluent: petroleum ether (60-90 °C)/ ethyl acetate = 10:1 (v/v). White solid, 30.2 mg, yield 53%. m.p: 90.2 ºC. NMR Spectroscopy: ^{1}H NMR ($CDCl_3$, 400 MHz) δ 3.75 (s, 3H), 6.44 (d, *J* = 1.6 Hz, 1H), 6.59 (d, *J* = 13.6 Hz, 1H), 7.39-7.41 (m, 2H), 7.48-7.52 (m, 3H), 7.73 (s, 1H), 7.95 (d, *J* = 13.6 Hz, 1H). ^{13}C NMR ($CDCl_3$, 100 MHz) δ 51.8, 106.9, 109.1, 128.9, 129.2 (2C), 129.6 (3C), 137.6, 143.0, 146.0, 167.5. Mass Spectrometry: HRMS (ESI-TOF) (m/z): Calcd for $C_{13}H_{13}N_2O_2$ ($[M + H]^+$) 229.0972, found 229.0979.

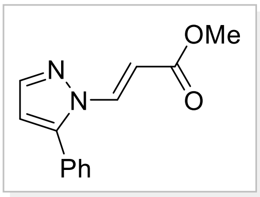

Methyl (E)-3-(5-phenyl-1H-pyrazol-1-yl)acrylate (5j'). Eluent: petroleum ether (60-90 °C)/ ethyl acetate = 10:1 (v/v). White solid, 15.4 mg, yield 27%. m.p: 107.6 ºC. NMR Spectroscopy: ^{1}H NMR ($CDCl_3$, 400 MHz) δ 3.81 (s, 3H), 6.46 (d, *J* = 13.8 Hz, 1H), 6.76 (d, *J* = 2.6 Hz, 1H), 7.35-7.39 (m, 1H), 7.43 (t, *J* = 7.0 Hz, 2H), 7.66 (d, *J* = 2.6 Hz, 1H), 7.86-7.88 (m, 2H), 7.98 (d, *J* = 13.8 Hz, 1H). ^{13}C NMR ($CDCl_3$, 100 MHz) δ 51.9, 105.3, 106.8, 126.3 (2C), 128.9 (2C), 129.1, 131.9, 132.1, 139.8, 155.4, 167.3. Mass Spectrometry: HRMS (ESI-TOF) (m/z): Calcd for $C_{13}H_{13}N_2O_2$ ($[M + H]^+$) 229.0972, found 229.0979.

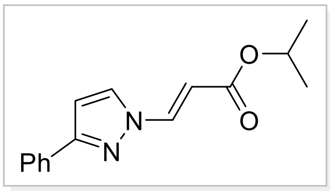

Isopropyl (E)-3-(3-phenyl-1H-pyrazol-1-yl)acrylate (5k). Eluent: petroleum ether (60-90 °C)/ ethyl acetate = 10:1 (v/v). White solid, 35.2 mg, yield 55%. m.p: 98.7 ºC. NMR Spectroscopy: ^{1}H NMR ($CDCl_3$, 400 MHz) δ 1.25 (s, 3H), 1.27 (s, 3H), 5.05-5.11 (m, 1H), 6.43 (d, *J* = 1.6 Hz, 1H), 6.57 (d, *J* = 13.6 Hz, 1H), 7.38-7.41 (m, 2H), 7.47-7.50 (m, 3H), 7.72 (s, 1H), 7.94 (d, *J* = 13.6 Hz, 1H). ^{13}C NMR ($CDCl_3$, 100 MHz) δ 22.0 (2C), 67.9, 107.8, 109.0, 128.9, 129.1 (2C), 129.5, 129.6 (2C), 137.3, 142.8, 145.8, 166.6. Mass Spectrometry: HRMS (ESI-TOF) (m/z): Calcd for $C_{15}H_{17}N_2O_2$ ($[M + H]^+$) 257.1285, found 257.1281.

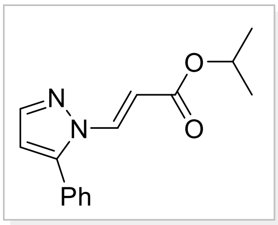

Isopropyl (E)-3-(5-phenyl-1H-pyrazol-1-yl)acrylate (5k'). Eluent: petroleum ether (60-90 °C)/ ethyl acetate = 10:1 (v/v). White solid, 19.2 mg, yield 30%. m.p: 88.5 ºC. NMR Spectroscopy: ^{1}H NMR ($CDCl_3$, 400 MHz) δ 1.30 (s, 3H), 1.32 (s, 3H), 5.11-5.18 (m, 1H), 6.42 (d, *J* = 13.9 Hz, 1H), 6.75 (d, *J* = 2.6 Hz, 1H), 7.35-7.38 (m, 1H), 7.41-7.45 (m, 2H), 7.66 (d, *J* = 2.6 Hz, 1H), 7.85-7.87 (m, 2H), 7.96 (d, *J* = 13.8 Hz, 1H). ^{13}C NMR ($CDCl_3$, 100 MHz) δ 22.1 (2C), 68.0, 106.3, 106.7, 126.2 (2C), 128.9 (2C), 129.0, 131.6, 132.2, 139.5, 155.2, 166.3. Mass Spectrometry: HRMS (ESI-TOF) (m/z): Calcd for $C_{15}H_{17}N_2O_2$ ([M + H]$^+$) 257.1285, found 257.1287.

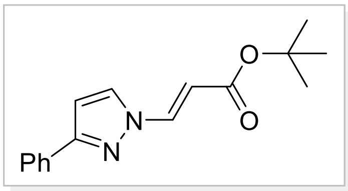

Tert-butyl (E)-3-(3-phenyl-1H-pyrazol-1-yl)acrylate (5l). Eluent: petroleum ether (60-90 °C)/ ethyl acetate = 10:1 (v/v). White solid, 33.8 mg, yield 50%. m.p: 107.2 ºC. NMR Spectroscopy: ^{1}H NMR ($CDCl_3$, 400 MHz) δ 1.48 (s, 9H), 6.42 (d, *J* = 1.5 Hz, 1H), 6.52 (d, *J* = 13.6 Hz, 1H), 7.38-7.41 (m, 2H), 7.46-7.50 (m, 3H), 7.71 (s, 1H), 7.88 (d, *J* = 13.6 Hz, 1H). ^{13}C NMR ($CDCl_3$, 100 MHz) δ 28.3 (3C), 80.7, 108.9, 109.1, 129.0, 129.1 (2C), 129.5, 129.6 (2C), 136.8, 142.7, 145.8, 166.5. Mass Spectrometry: HRMS (ESI-TOF) (m/z): Calcd for $C_{16}H_{19}N_2O_2$ ([M + H]$^+$) 271.1441, found 271.1446.

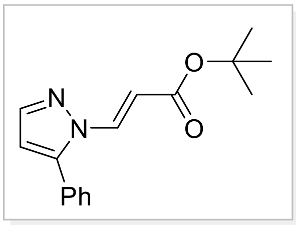

Tert-butyl (E)-3-(5-phenyl-1H-pyrazol-1-yl)acrylate (5l'). Eluent: petroleum ether (60-90 °C)/ ethyl acetate = 10:1 (v/v). White solid, 20.9 mg, yield 31%. m.p: 130.3 ºC. NMR Spectroscopy: ^{1}H NMR ($CDCl_3$, 400 MHz) δ 1.53 (s, 9H), 6.37 (d, *J* = 13.9 Hz, 1H), 6.74 (d, *J* = 2.5 Hz, 1H), 7.34-7.38 (m, 1H), 7.41-7.44 (m, 2H), 7.65 (d, *J* = 2.5 Hz, 1H), 7.85-7.90 (m, 3H). ^{13}C NMR ($CDCl_3$, 100 MHz) δ 28.4 (3C), 80.9, 106.6, 107.7, 126.2 (2C), 128.8 (2C), 128.9, 131.4, 132.2, 139.0, 155.0, 166.1. Mass Spectrometry: HRMS (ESI-TOF) (m/z): Calcd for $C_{16}H_{19}N_2O_2$ ([M + H]$^+$) 271.1441, found 271.1447.

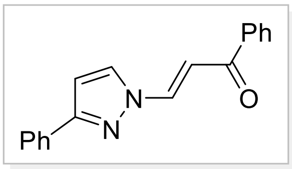

(E)-1-Phenyl-3-(3-phenyl-1H-pyrazol-1-yl)prop-2-en-1-one (5m). Eluent: petroleum ether (60-90 °C)/ ethyl acetate = 10:1 (v/v). White solid, 34.9 mg, yield 51%. m.p: 108.4 ºC. NMR Spectroscopy: ^{1}H NMR (CDCl$_3$, 400 MHz) δ 6.49 (d, *J* = 1.6 Hz, 1H), 7.42-7.45 (m, 2H), 7.47-7.53 (m, 5H), 7.58 (t, *J* = 7.4 Hz, 1H), 7.76-7.79 (m, 2H), 8.04-8.06 (m, 2H), 8.14 (d, *J* = 13.2 Hz, 1H). ^{13}C NMR (CDCl$_3$, 100 MHz) δ 109.4, 110.6, 128.6 (2C), 128.8 (2C), 128.9, 129.3 (2C), 129.7 (2C), 133.1, 137.7, 138.1, 143.1, 146.6, 189.6. Mass Spectrometry: HRMS (ESI-TOF) (m/z): Calcd for $C_{18}H_{15}N_2O$ ([M + H]$^+$) 275.1179, found 275.1181.

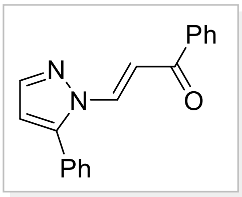

(E)-1-Phenyl-3-(5-phenyl-1H-pyrazol-1-yl)prop-2-en-1-one (5m'). Eluent: petroleum ether (60-90 °C)/ ethyl acetate = 10:1 (v/v). White solid, 16.4 mg, yield 24%. m.p: 167.4 ºC. NMR Spectroscopy: ^{1}H NMR (CDCl$_3$, 400 MHz) δ 6.81 (d, *J* = 2.6 Hz, 1H), 7.41 (d, *J* = 7.2 Hz, 1H), 7.46 (t, *J* = 7.0 Hz, 2H), 7.52 (t, *J* = 7.7 Hz, 2H), 7.60 (t, *J* = 7.3 Hz, 1H), 7.68 (d, *J* = 13.4 Hz, 1H), 7.73 (d, *J* = 2.6 Hz, 1H), 7.91-7.94 (m, 2H), 8.07-8.09 (m, 2H), 8.11 (d, *J* = 13.4 Hz, 1H). ^{13}C NMR (CDCl$_3$, 100 MHz) δ 107.1, 109.4, 126.4 (2C), 128.6 (2C), 128.8 (2C), 129.0 (2C), 129.2, 132.1, 133.1 (2C), 138.2, 140.0, 155.7, 189.7. Mass Spectrometry: HRMS (ESI-TOF) (m/z): Calcd for $C_{18}H_{15}N_2O$ ([M + H]$^+$) 275.1179, found 275.1181.

Analytical data of compounds 6

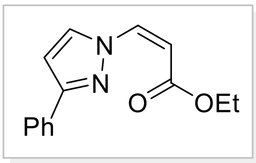

Ethyl (Z)-3-(3-phenyl-1H-pyrazol-1-yl)acrylate (6a). Eluent: petroleum ether (60-90 °C)/ ethyl acetate = 8:1 (v/v). White solid, 43.0 mg, yield 71%. m.p: 68.7 ºC. NMR Spectroscopy: ^{1}H NMR ($CDCl_3$, 400 MHz) δ 1.32 (t, *J* = 7.1 Hz, 3H), 4.23 (q, *J* = 7.1 Hz, 2H), 5.42 (d, *J* = 11.1 Hz, 1H), 6.73 (d, *J* = 2.8 Hz, 1H), 7.32-7.37 (m, 2H), 7.40-7.44 (m, 2H), 7.83-7.85 (m, 2H), 9.14 (d, *J* = 2.8 Hz, 1H). ^{13}C NMR ($CDCl_3$, 100 MHz) δ 14.3, 60.7, 102.1, 106.5, 126.2 (2C), 128.8, 128.9 (2C), 132.2, 134.7, 137.9, 153.9, 165.2. Mass Spectrometry: HRMS (ESI-TOF) (m/z): Calcd for $C_{14}H_{15}N_2O_2$ ($[M + H]^+$) 243.1128, found 243.1126.

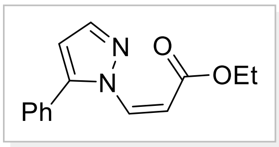

Ethyl (Z)-3-(5-phenyl-1H-pyrazol-1-yl)acrylate (6a'). Eluent: petroleum ether (60-90 °C)/ ethyl acetate = 8:1 (v/v). Colourless oil, 10.9 mg, yield 18%. NMR Spectroscopy: ^{1}H NMR ($CDCl_3$, 400 MHz) δ 1.28 (t, *J* = 7.2 Hz, 3H), 4.25 (q, *J* = 7.2 Hz, 2H), 5.62 (d, *J* = 9.6 Hz, 1H), 6.39 (d, *J* = 1.7 Hz, 1H), 6.90 (d, *J* = 9.6 Hz, 1H), 7.41-7.46 (m, 5H), 7.65 (d, *J* = 1.6 Hz, 1H). ^{13}C NMR ($CDCl_3$, 100 MHz) δ 14.2, 61.0, 107.5, 110.1, 128.9 (2C), 129.1, 129.3 (2C), 129.5, 129.6, 141.3, 144.4, 166.2. Mass Spectrometry: HRMS (ESI-TOF) (m/z): Calcd for $C_{14}H_{15}N_2O_2$ ($[M + H]^+$) 243.1128, found 243.1131.

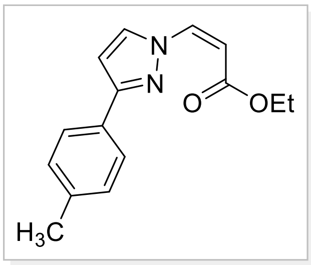

Ethyl (Z)-3-(3-(p-tolyl)-1H-pyrazol-1-yl)acrylate (6b). Eluent: petroleum ether (60-90 °C)/ ethyl acetate = 8:1 (v/v). White solid, 46.7 mg, yield 73%. m.p: 108.1 ºC. NMR Spectroscopy: ^{1}H NMR ($CDCl_3$, 400 MHz) δ 1.32 (t, *J* = 7.1 Hz, 3H), 2.38 (s, 3H), 4.23 (q, *J* = 7.2 Hz, 2H), 5.40 (d, *J* = 11.1 Hz, 1H), 6.70 (d, *J* = 2.7 Hz, 1H), 7.22 (d, *J* = 8.0 Hz, 2H), 7.32 (d, *J* = 11.1 Hz, 1H), 7.72 (d, *J* = 8.1 Hz, 2H), 9.13 (d, *J* = 2.7 Hz, 1H). ^{13}C NMR ($CDCl_3$, 100 MHz) δ 14.3, 21.5, 60.7, 101.8, 106.5, 126.1 (2C), 129.4, 129.6 (2C), 134.6, 138.0, 138.8, 154.0, 165.3. Mass Spectrometry: HRMS (ESI-TOF) (m/z): Calcd for $C_{15}H_{17}N_2O_2$ ($[M + H]^+$) 257.1285, found 257.1287.

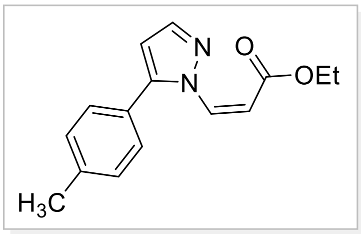

Ethyl (Z)-3-(5-(p-tolyl)-1H-pyrazol-1-yl)acrylate (6b'). Eluent: petroleum ether (60-90 °C)/ ethyl acetate = 8:1 (v/v). Colourless oil, 9.6 mg, yield 15%. NMR Spectroscopy: ^{1}H NMR ($CDCl_3$, 400 MHz) δ 1.29 (t, *J* = 7.1 Hz, 3H), 2.41 (s, 3H), 4.26 (q, *J* = 7.1 Hz, 2H), 5.60 (d, *J* = 9.7 Hz, 1H), 6.35 (d, *J* = 1.6 Hz, 1H), 6.89 (d, *J* = 9.7 Hz, 1H), 7.25-7.27 (m, 2H), 7.31 (d, *J* = 8.1 Hz, 2H), 7.63 (d, *J* = 1.5 Hz, 1H). ^{13}C NMR ($CDCl_3$, 100 MHz) δ 14.2, 21.4, 61.0, 107.2, 109.8, 126.7, 129.2 (2C), 129.6 (2C), 139.2, 141.3, 144.5, 166.3. Mass Spectrometry: HRMS (ESI-TOF) (m/z): Calcd for $C_{15}H_{17}N_2O_2$ ([M + H]$^+$) 257.1285, found 257.1286.

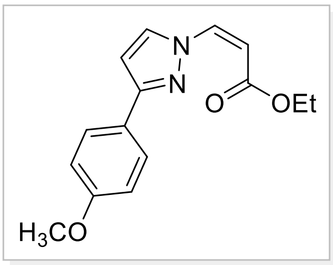

Ethyl (Z)-3-(3-(4-methoxyphenyl)-1H-pyrazol-1-yl)acrylate (6c). Eluent: petroleum ether (60-90 °C)/ ethyl acetate = 8:1 (v/v). White solid, 46.9 mg, yield 68%. m.p: 101.5 ºC. NMR Spectroscopy: ^{1}H NMR ($CDCl_3$, 400 MHz) δ 1.32 (t, *J* = 7.2 Hz, 3H), 3.84 (s, 3H), 4.23 (q, *J* = 7.1 Hz, 2H), 5.39 (d, *J* = 11.1 Hz, 1H), 6.66 (d, *J* = 2.7 Hz, 1H), 6.95 (d, *J* = 8.8 Hz, 2H), 7.31 (d, *J* = 11.1 Hz, 1H), 7.77 (d, *J* = 8.8 Hz, 2H), 9.13 (d, *J* = 2.7 Hz, 1H). ^{13}C NMR ($CDCl_3$, 100 MHz) δ 14.3, 55.4, 60.6, 101.6, 106.3, 114.3 (2C), 124.9, 127.6 (2C), 134.6, 138.0, 153.7, 160.2, 165.3. Mass Spectrometry: HRMS (ESI-TOF) (m/z): Calcd for $C_{15}H_{17}N_2O_3$ ([M + H]$^+$) 273.1234, found 273.1239.

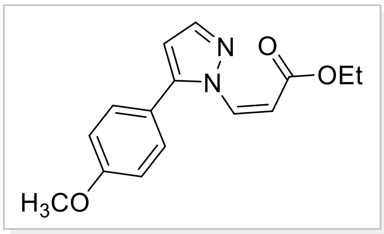

Ethyl (Z)-3-(5-(4-methoxyphenyl)-1H-pyrazol-1-yl)acrylate (6c'). Eluent: petroleum ether (60-90 °C)/ ethyl acetate = 8:1 (v/v). White solid, 10.2 mg, yield 15%. m.p: 100.2 ºC. NMR Spectroscopy: ^{1}H NMR ($CDCl_3$, 400 MHz) δ 1.29 (t, *J* = 7.2 Hz, 3H), 3.85 (s, 3H), 4.26 (q, *J* = 7.2 Hz, 2H), 5.60 (d, *J* = 9.7 Hz, 1H), 6.32 (d, *J* = 1.6 Hz, 1H), 6.88 (d, *J* = 9.7 Hz, 1H), 6.97 (d, *J* = 8.7 Hz, 2H), 7.33-7.36 (m, 2H), 7.62 (d, *J* = 1.5 Hz, 1H). ^{13}C NMR ($CDCl_3$, 100 MHz) δ 14.2,

55.5, 61.0, 107.0, 109.7, 114.4 (2C), 121.9, 129.6, 130.6 (2C), 141.3, 144.3, 160.3, 166.3. Mass Spectrometry: HRMS (ESI-TOF) (m/z): Calcd for $C_{15}H_{17}N_2O_3$ ([M + H]$^+$) 273.1234, found 273.1239.

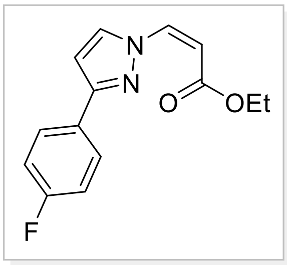

Ethyl (Z)-3-(3-(4-fluorophenyl)-1H-pyrazol-1-yl)acrylate (6d). Eluent: petroleum ether (60-90 °C)/ ethyl acetate = 8:1 (v/v). White solid, 42.3 mg, yield 65%. m.p: 109.1 ºC. NMR Spectroscopy: ^{1}H NMR ($CDCl_3$, 400 MHz) δ 1.33 (t, *J* = 7.1 Hz, 3H), 4.23 (q, *J* = 7.2 Hz, 2H), 5.43 (d, *J* = 11.1 Hz, 1H), 6.68 (d, *J* = 2.7 Hz, 1H), 7.11 (t, *J* = 8.7 Hz, 2H), 7.31 (d, *J* = 11.1 Hz, 1H), 7.79-7.83 (m, 2H), 9.13 (d, *J* = 2.8 Hz, 1H). ^{13}C NMR ($CDCl_3$, 100 MHz) δ 14.3, 60.7, 102.3, 106.3, 115.9 (d, *J* = 21.6 Hz, 2C), 128.0 (d, *J* = 8.3 Hz, 2C), 128.5, 134.8, 137.8, 153.0, 163.3 (d, *J* = 246.3 Hz), 165.2. Mass Spectrometry: HRMS (ESI-TOF) (m/z): Calcd for $C_{14}H_{14}FN_2O_2$ ([M + H]$^+$) 261.1034, found 261.1038.

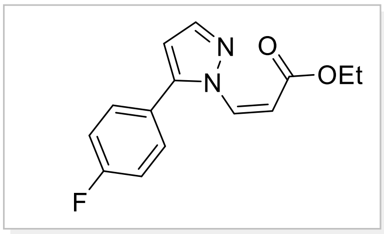

Ethyl (Z)-3-(5-(4-fluorophenyl)-1H-pyrazol-1-yl)acrylate (6d'). Eluent: petroleum ether (60-90 °C)/ ethyl acetate = 8:1 (v/v). White solid, 9.8 mg, yield 15%. m.p: 110.1 ºC. NMR Spectroscopy: ^{1}H NMR ($CDCl_3$, 400 MHz) δ 1.28 (t, *J* = 7.1 Hz, 3H), 4.24 (q, *J* = 7.1 Hz, 2H), 5.64 (d, *J* = 9.6 Hz, 1H), 6.38 (d, *J* = 1.5 Hz, 1H), 6.86 (d, *J* = 9.6 Hz, 1H), 7.15 (t, *J* = 8.6 Hz, 2H), 7.39-7.42 (m, 2H), 7.64 (d, *J* = 1.4 Hz, 1H). ^{13}C NMR ($CDCl_3$, 100 MHz) δ 14.2, 61.1, 107.6, 110.6, 116.1 (d, *J* = 21.7 Hz, 2C), 125.7 (2C), 129.5, 131.2 (d, *J* = 8.4 Hz), 141.4, 143.4, 163.2 (d, *J* = 250.1 Hz), 166.1. Mass Spectrometry: HRMS (ESI-TOF) (m/z): Calcd for $C_{14}H_{14}FN_2O_2$ ([M + H]$^+$) 261.1034, found 261.1038.

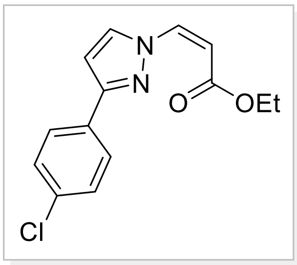

Ethyl (Z)-3-(3-(4-chlorophenyl)-1H-pyrazol-1-yl)acrylate (6e). Eluent: petroleum ether (60-90 °C)/ ethyl acetate = 8:1 (v/v). White solid, 42.8 mg, yield 62%. m.p: 101.5 ºC. NMR Spectroscopy: ^{1}H NMR ($CDCl_3$, 400 MHz) δ 1.32 (t, *J* = 7.1 Hz, 3H), 4.23 (q, *J* = 7.1 Hz, 2H), 5.44 (d, *J* = 11.1 Hz, 1H), 6.69 (d, *J* = 2.8 Hz, 1H), 7.30 (d, *J* = 11.1 Hz, 1H), 7.38-7.41 (m, 2H), 7.76-7.78 (m, 2H), 9.13 (d, *J* = 2.8 Hz, 1H). ^{13}C NMR ($CDCl_3$, 100 MHz) δ 14.3, 60.8, 102.6, 106.4, 127.5 (2C), 129.1 (2C), 130.7, 134.6, 134.8, 137.7, 152.7, 165.2. Mass Spectrometry: HRMS (ESI-TOF) (m/z): Calcd for $C_{14}H_{14}ClN_2O_2$ ([M + H]$^+$) 277.0738, found 277.0741.

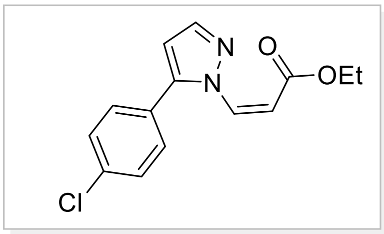

Ethyl (Z)-3-(5-(4-chlorophenyl)-1H-pyrazol-1-yl)acrylate (6e'). Eluent: petroleum ether (60-90 °C)/ ethyl acetate = 8:1 (v/v). Colourless oil, 11.7 mg, yield 17%. NMR Spectroscopy: ^{1}H NMR ($CDCl_3$, 400 MHz) δ 1.28 (t, *J* = 7.1 Hz, 3H), 4.23 (q, *J* = 7.2 Hz, 2H), 5.65 (d, *J* = 9.6 Hz, 1H), 6.39 (d, *J* = 1.7 Hz, 1H), 6.87 (d, *J* = 9.6 Hz, 1H), 7.35-7.38 (m, 2H), 7.43-7.45 (m, 2H), 7.65 (d, *J* = 1.6 Hz, 1H). ^{13}C NMR ($CDCl_3$, 100 MHz) δ 14.2, 61.1, 107.7, 110.9, 128.1, 129.2 (2C), 129.6, 130.5 (2C), 135.4, 141.4, 143.2, 166.0. Mass Spectrometry: HRMS (ESI-TOF) (m/z): Calcd for $C_{14}H_{14}ClN_2O_2$ ([M + H]$^+$) 277.0738, found 277.0741.

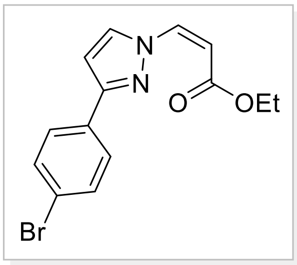

Ethyl (Z)-3-(3-(4-bromophenyl)-1H-pyrazol-1-yl)acrylate (6f). Eluent: petroleum ether (60-90 °C)/ ethyl acetate = 8:1 (v/v). White solid, 52 mg, yield 65%. m.p: 118.5 ºC. NMR Spectroscopy: ^{1}H NMR ($CDCl_3$, 400 MHz) δ 1.32 (t, *J* = 7.2 Hz, 3H), 4.23 (q, *J* = 7.1 Hz, 2H), 5.44 (d, *J* = 11.1 Hz, 1H), 6.69 (d, *J* = 2.8 Hz, 1H), 7.30 (d, *J* = 11.2 Hz, 1H), 7.54 (d, *J* = 8.5 Hz, 2H), 7.70 (d, *J* = 8.5 Hz, 2H), 9.12 (d, *J* = 2.8 Hz, 1H). ^{13}C NMR ($CDCl_3$, 100 MHz) δ 14.3, 60.8, 102.7, 106.3, 122.9, 127.8 (2C), 131.2, 132.0 (2C), 134.8, 137.7, 152.8, 165.2. Mass Spectrometry: HRMS (ESI-TOF) (m/z): Calcd for $C_{14}H_{14}BrN_2O_2$ ([M + H]$^+$) 321.0233, found 321.0237.

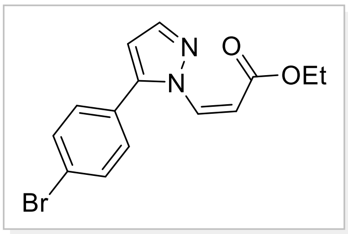

Ethyl (Z)-3-(5-(4-bromophenyl)-1H-pyrazol-1-yl)acrylate (6f'). Eluent: petroleum ether (60-90 °C)/ ethyl acetate = 8:1 (v/v). White solid, 12 mg, yield 15%. m.p: 110.5 ºC. NMR Spectroscopy: ^{1}H NMR ($CDCl_3$, 400 MHz) δ 1.28 (t, *J* = 7.1 Hz, 3H), 4.23 (q, *J* = 7.2 Hz, 2H), 5.65 (d, *J* = 9.6 Hz, 1H), 6.39 (d, *J* = 1.6 Hz, 1H), 6.87 (d, *J* = 9.6 Hz, 1H), 7.30 (d, *J* = 8.4 Hz, 2H), 7.59 (d, *J* = 8.4 Hz, 2H), 7.65 (d, *J* = 1.4 Hz, 1H). ^{13}C NMR ($CDCl_3$, 100 MHz) δ 14.2, 61.1, 107.7, 111.0, 123.6, 128.5, 129.6, 130.7 (2C), 132.2 (2C), 141.4, 143.3, 166.0. Mass Spectrometry: HRMS (ESI-TOF) (m/z): Calcd for $C_{14}H_{14}BrN_2O_2$ ([M + H]$^+$) 321.0233, found 321.0237.

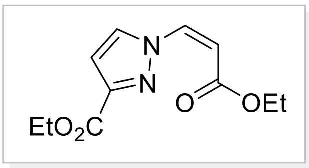

Ethyl (Z)-1-(3-ethoxy-3-oxoprop-1-en-1-yl)-1H-pyrazole-3-carboxylate (6g). Eluent: petroleum ether (60-90 °C)/ ethyl acetate = 8:1 (v/v). White solid, 41.1 mg, yield 69%. m.p: 65 ºC. NMR Spectroscopy: ^{1}H NMR ($CDCl_3$, 400 MHz) δ 1.32 (t, *J* = 7.2 Hz, 3H), 1.41 (t, *J* = 7.1 Hz, 3H), 4.24 (q, *J* = 7.1 Hz, 2H), 4.43 (q, *J* = 7.1 Hz, 2H), 5.59 (d, *J* = 11.1 Hz, 1H), 6.89 (d, *J* = 2.7 Hz, 1H), 7.35 (d, *J* = 11.1 Hz, 1H), 9.09 (d, *J* = 2.7 Hz, 1H). ^{13}C NMR ($CDCl_3$, 100 MHz) δ 14.2, 14.4, 61.1, 61.6, 105.8, 110.5, 134.7, 137.4, 146.0, 161.8, 164.6. Mass Spectrometry: HRMS (ESI-TOF) (m/z): Calcd for $C_{11}H_{15}N_2O_4$ ([M + H]$^+$) 239.1026, found 239.1029.

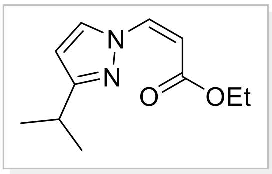

Ethyl (Z)-3-(3-isopropyl-1H-pyrazol-1-yl)acrylate (6h). Eluent: petroleum ether (60-90 °C)/ ethyl acetate = 8:1 (v/v). Colourless oil, 32.2 mg, yield 62%. NMR Spectroscopy: ^{1}H NMR ($CDCl_3$, 400 MHz) δ 1.26 (s, 3H), 1.28 (s, 3H), 1.31 (t, *J* = 7.1 Hz, 3H), 2.96-3.03 (m, 1H), 4.20 (q, *J* = 7.1 Hz, 2H), 5.31 (d, *J* = 11.2 Hz, 1H), 6.25 (d, *J* = 2.7 Hz, 1H), 7.21 (d, *J* = 11.1 Hz, 1H), 9.02 (d, *J* = 2.7 Hz, 1H). ^{13}C NMR ($CDCl_3$, 100 MHz) δ 14.3, 22.4 (2C), 27.9, 60.5, 100.7, 106.5, 133.8, 138.1, 162.0, 165.3. Mass Spectrometry: HRMS (ESI-TOF) (m/z): Calcd for $C_{11}H_{17}N_2O_2$ ([M + H]$^+$) 209.1285, found 209.1287.

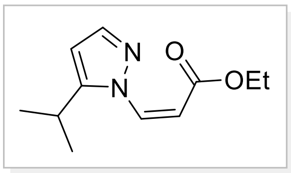

Ethyl (Z)-3-(5-isopropyl-1H-pyrazol-1-yl)acrylate (6h'). Eluent: petroleum ether (60-90 °C)/ ethyl acetate = 8:1 (v/v). Colourless oil, 9.4 mg, yield 18%. NMR Spectroscopy: ^{1}H NMR ($CDCl_3$, 400 MHz) δ 1.26 (s, 3H), 1.27 (t, *J* = 7.1 Hz, 3H), 1.28 (s, 3H), 2.95-3.02 (m, 1H), 4.26 (q, *J* = 7.2 Hz, 2H), 5.63 (d, *J* = 9.6 Hz, 1H), 6.07 (d, *J* = 1.4 Hz, 1H), 6.92 (d, *J* = 9.6 Hz, 1H), 7.49 (d, *J* = 1.4 Hz, 1H). ^{13}C NMR ($CDCl_3$, 100 MHz) δ 14.2, 22.5 (2C), 25.3, 61.0, 103.6, 109.7, 127.9, 141.1, 150.2, 166.5. Mass Spectrometry: HRMS (ESI-TOF) (m/z): Calcd for $C_{11}H_{17}N_2O_2$ ([M + H]$^+$) 209.1285, found 209.1287.

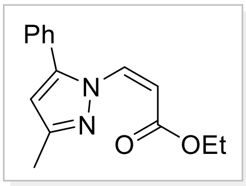

Ethyl (Z)-3-(3-methyl-5-phenyl-1H-pyrazol-1-yl)acrylate (6i). Eluent: petroleum ether (60-90 °C)/ ethyl acetate = 8:1 (v/v). White solid, 36.5 mg, yield 57%. m.p: 120.5 ºC. NMR Spectroscopy: ^{1}H NMR ($CDCl_3$, 400 MHz) δ 1.31 (t, *J* = 7.2 Hz, 3H), 2.29 (s, 3H), 4.25 (q, *J* = 7.1 Hz, 2H), 5.50 (d, *J* = 9.7 Hz, 1H), 6.17 (s, 1H), 6.82 (d, *J* = 9.7 Hz, 1H), 7.38-7.44 (m, 5H). ^{13}C NMR ($CDCl_3$, 100 MHz) δ 13.9, 14.1, 60.9, 107.8, 108.4, 128.8 (2C), 128.9, 128.9, 129.2 (2C), 129.8, 145.1, 150.7, 166.6. Mass Spectrometry: HRMS (ESI-TOF) (m/z): Calcd for $C_{15}H_{17}N_2O_2$ ([M + H]$^+$) 257.1285, found 257.1287.

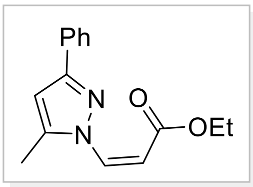

Ethyl (Z)-3-(5-methyl-3-phenyl-1H-pyrazol-1-yl)acrylate (6i'). Eluent: petroleum ether (60-90 °C)/ ethyl acetate = 8:1 (v/v). White solid, 19.2 mg, yield 30%. m.p: 101.5 ºC. NMR Spectroscopy: ^{1}H NMR ($CDCl_3$, 400 MHz) δ 1.25 (t, *J* = 7.1 Hz, 3H), 2.33 (s, 3H), 4.29 (q, *J* = 7.1 Hz, 2H), 5.61 (d, *J* = 9.7 Hz, 1H), 6.40 (s, 1H), 6.86 (d, *J* = 9.7 Hz, 1H), 7.28-7.31 (m, 1H), 7.35-7.39 (m, 2H), 7.75-7.77 (m, 2H). ^{13}C NMR ($CDCl_3$, 100 MHz) δ 11.2, 14.2, 61.2, 104.6, 108.6, 125.9 (2C), 127.3, 128.3, 128.6 (2C), 132.8, 140.8, 152.5, 166.8. Mass Spectrometry: HRMS (ESI-TOF) (m/z): Calcd for $C_{15}H_{17}N_2O_2$ ([M + H]$^+$) 257.1285, found 257.1289.

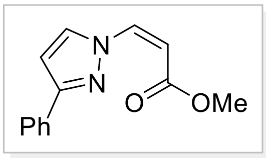

Methyl (Z)-3-(3-phenyl-1H-pyrazol-1-yl)acrylate (6j). Eluent: petroleum ether (60-90 °C)/ ethyl acetate = 8:1 (v/v). White solid, 39.3 mg, yield 69%. m.p: 91.9 ºC. NMR Spectroscopy: ^{1}H NMR ($CDCl_3$, 400 MHz) δ 3.78 (s, 3H), 5.43 (d, J = 11.1 Hz, 1H), 6.74 (d, J = 2.7 Hz, 1H), 7.33-7.38 (m, 2H), 7.43 (t, J = 7.1 Hz, 2H), 7.84 (d, J = 7.2 Hz, 2H), 9.14 (d, J = 2.7 Hz, 1H). ^{13}C NMR ($CDCl_3$, 100 MHz) δ 51.8, 101.6, 106.7, 126.3 (2C), 128.8, 128.9 (2C), 132.2, 134.6, 138.1, 154.0, 165.7. Mass Spectrometry: HRMS (ESI-TOF) (m/z): Calcd for $C_{13}H_{13}N_2O_2$ ($[M + H]^+$) 229.0972, found 229.0977.

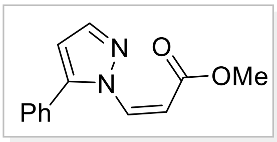

Methyl (Z)-3-(5-phenyl-1H-pyrazol-1-yl)acrylate (6j’). Eluent: petroleum ether (60-90 °C)/ ethyl acetate = 8:1 (v/v). White solid, 12.0 mg, yield 21%. m.p: 89 ºC. NMR Spectroscopy: ^{1}H NMR ($CDCl_3$, 400 MHz) δ 3.78 (s, 3H), 5.61 (d, J = 9.7 Hz, 1H), 6.39 (d, J = 1.7 Hz, 1H), 6.91 (d, J = 9.7 Hz, 1H), 7.41-7.46 (m, 5H), 7.66 (d, J = 1.4 Hz, 1H). ^{13}C NMR ($CDCl_3$, 100 MHz) δ 52.0, 107.6, 109.4, 128.9 (2C), 129.2, 129.3 (2C), 129.5, 129.7, 141.5, 144.5, 166.7. Mass Spectrometry: HRMS (ESI-TOF) (m/z): Calcd for $C_{13}H_{13}N_2O_2$ ($[M + H]^+$) 229.0972, found 229.0977.

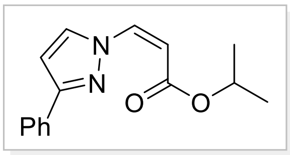

Isopropyl (Z)-3-(3-phenyl-1H-pyrazol-1-yl)acrylate (6k). Eluent: petroleum ether (60-90 °C)/ ethyl acetate = 8:1 (v/v). White solid, 39.7 mg, yield 62%. m.p: 75 ºC. NMR Spectroscopy: ^{1}H NMR ($CDCl_3$, 400 MHz) δ 1.29 (s, 3H), 1.31 (s, 3H), 5.06-5.12 (m, 1H), 5.40 (d, J = 11.1 Hz, 1H), 6.73 (d, J = 2.7 Hz, 1H), 7.32 (d, J = 11.2 Hz, 1H), 7.35-7.37 (m, 1H), 7.42 (t, J = 7.0 Hz, 2H), 7.83-7.85 (m, 2H), 9.15 (d, J = 2.8 Hz, 1H). ^{13}C NMR ($CDCl_3$, 100 MHz) δ 22.0 (2C), 68.2, 102.7, 106.4, 126.3 (2C), 128.8, 128.9 (2C), 132.2, 134.7, 137.7, 153.8, 164.8. Mass Spectrometry: HRMS (ESI-TOF) (m/z): Calcd for $C_{15}H_{17}N_2O_2$ ($[M + H]^+$) 257.1285, found 257.1289.

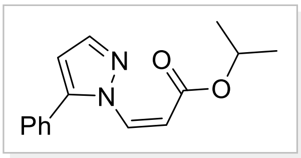

Isopropyl (Z)-3-(5-phenyl-1H-pyrazol-1-yl)acrylate (6k’). Eluent: petroleum ether (60-90 °C)/

ethyl acetate = 8:1 (v/v). White solid, 11.5 mg, yield 18%. m.p: 101.5 ºC. NMR Spectroscopy: ^{1}H NMR ($CDCl_3$, 400 MHz) δ 1.27 (s, 3H), 1.29 (s, 3H), 5.13-5.19 (m, 1H), 5.61 (d, *J* = 9.6 Hz, 1H), 6.38 (s, 1H), 6.88 (d, *J* = 9.6 Hz, 1H), 7.42-7.45 (m, 5H), 7.63 (s, 1H). ^{13}C NMR ($CDCl_3$, 100 MHz) δ 21.8 (2C), 68.6, 107.5, 110.7, 128.9 (2C), 129.0, 129.1, 129.3 (2C), 129.7, 141.2, 144.3, 165.7. Mass Spectrometry: HRMS (ESI-TOF) (m/z): Calcd for $C_{15}H_{17}N_2O_2$ ([M + H]$^+$) 257.1285, found 257.1291.

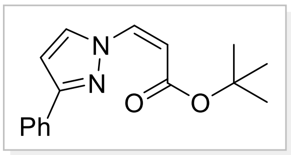

Tert-butyl (Z)-3-(3-phenyl-1H-pyrazol-1-yl)acrylate (6l). Eluent: petroleum ether (60-90 °C)/ ethyl acetate = 8:1 (v/v). White solid, 45.2 mg, yield 67%. m.p: 91.4 ºC. NMR Spectroscopy: ^{1}H NMR ($CDCl_3$, 400 MHz) δ 1.52 (s, 9H), 5.36 (d, *J* = 11.1 Hz, 1H), 6.71 (d, *J* = 2.7 Hz, 1H), 7.25 (t, *J* = 6.6 Hz, 1H), 7.33-7.37 (m, 1H), 7.42 (t, *J* = 7.1 Hz, 2H), 7.82-7.85 (m, 2H), 9.09 (d, *J* = 2.8 Hz, 1H). ^{13}C NMR ($CDCl_3$, 100 MHz) δ 28.3 (3C), 81.2, 104.4, 106.3, 126.2 (2C), 128.7, 128.8 (2C), 132.3, 134.7, 137.0, 153.6, 164.6. Mass Spectrometry: HRMS (ESI-TOF) (m/z): Calcd for $C_{16}H_{19}N_2O_2$ ([M + H]$^+$) 271.1441, found 271.1445.

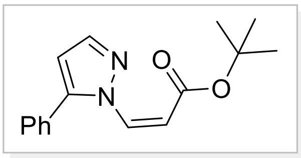

Tert-butyl (Z)-3-(5-phenyl-1H-pyrazol-1-yl)acrylate (6l'). Eluent: petroleum ether (60-90 °C)/ ethyl acetate = 8:1 (v/v). White solid, 9.5 mg, yield 14%. m.p: 89.5 ºC. NMR Spectroscopy: ^{1}H NMR ($CDCl_3$, 400 MHz) δ 1.50 (s, 9H), 5.61 (d, *J* = 9.6 Hz, 1H), 6.39 (d, *J* = 1.7 Hz, 1H), 6.83 (d, *J* = 9.6 Hz, 1H), 7.43-7.45 (m, 5H), 7.63 (d, *J* = 1.5 Hz, 1H). ^{13}C NMR ($CDCl_3$, 100 MHz) δ 28.1 (3C), 81.7, 107.3, 112.7, 128.8, 128.9 (2C), 129.0, 129.2 (2C), 129.8, 140.9, 144.0, 165.2. Mass Spectrometry: HRMS (ESI-TOF) (m/z): Calcd for $C_{16}H_{19}N_2O_2$ ([M + H]$^+$) 271.1441, found 271.1445.

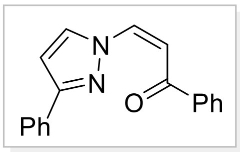

(Z)-1-Phenyl-3-(3-phenyl-1H-pyrazol-1-yl)prop-2-en-1-one (6m). Eluent: petroleum ether (60-90 °C)/ ethyl acetate = 8:1 (v/v). White solid, 41.1 mg, yield 60%. m.p: 100.7 ºC. NMR Spectroscopy: ^{1}H NMR ($CDCl_3$, 400 MHz) δ 6.49 (d, *J* = 1.6 Hz, 1H), 7.42-7.45 (m, 2H), 7.47-7.53 (m, 5H), 7.56-7.59 (m, 1H), 7.76-7.80 (m, 2H), 8.04-8.06 (m, 2H), 8.14 (d, *J* = 13.2 Hz, 1H). ^{13}C NMR ($CDCl_3$, 100 MHz) δ 109.4, 110.6, 126.4, 128.5 (2C), 128.8 (2C), 128.9, 129.2

(2C), 129.7 (2C), 133.1, 137.7, 138.1, 143.1, 146.6, 189.6. Mass Spectrometry: HRMS (ESI-TOF) (m/z): Calcd for $C_{18}H_{15}N_2O$ ($[M + H]^+$) 275.1179, found 275.1181.

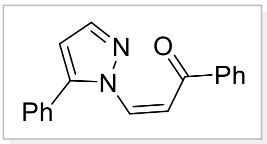

(Z)-1-phenyl-3-(5-phenyl-1H-pyrazol-1-yl)prop-2-en-1-one (6m'). Eluent: petroleum ether (60-90 °C)/ ethyl acetate = 8:1 (v/v). White solid, 8.2 mg, yield 12%. m.p: 123.4 ºC. NMR Spectroscopy: ^{1}H NMR ($CDCl_3$, 400 MHz) δ 6.79 (d, J = 2.5 Hz, 1H), 7.40 (d, J = 7.2 Hz, 1H), 7.45 (t, J = 6.9 Hz, 2H), 7.51 (t, J = 7.8 Hz, 2H), 7.59 (t, J = 7.2 Hz, 1H), 7.67 (d, J = 13.4 Hz, 1H), 7.71 (d, J = 2.6 Hz, 1H), 7.91-7.93 (m, 2H), 8.06-8.12 (m, 3H). ^{13}C NMR ($CDCl_3$, 100 MHz) δ 107.1, 109.4, 125.9, 126.4 (2C), 128.5 (2C), 128.8 (2C), 128.9 (2C), 129.2, 132.1, 133.1, 138.1, 139.6, 155.6, 189.7. Mass Spectrometry: HRMS (ESI-TOF) (m/z): Calcd for $C_{18}H_{15}N_2O$ ($[M + H]^+$) 275.1179, found 275.1181.

IV. General procedures for a gram scale synthesis of 3a and 4a

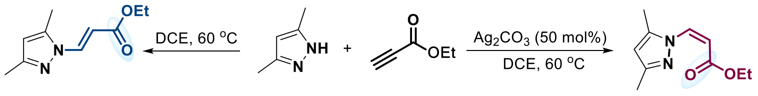

General procedure for a gram scale synthesis of 3a: To a solution of 3,5-dimethyl-1H-pyrazole (**1aa**) (0.96 g, 10.0 mmol) and ethyl propionate (**2a**) (1.18 mL, 12.0 mmol) in DCE (10 ml) at 60 ºC. After the reaction was finished as indicated by TLC (reaction time, 8 h), the resulting mixture was poured into water (20 mL) and extracted with DCM (CH_2Cl_2, 20 mL×3). The combined organic layer was dried over anhydrous Na_2SO_4 and concentrated *in vacuo*. Purification of the crude product with flash column chromatography (petroleum ether /EtOAc = 10:1) to give **3a** (1.571 g, 81%).

General procedure for a gram scale synthesis of 4a: To a solution of 3,5-dimethyl-1*H*-pyrazole (**1aa**) (0.96 g, 10.0 mmol) and ethyl propionate (**2a**) (1.18 mL, 12.0 mmol) in DCE (10 ml) at 60 ºC was added Ag_2CO_3 (1.38 g, 5.0 mmol). After the reaction was finished as indicated by TLC (reaction time, 12 h), the resulting mixture was poured into water (20 mL) and extracted with DCM (CH_2Cl_2, 20 mL×3). The combined organic layer was dried over anhydrous Na_2SO_4 and concentrated *in vacuo*. Purification of the crude product with flash column chromatography (petroleum ether /EtOAc = 8:1) to give **4a** (1.455 mg, 75%).

V. Copies of ^{1}H NMR and ^{13}C NMR spectra of compounds 3

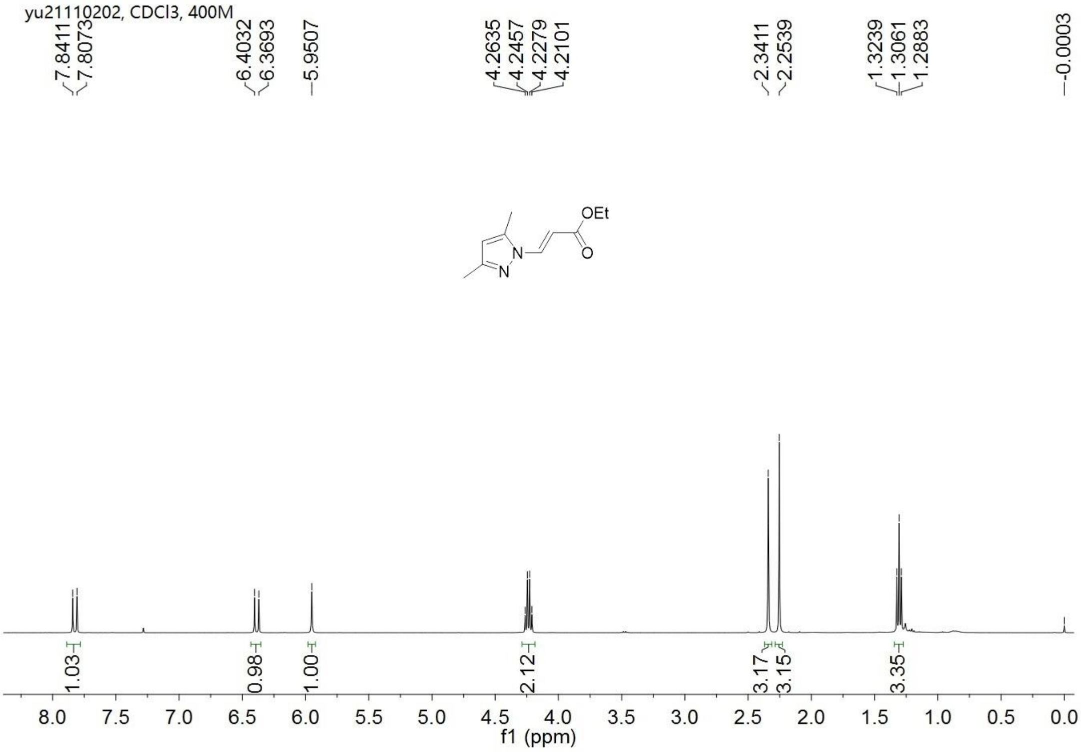

^{1}H NMR of **3a**

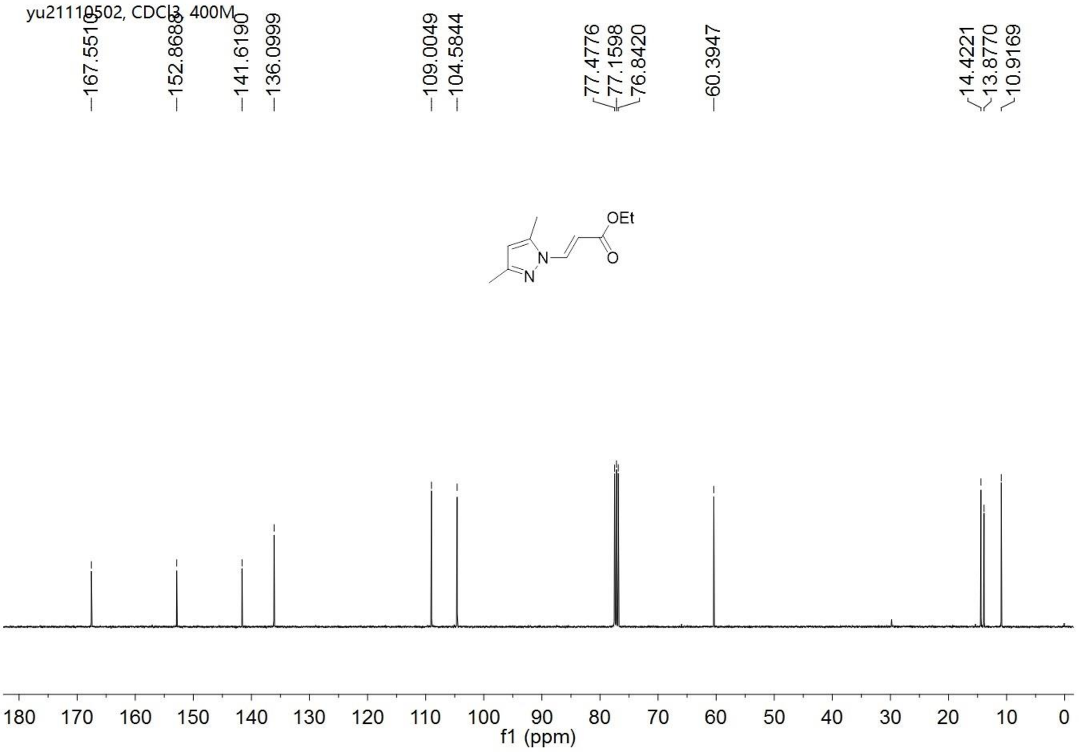

^{13}C NMR of **3a**

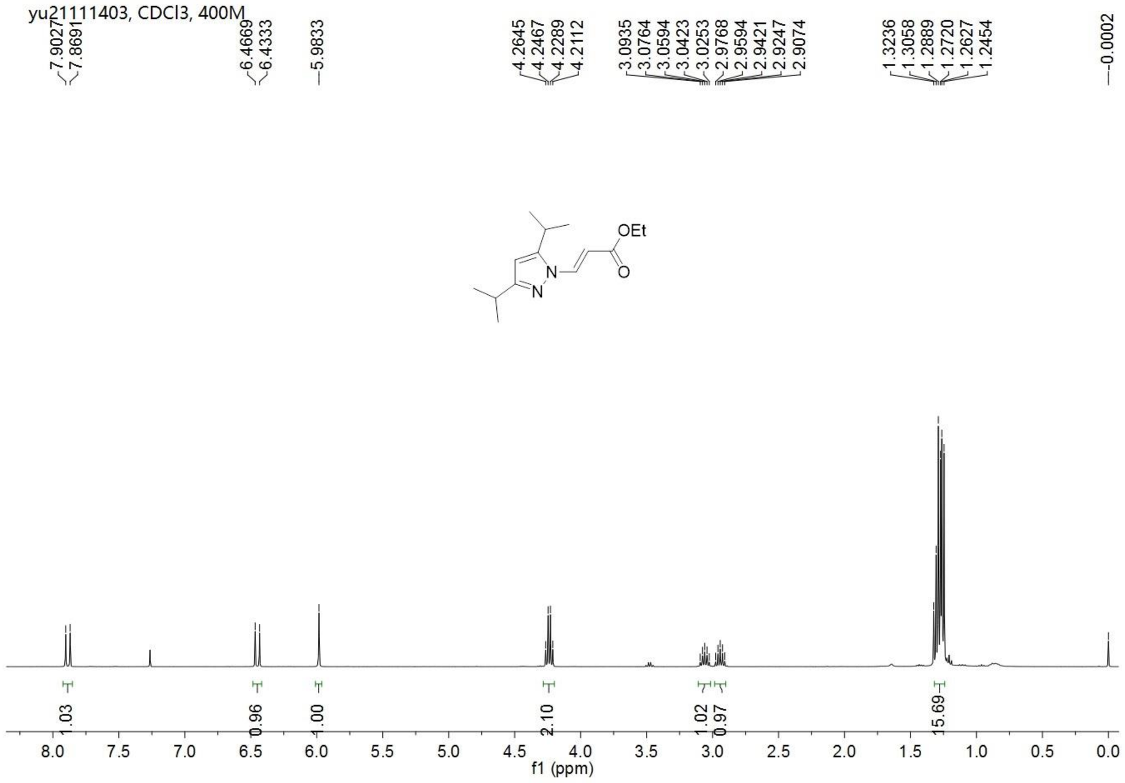

^{1}H NMR of **3b**

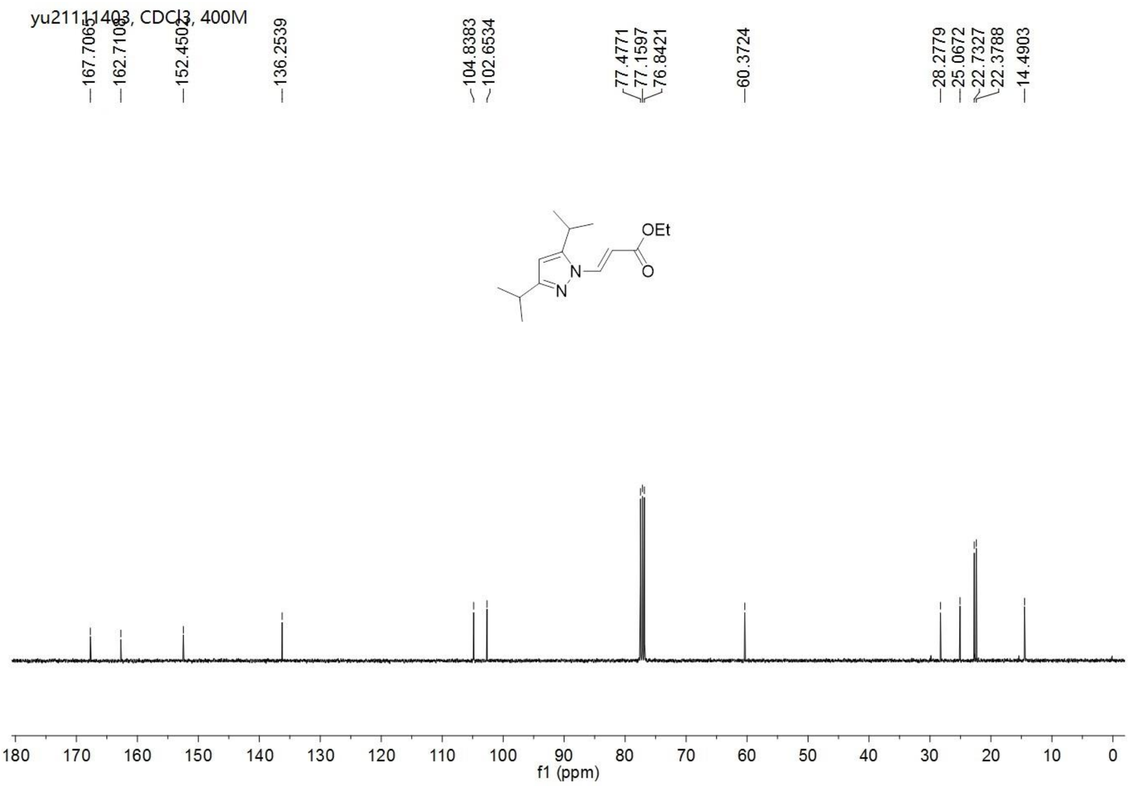

^{13}C NMR of **3b**

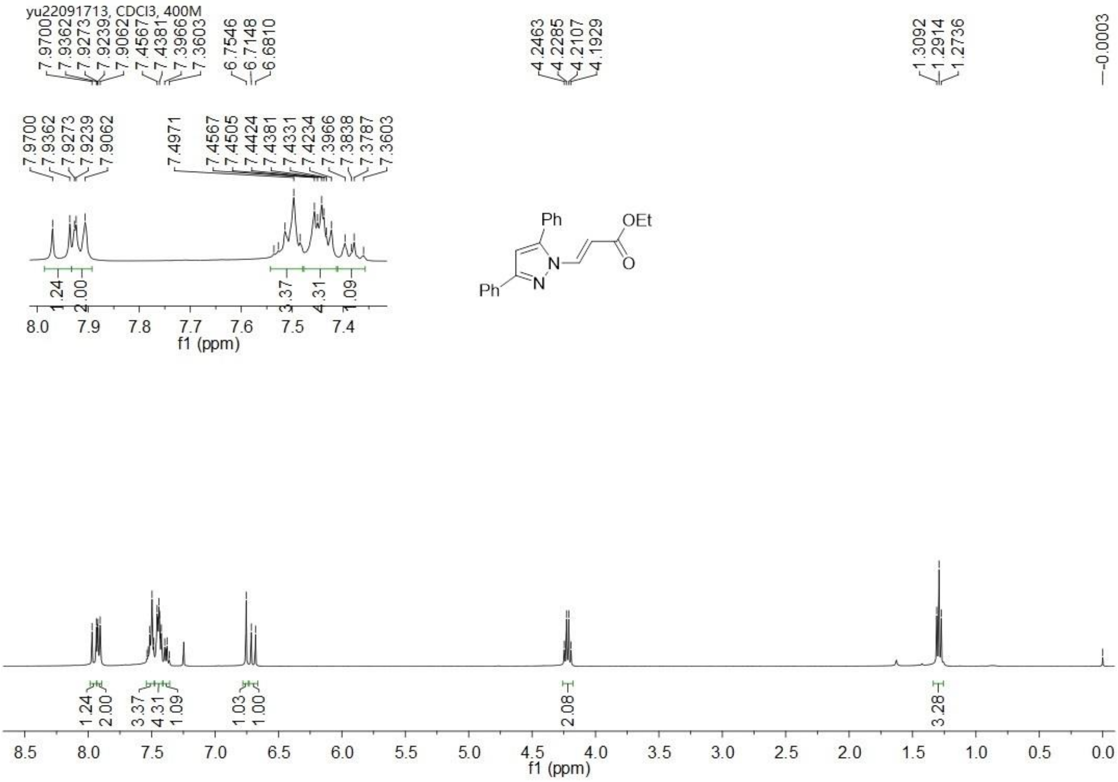

^{1}H NMR of **3c**

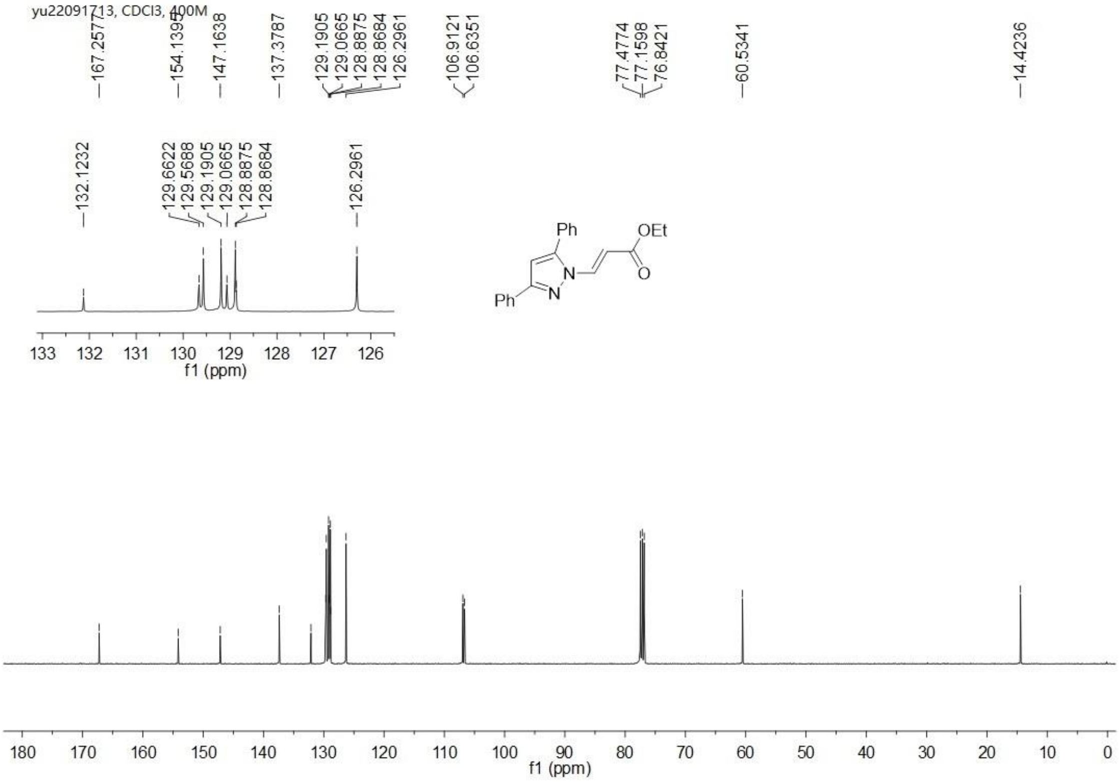

^{13}C NMR of **3c**

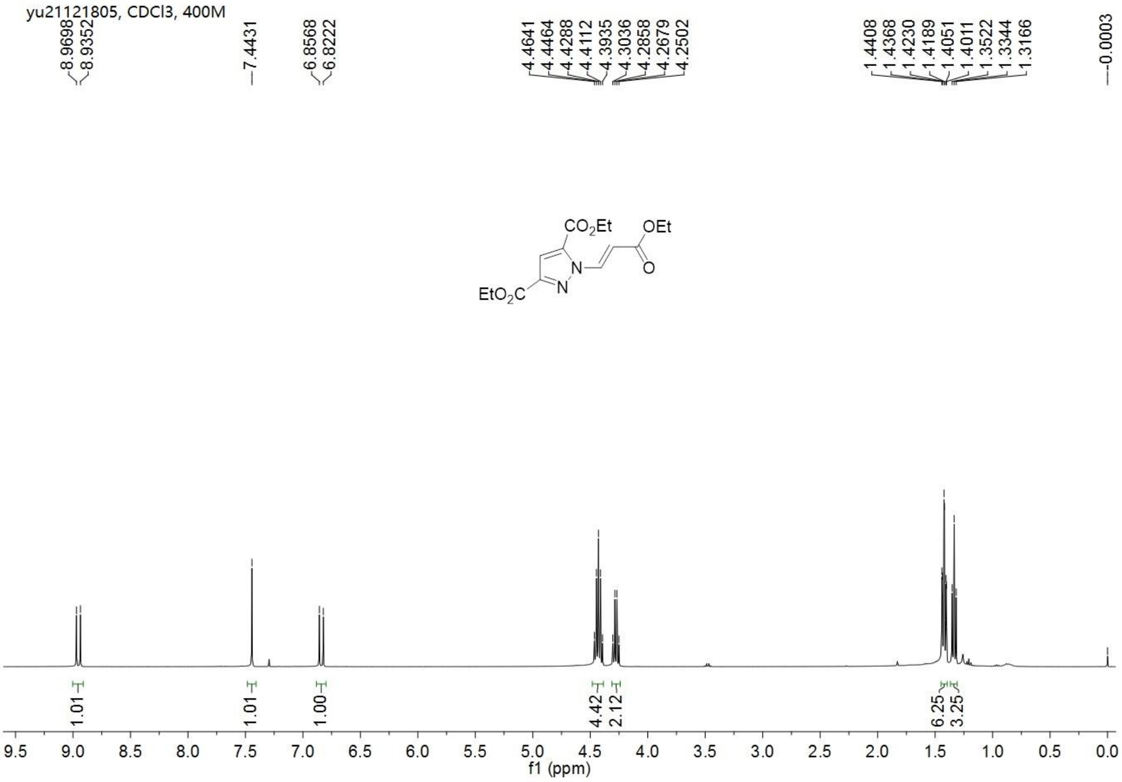

^{1}H NMR of **3d**

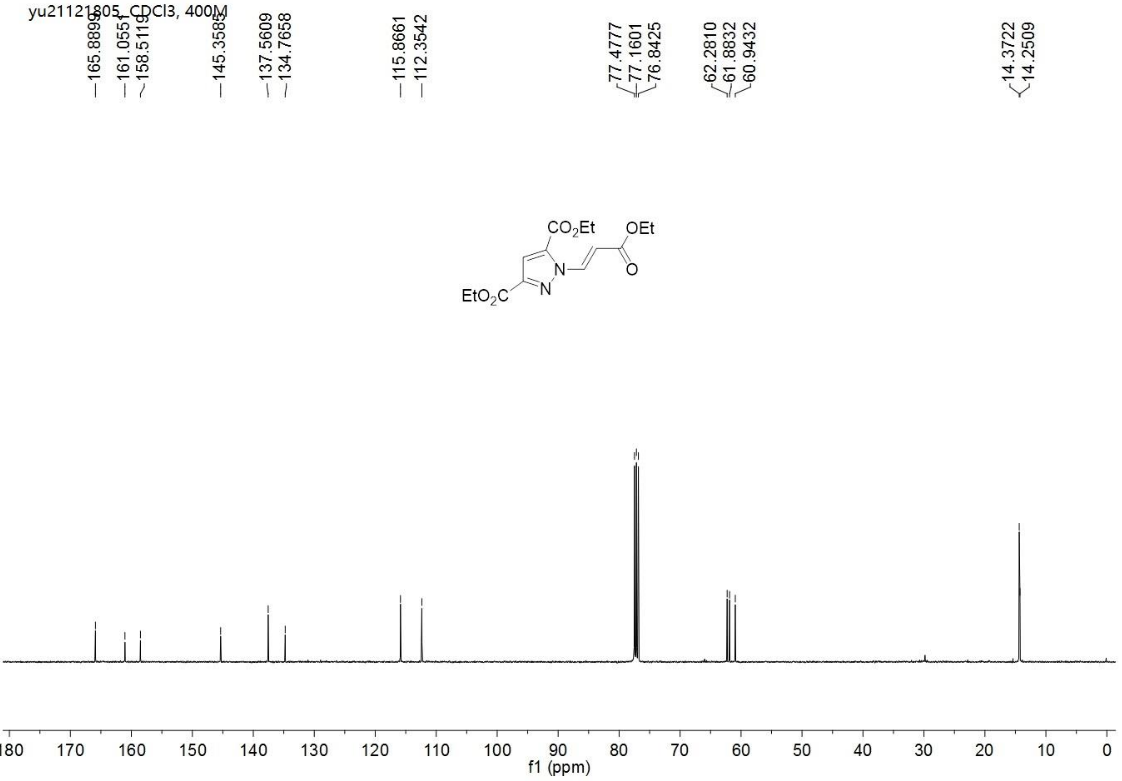

^{13}C NMR of **3d**

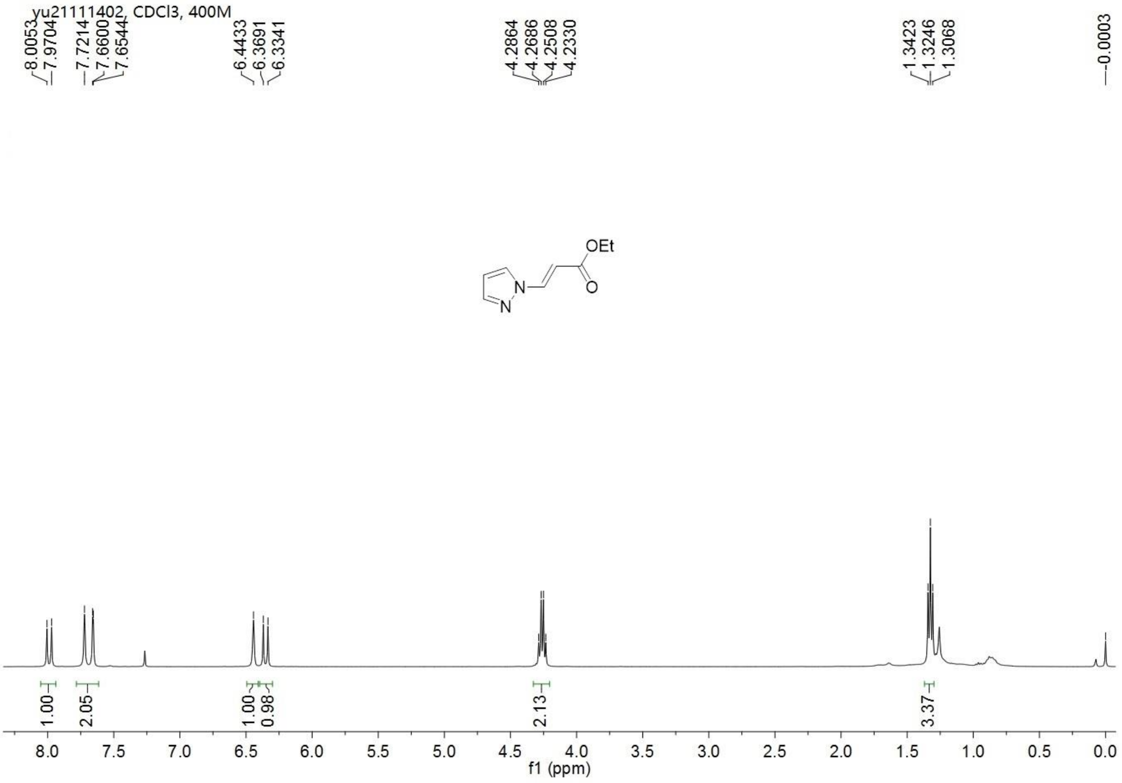

^{1}H NMR of **3e**

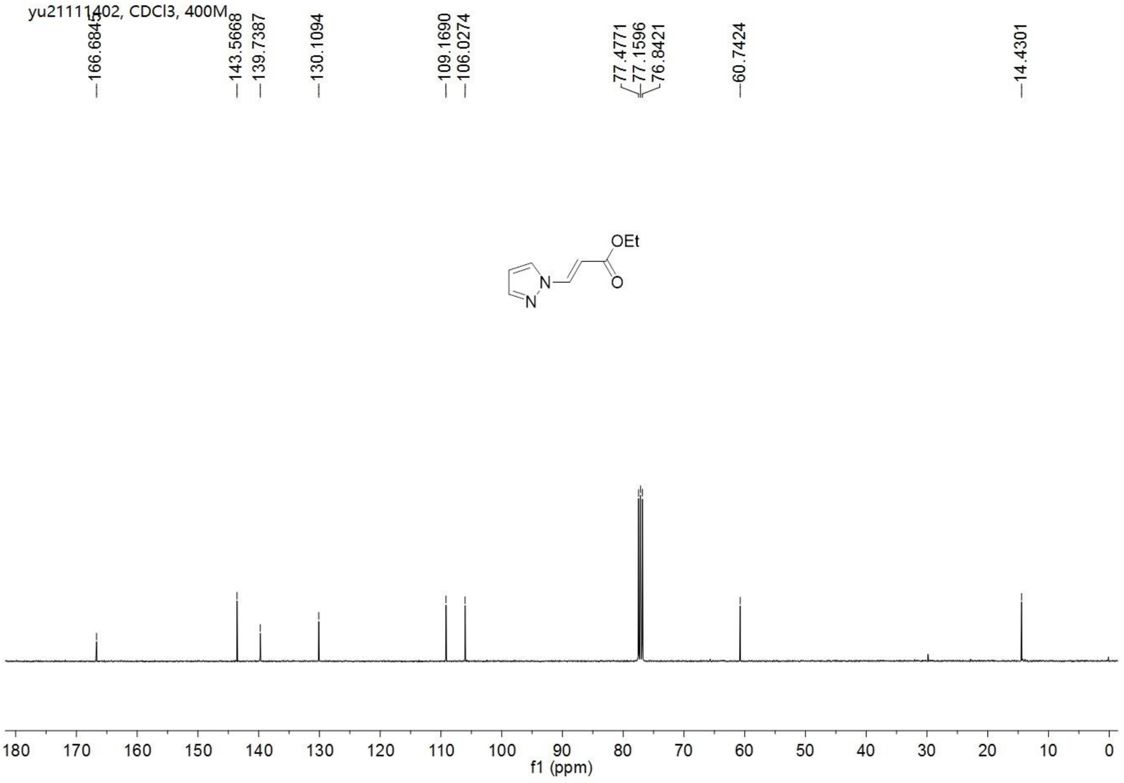

^{13}C NMR of **3e**

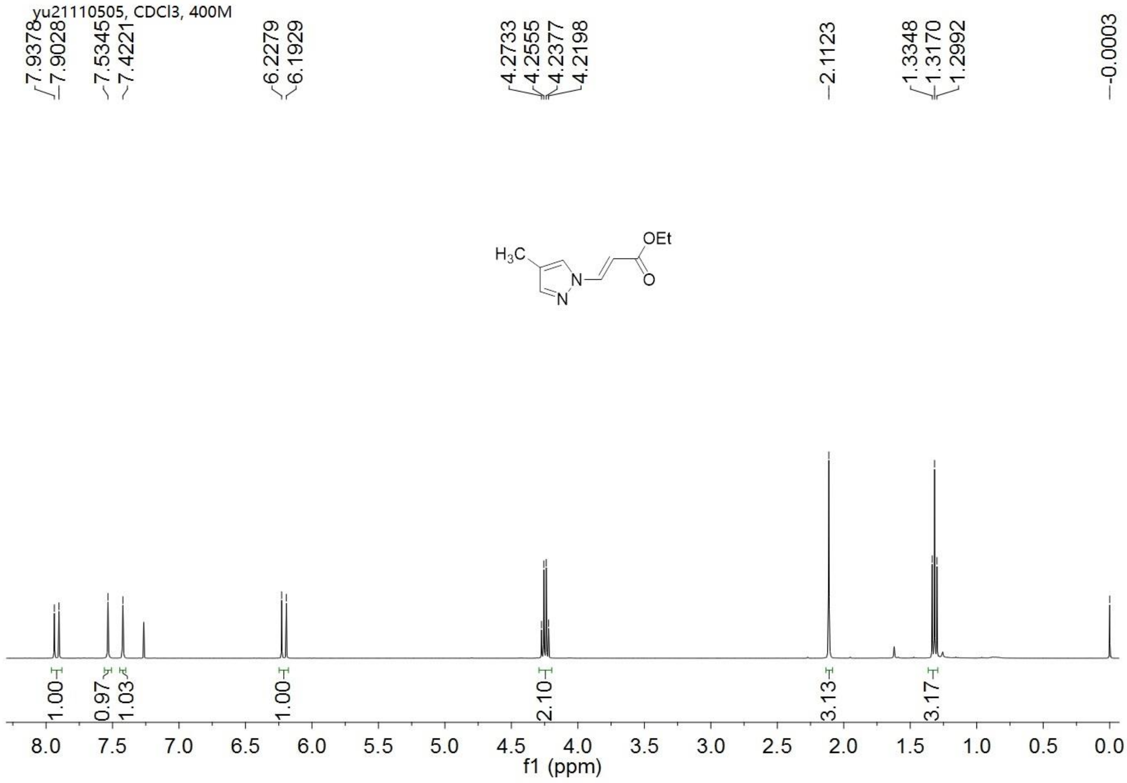

^{1}H NMR of **3f**

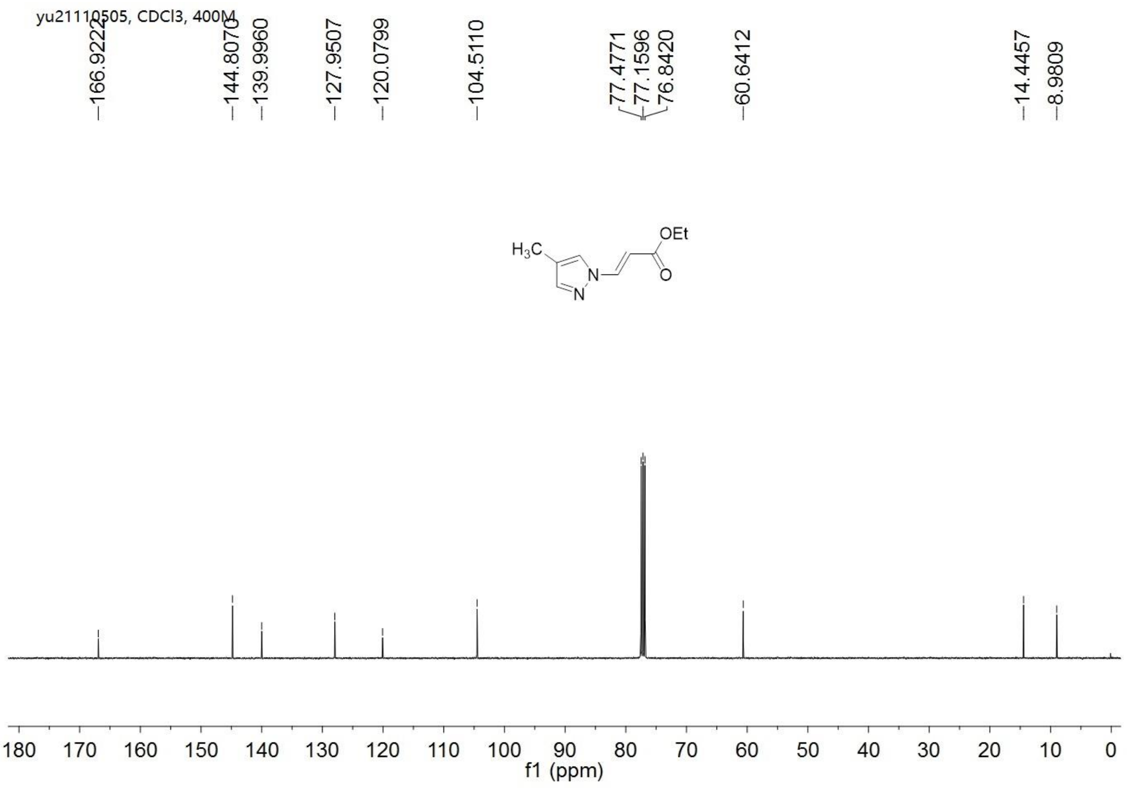

^{13}C NMR of **3f**

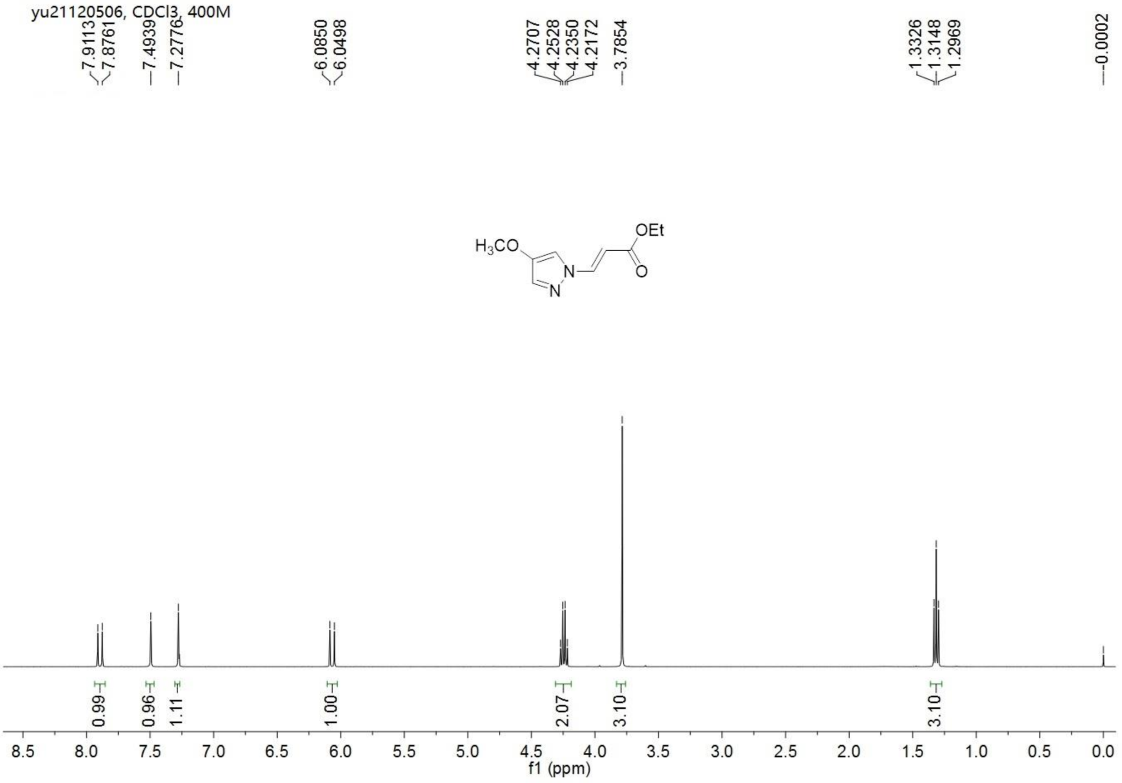

^{1}H NMR of **3g**

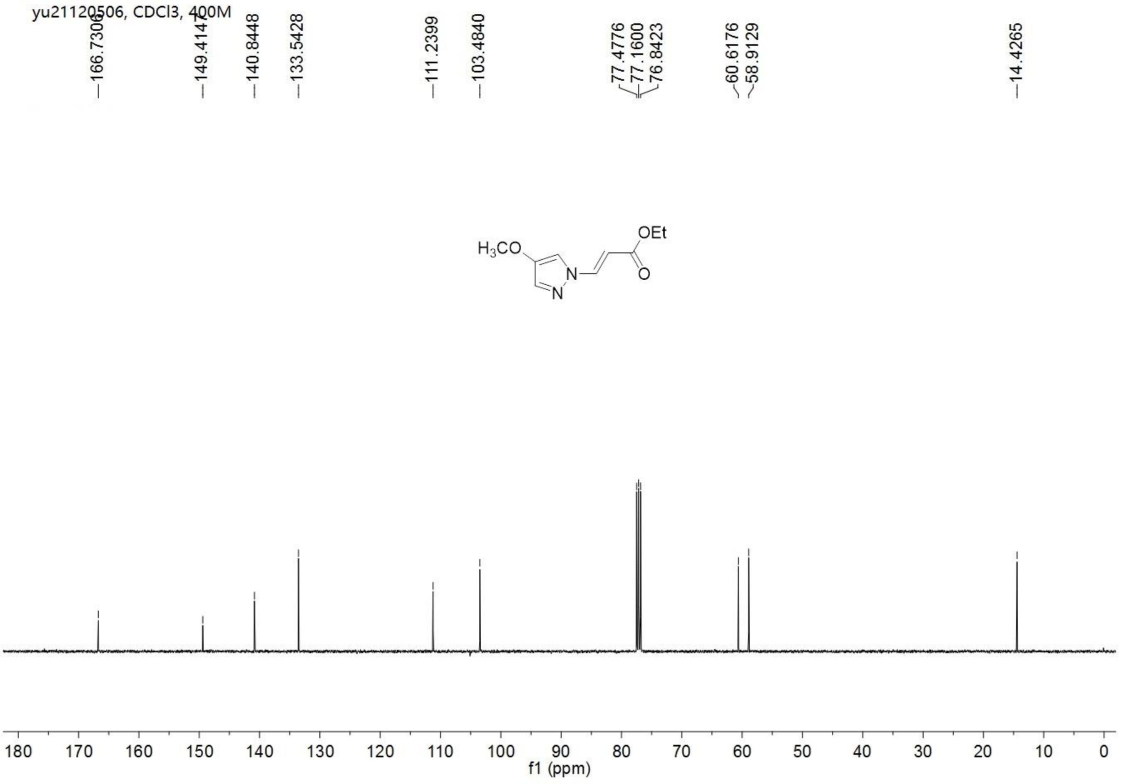

^{13}C NMR of **3g**

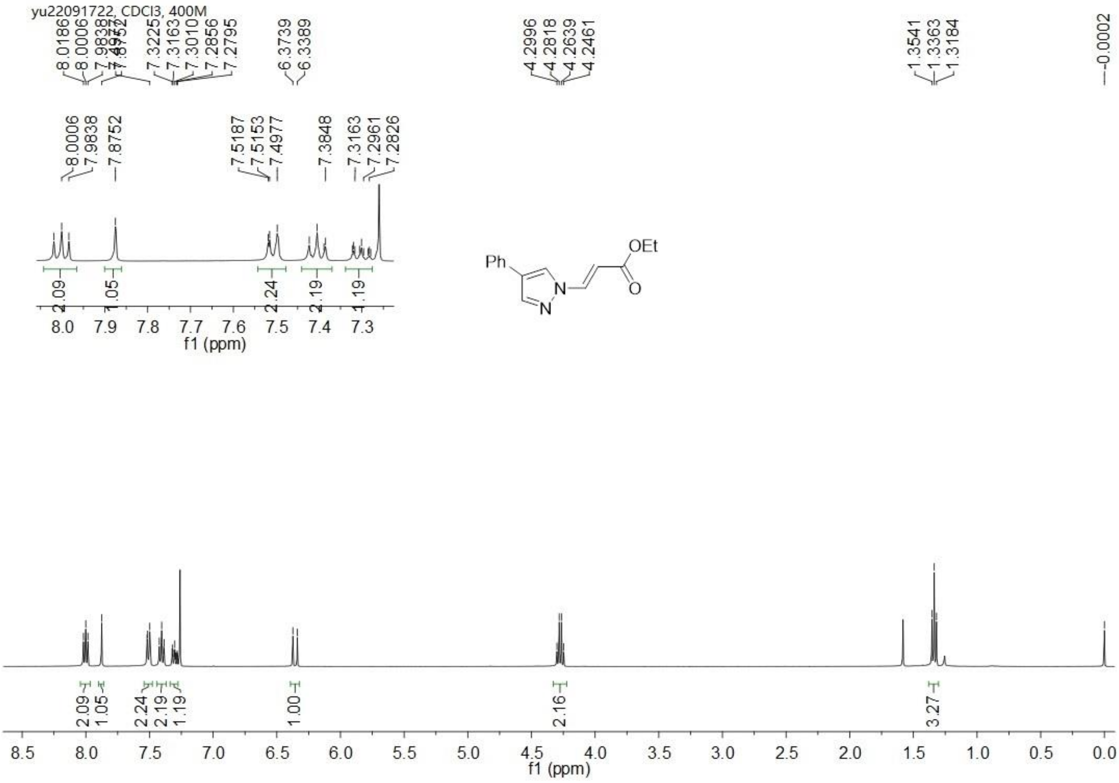

^{1}H NMR of **3h**

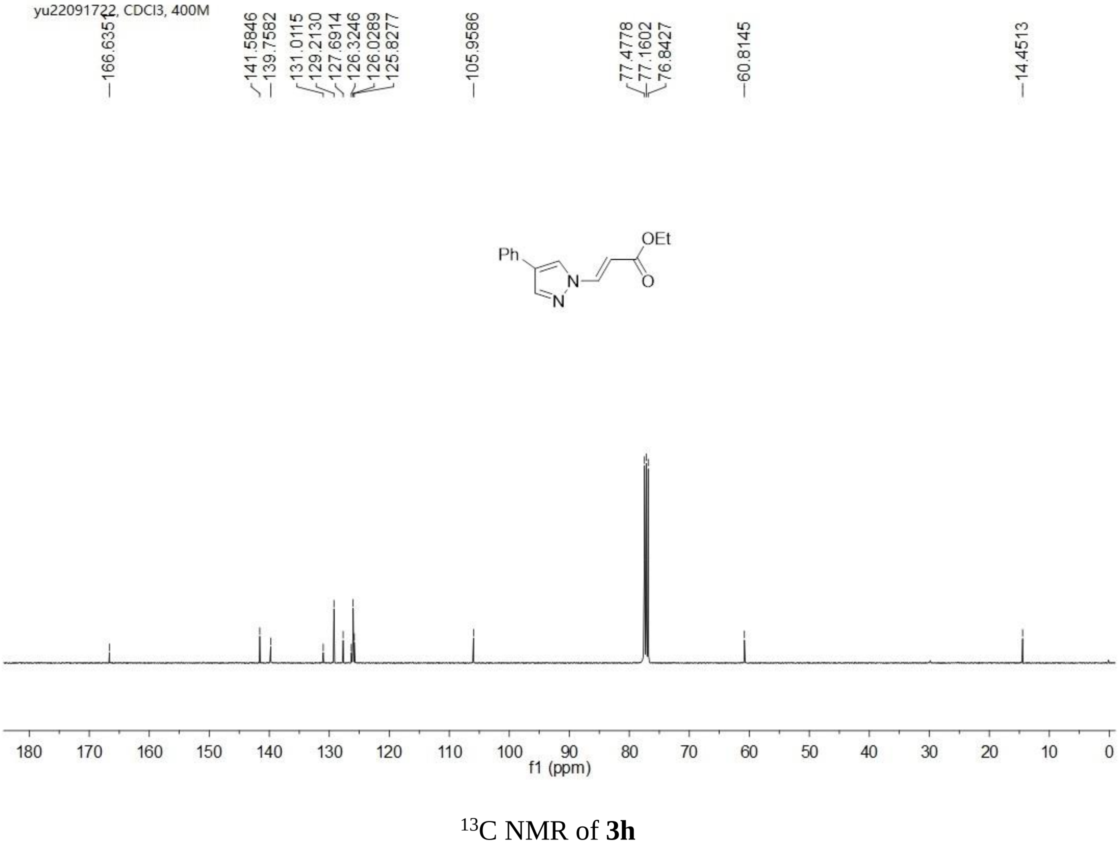

^{13}C NMR of **3h**

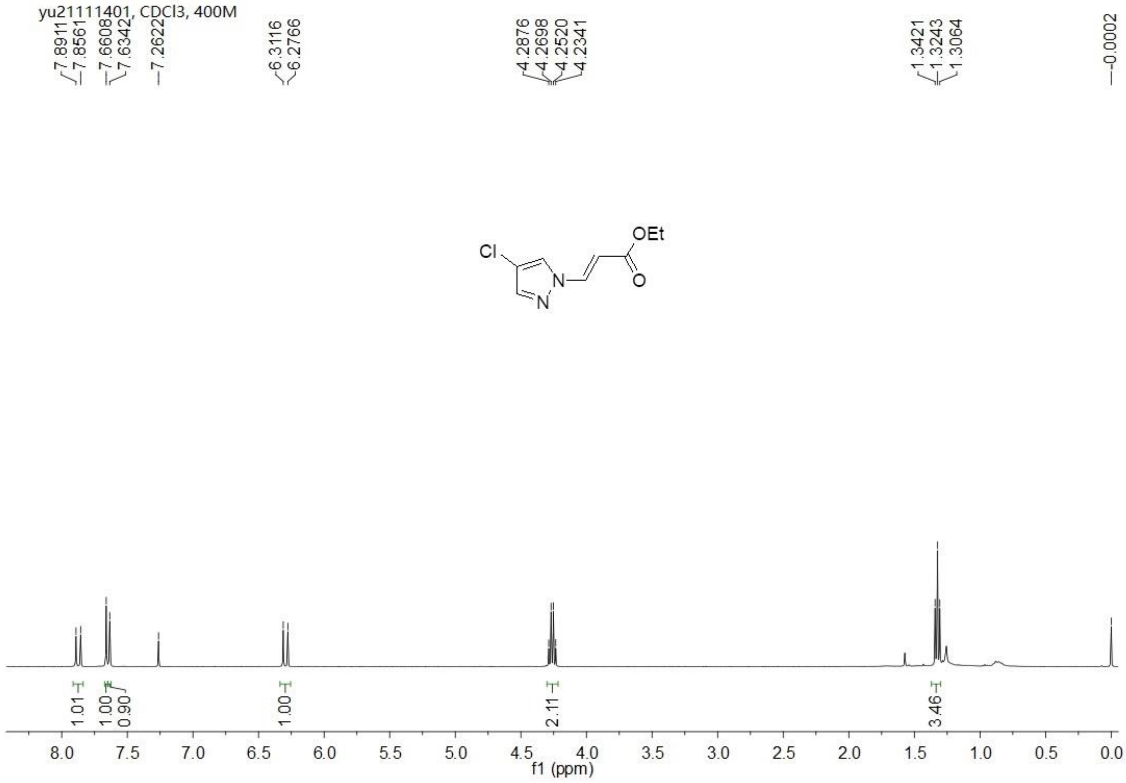

^{1}H NMR of **3i**

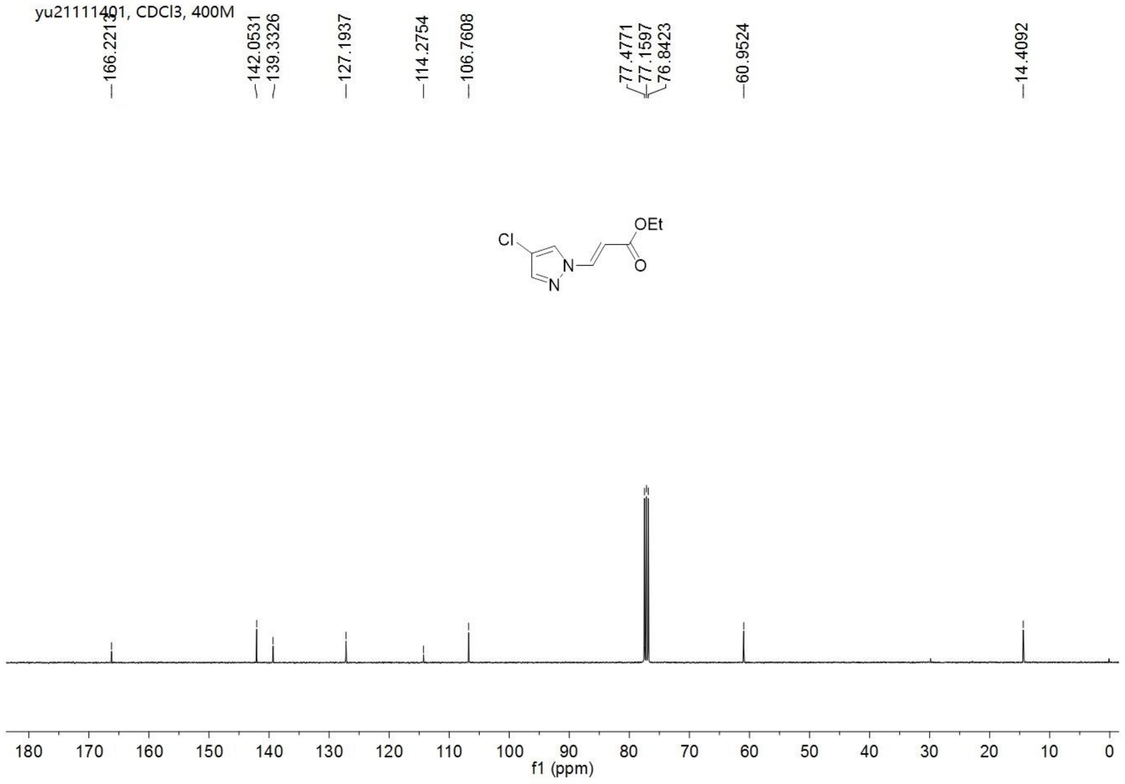

^{13}C NMR of **3i**

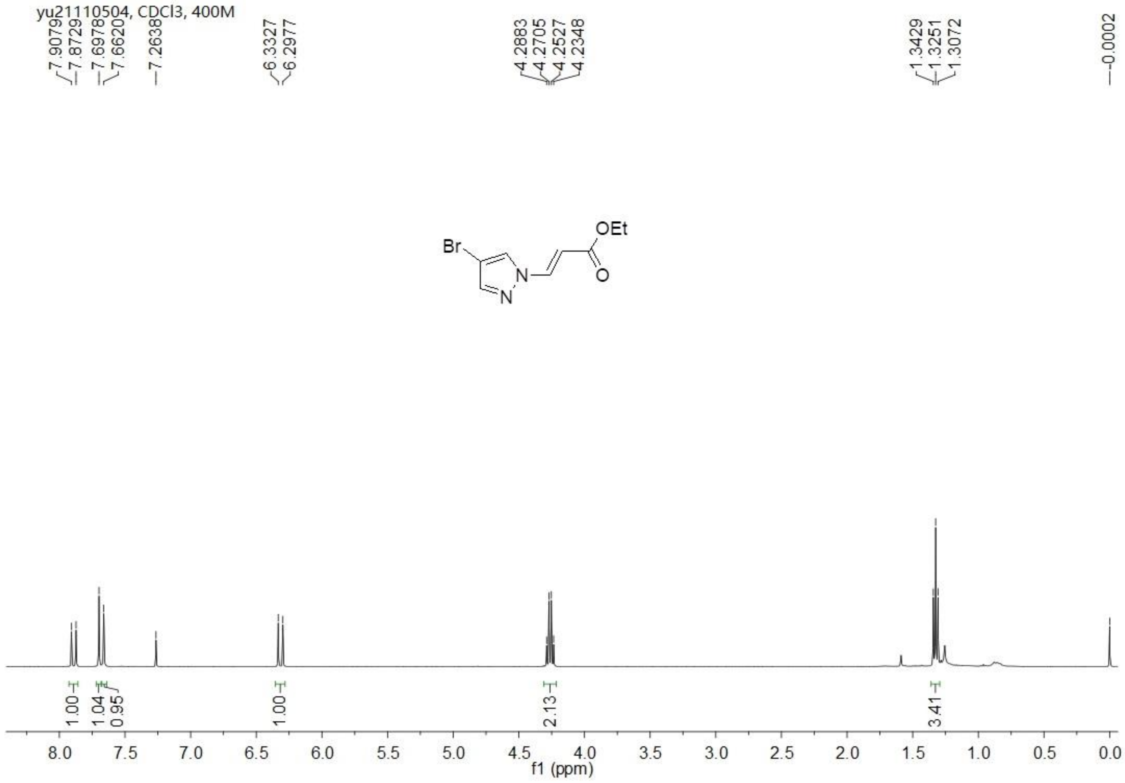

^{1}H NMR of **3j**

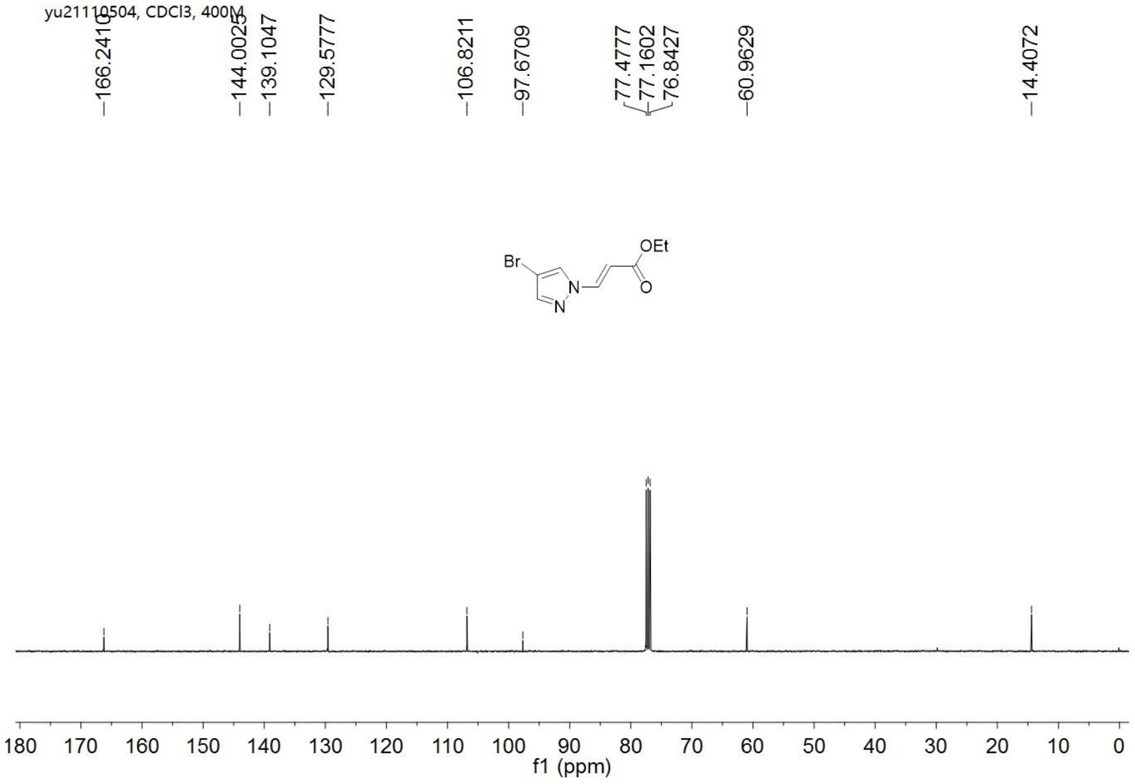

^{13}C NMR of **3j**

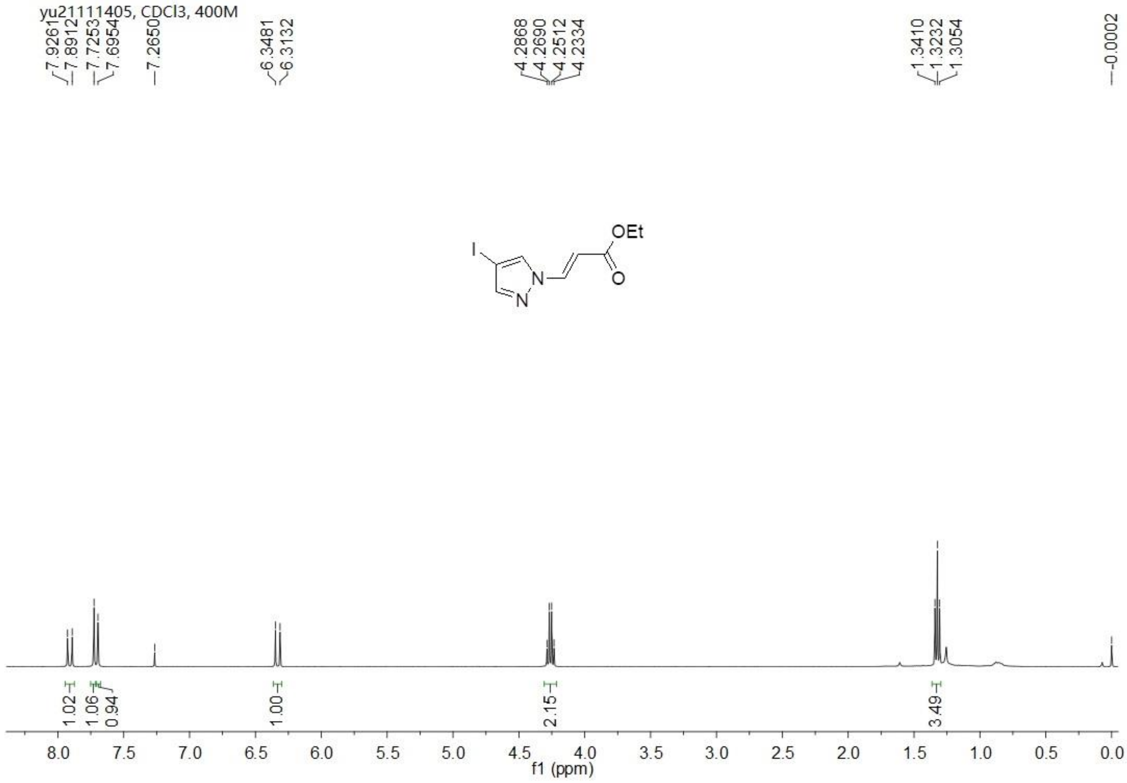

^{1}H NMR of **3k**

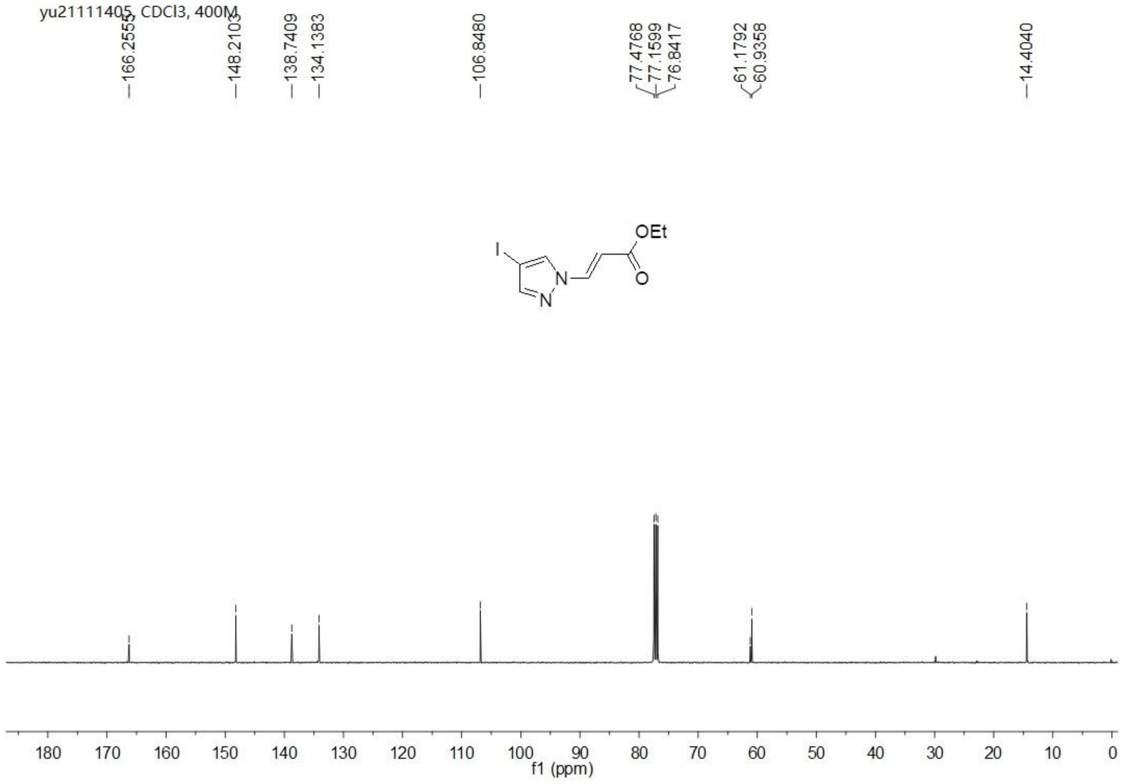

^{13}C NMR of **3k**

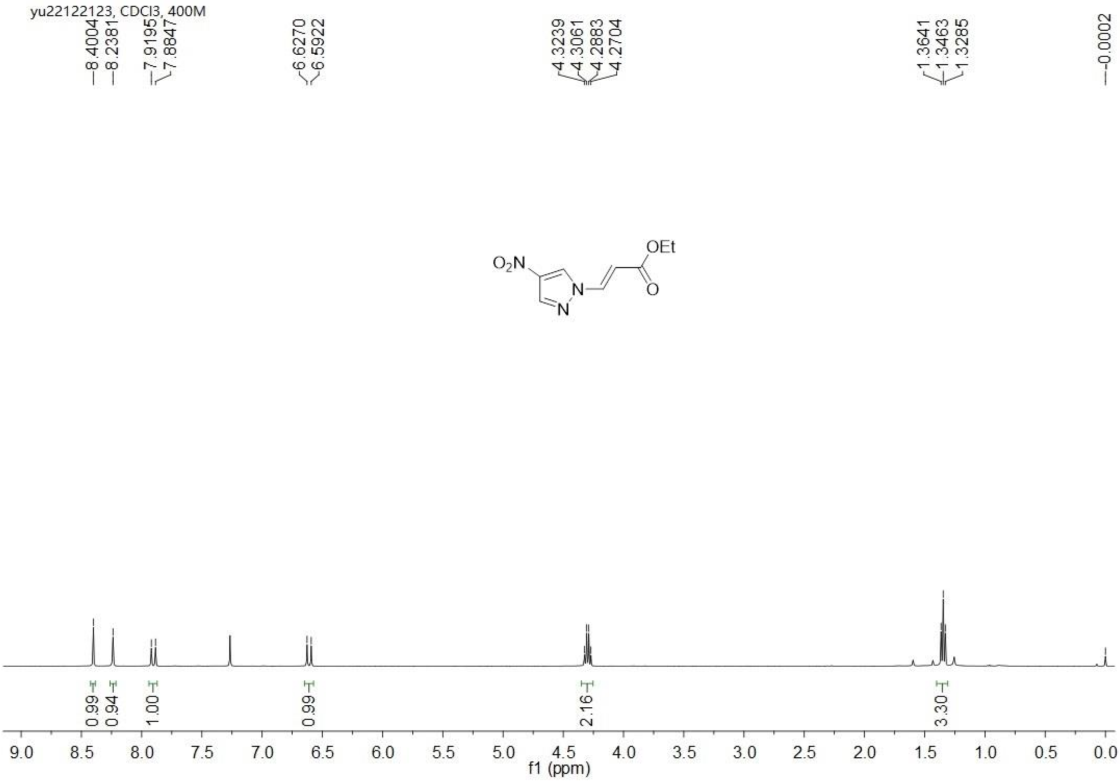

^{1}H NMR of **3l**

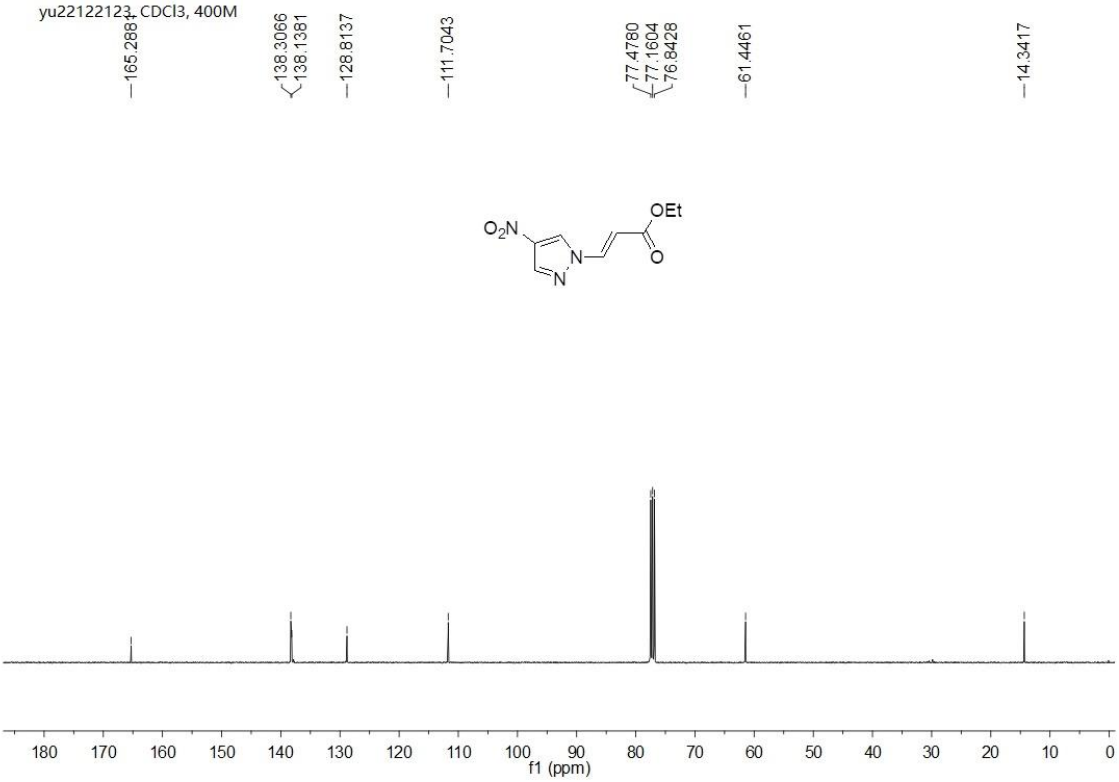

^{13}C NMR of **3l**

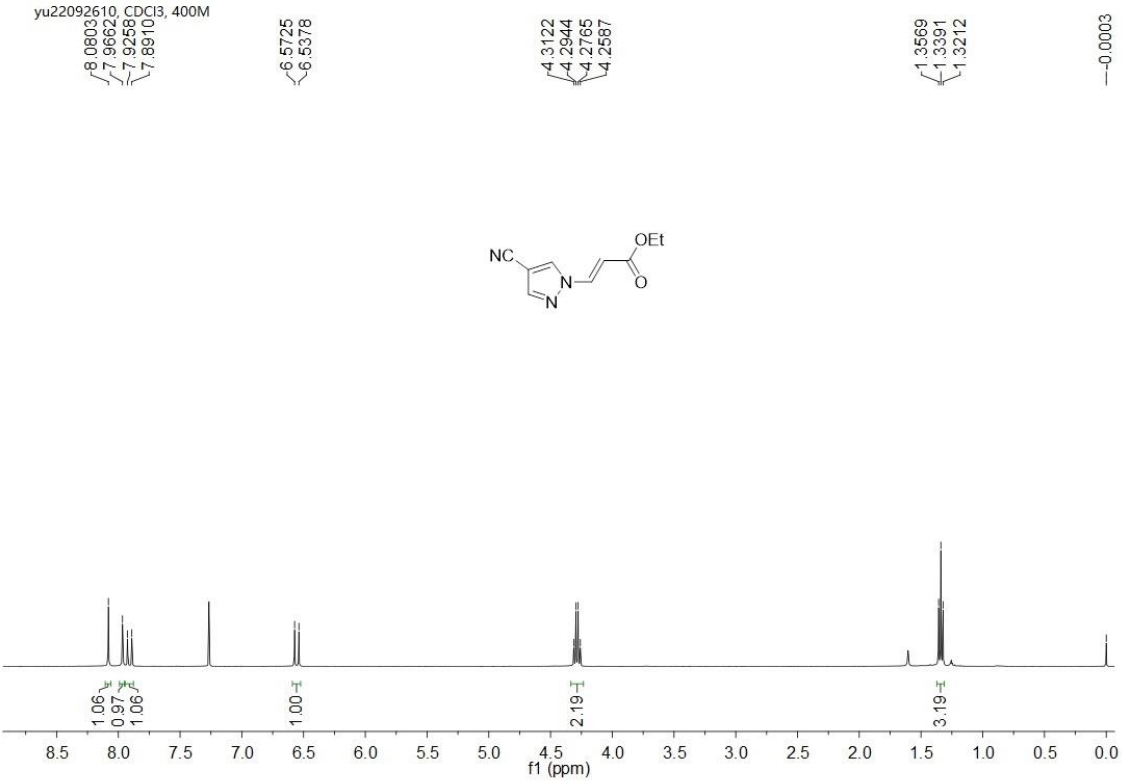

^{1}H NMR of **3m**

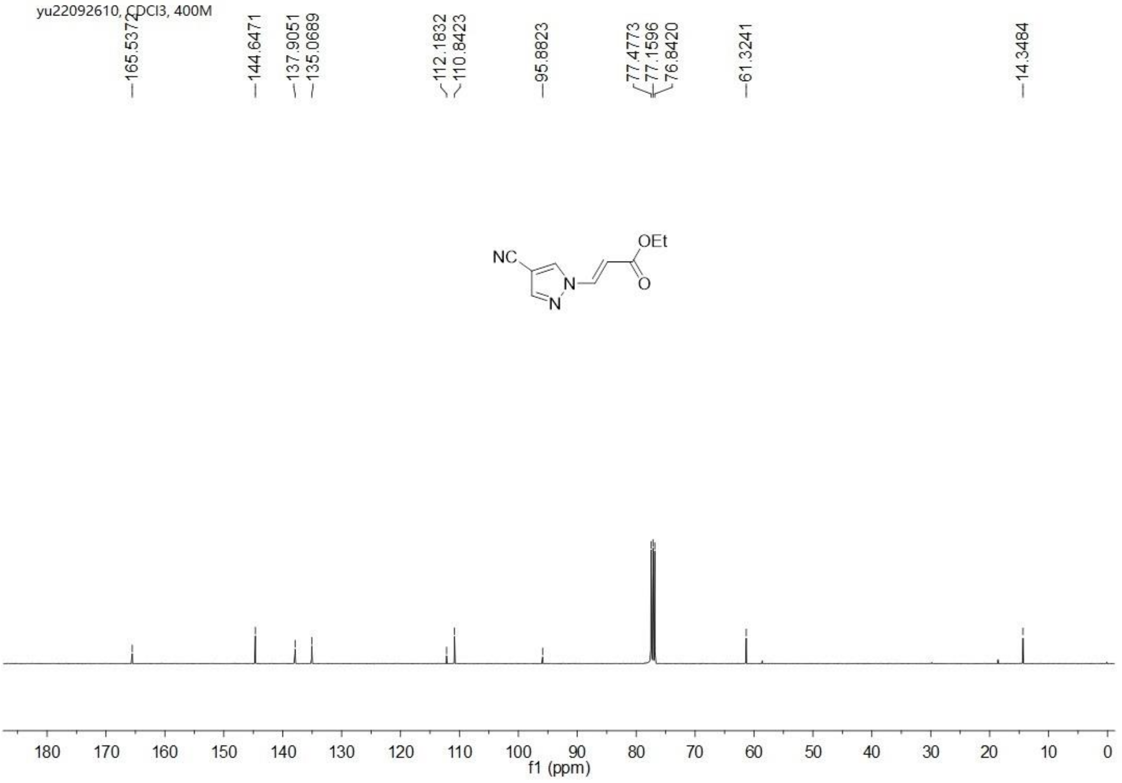

^{13}C NMR of **3m**

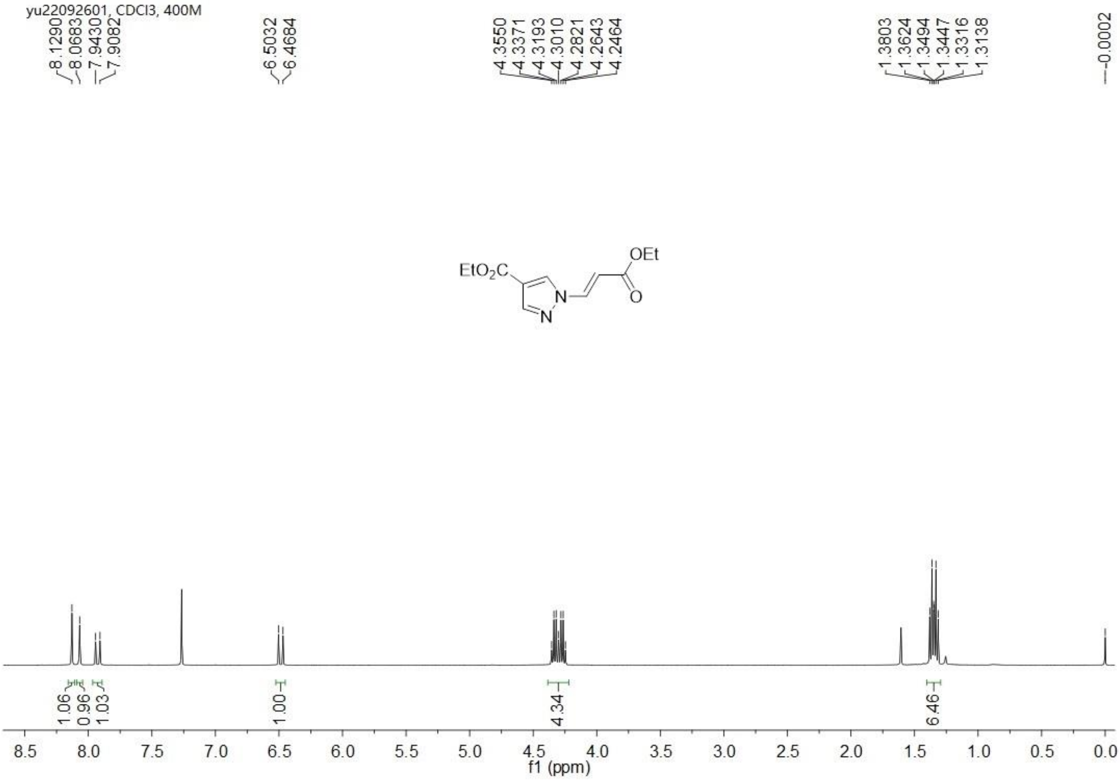

^{1}H NMR of **3n**

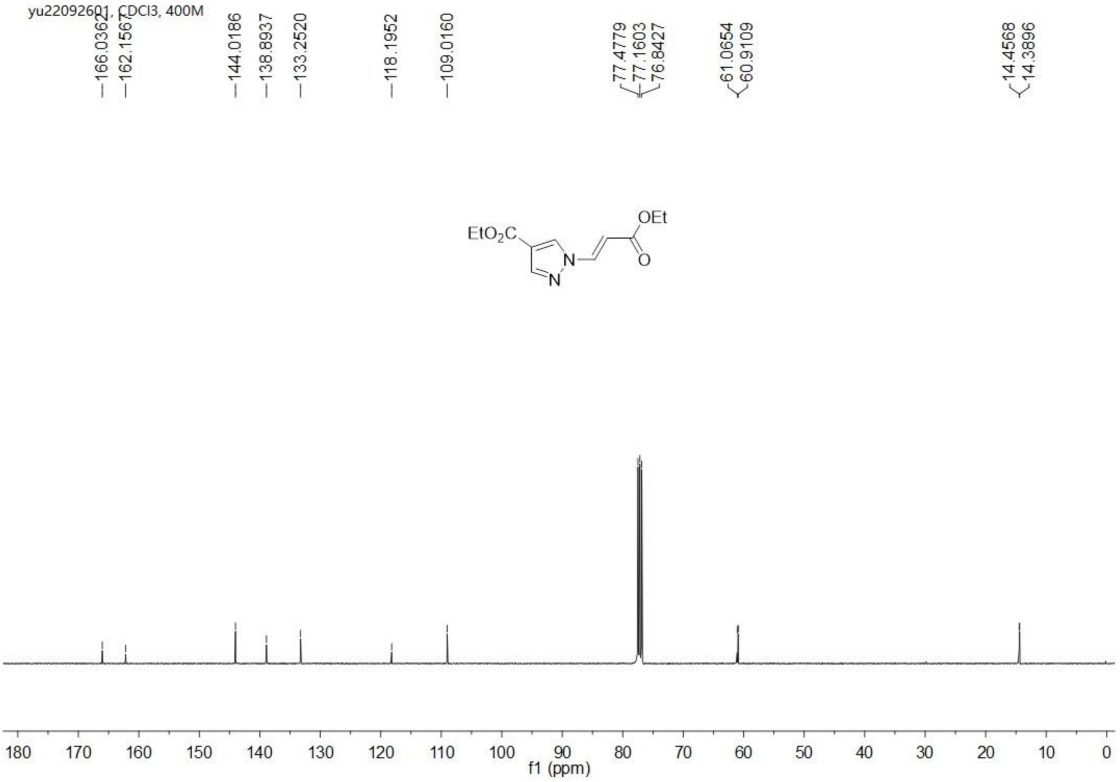

^{13}C NMR of **3n**

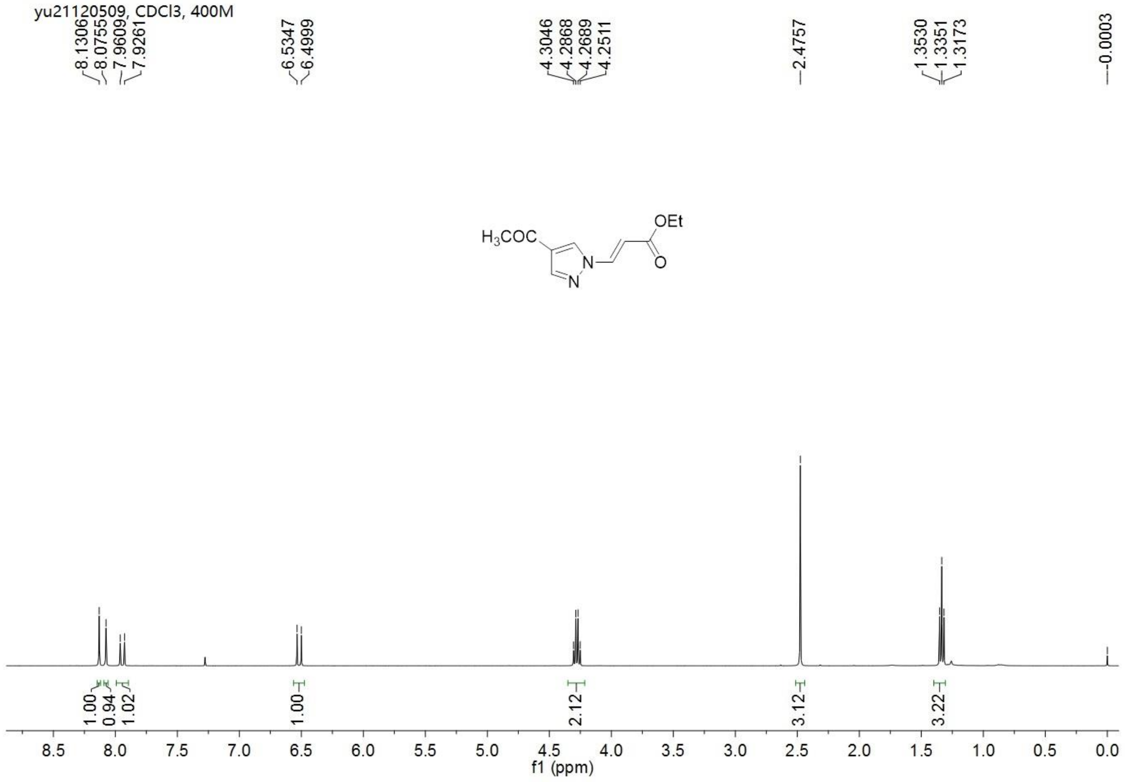

^{1}H NMR of **3o**

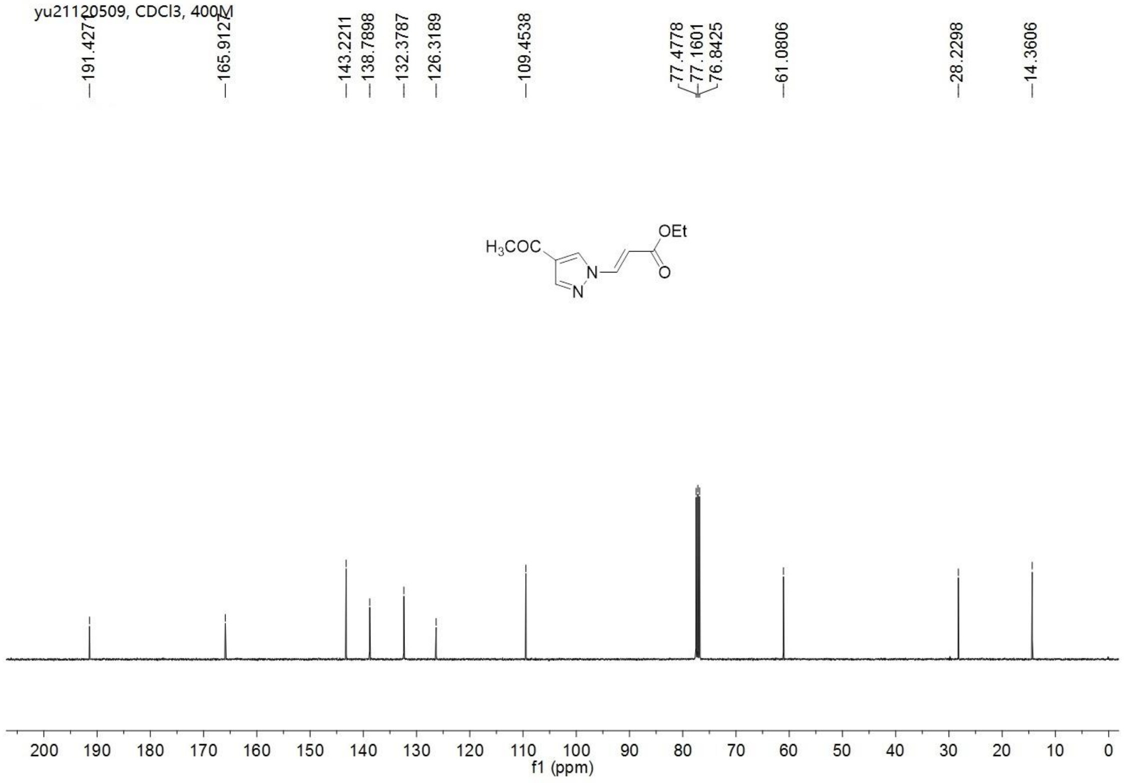

^{13}C NMR of **3o**

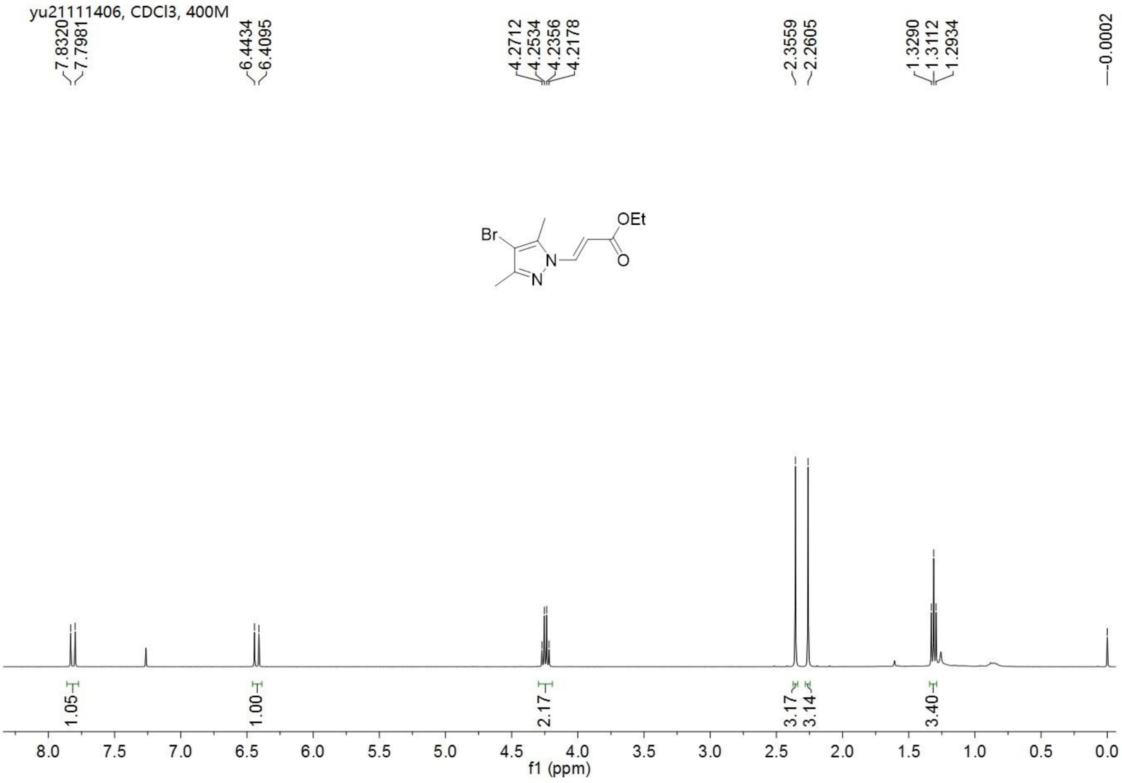

^{1}H NMR of **3p**

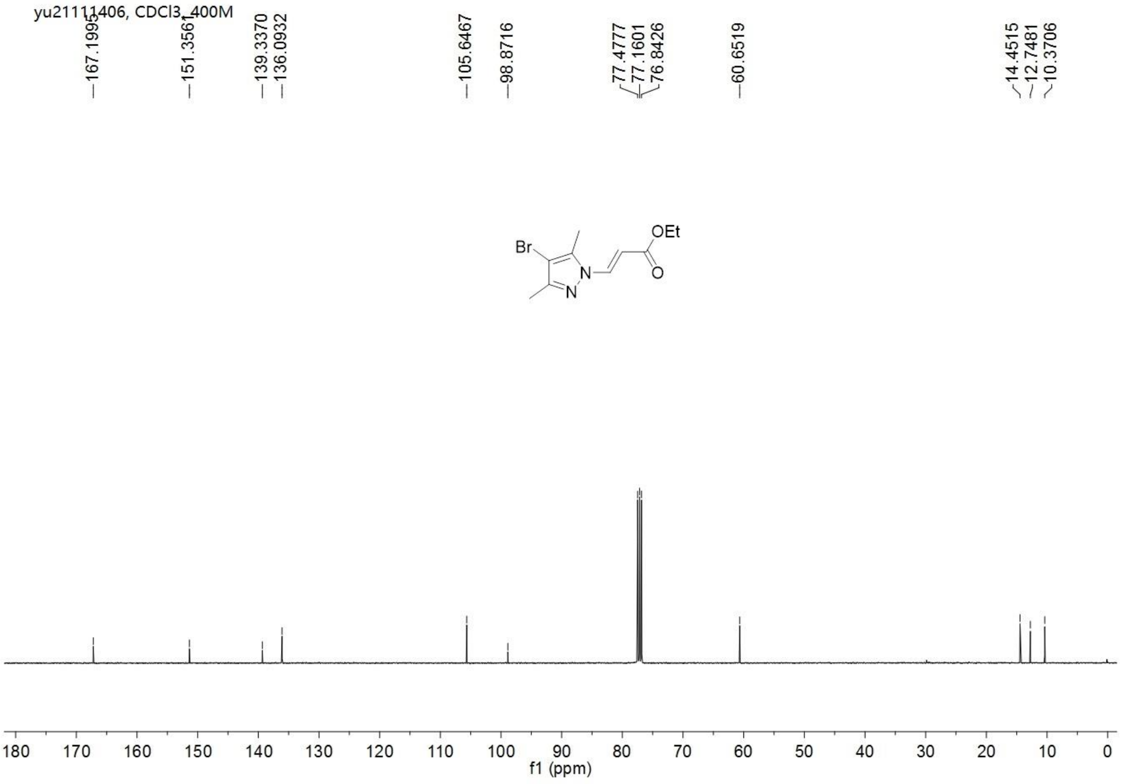

^{13}C NMR of **3p**

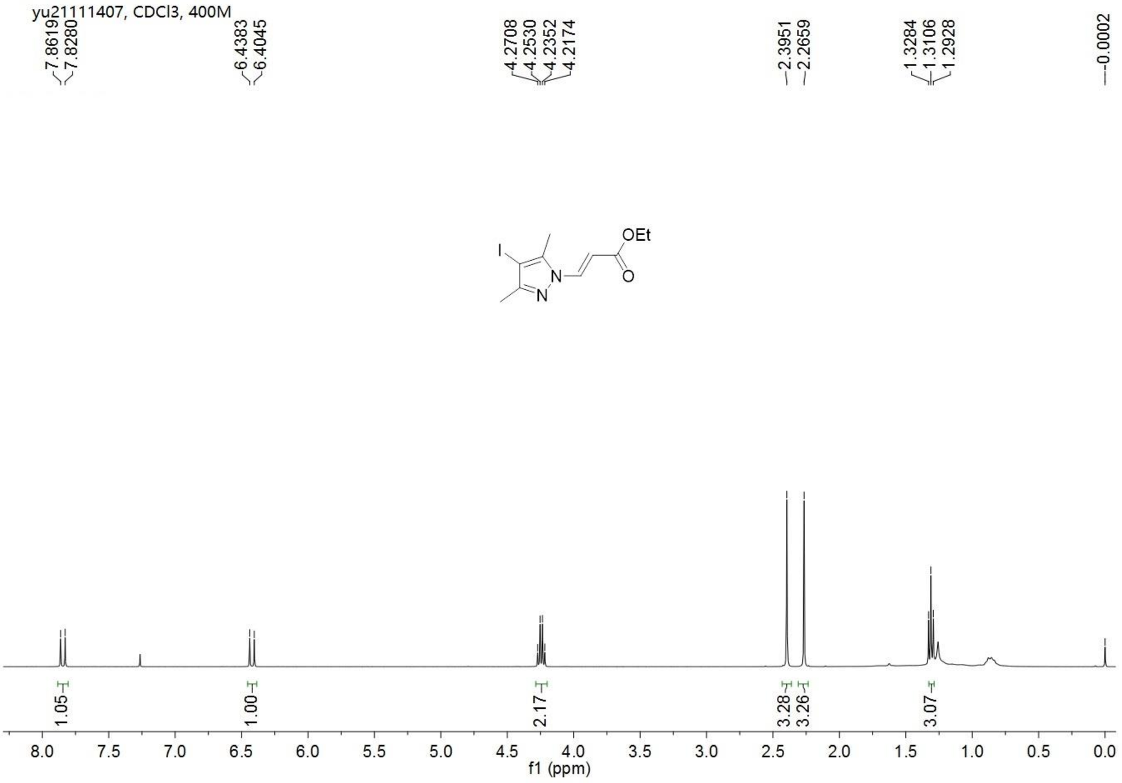

^{1}H NMR of **3q**

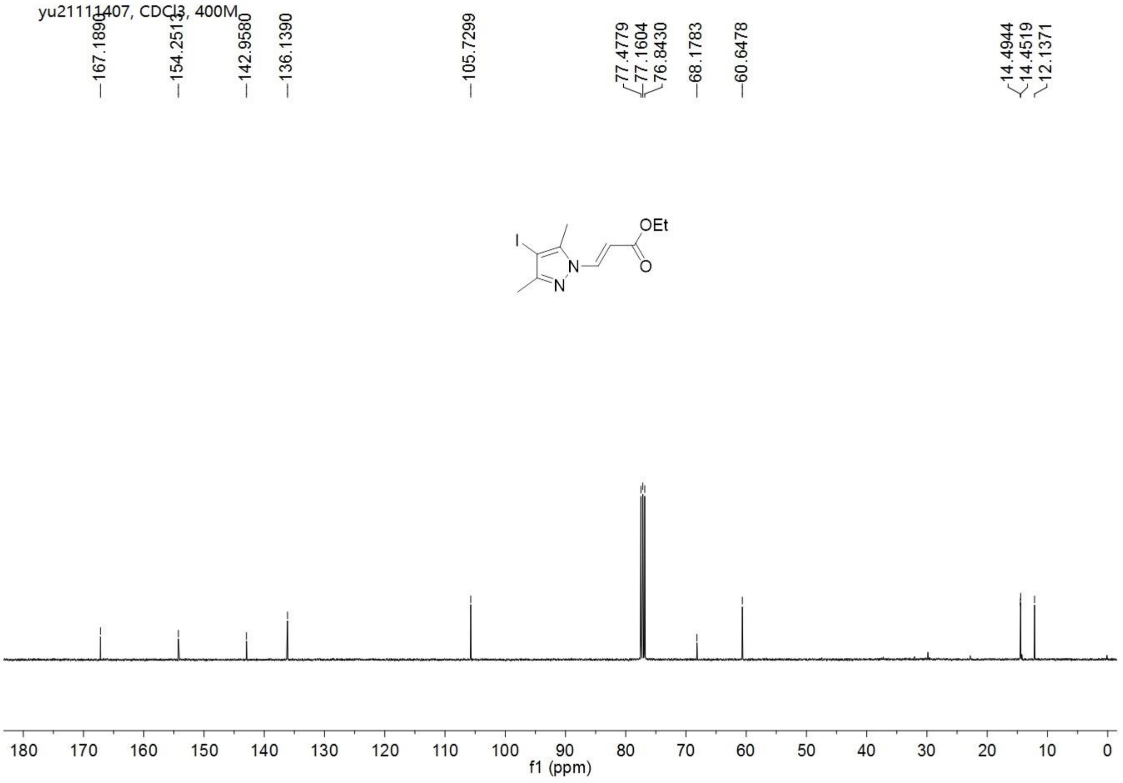

^{13}C NMR of **3q**

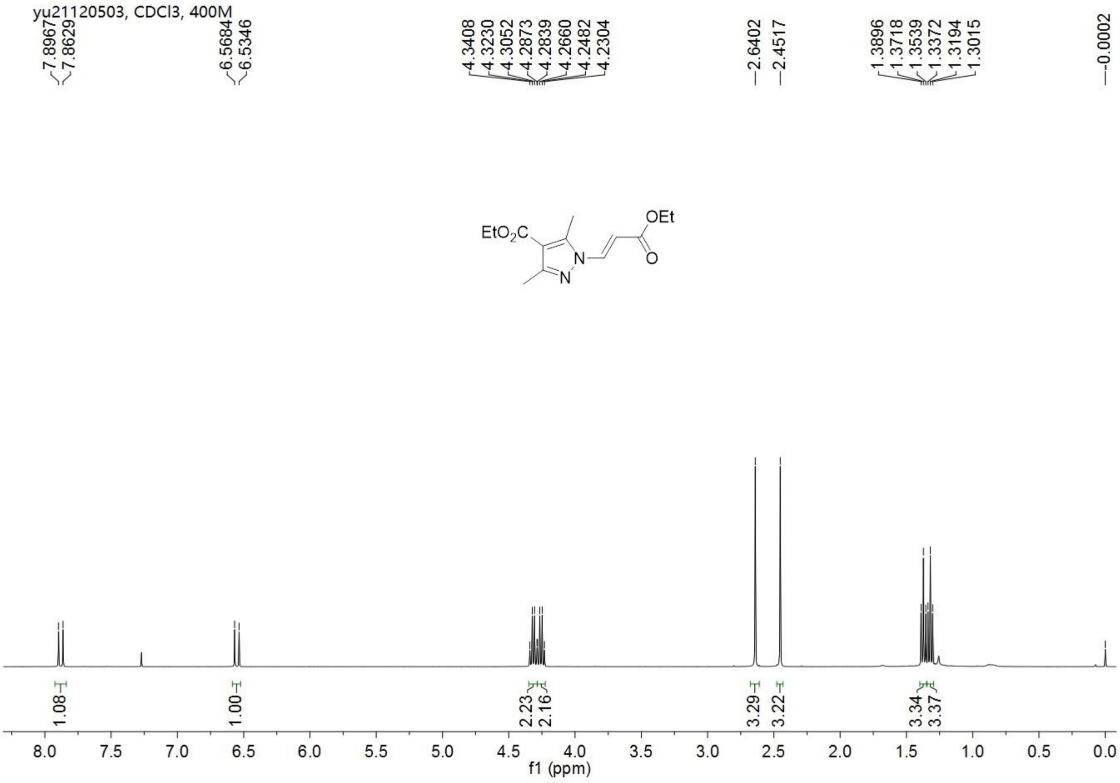

^{1}H NMR of **3r**

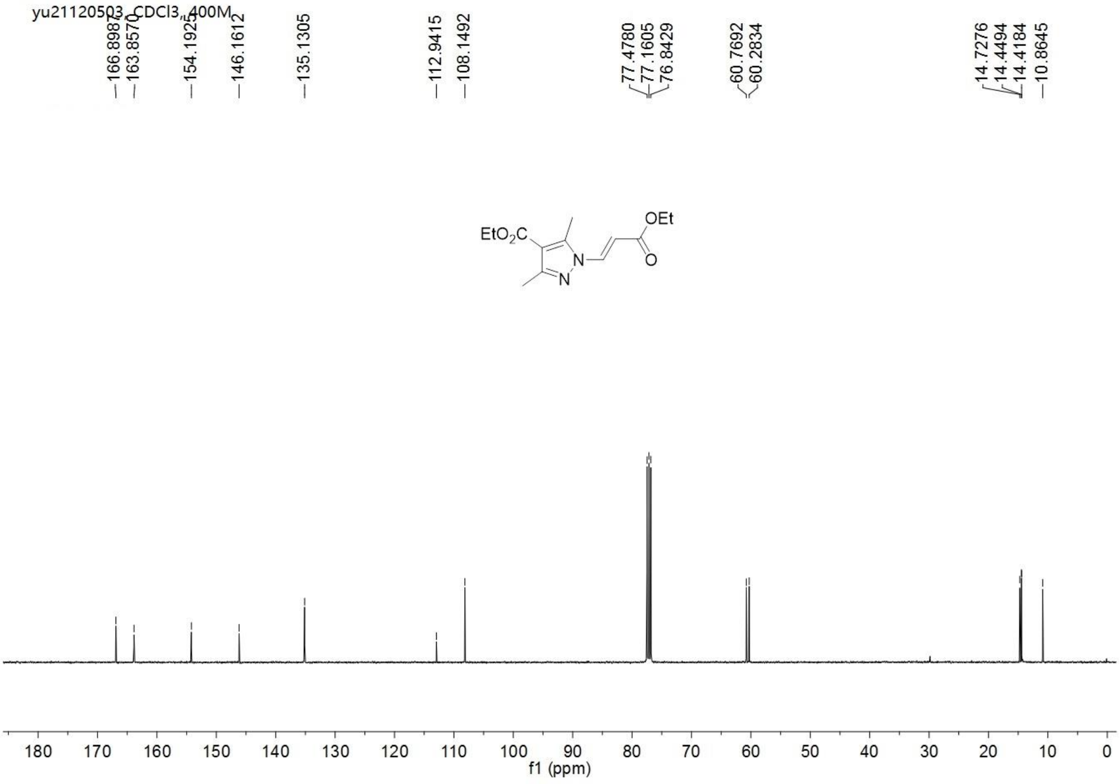

^{13}C NMR of **3r**

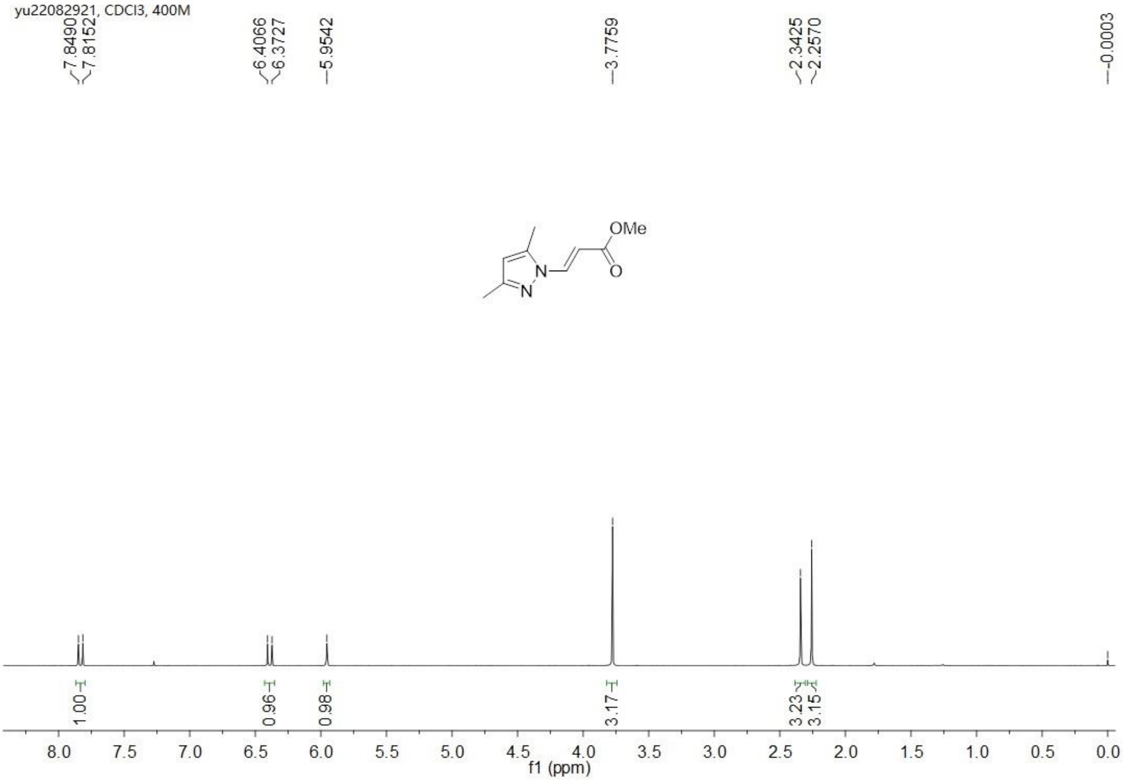

^{1}H NMR of **3s**

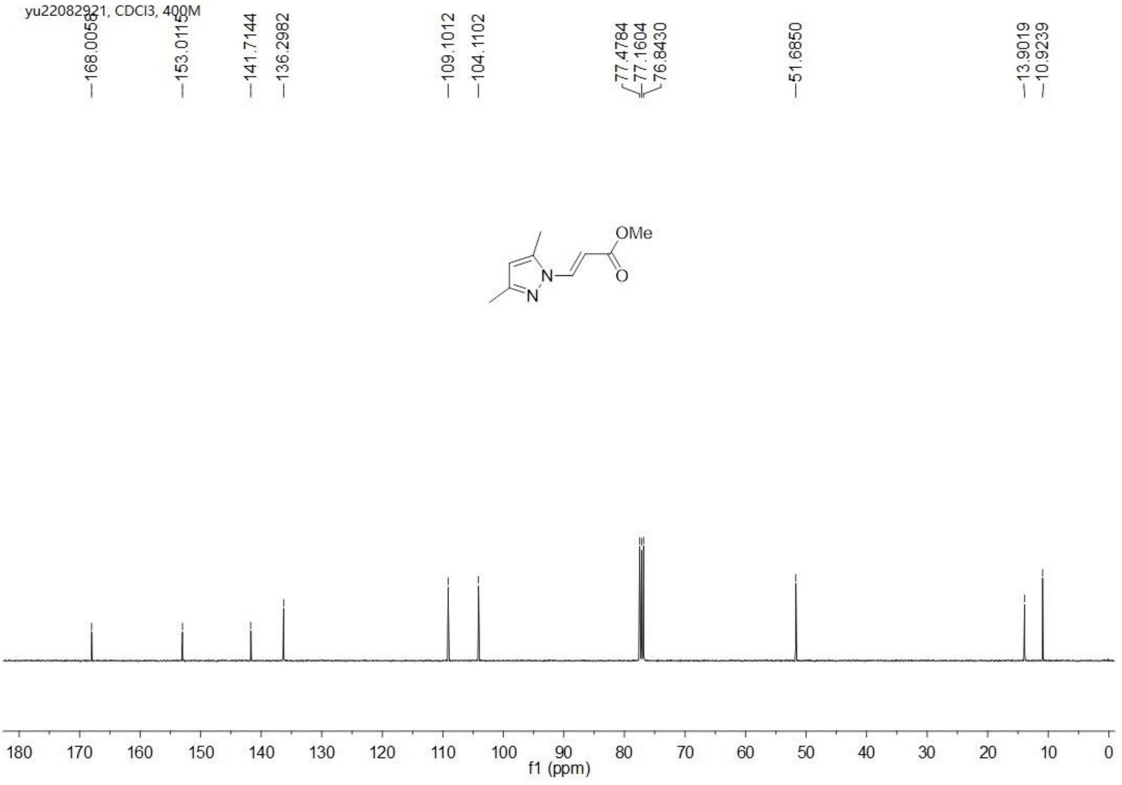

^{13}C NMR of **3s**

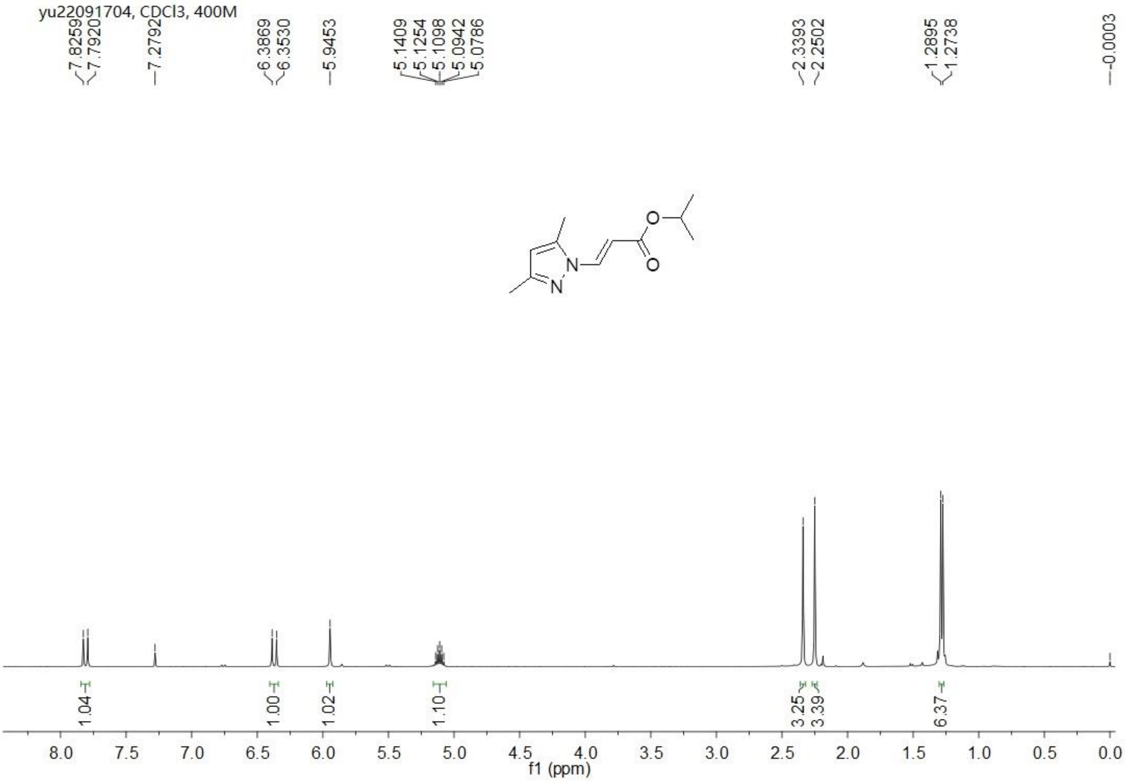

^{1}H NMR of **3t**

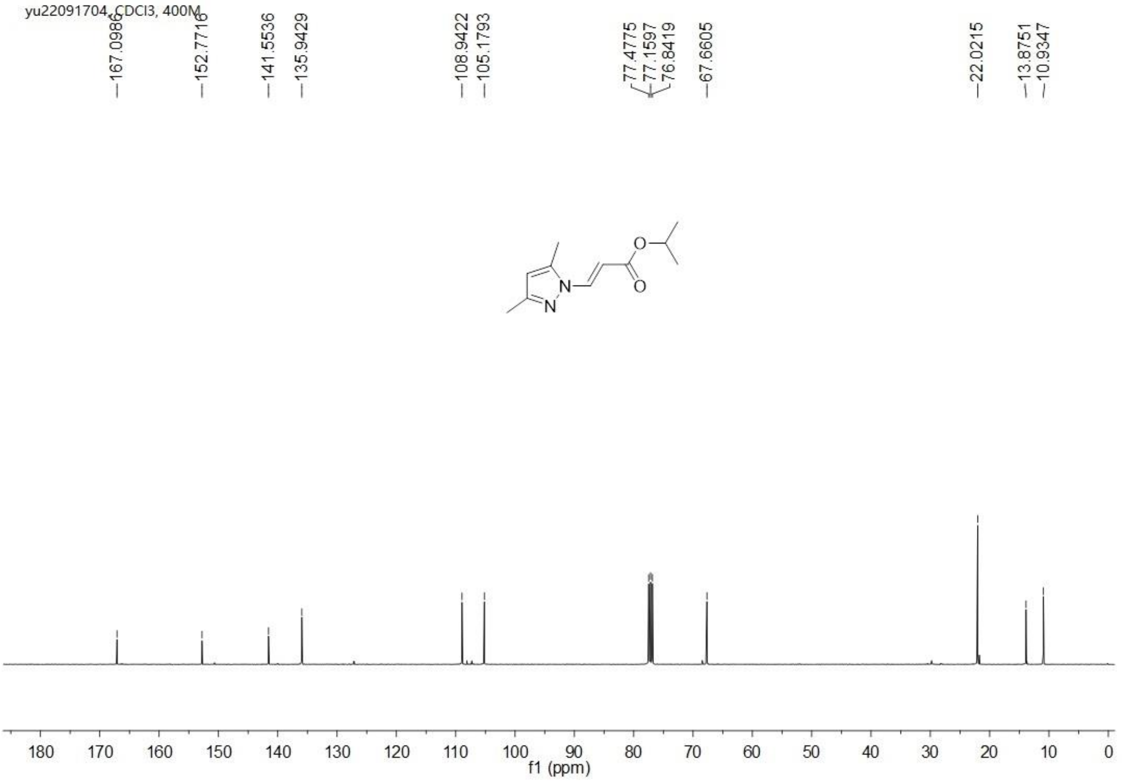

^{13}C NMR of **3t**

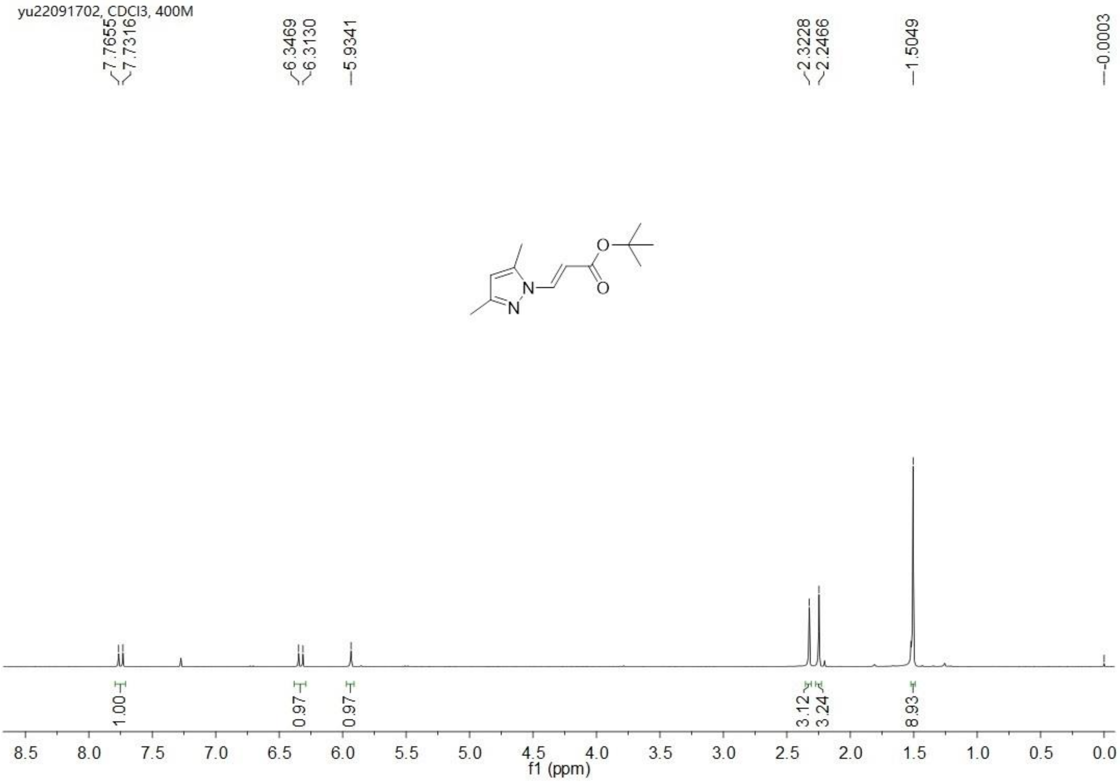

^{1}H NMR of **3u**

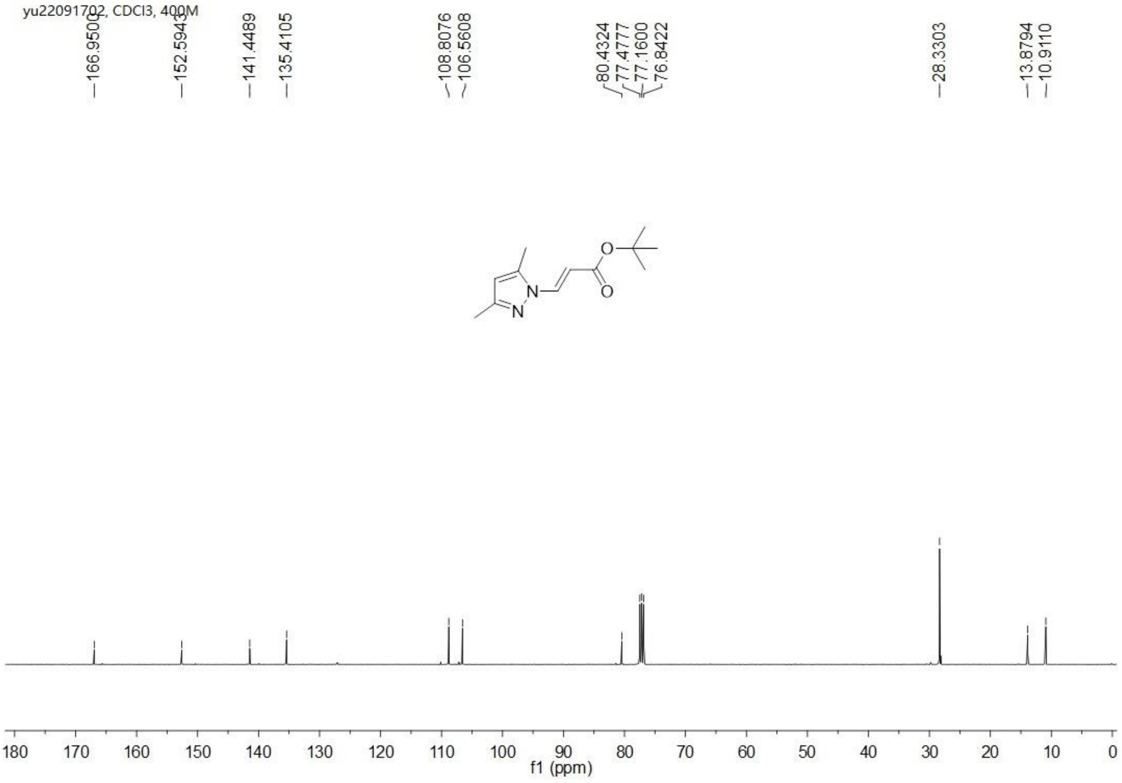

^{13}C NMR of **3u**

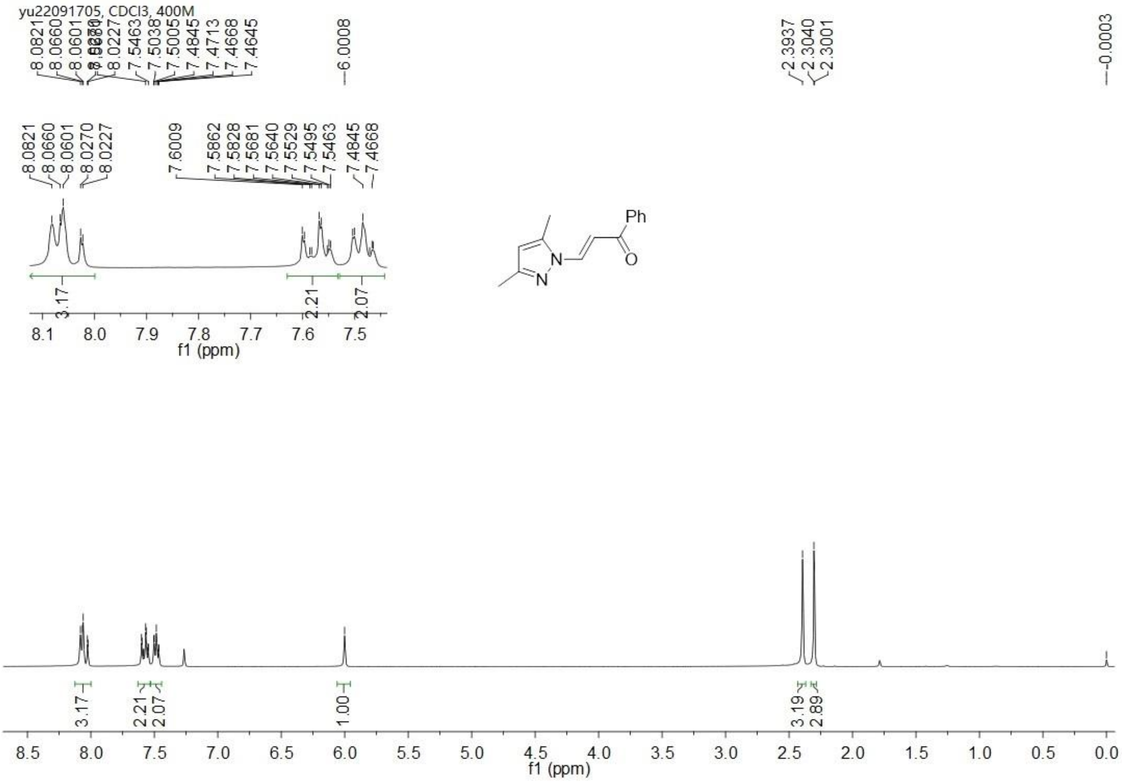

^{1}H NMR of **3v**

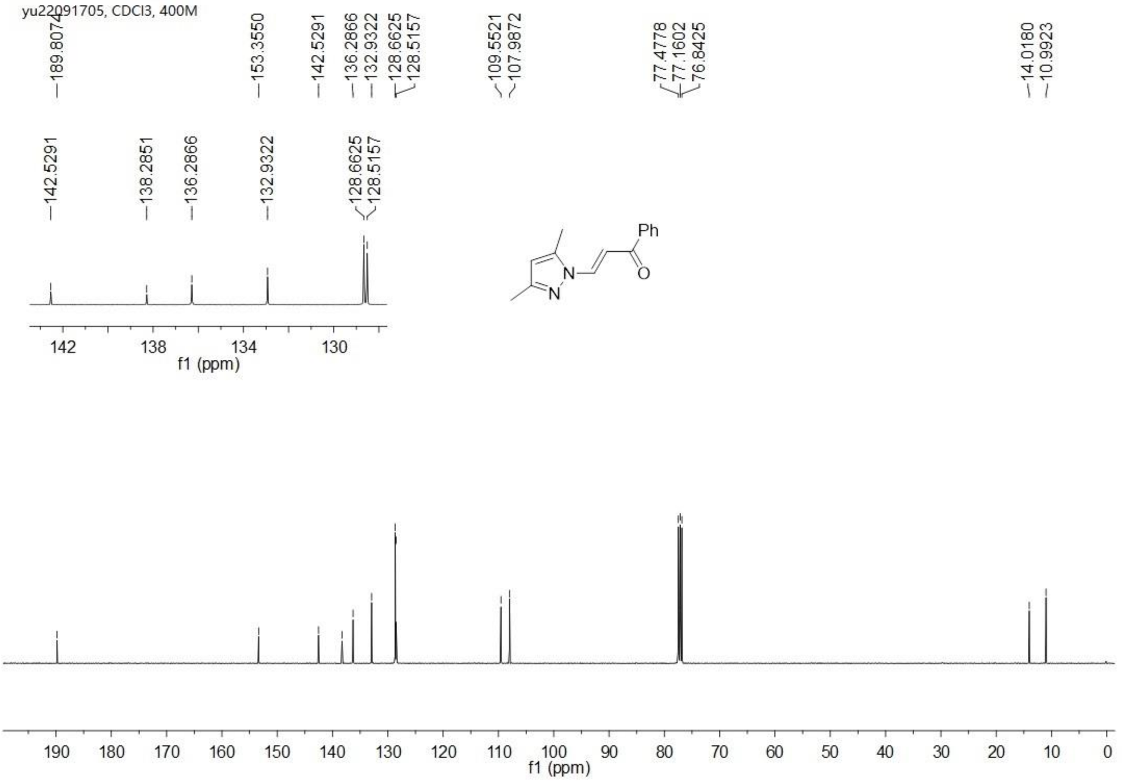

^{13}C NMR of **3v**

Copies of ^{1}H NMR and ^{13}C NMR spectra of compounds 4

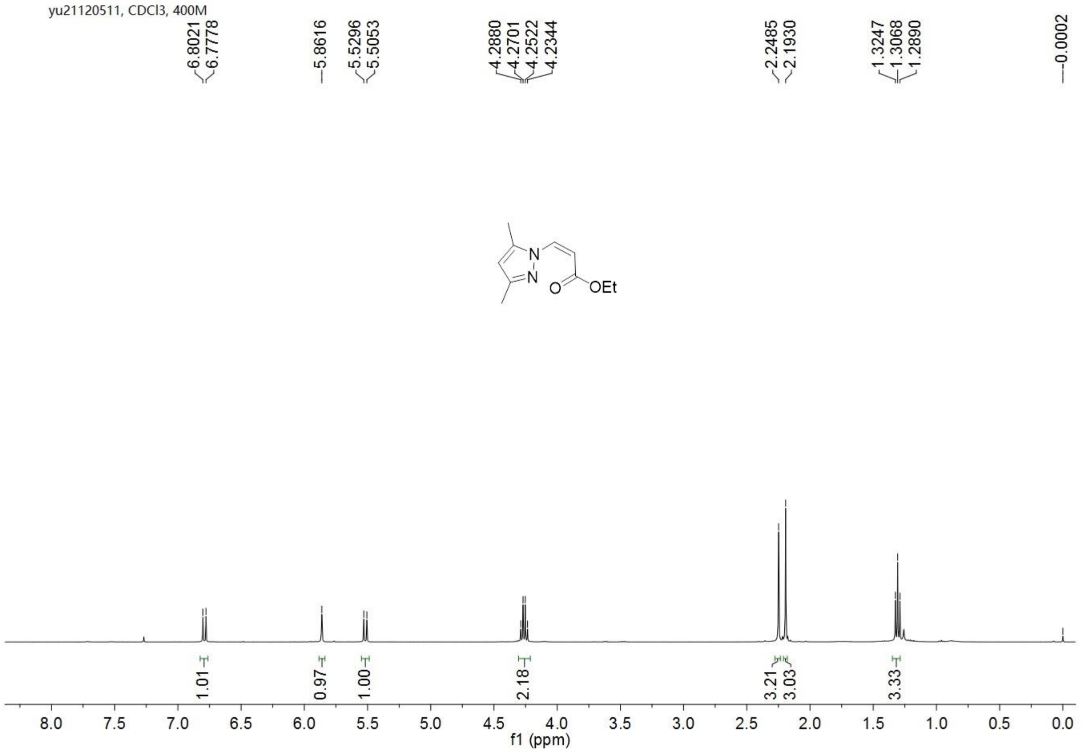

^{1}H NMR of **4a**

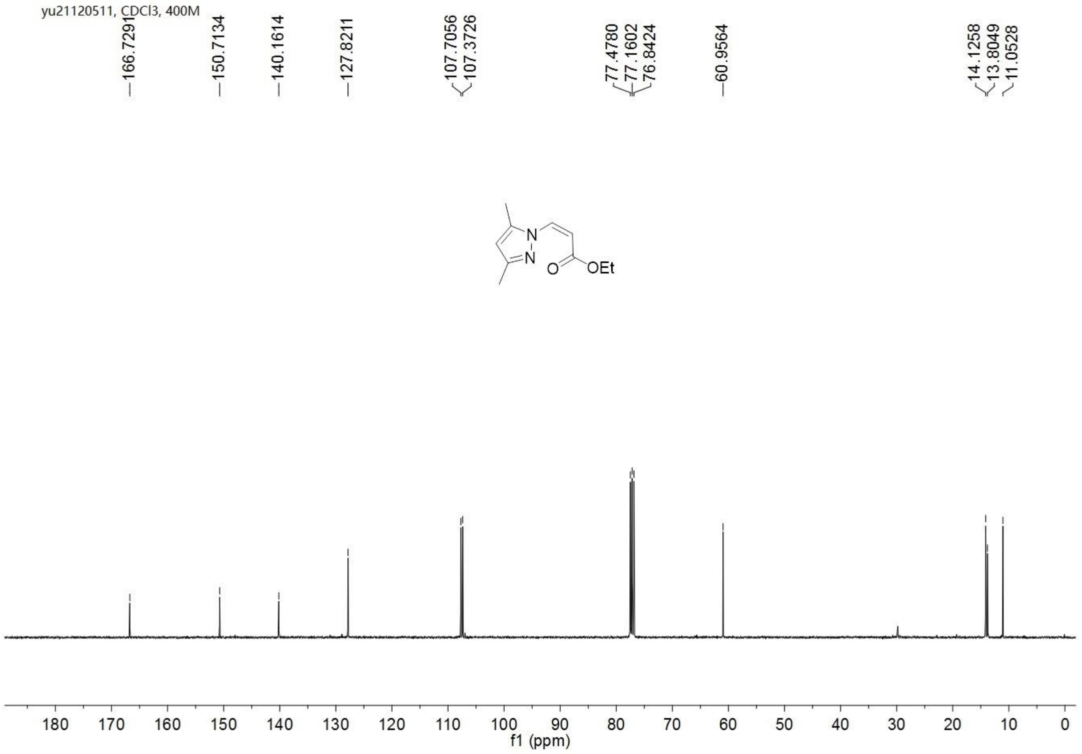

^{13}C NMR of **4a**

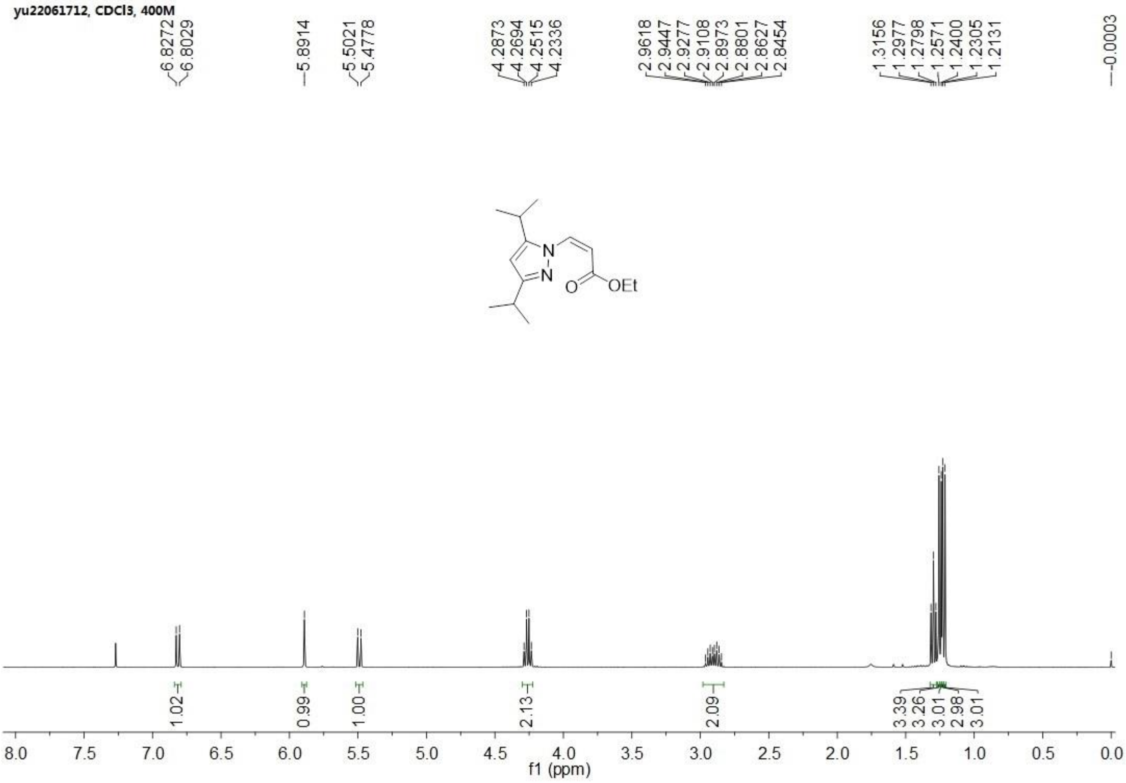

^{1}H NMR of **4b**

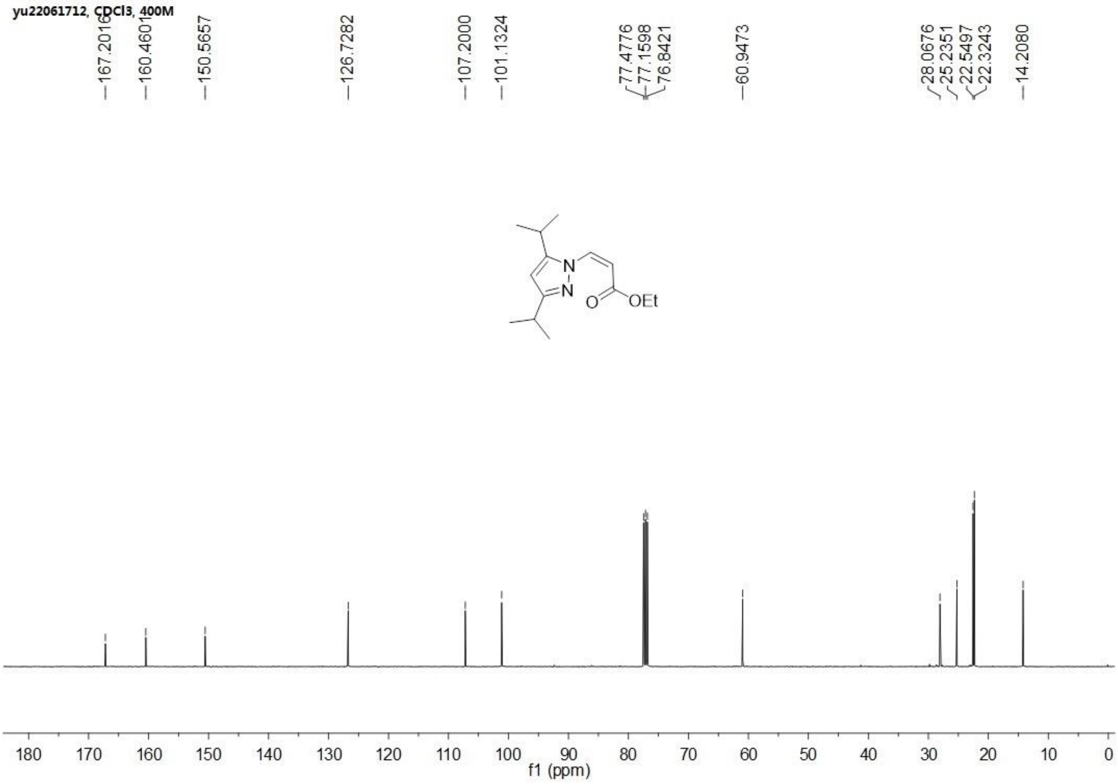

^{13}C NMR of **4b**

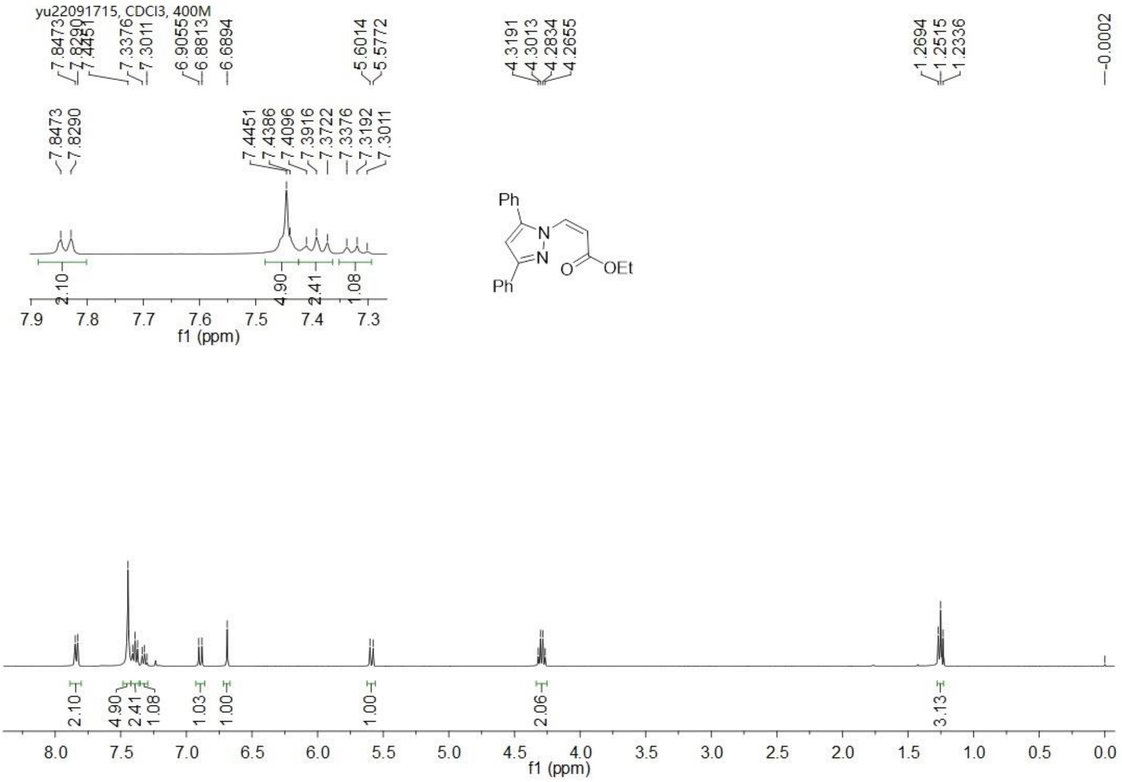

^{1}H NMR of **4c**

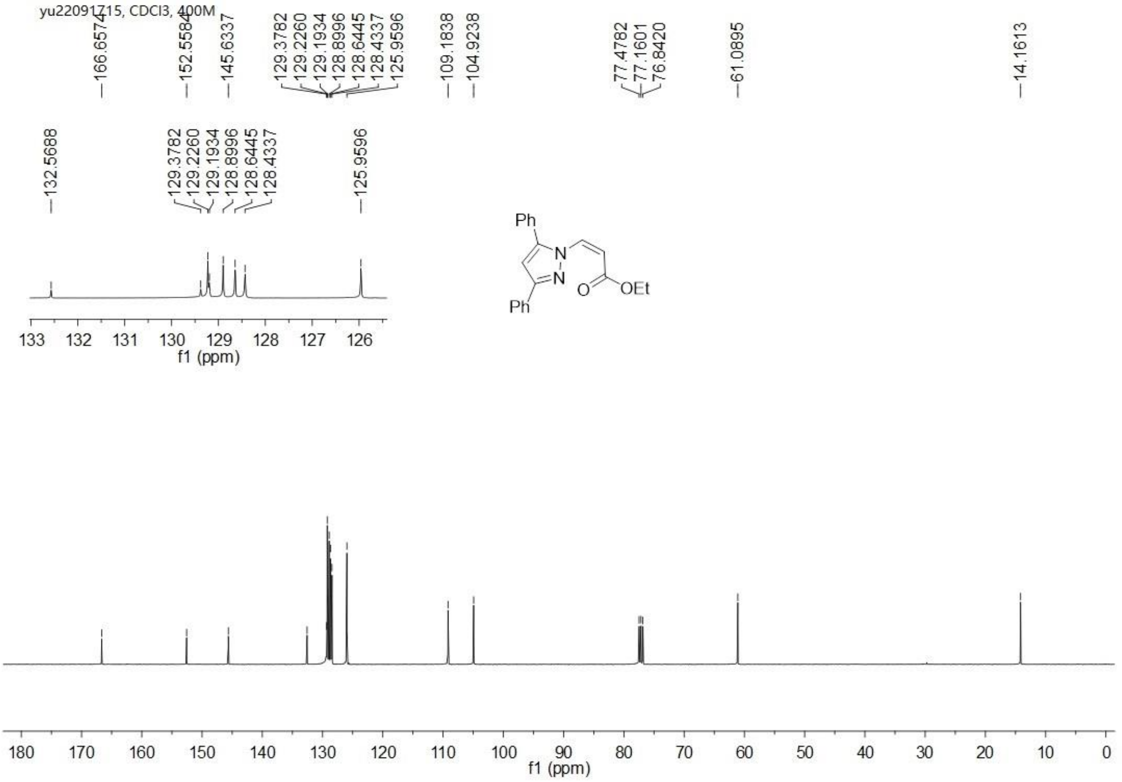

^{13}C NMR of **4c**

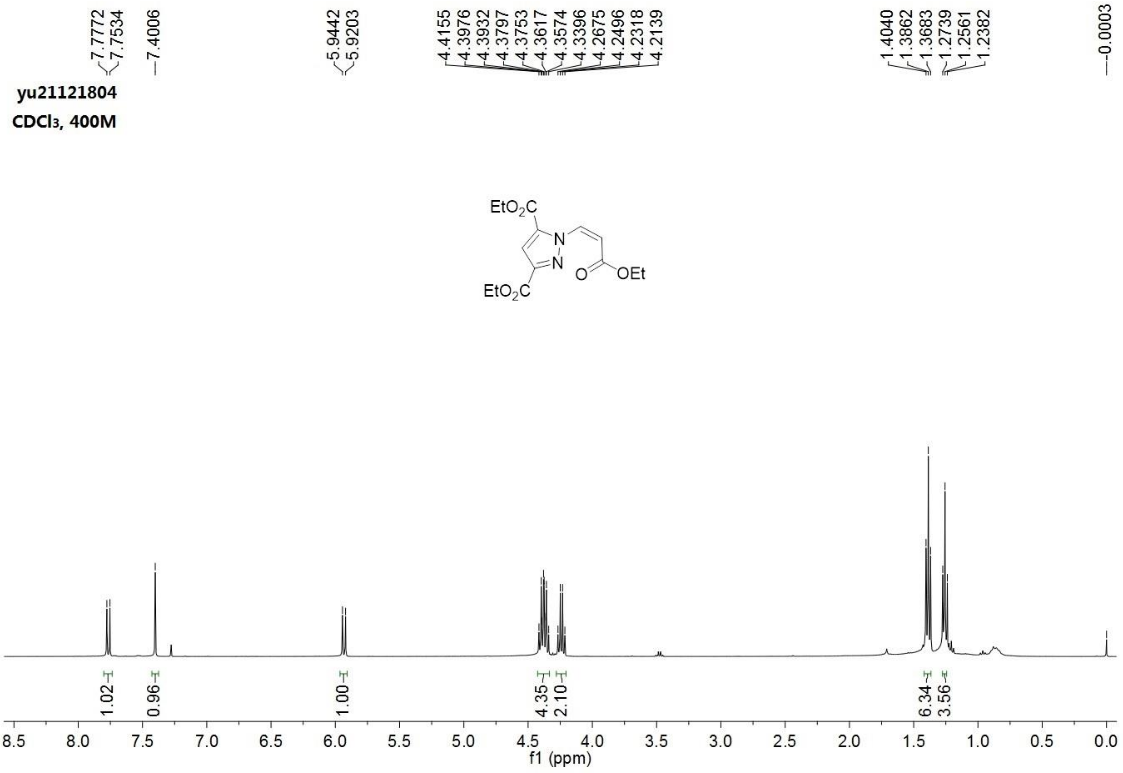

^{1}H NMR of **4d**

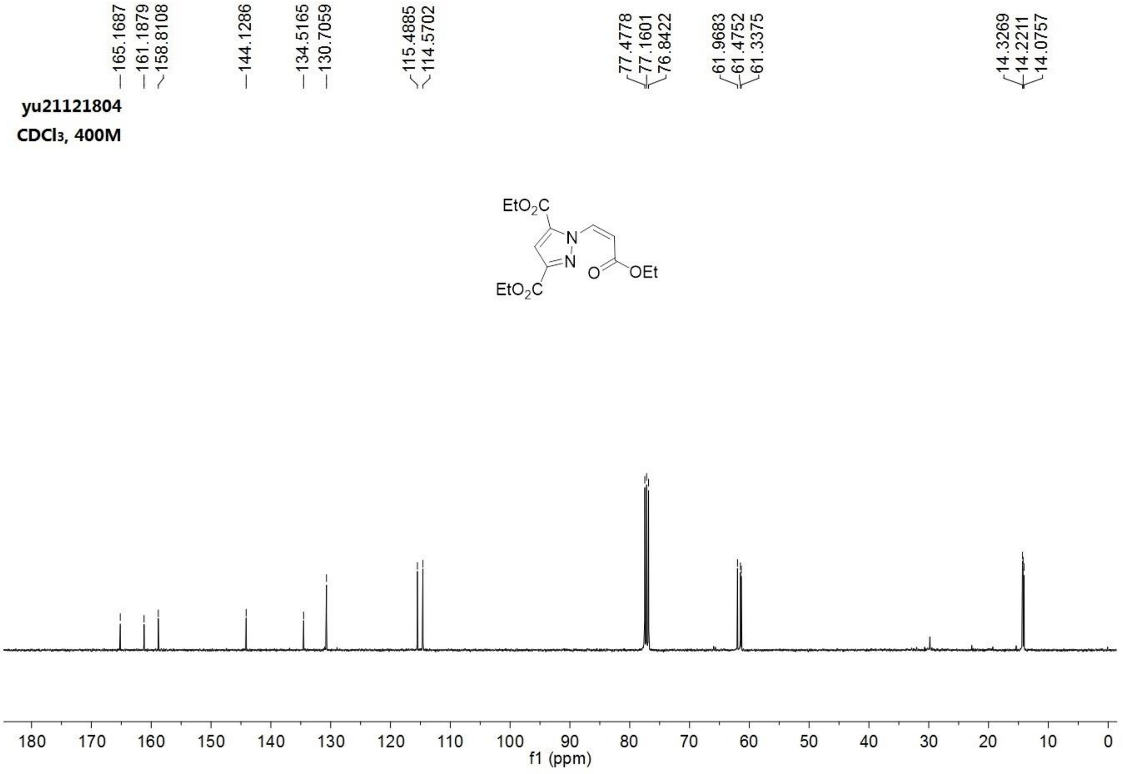

^{13}C NMR of **4d**

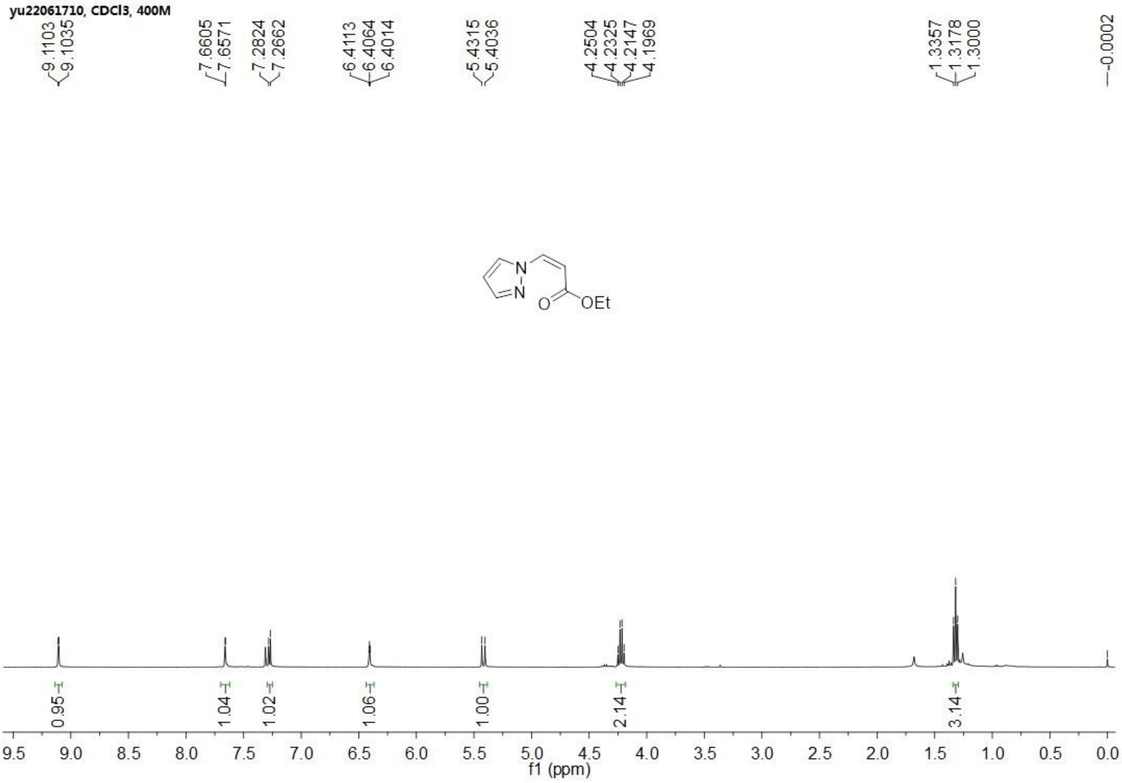

^{1}H NMR of **4e**

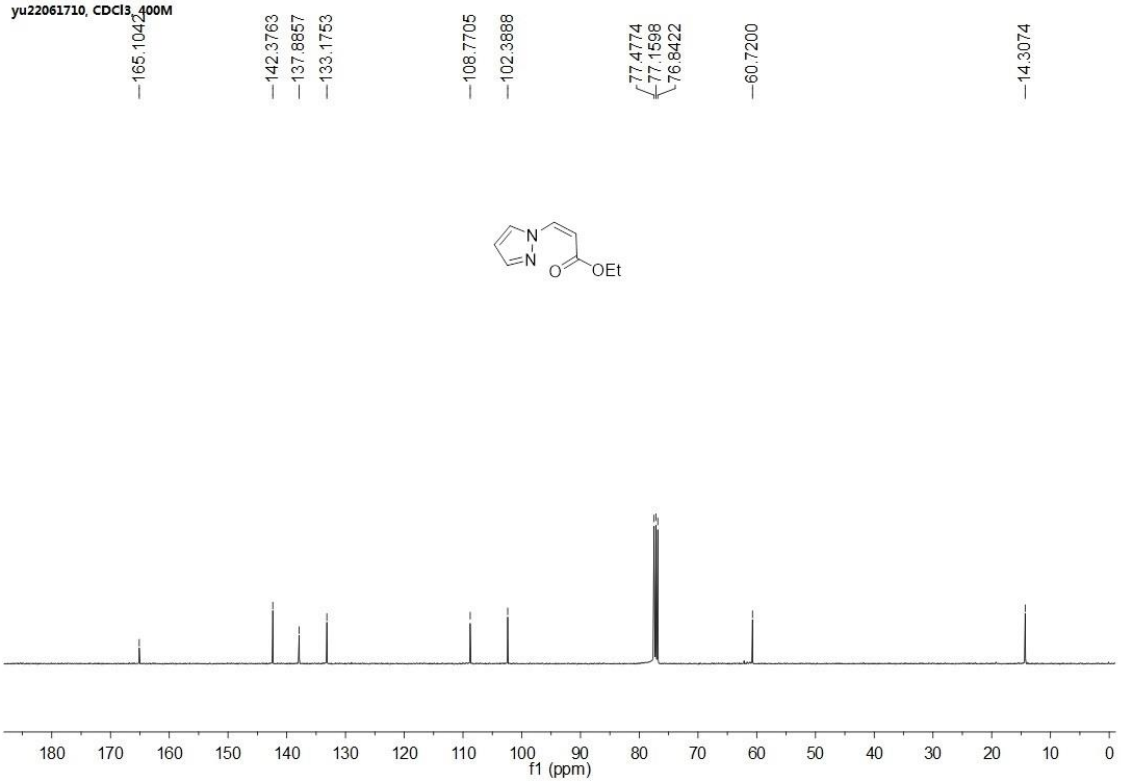

^{13}C NMR of **4e**

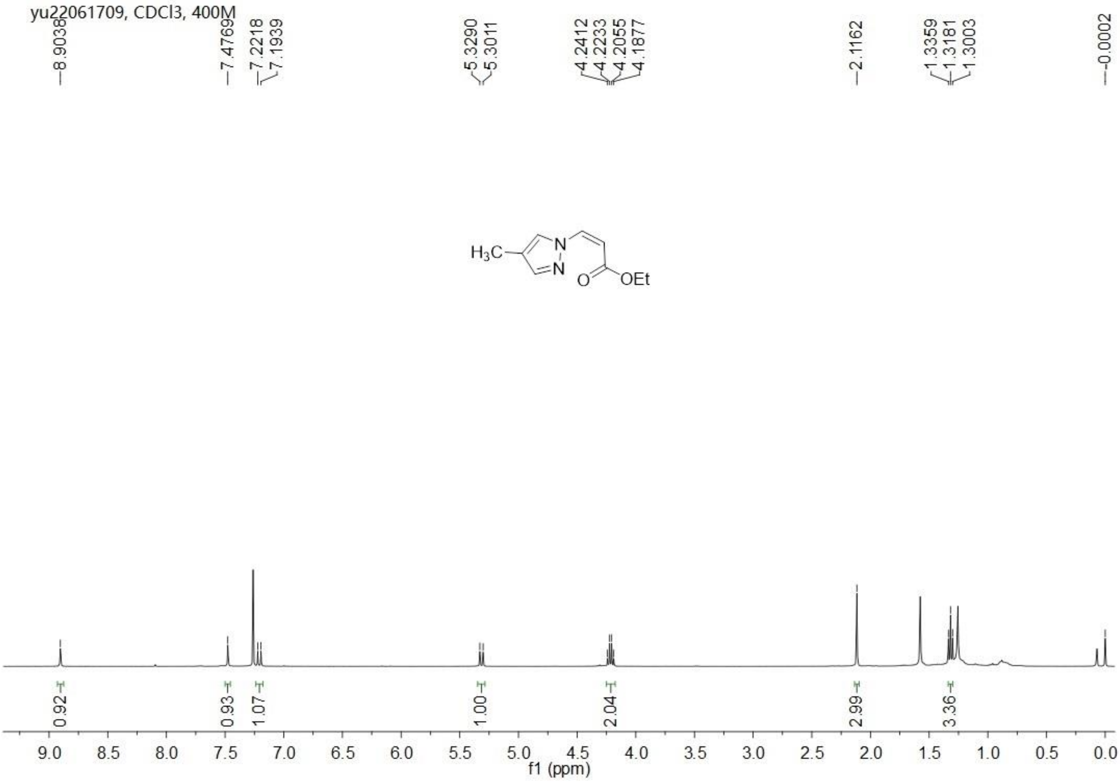

^{1}H NMR of **4f**

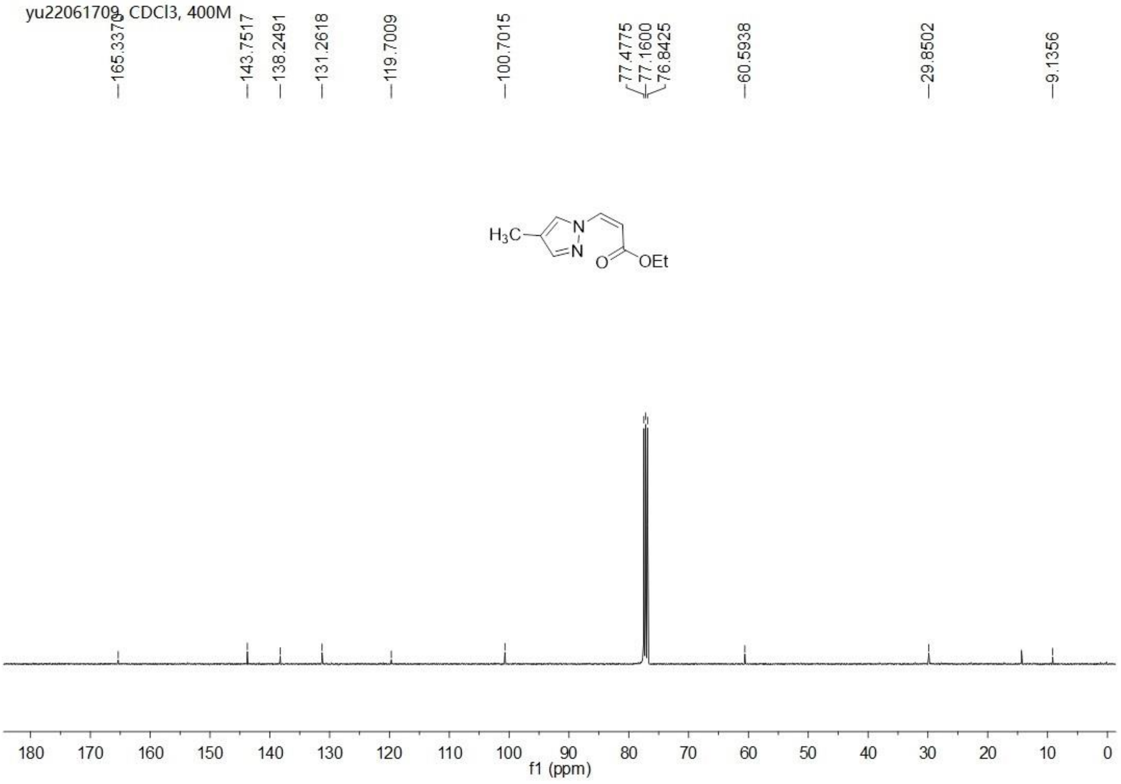

^{13}C NMR of **4f**

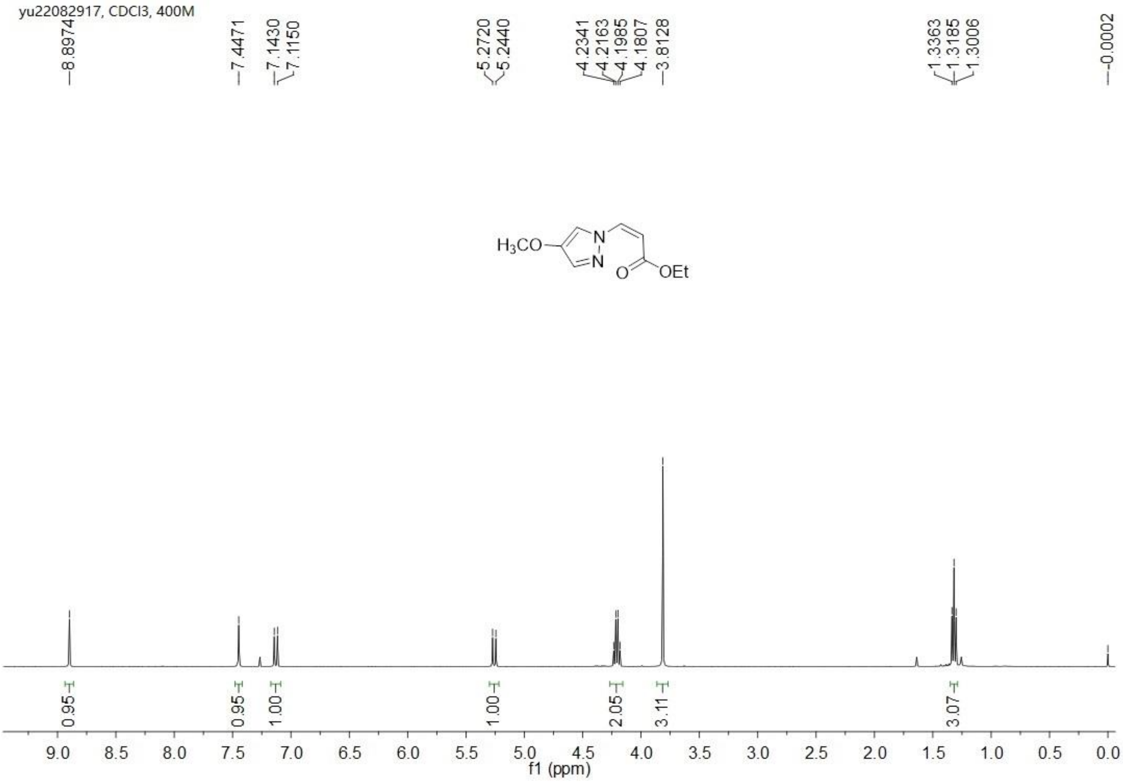

^{1}H NMR of **4g**

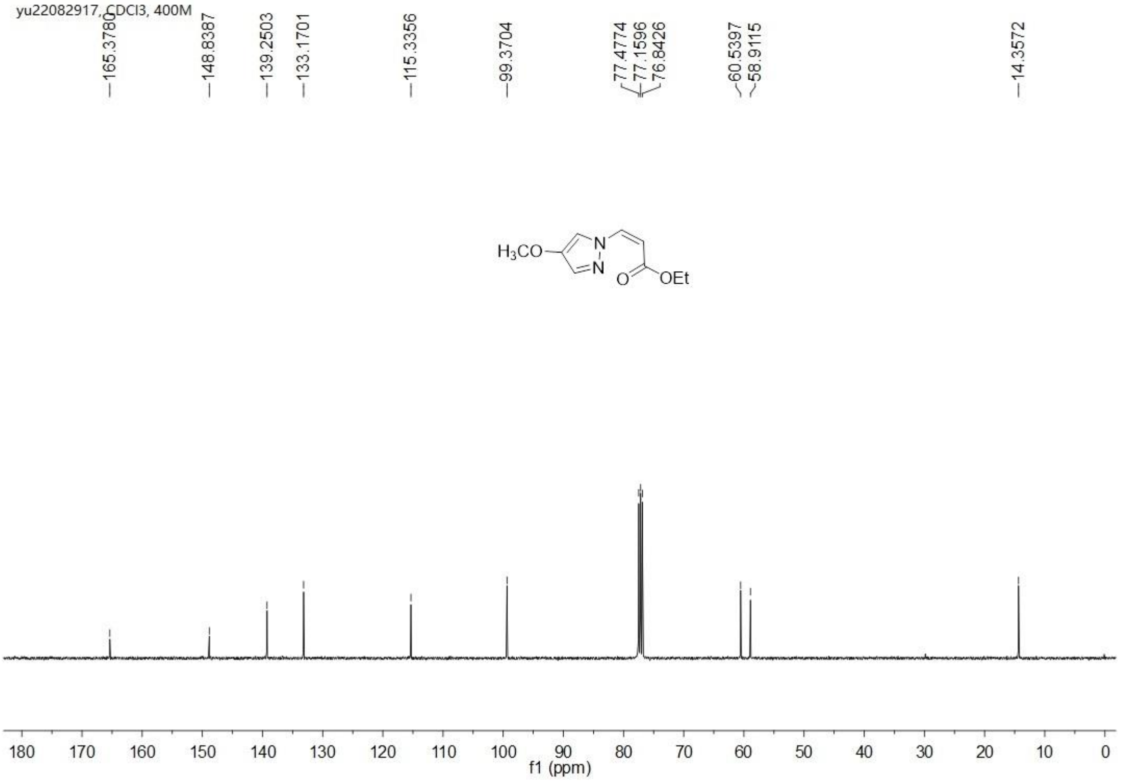

^{13}C NMR of **4g**

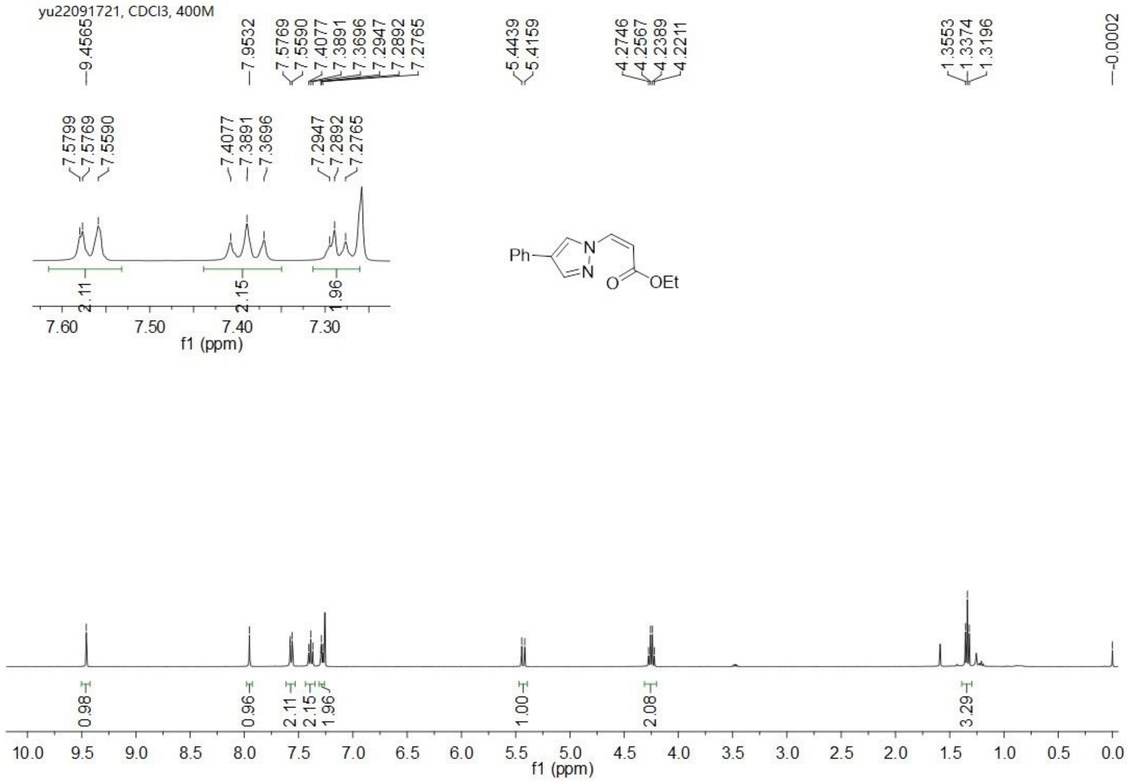

^{1}H NMR of **4h**

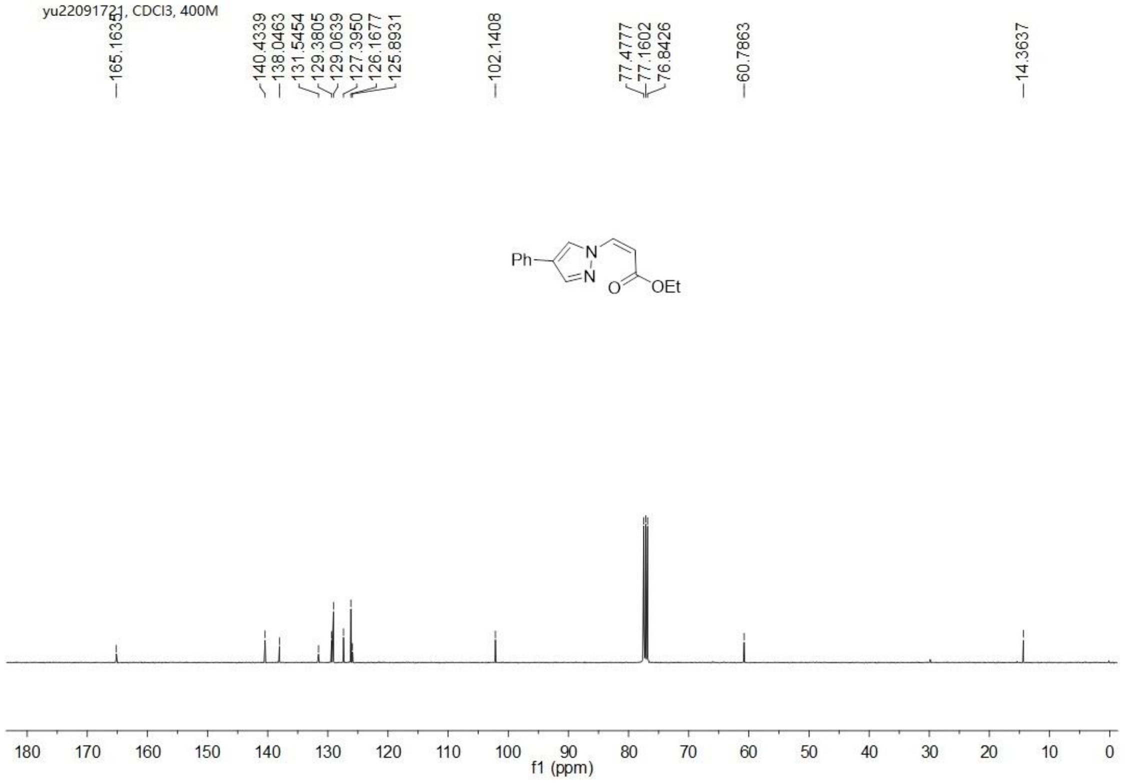

^{13}C NMR of **4h**

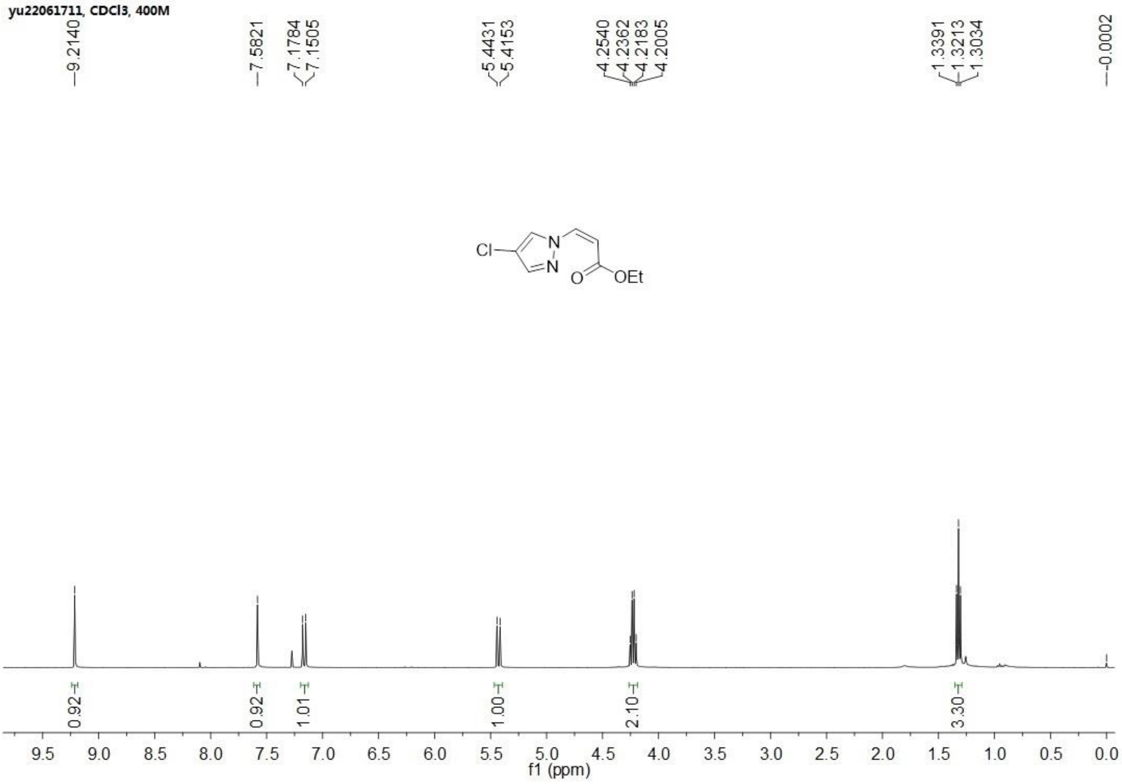

^{1}H NMR of **4i**

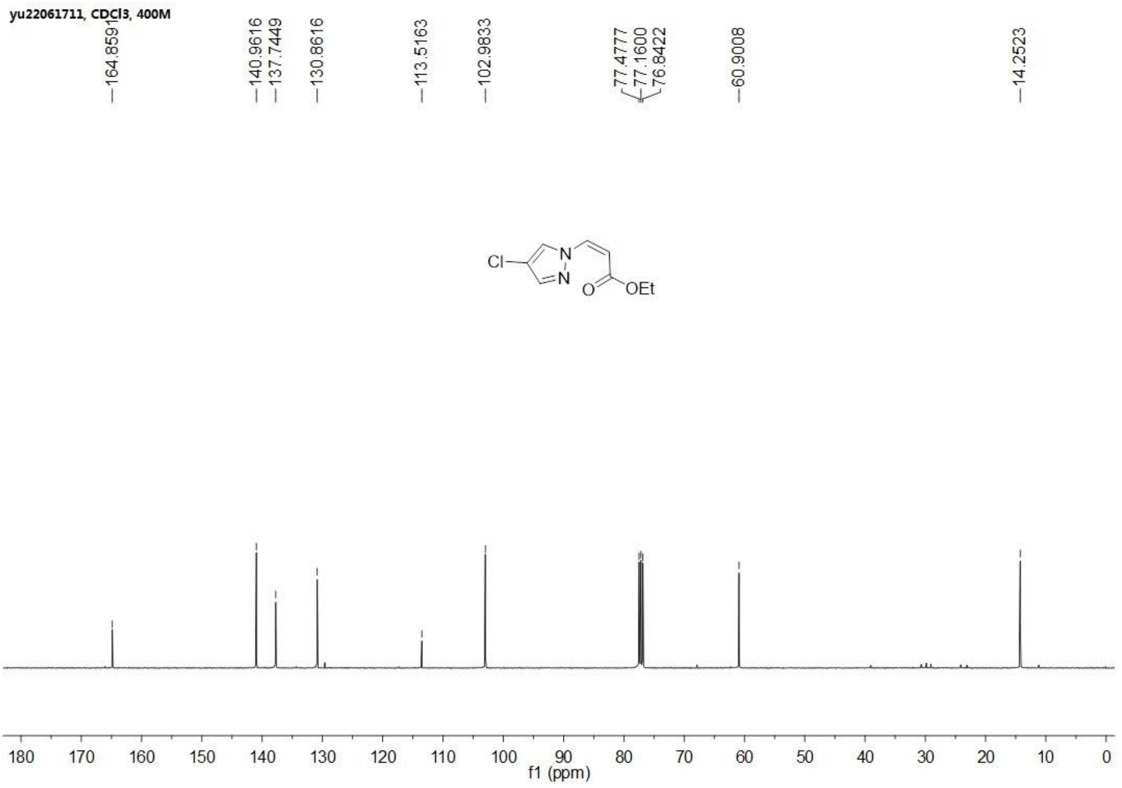

^{13}C NMR of **4i**

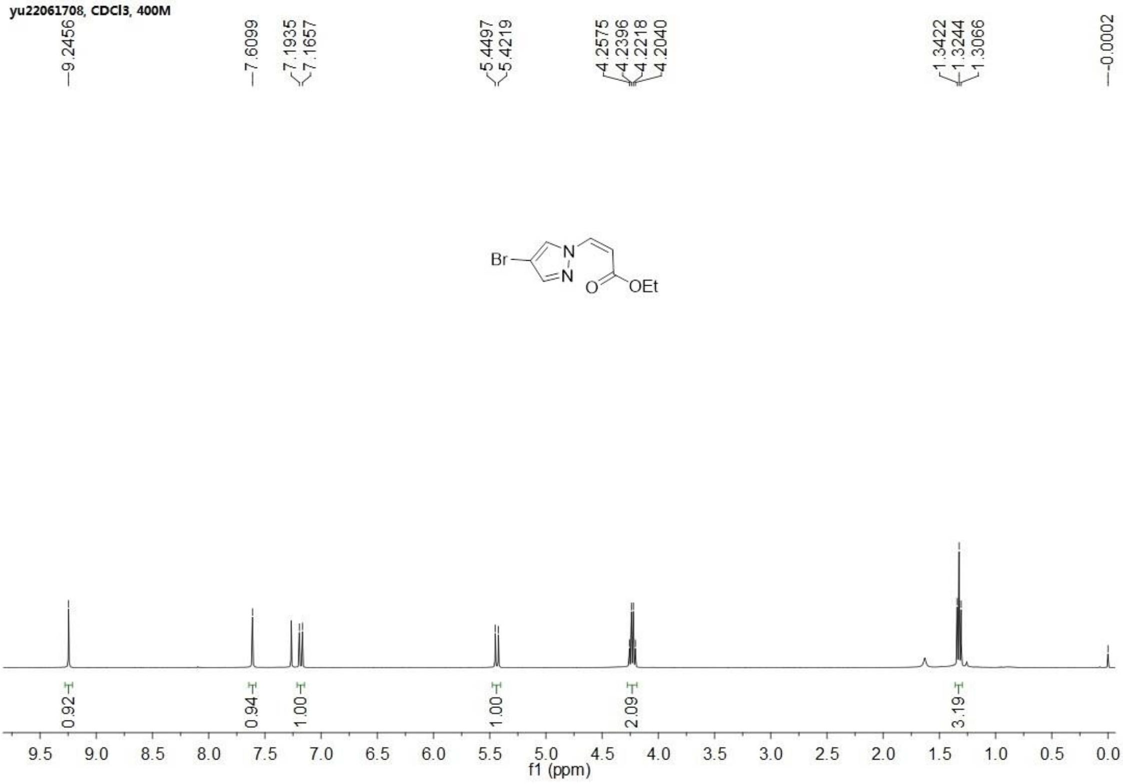

^{1}H NMR of **4j**

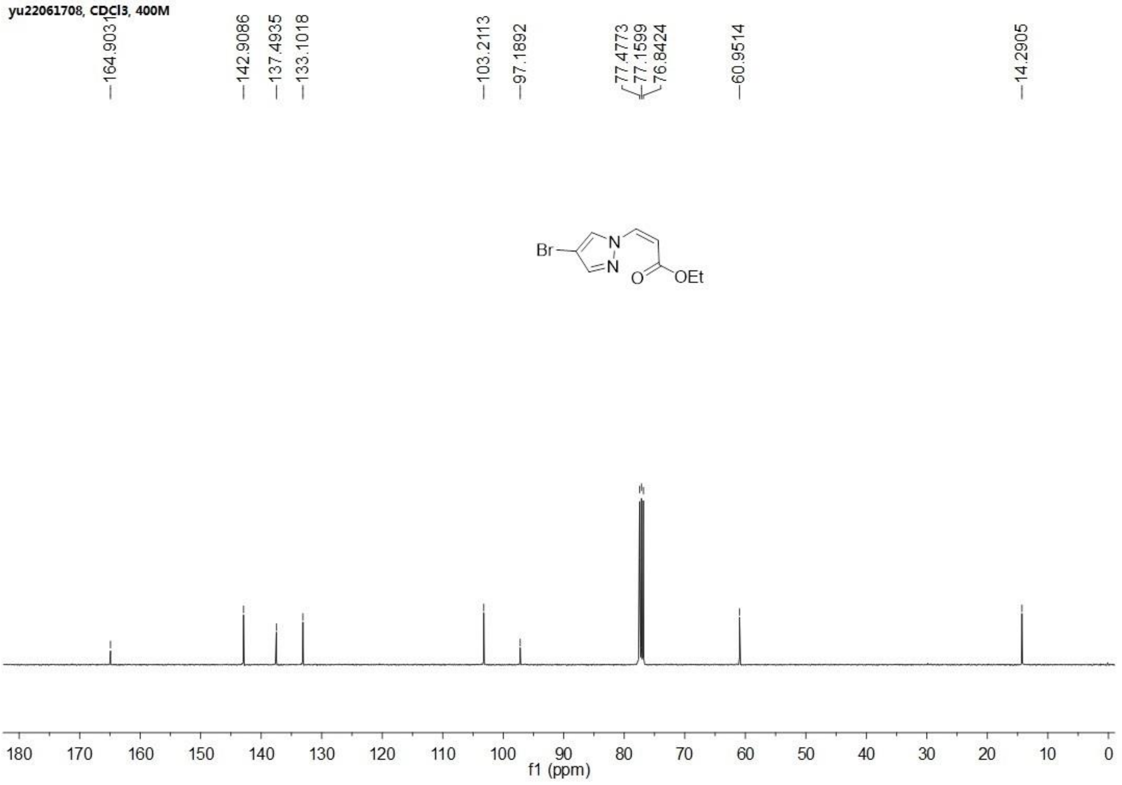

^{13}C NMR of **4j**

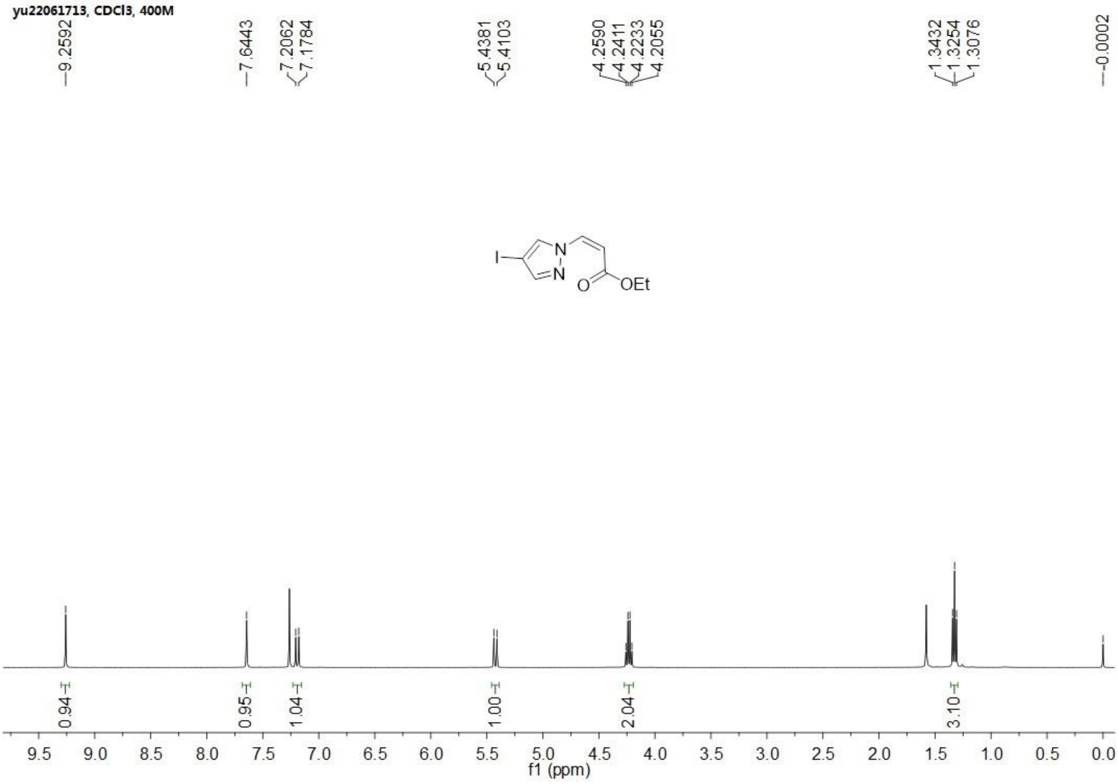

^{1}H NMR of **4k**

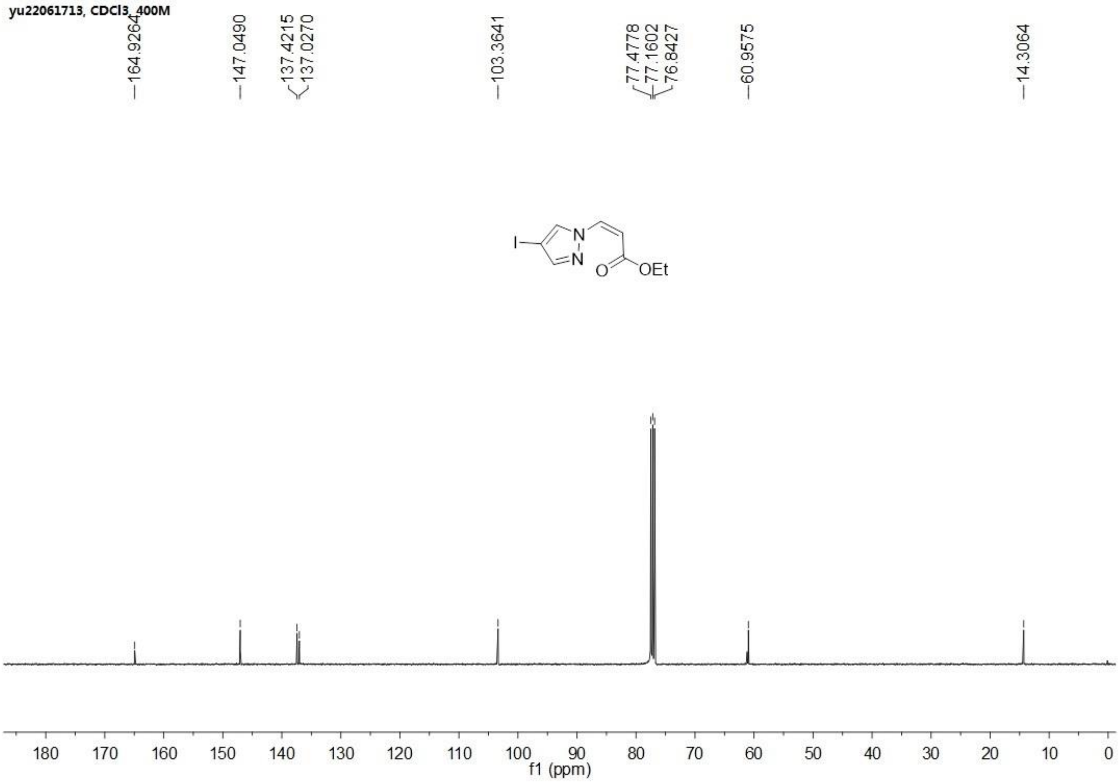

^{13}C NMR of **4k**

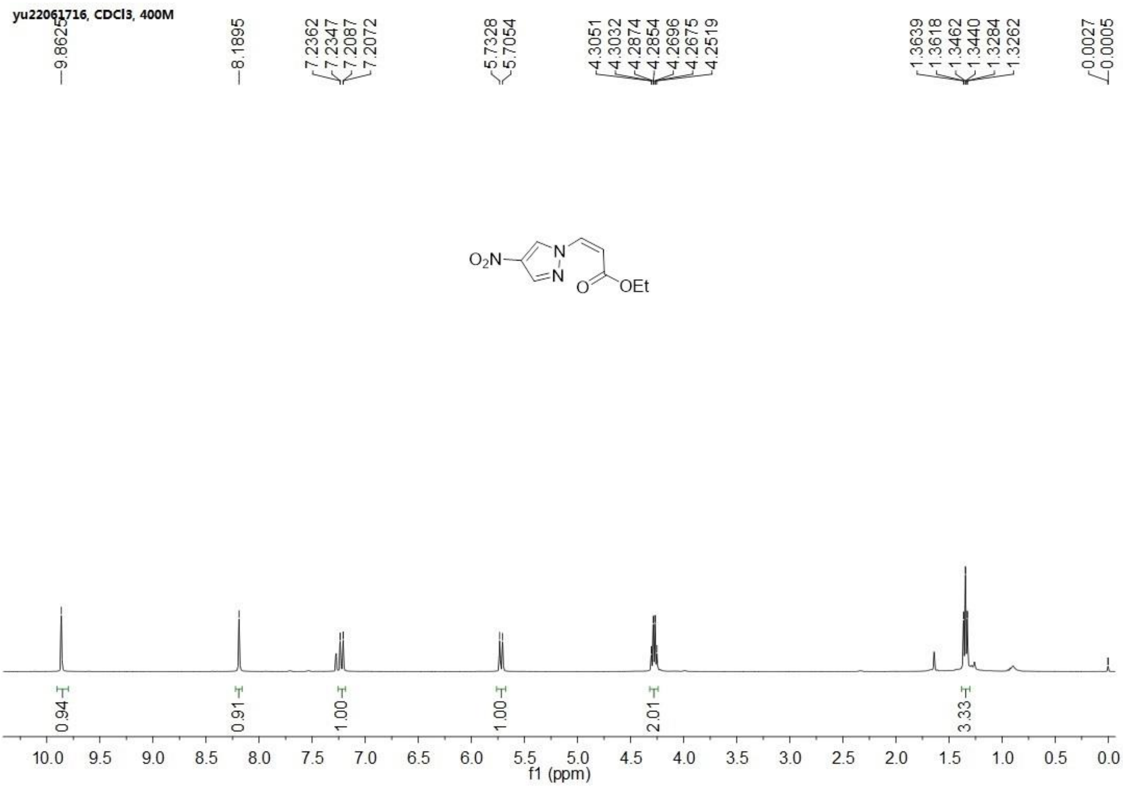

^{1}H NMR of **4l**

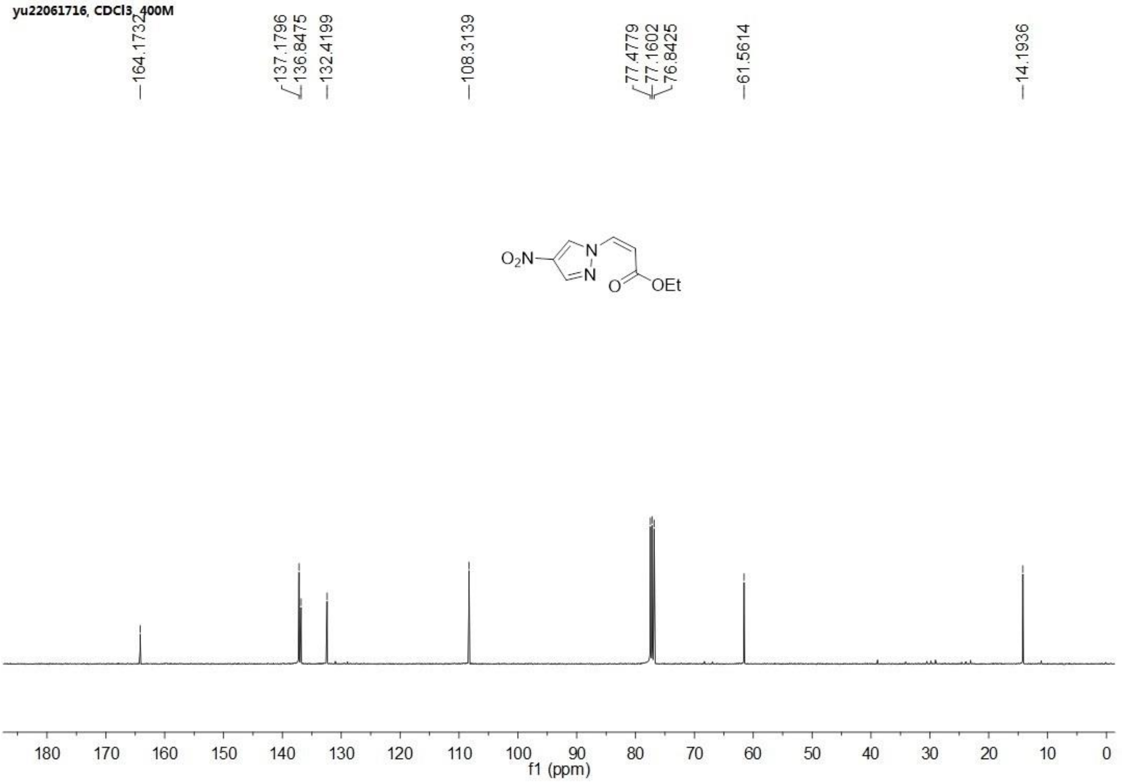

^{13}C NMR of **4l**

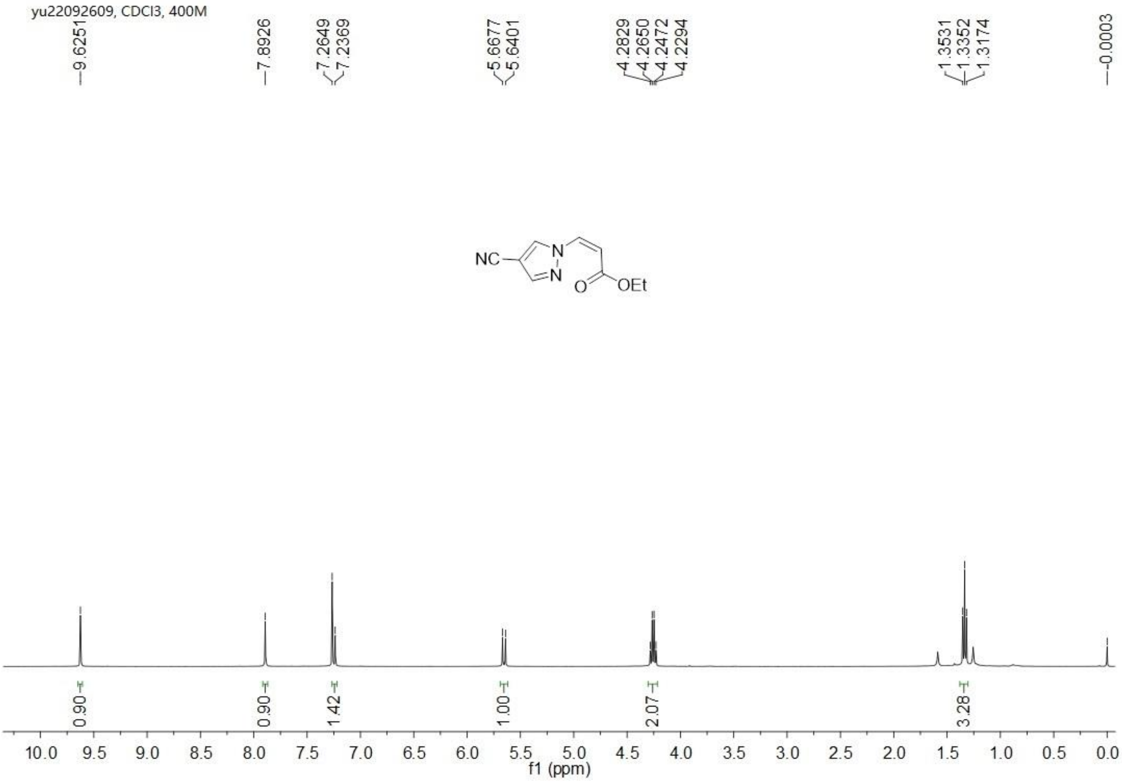

^{1}H NMR of **4m**

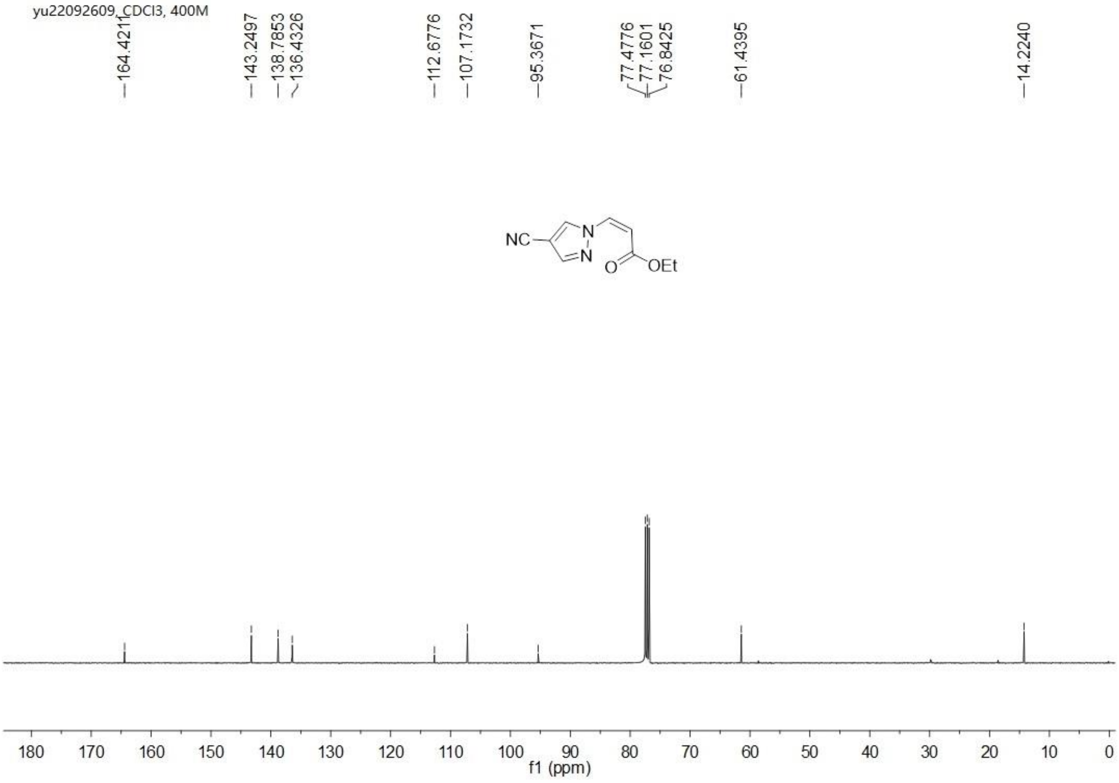

^{13}C NMR of **4m**

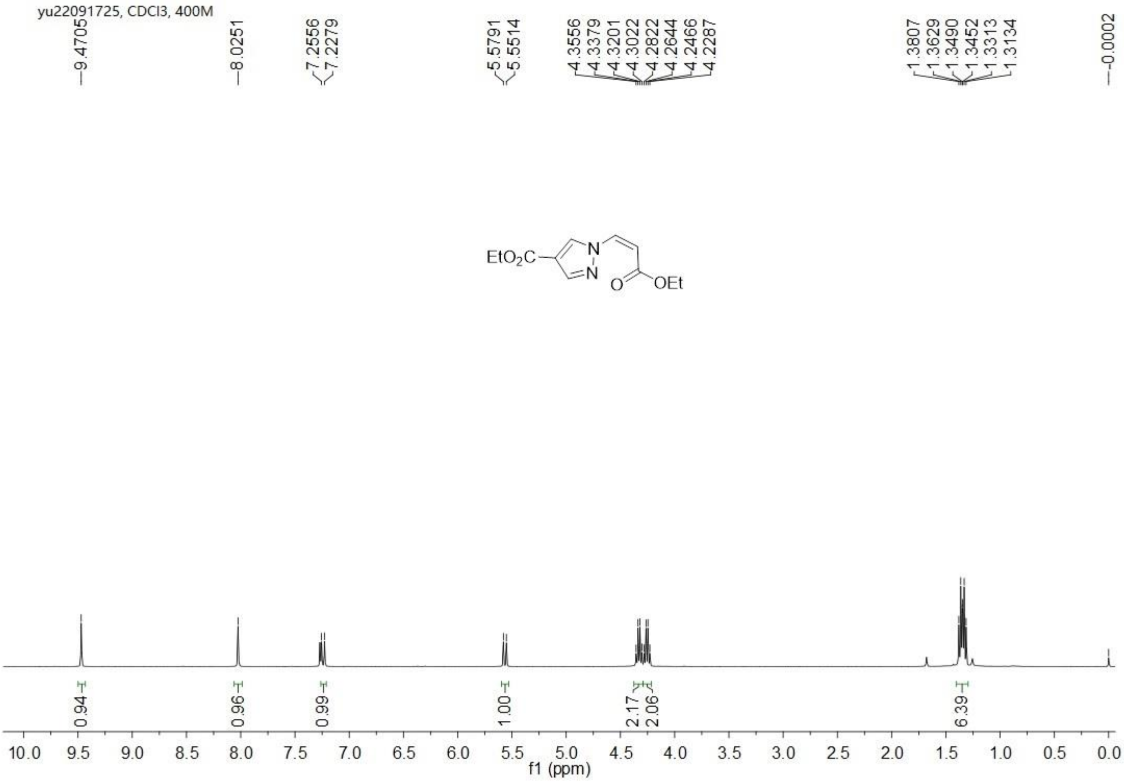

^{1}H NMR of **4n**

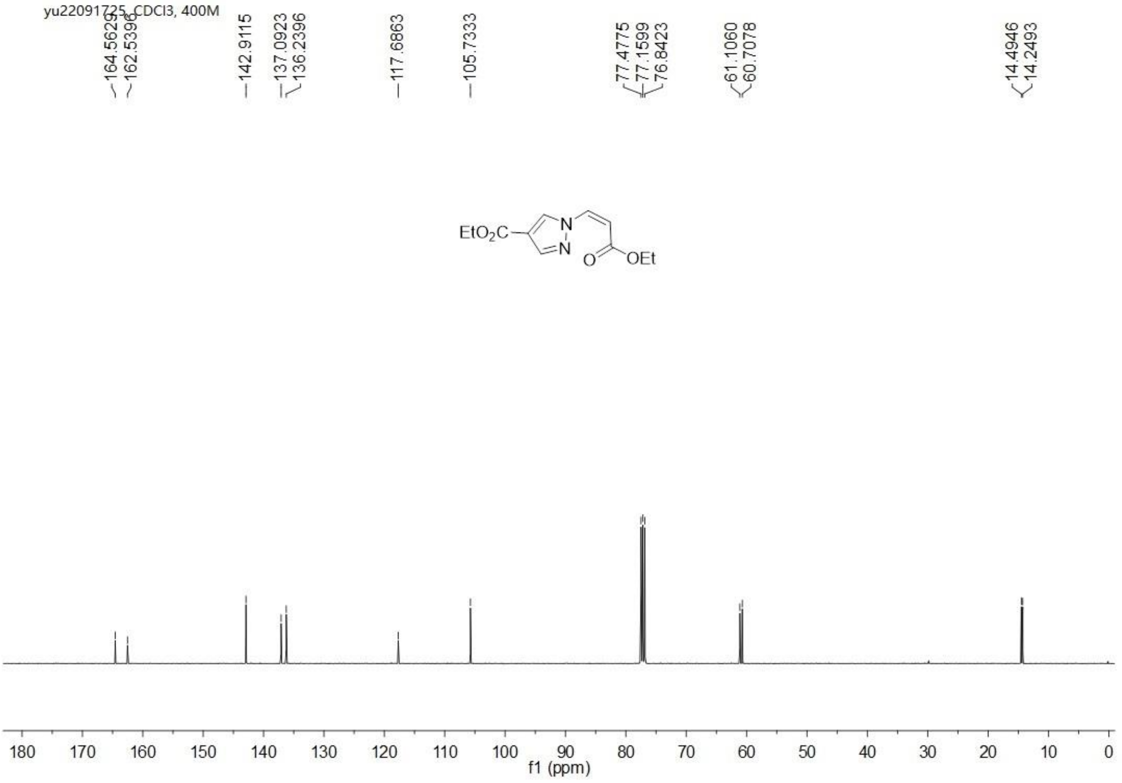

^{13}C NMR of **4n**

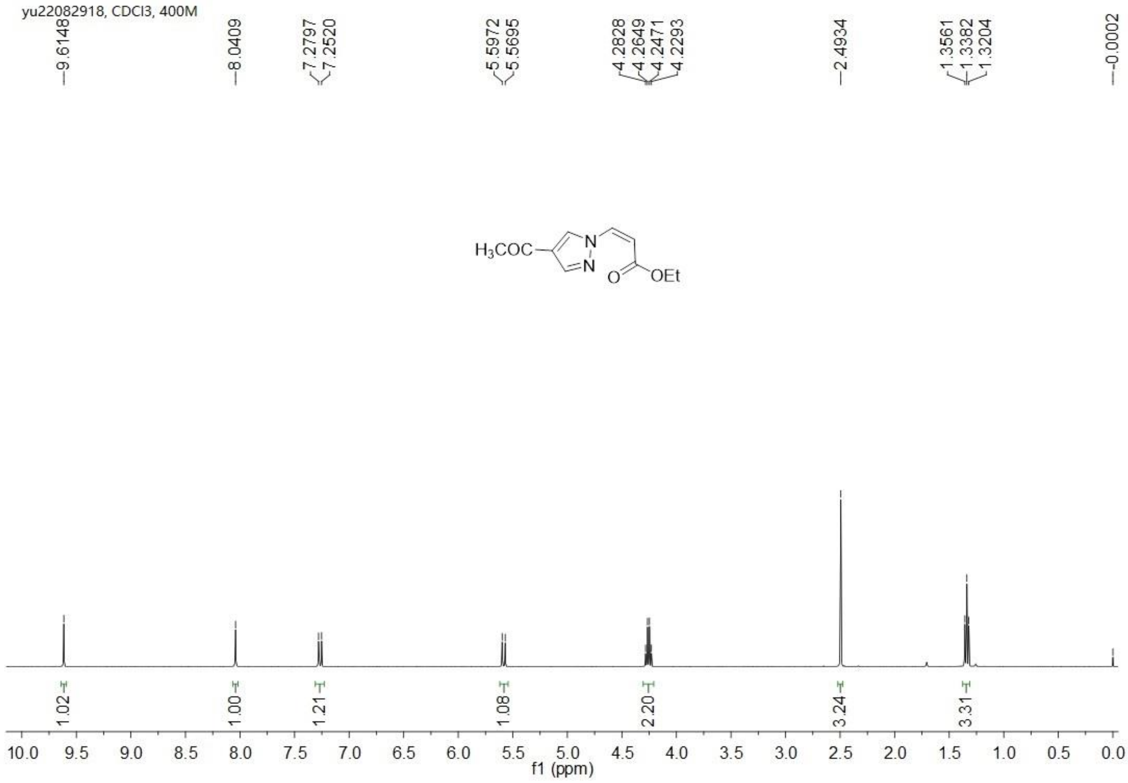

^{1}H NMR of **4o**

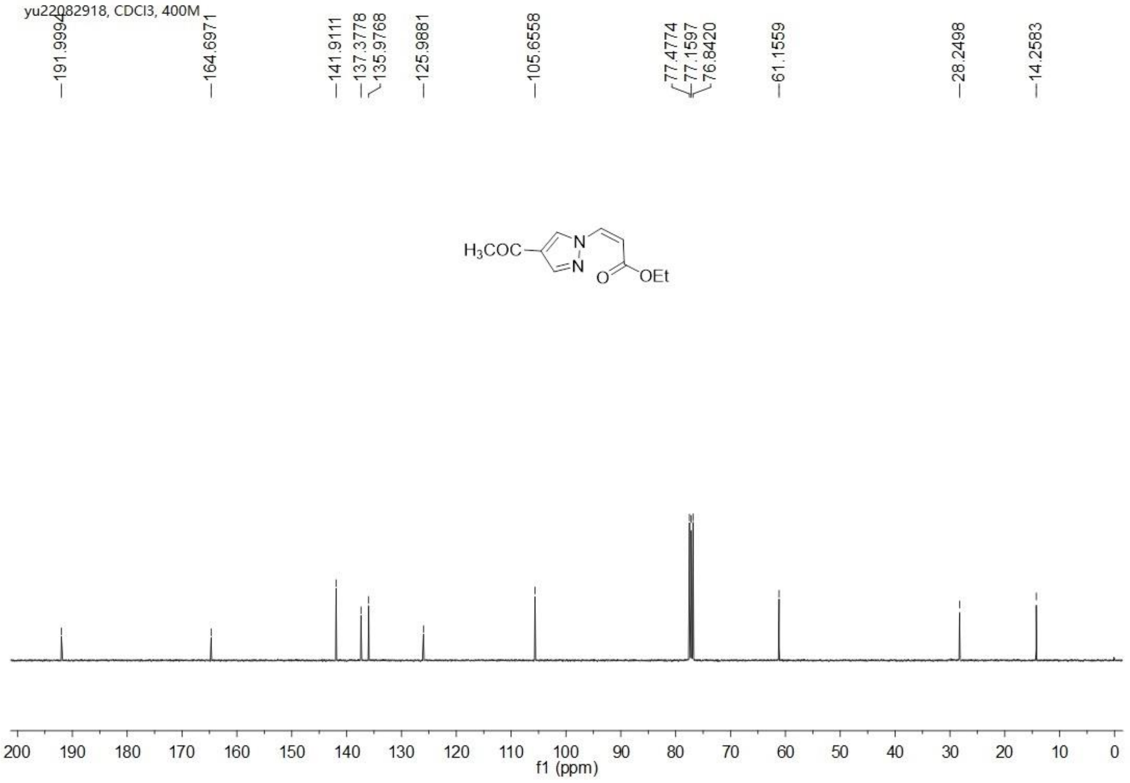

^{13}C NMR of **4o**

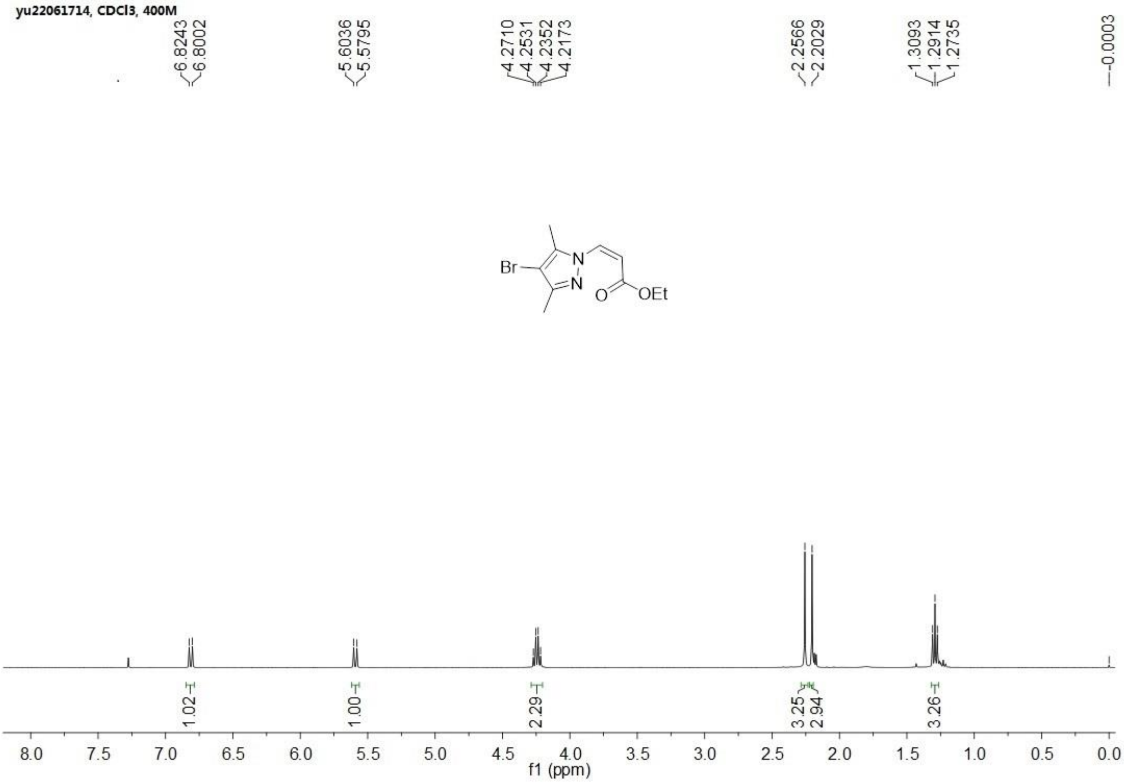

^{1}H NMR of **4p**

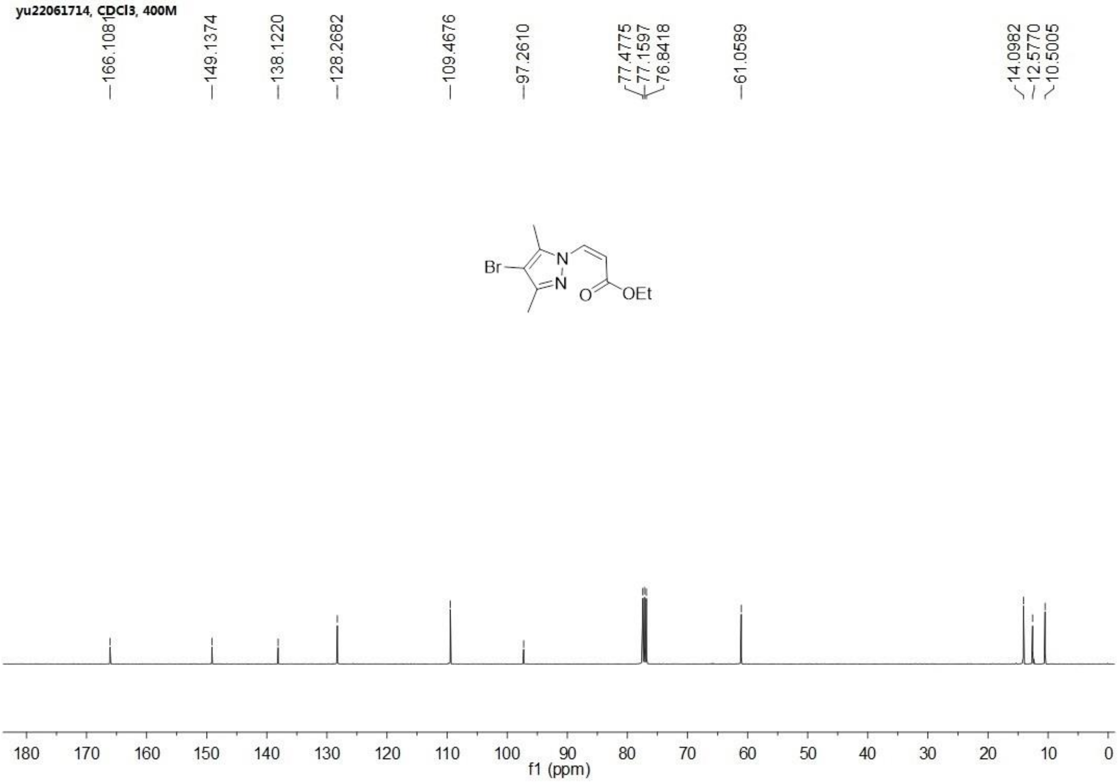

^{13}C NMR of **4p**

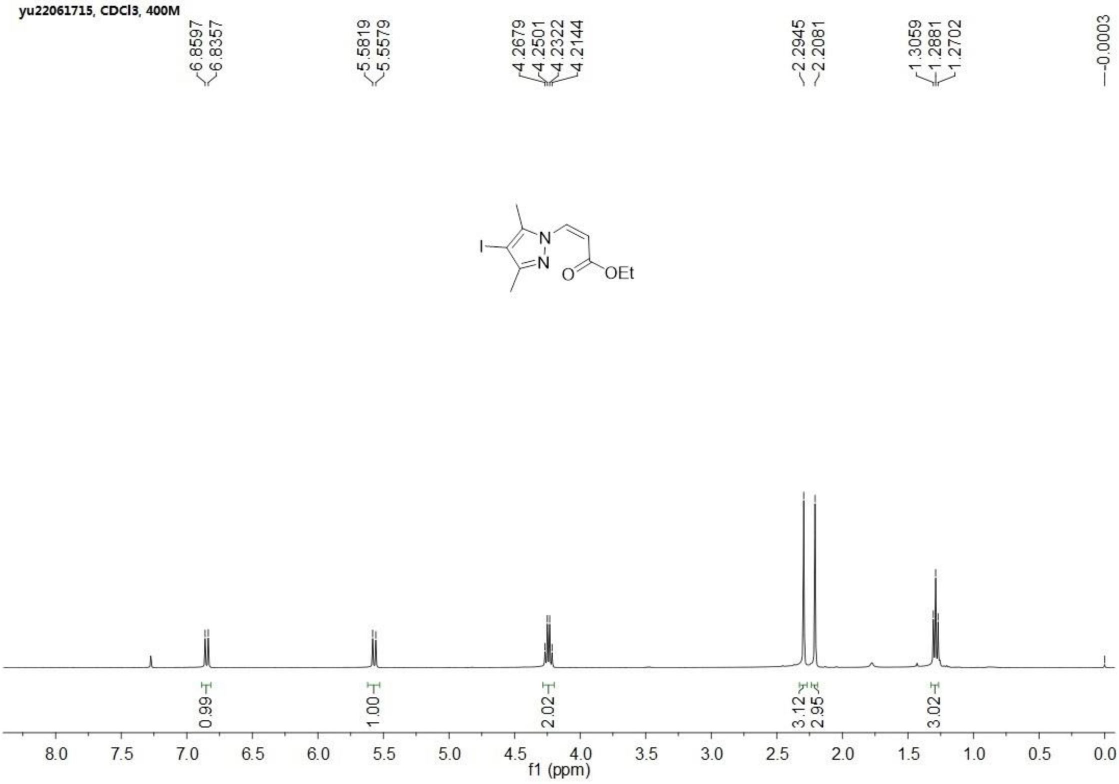

^{1}H NMR of **4q**

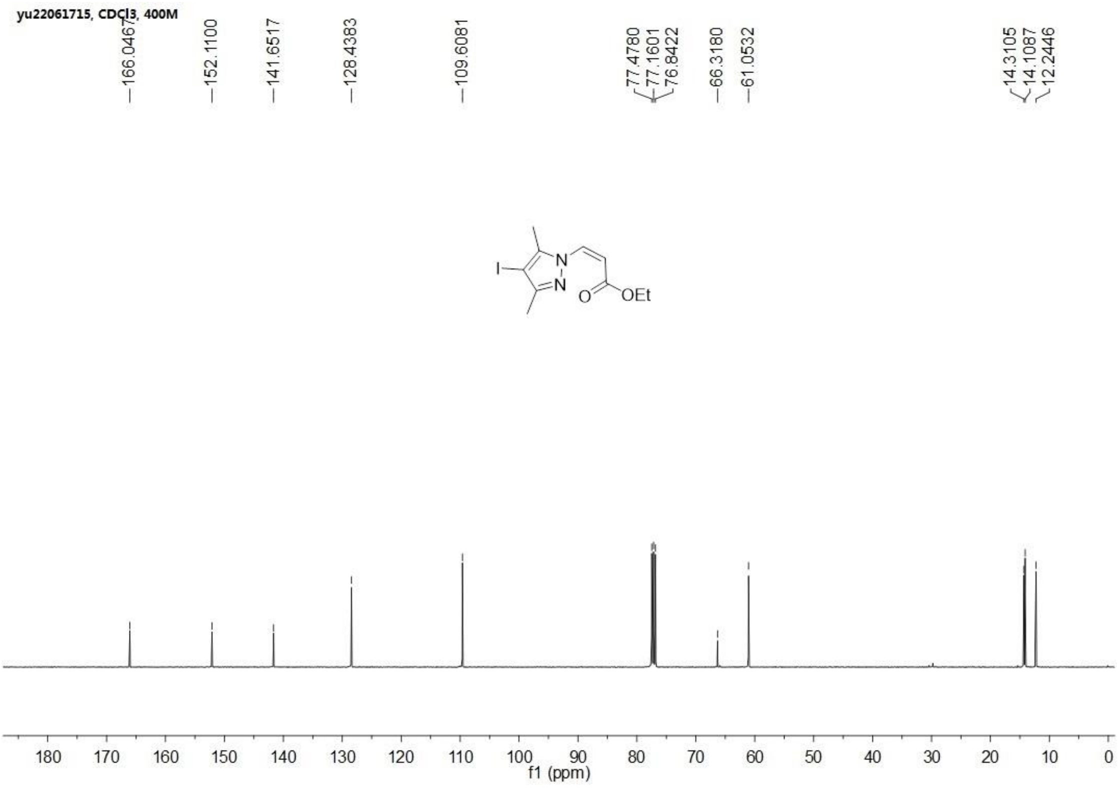

^{13}C NMR of **4q**

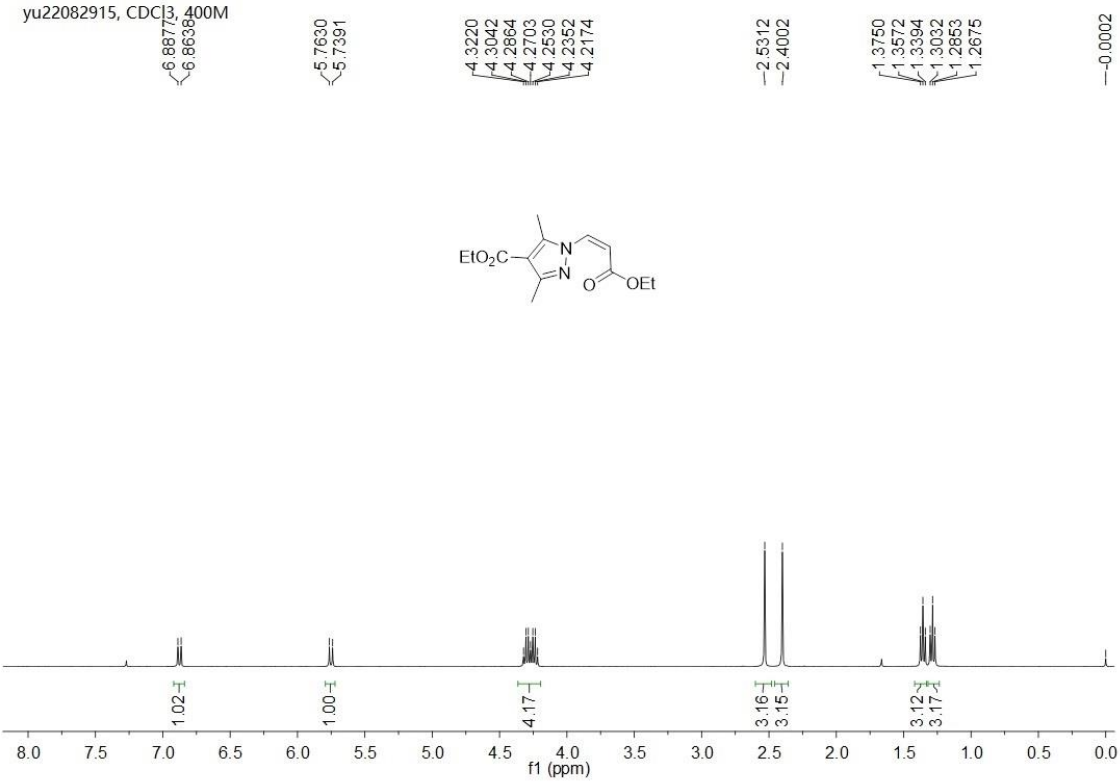

^{1}H NMR of **4r**

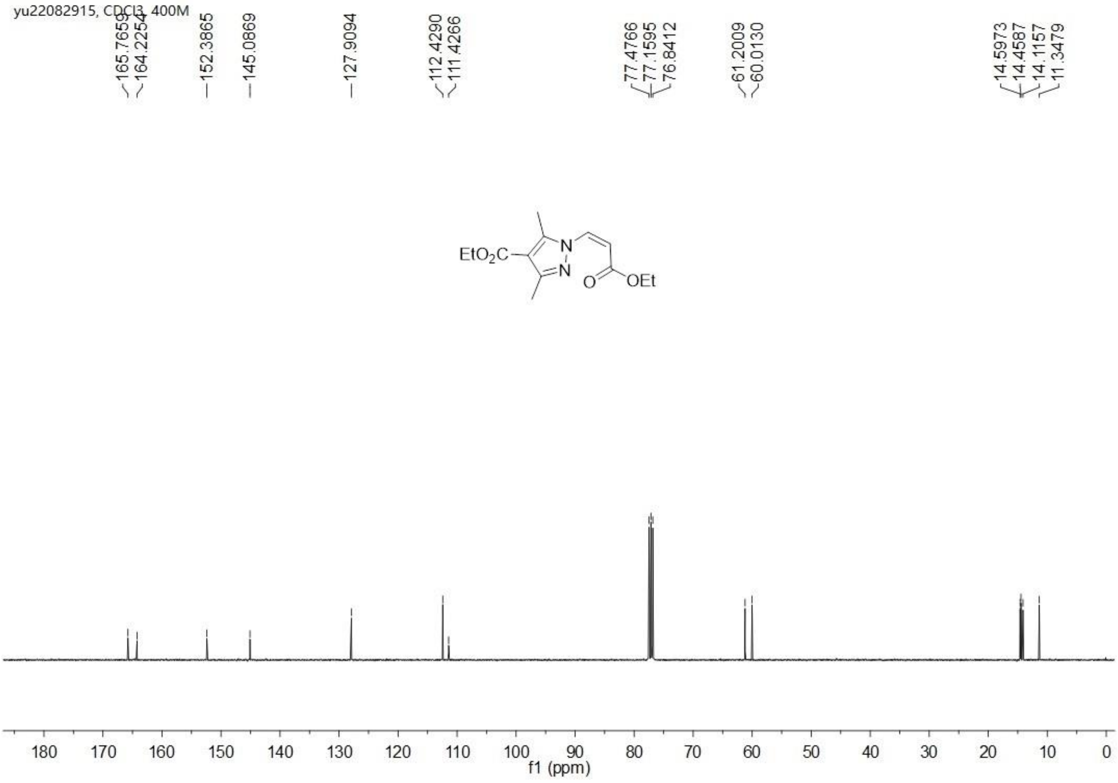

^{13}C NMR of **4r**

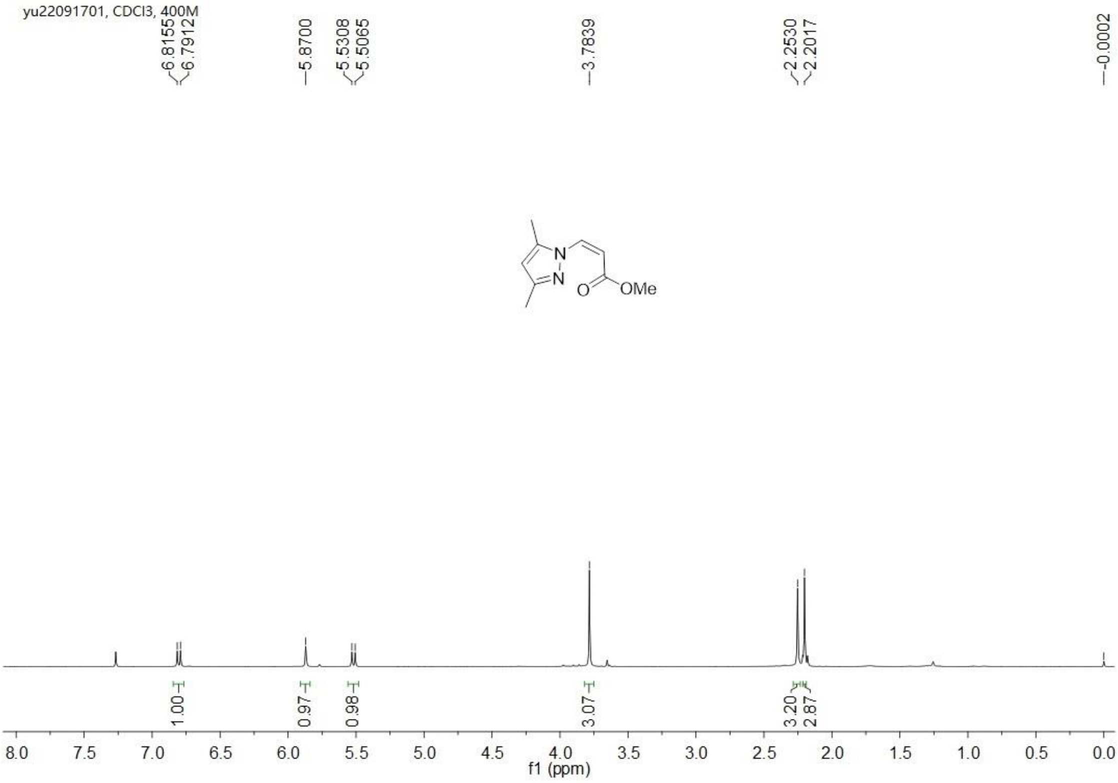

^{1}H NMR of **4s**

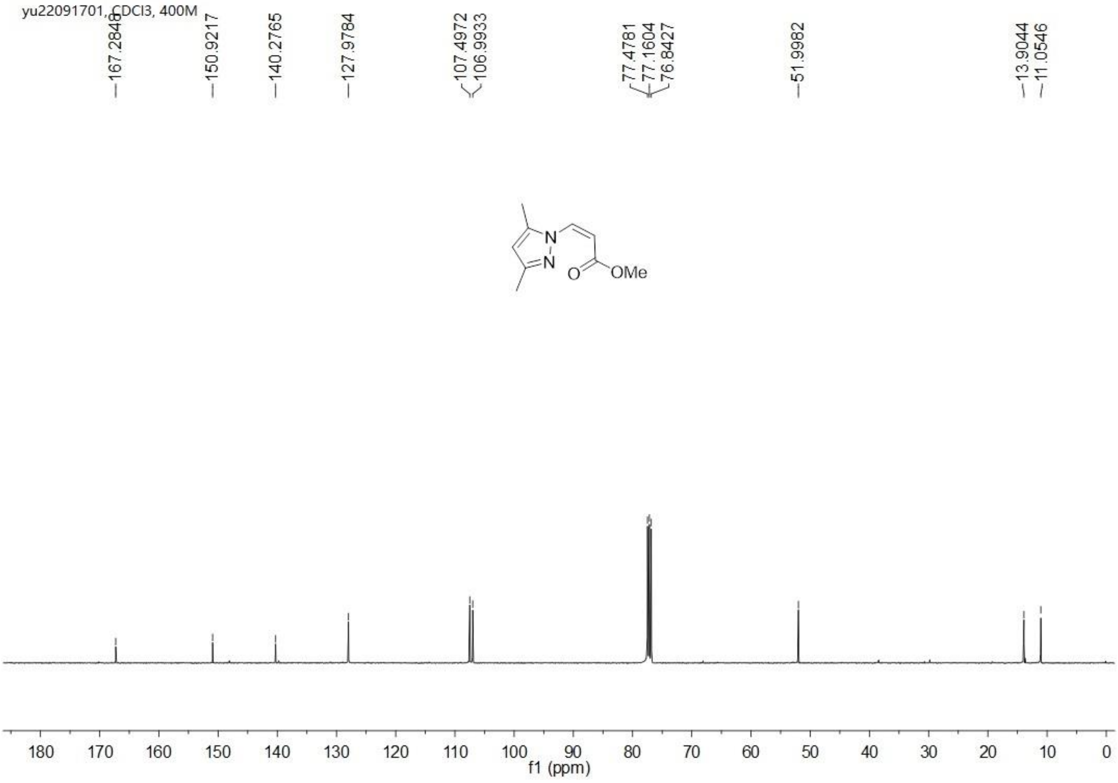

^{13}C NMR of **4s**

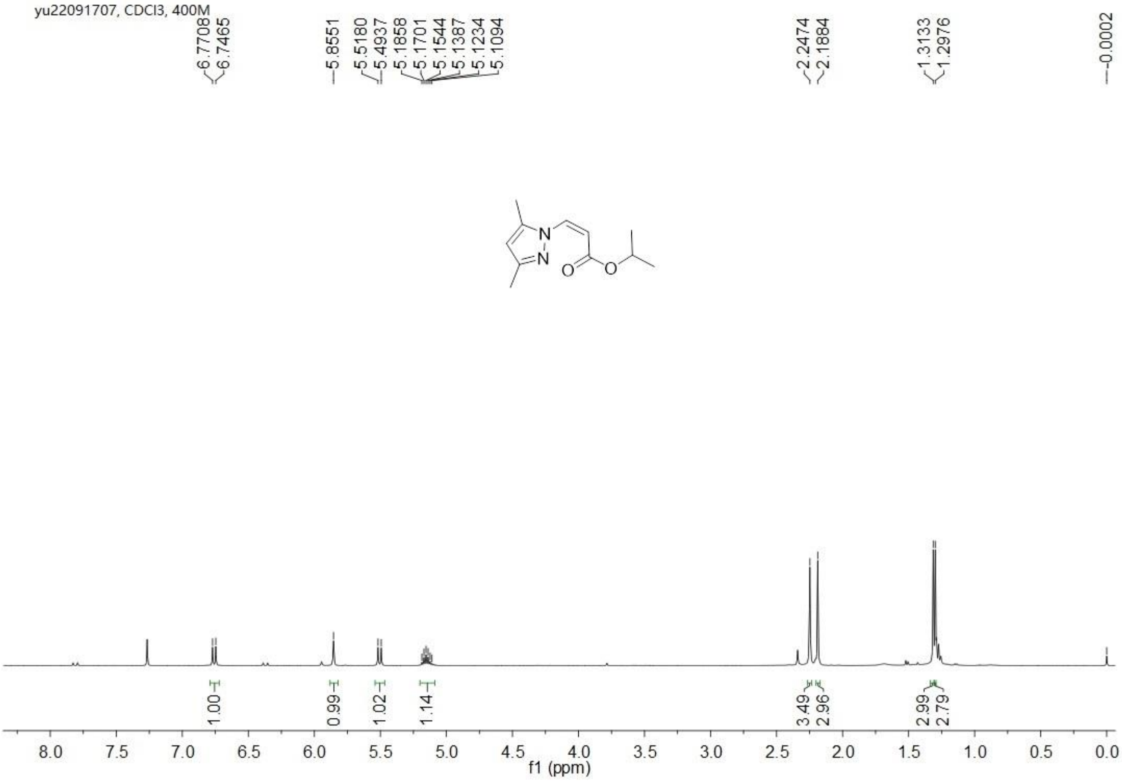

^{1}H NMR of **4t**

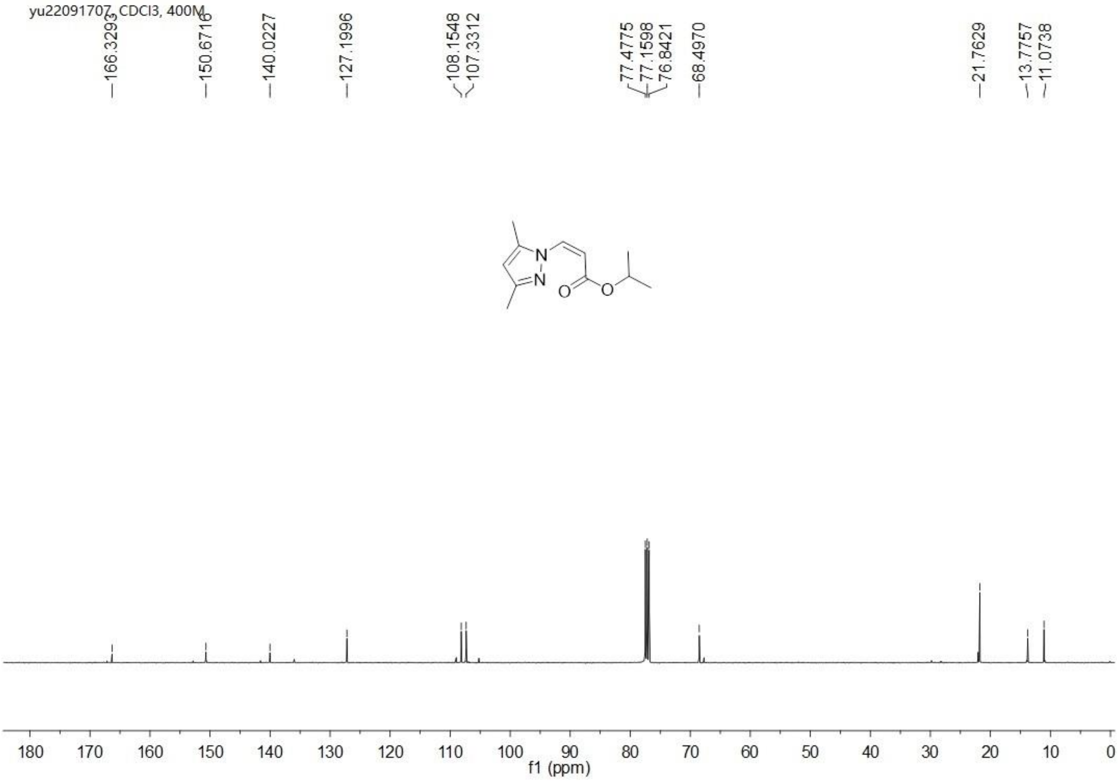

^{13}C NMR of **4t**

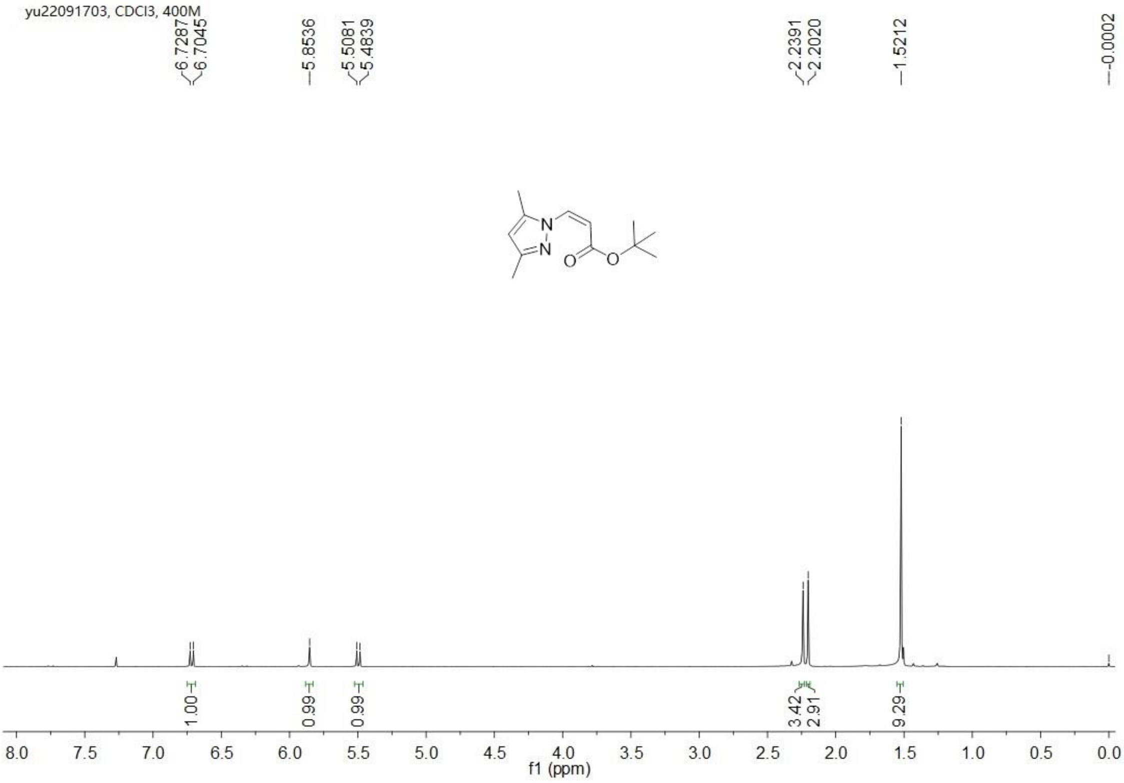

^{1}H NMR of **4u**

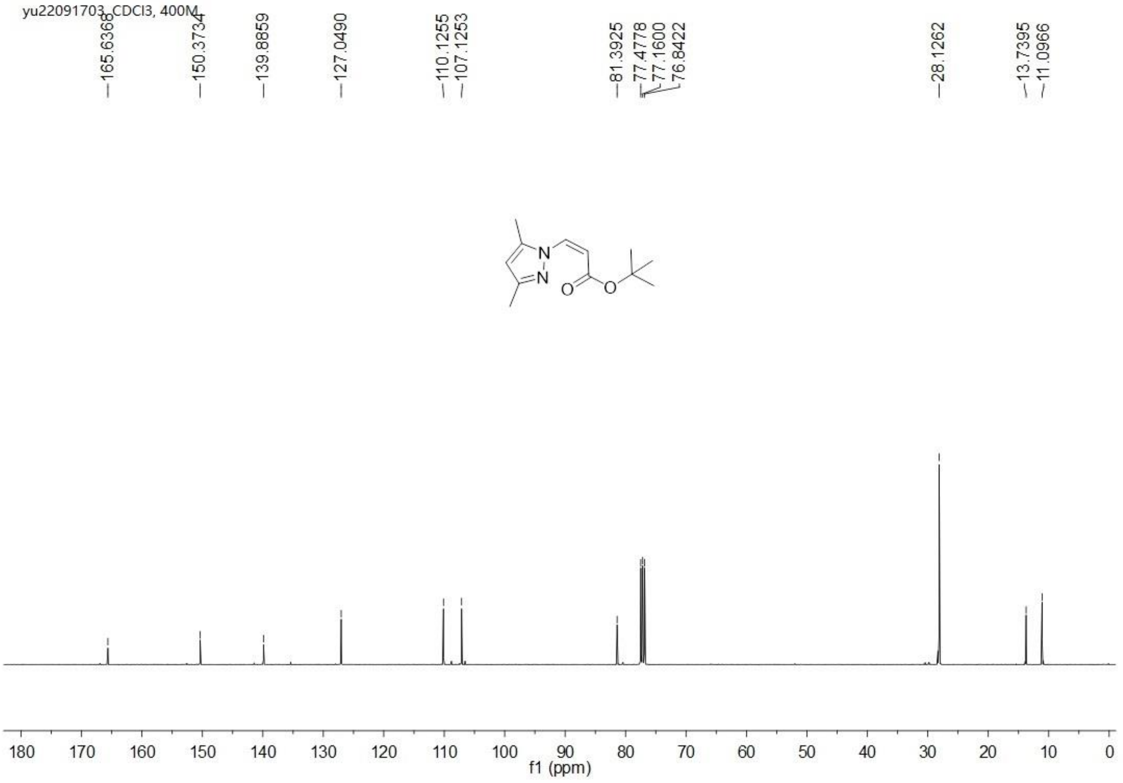

^{13}C NMR of **4u**

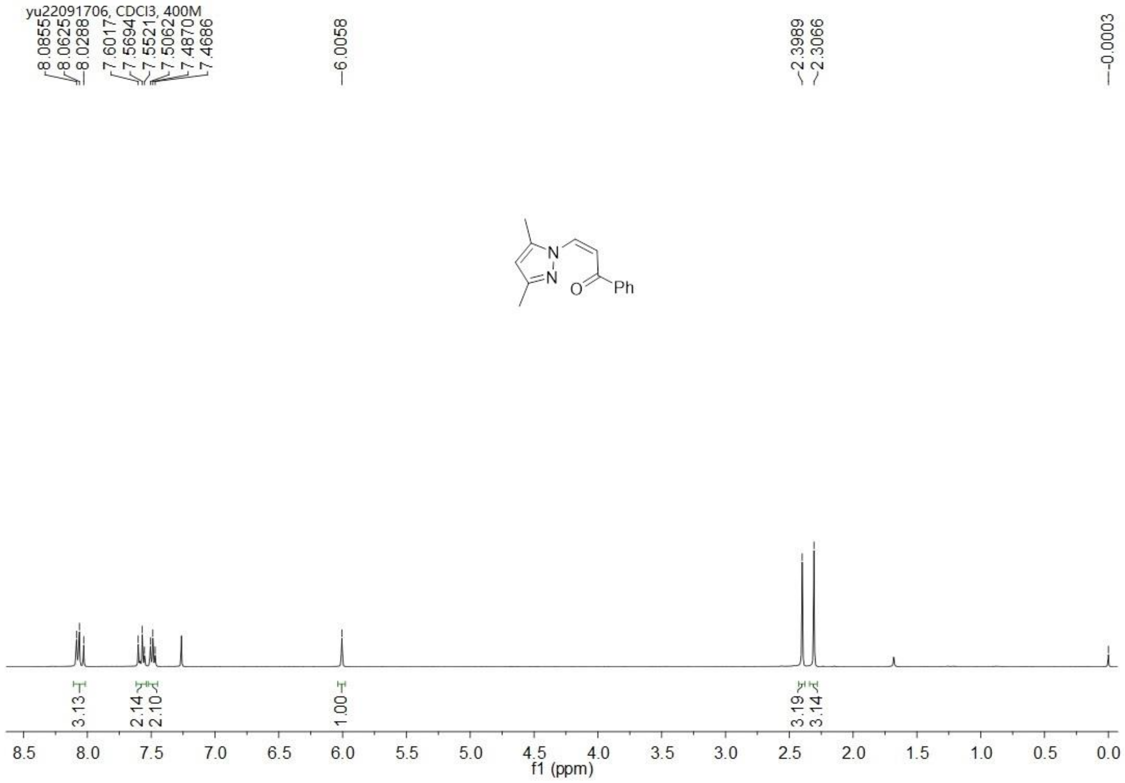

^{1}H NMR of **4v**

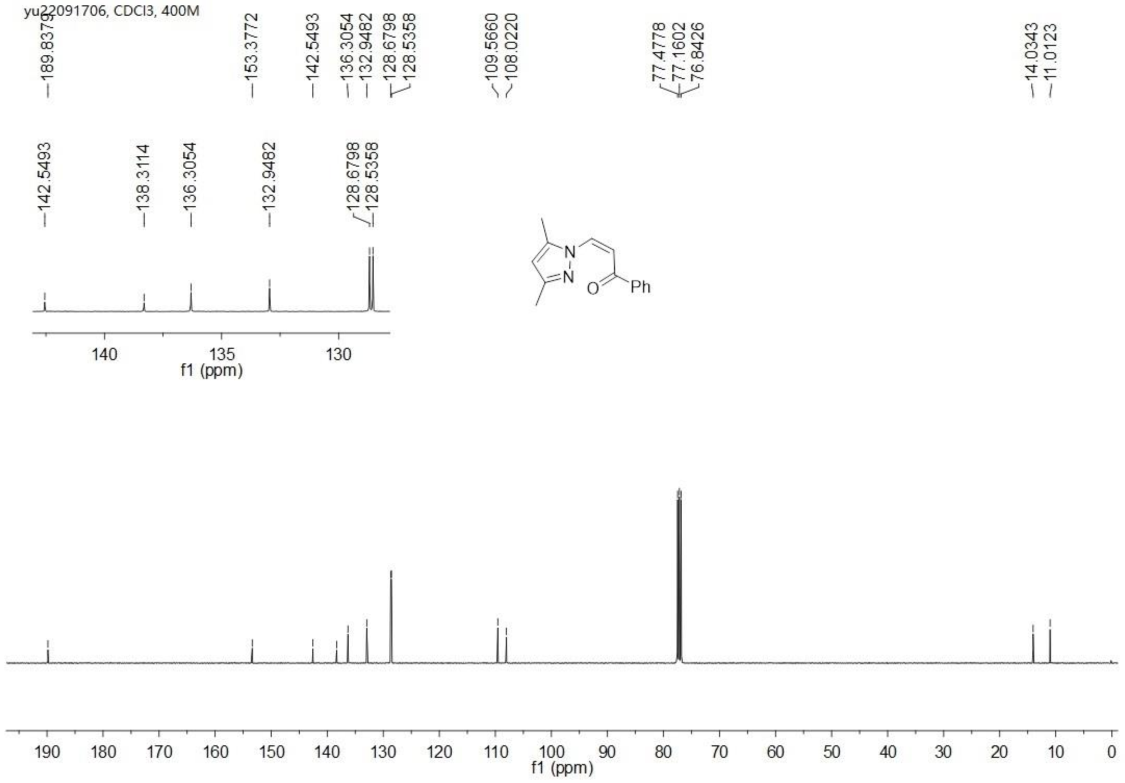

^{13}C NMR of **4v**

Copies of ^{1}H NMR and ^{13}C NMR spectra of compounds 5

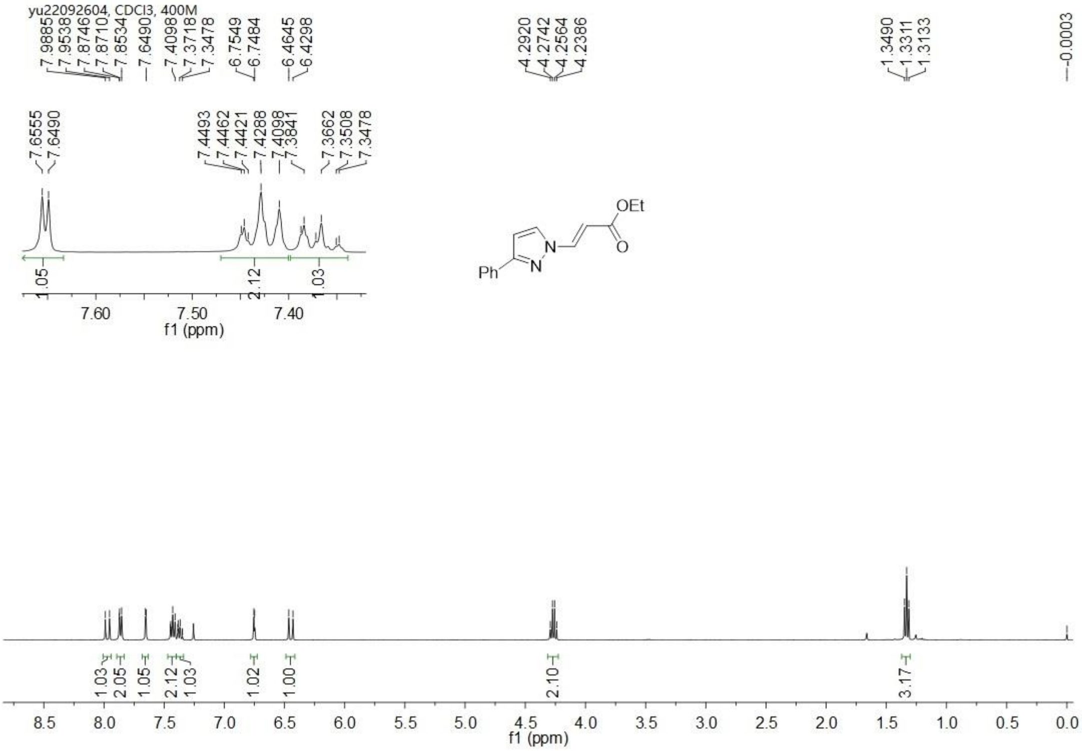

^{1}H NMR of **5a**

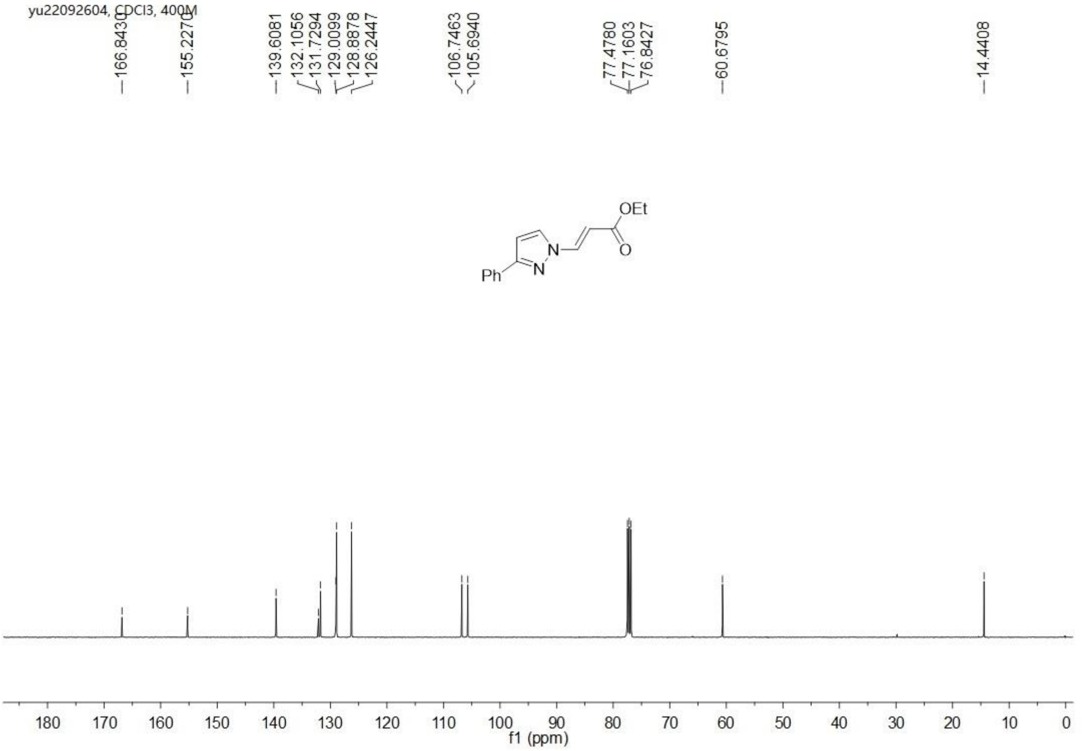

^{13}C NMR of **5a**

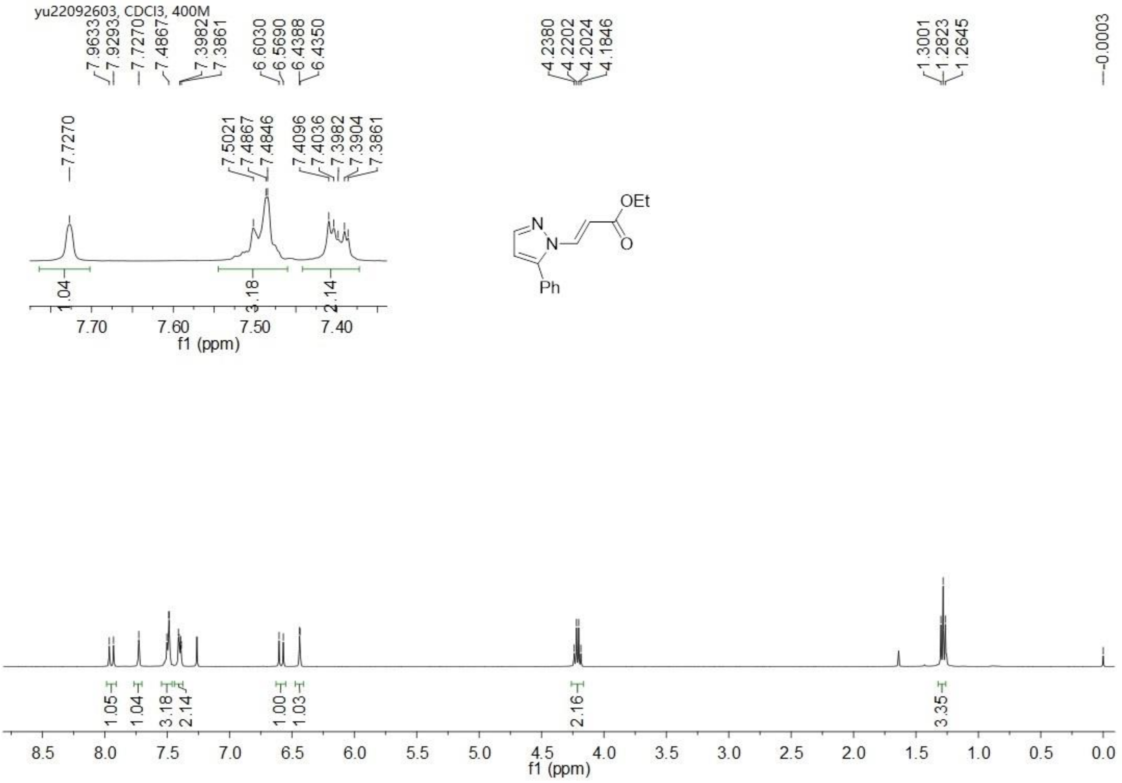

^{1}H NMR of **5a’**

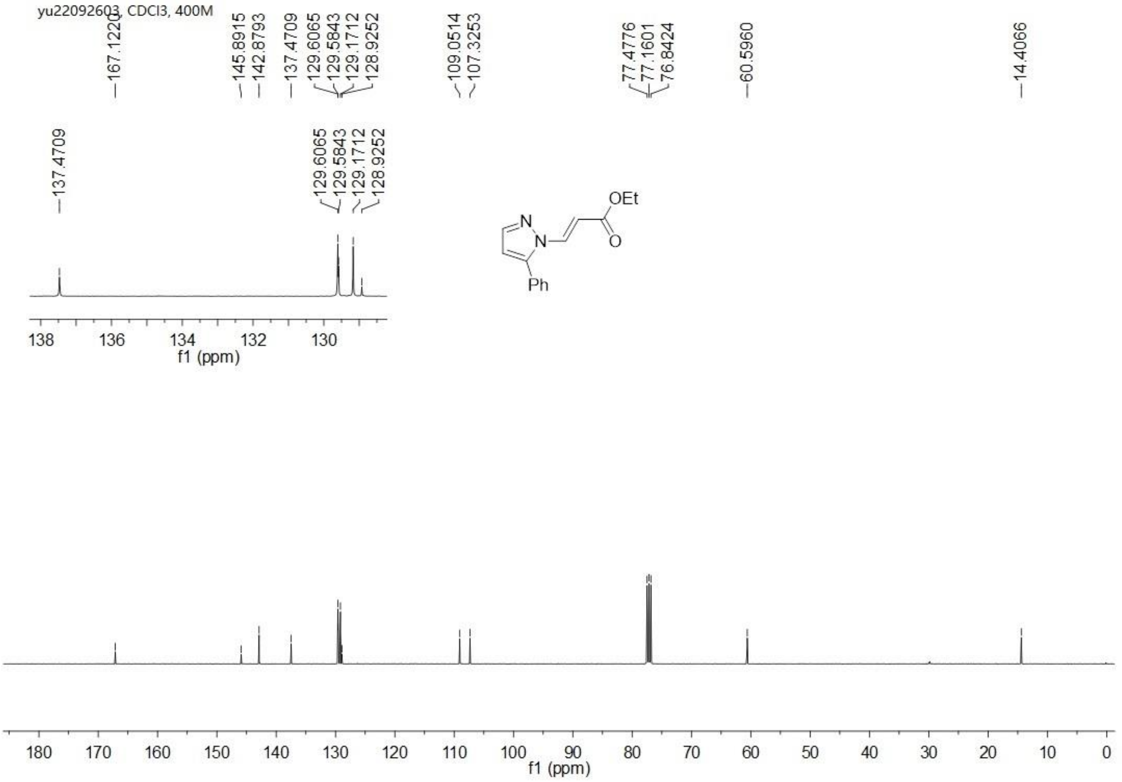

^{13}C NMR of **5a’**

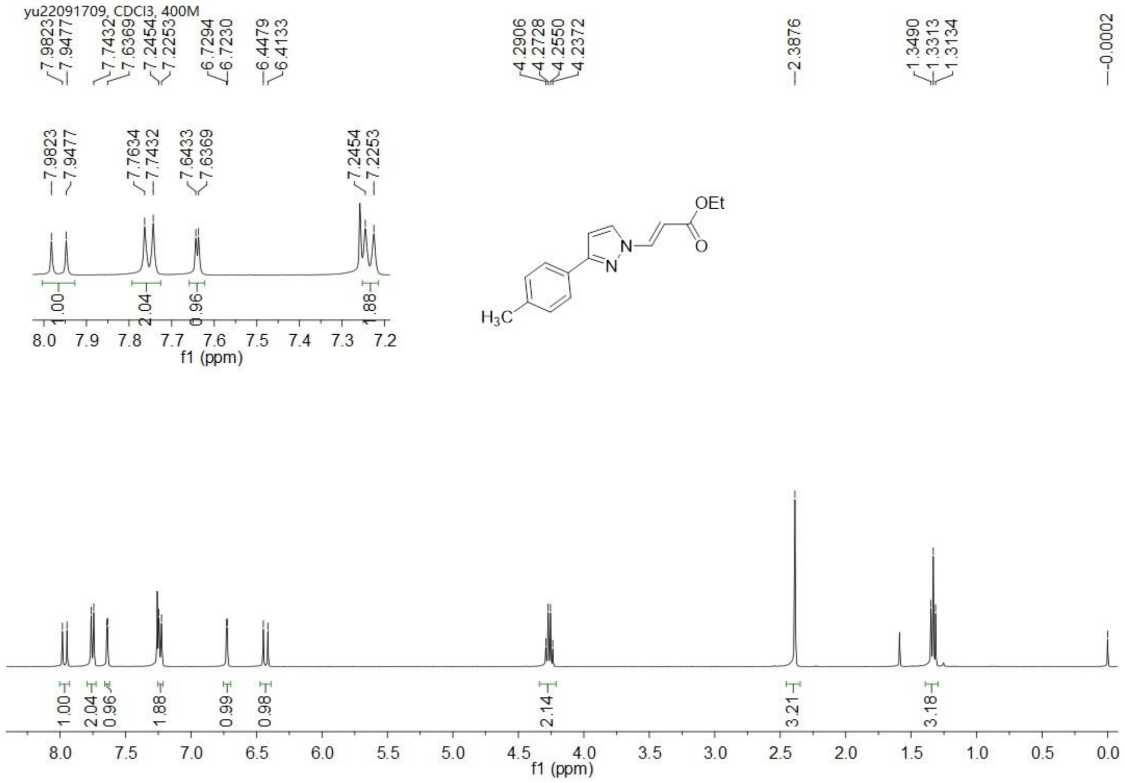

^{1}H NMR of **5b**

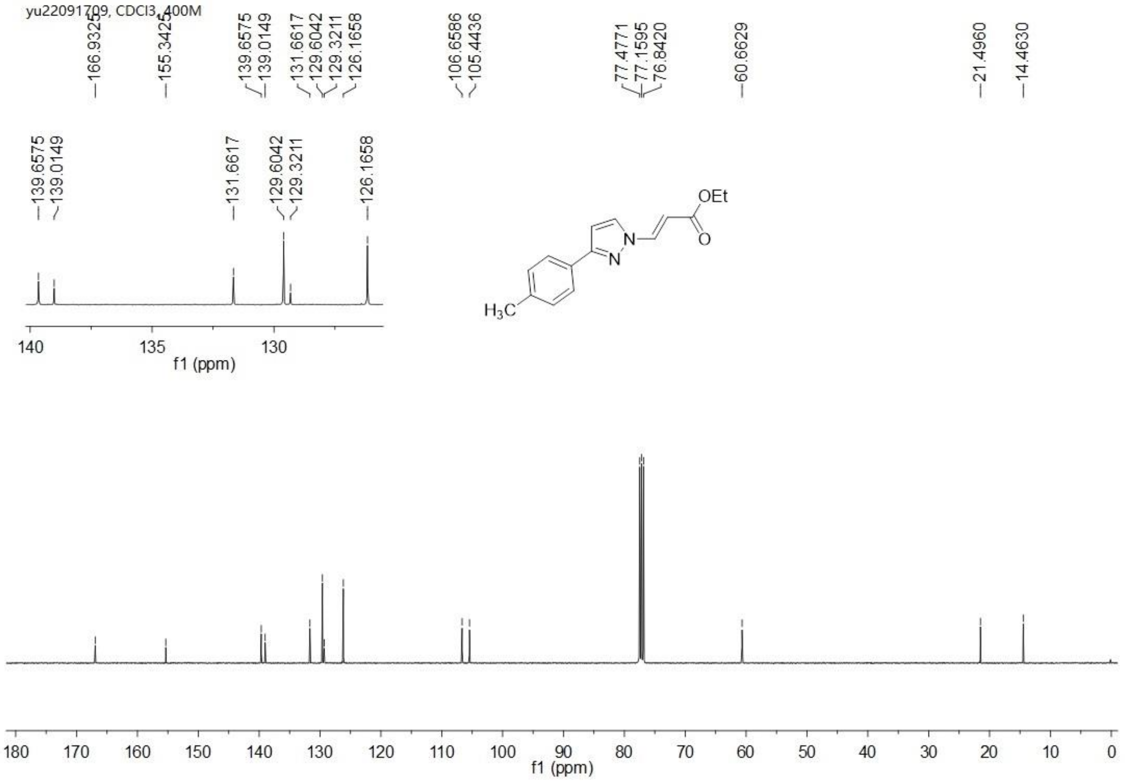

^{13}C NMR of **5b**

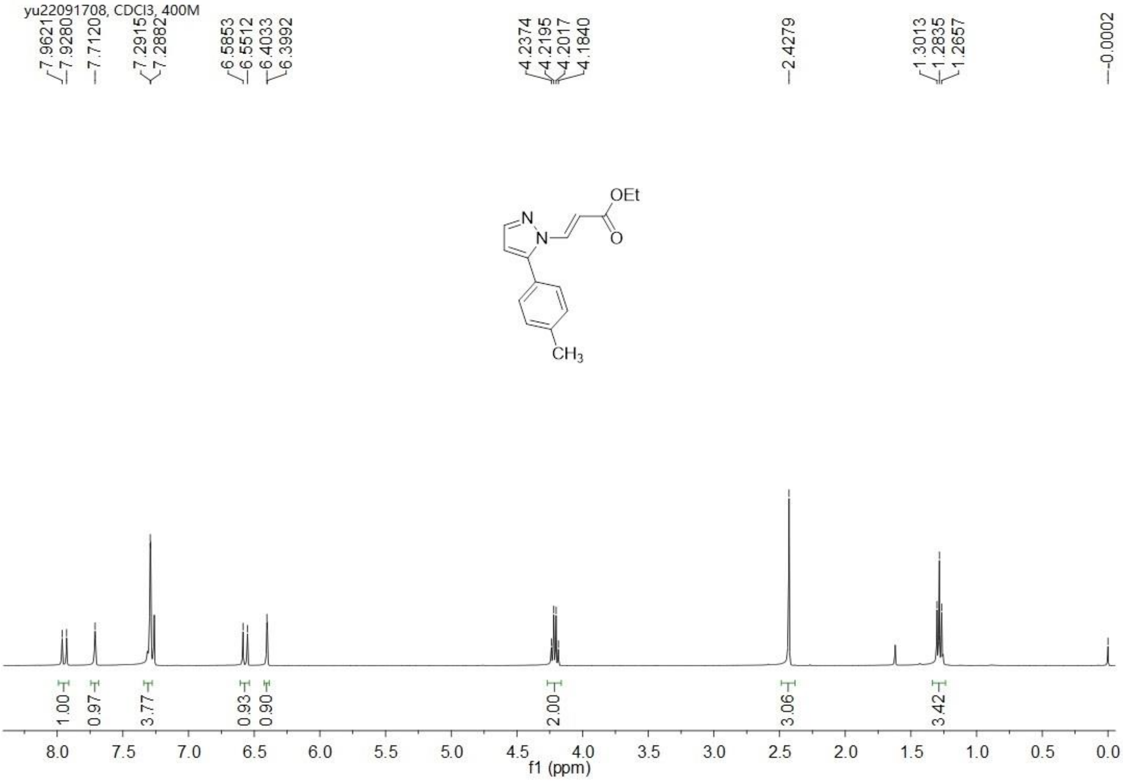

^{1}H NMR of **5b’**

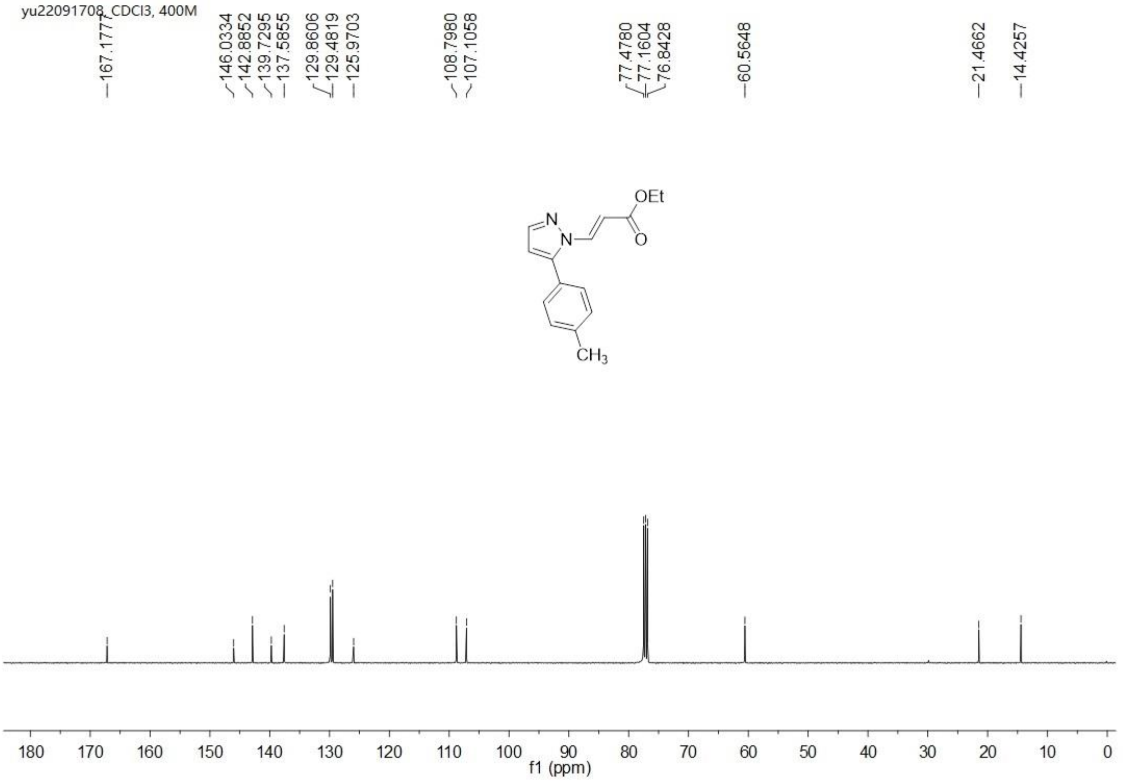

^{13}C NMR of **5b’**

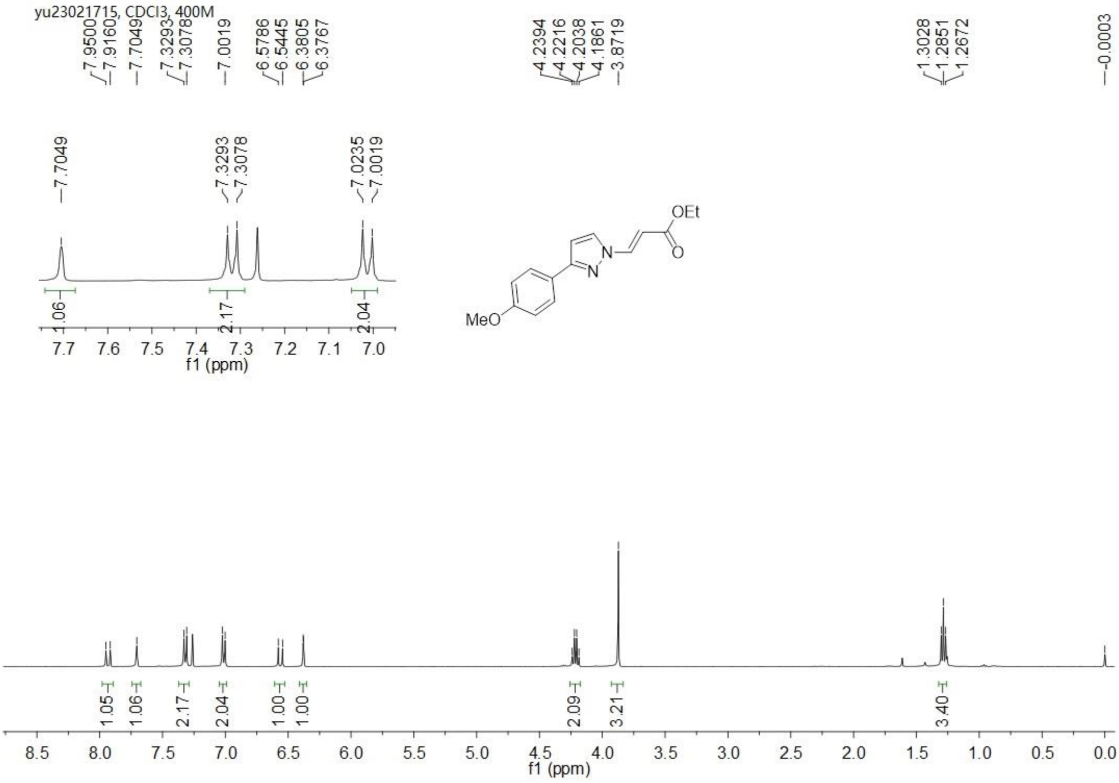

^{1}H NMR of **5c**

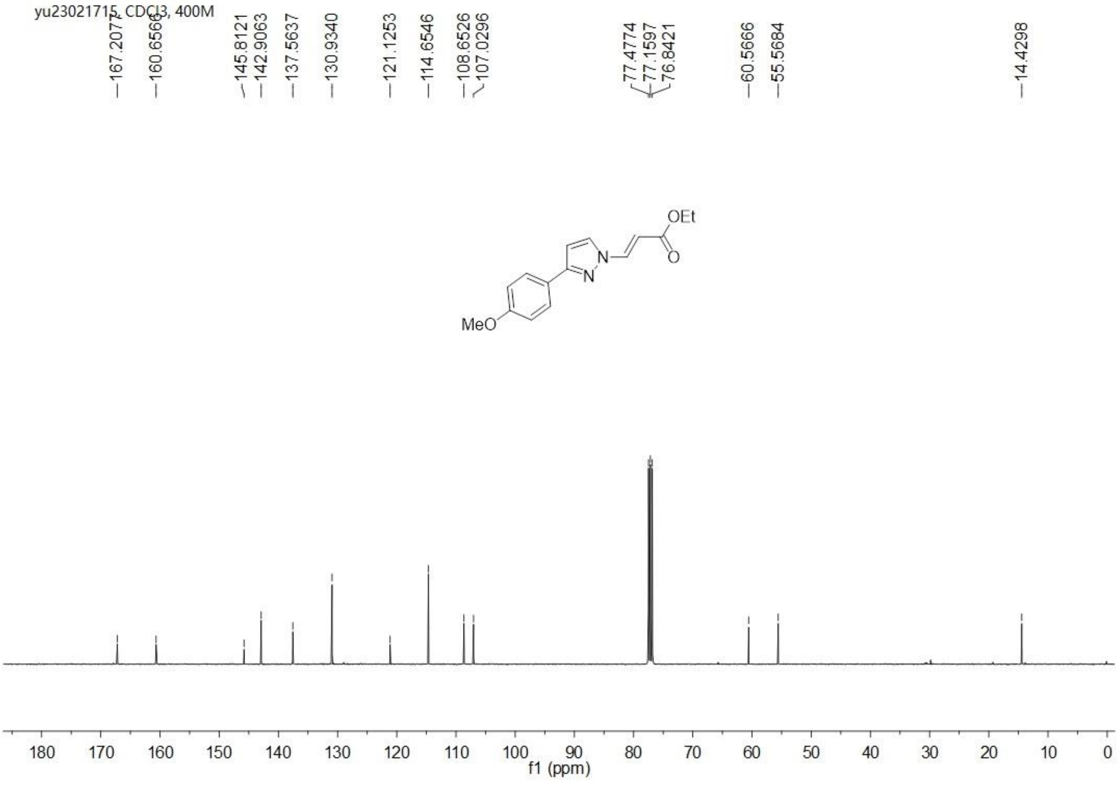

^{13}C NMR of **5c**

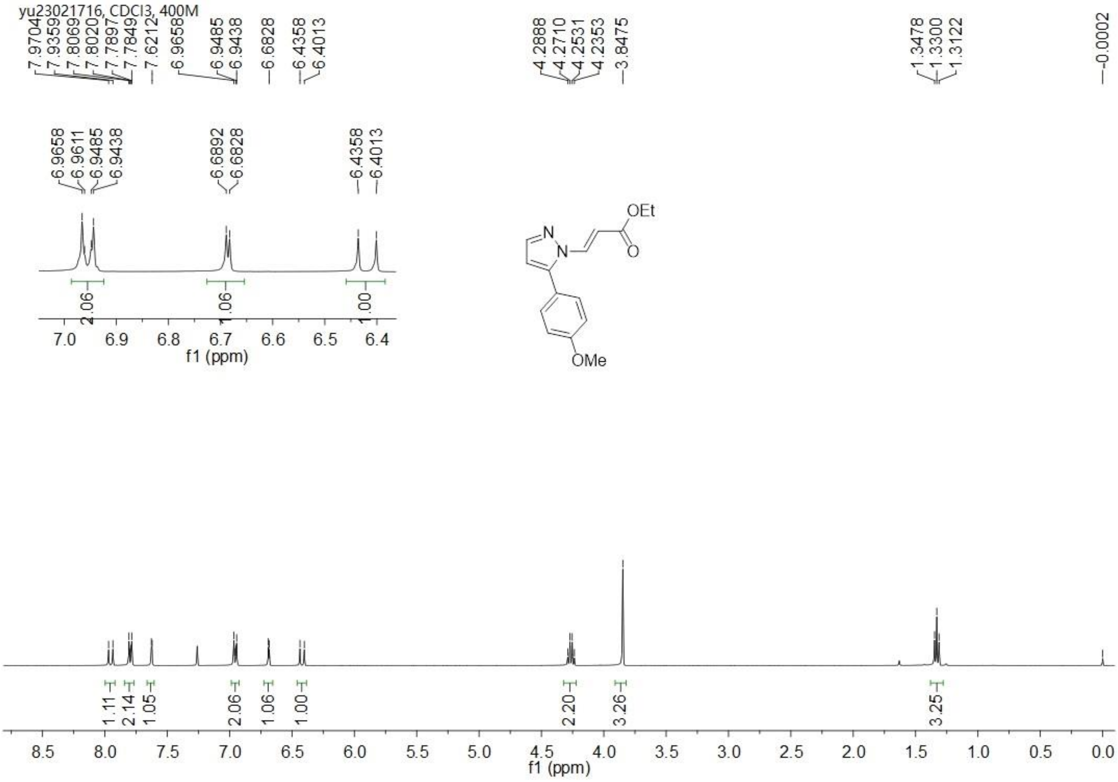

^{1}H NMR of **5c’**

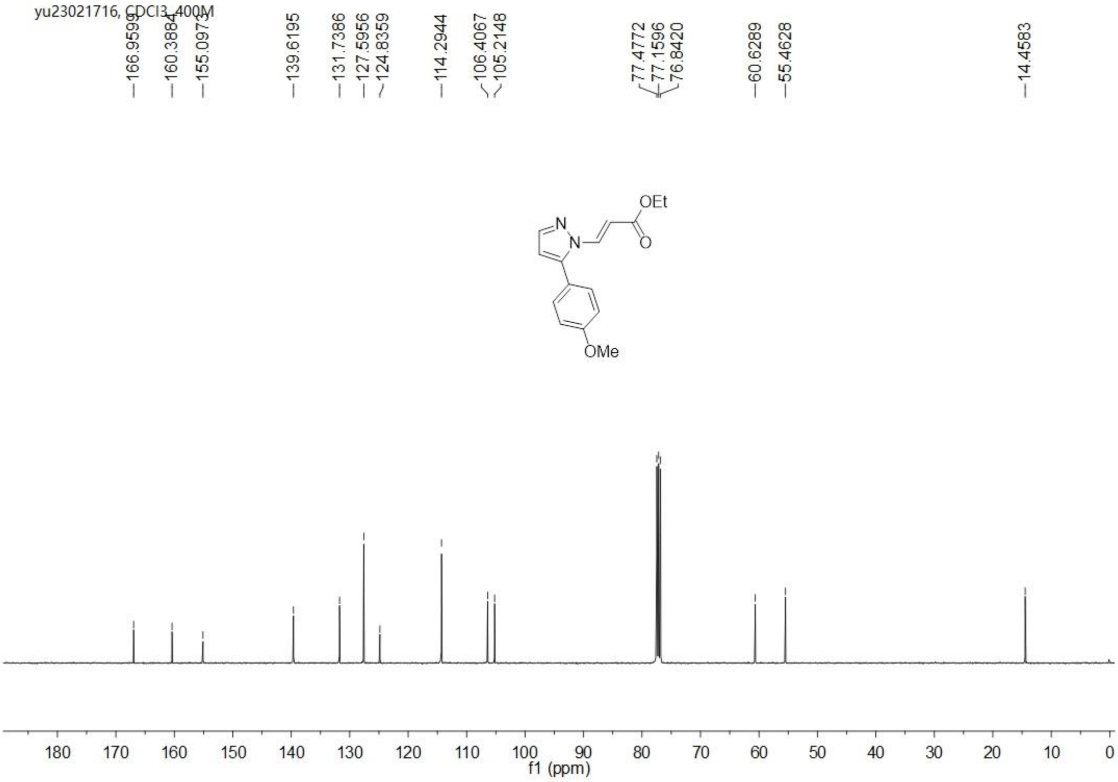

^{13}C NMR of **5c’**

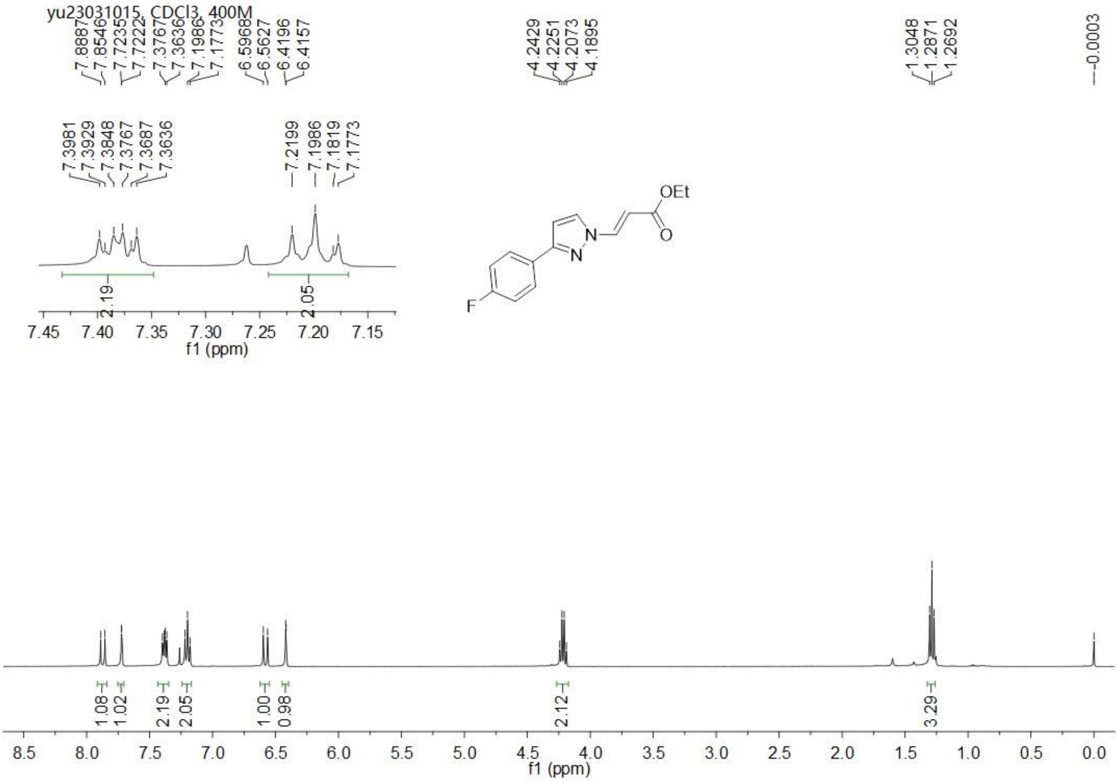

^{1}H NMR of **5d**

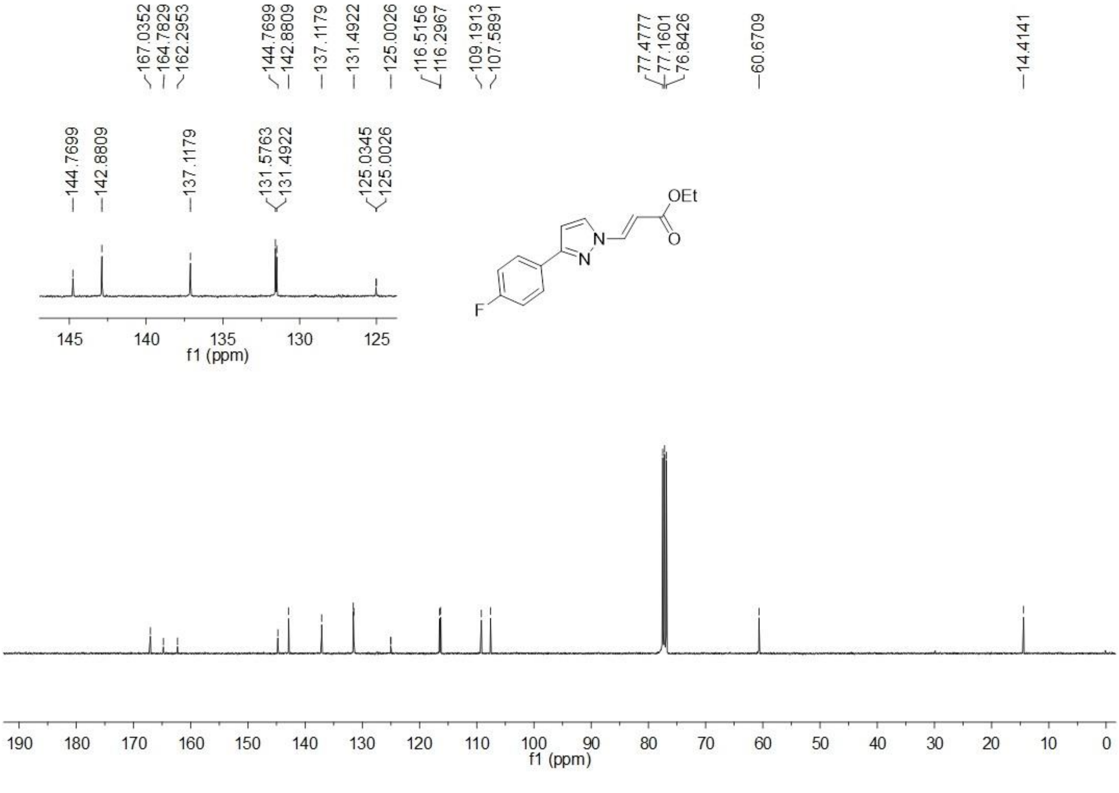

^{13}C NMR of **5d**

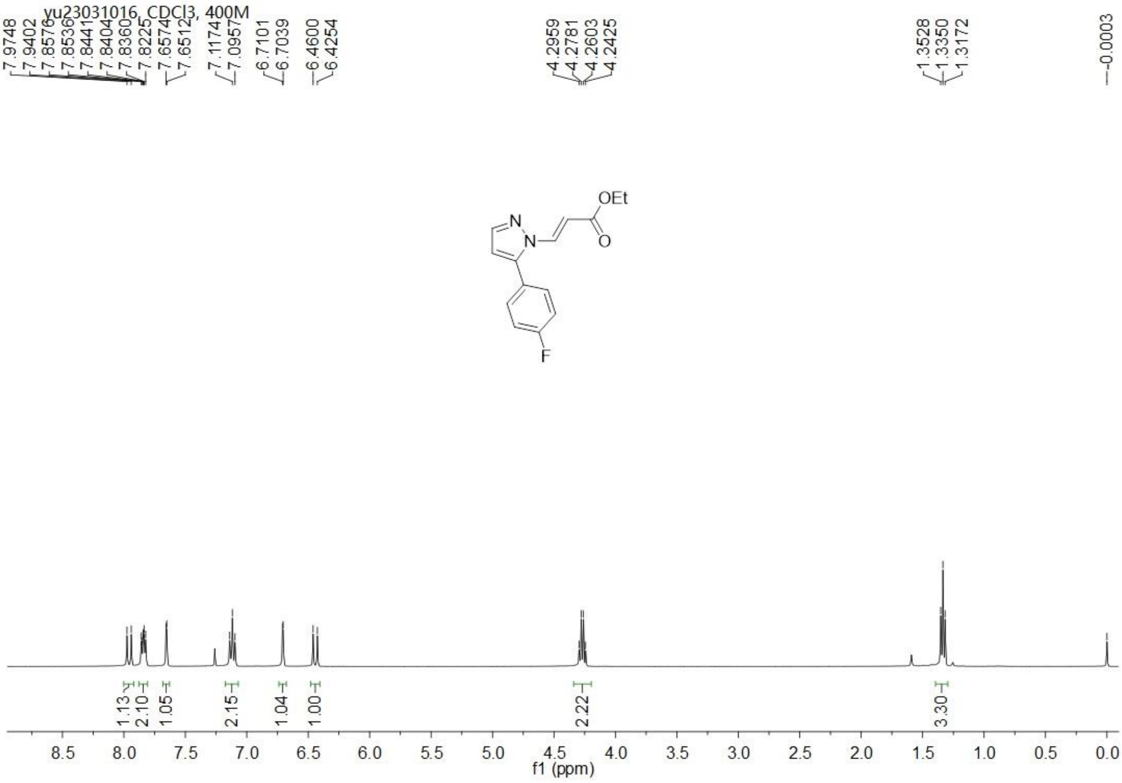

^{1}H NMR of **5d’**

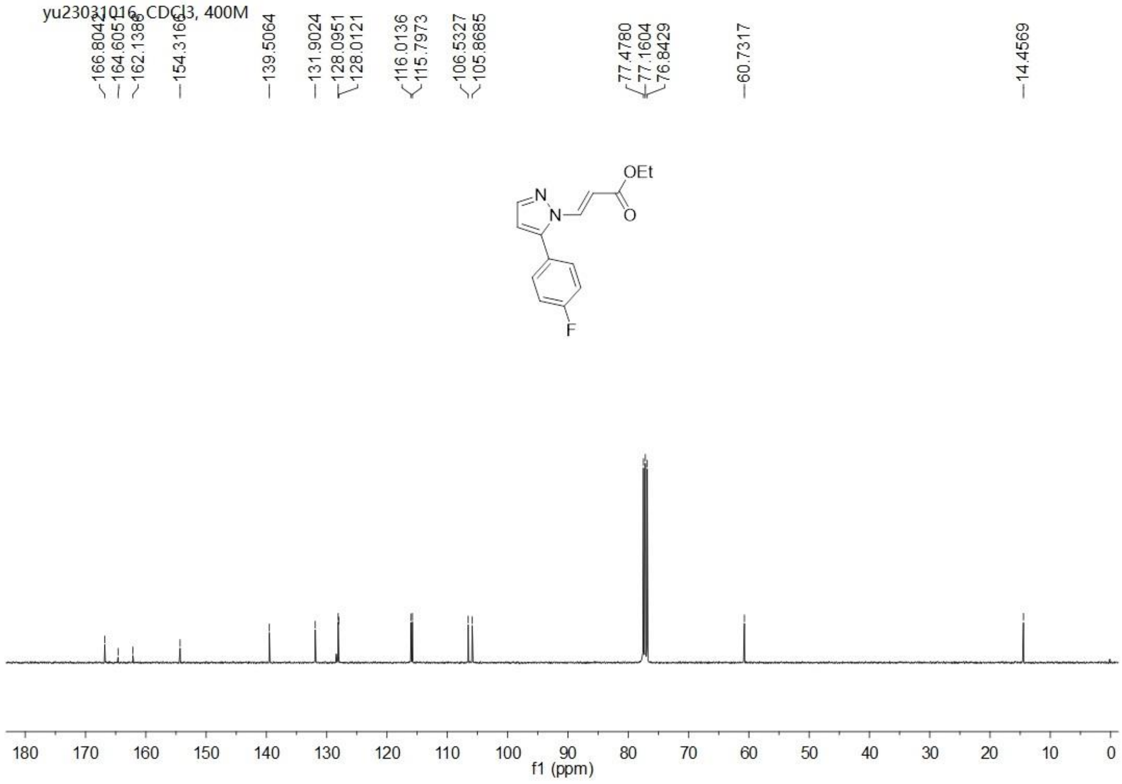

^{13}C NMR of **5d’**

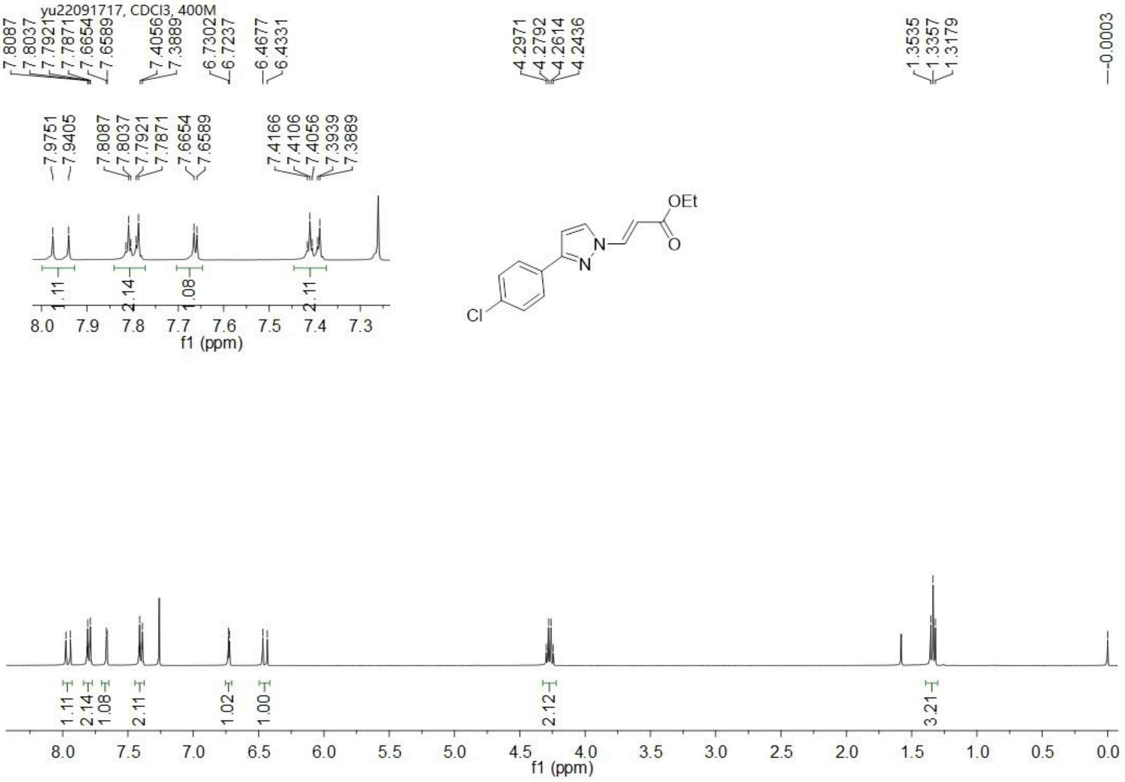

^{1}H NMR of **5e**

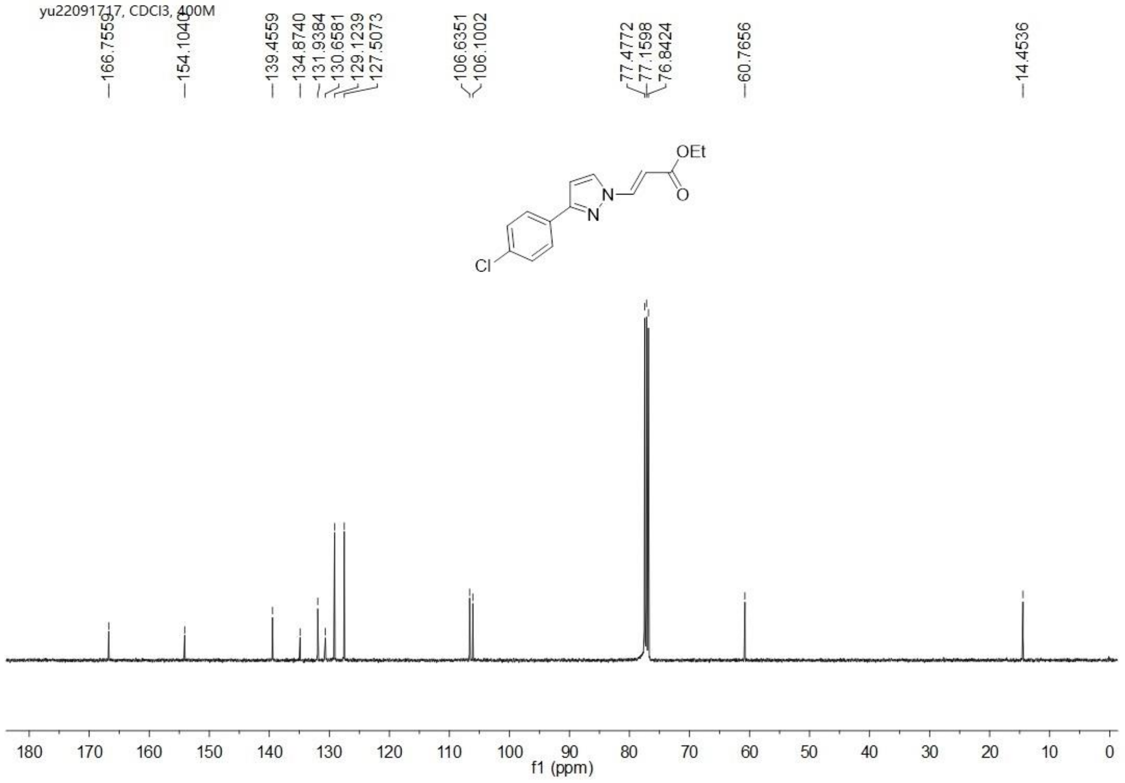

^{13}C NMR of **5e**

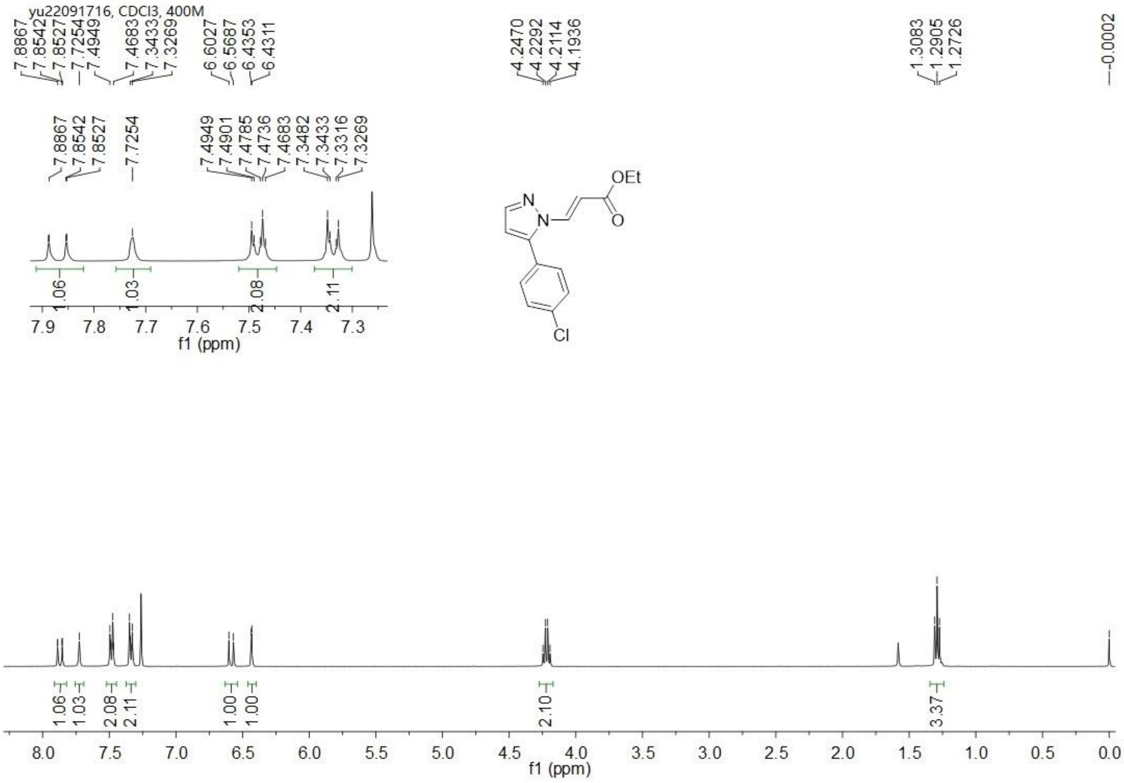

^{1}H NMR of **5e’**

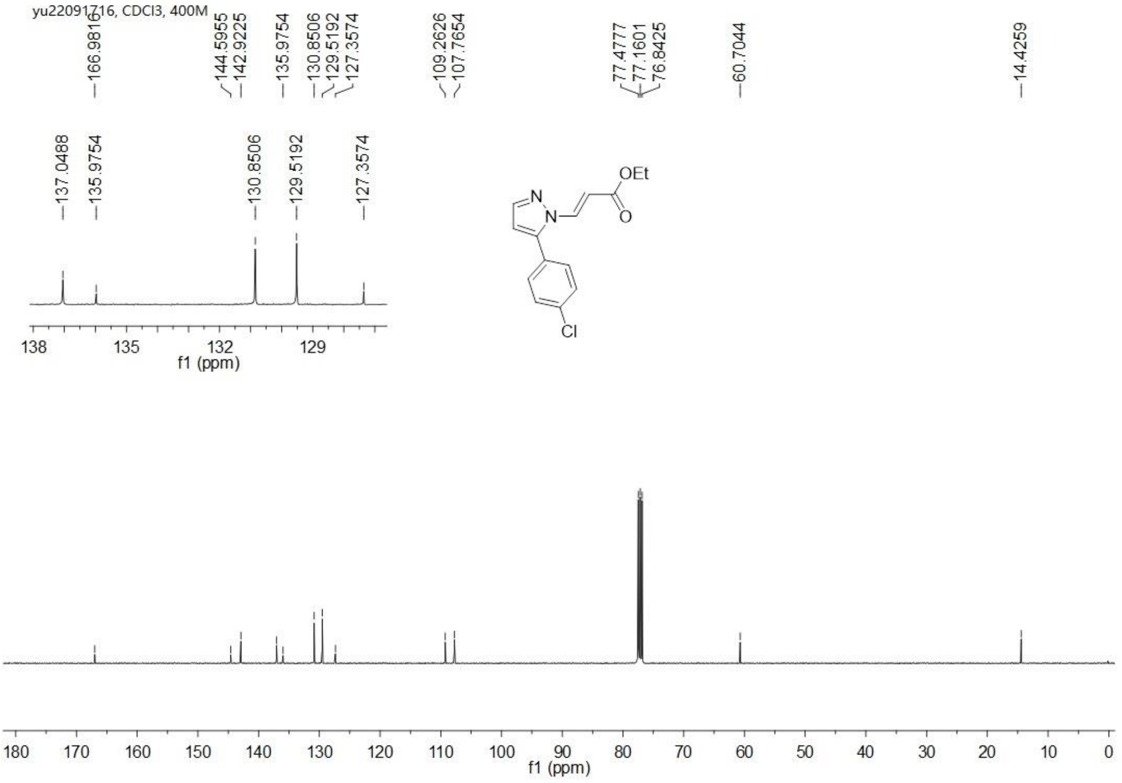

^{13}C NMR of **5e’**

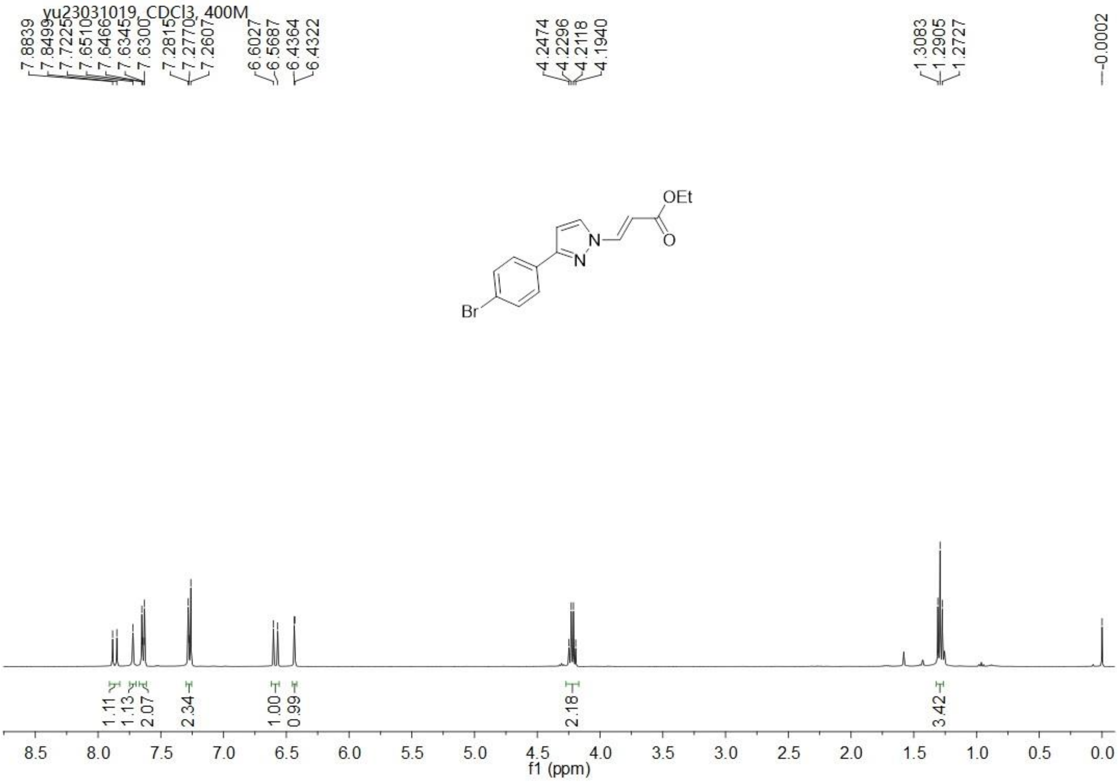

^{1}H NMR of **5f**

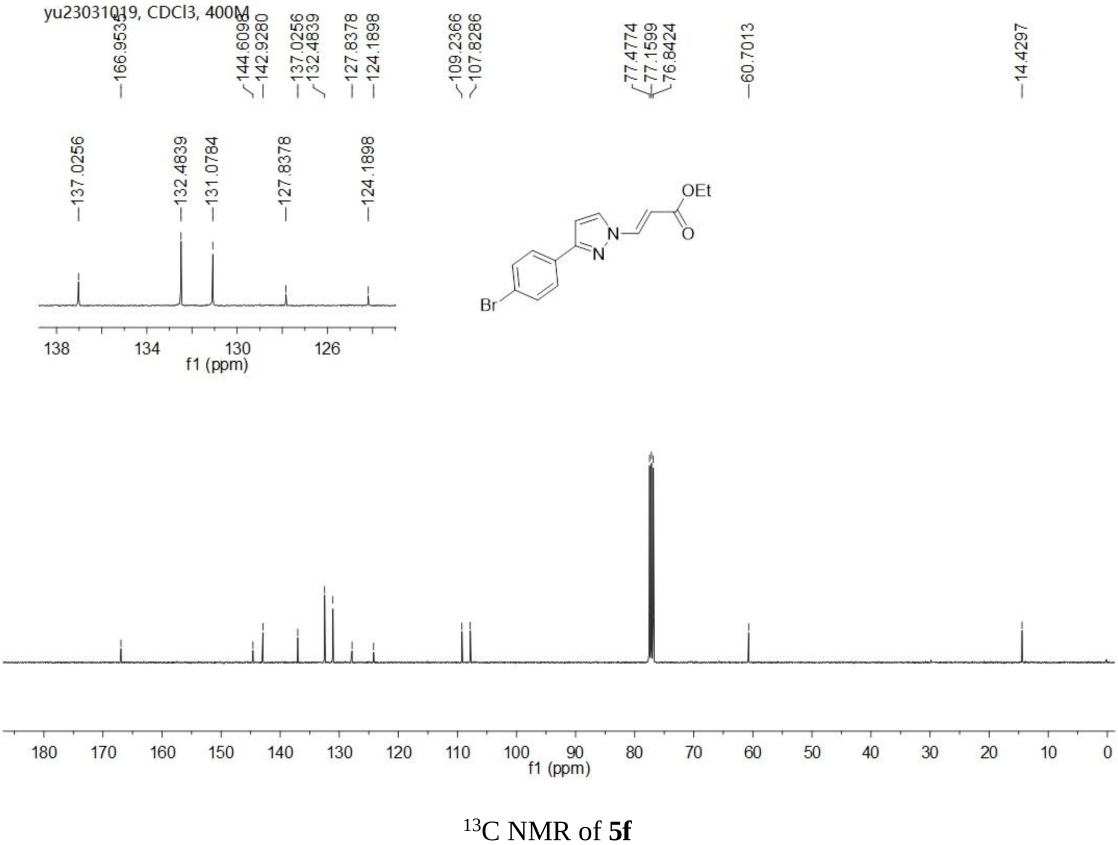

^{13}C NMR of **5f**

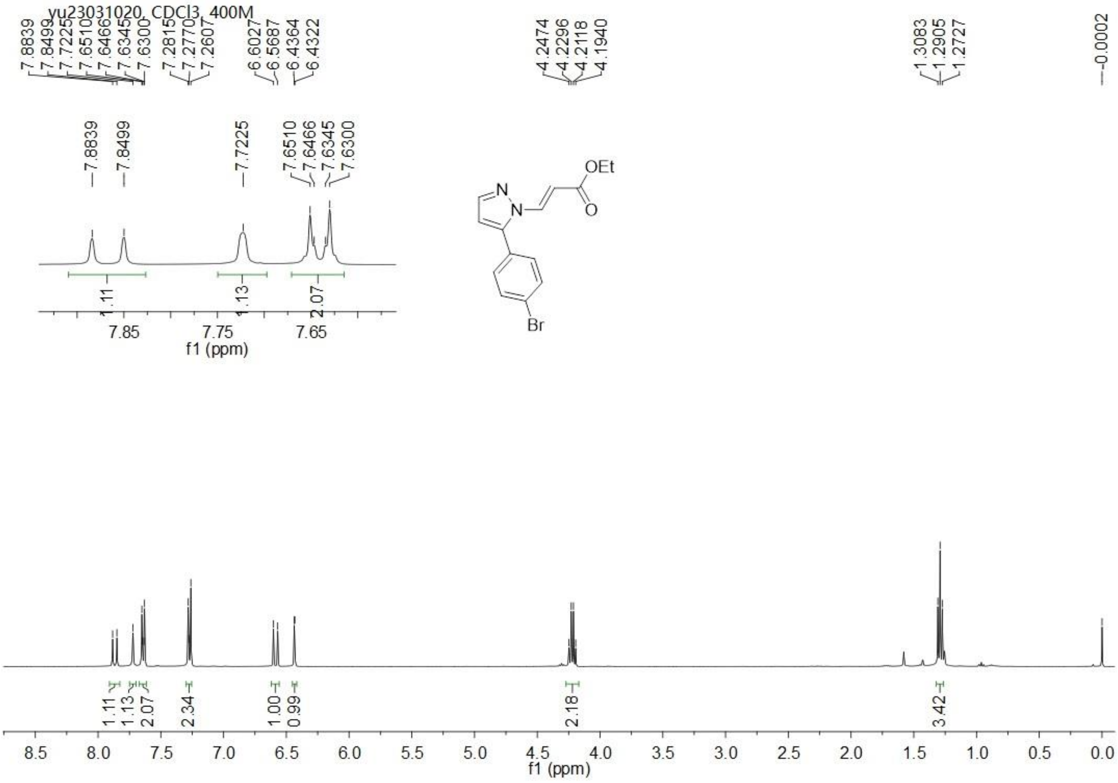

^{1}H NMR of **5f’**

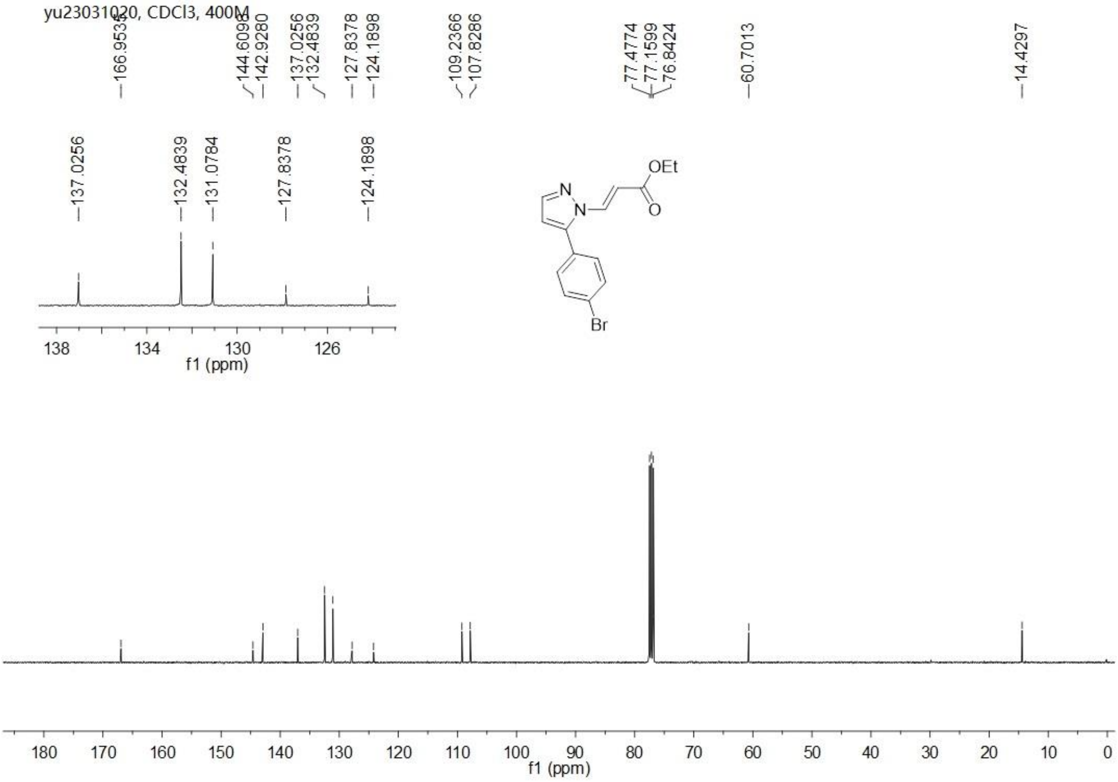

^{13}C NMR of **5f’**

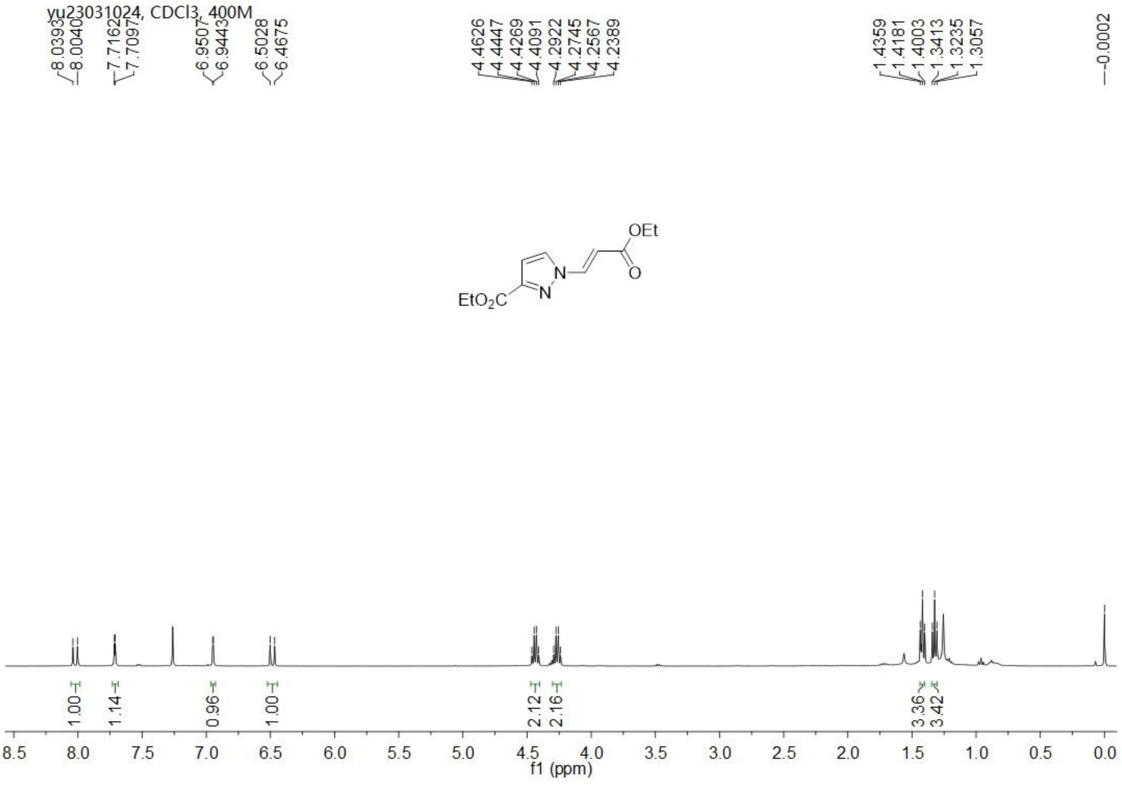

^{1}H NMR of **5g**

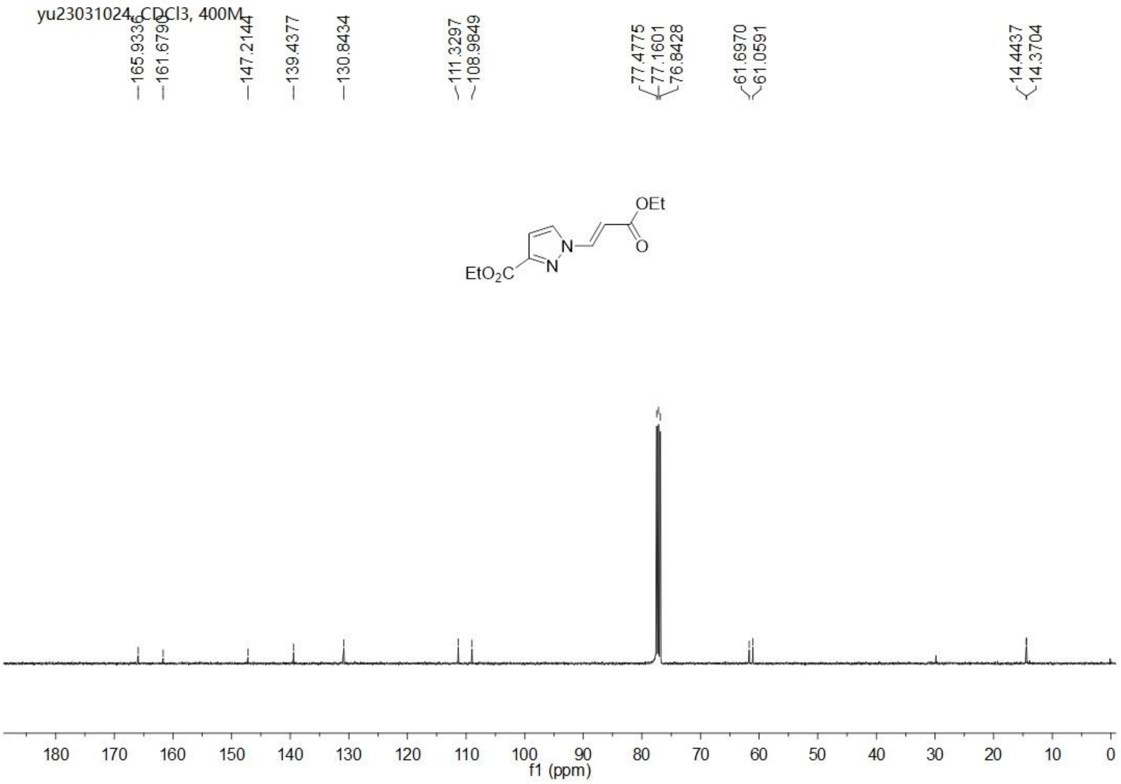

^{13}C NMR of **5g**

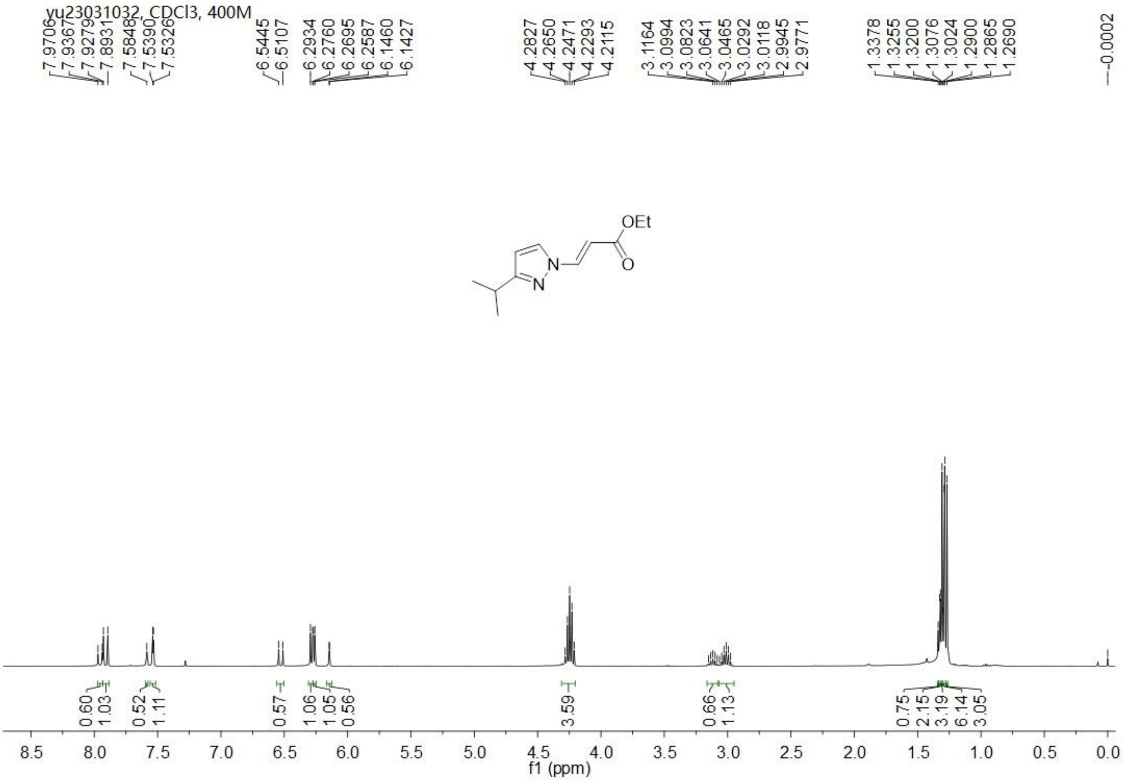

^{1}H NMR of **5h** and **5h’**

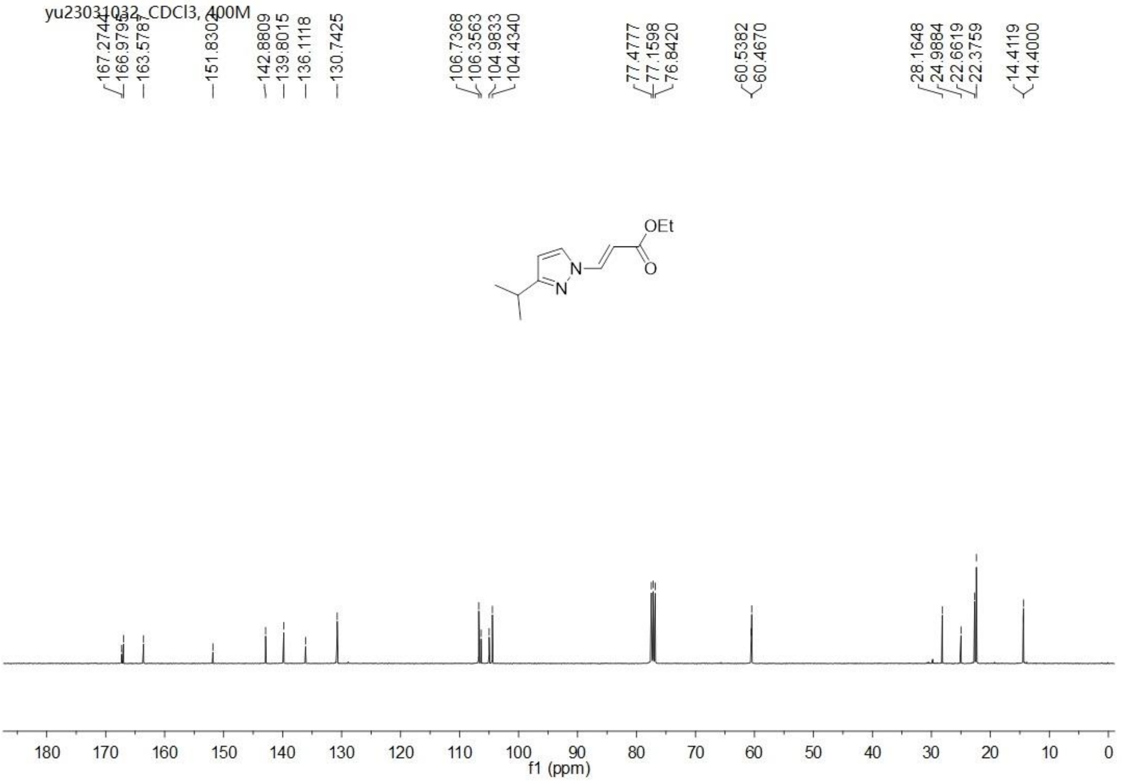

^{13}C NMR of **5h** and **5h’**

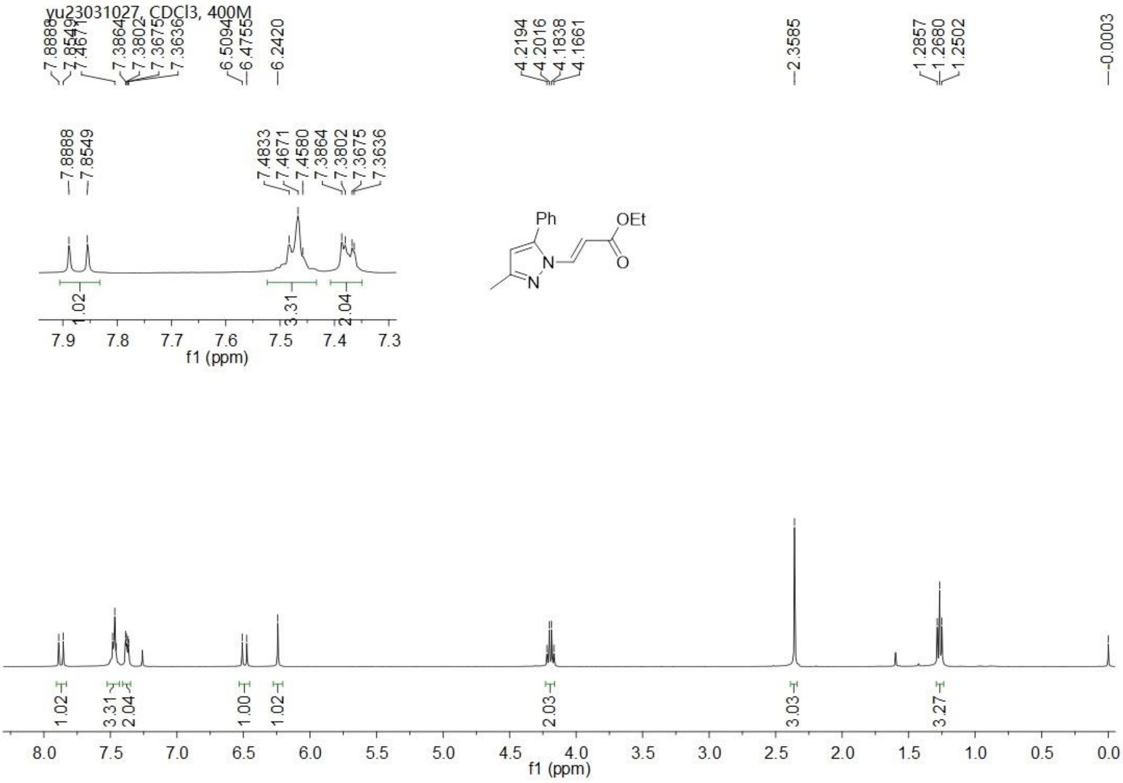

^{1}H NMR of **5i**

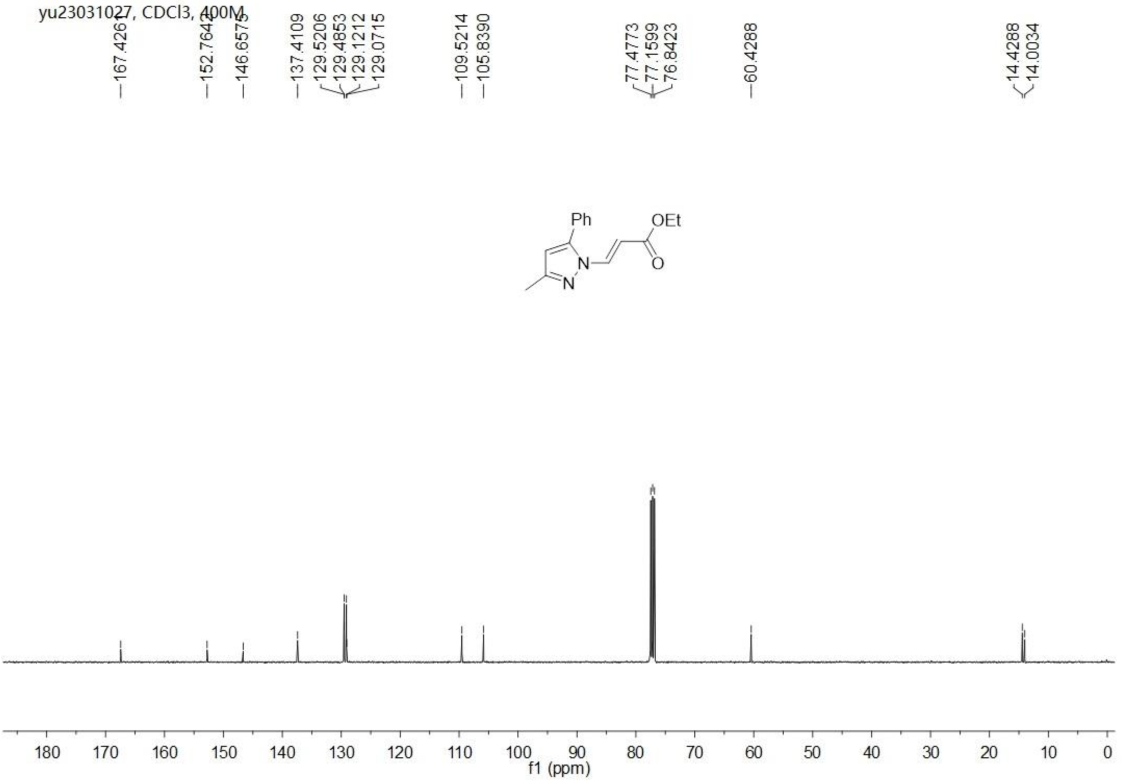

^{13}C NMR of **5i**

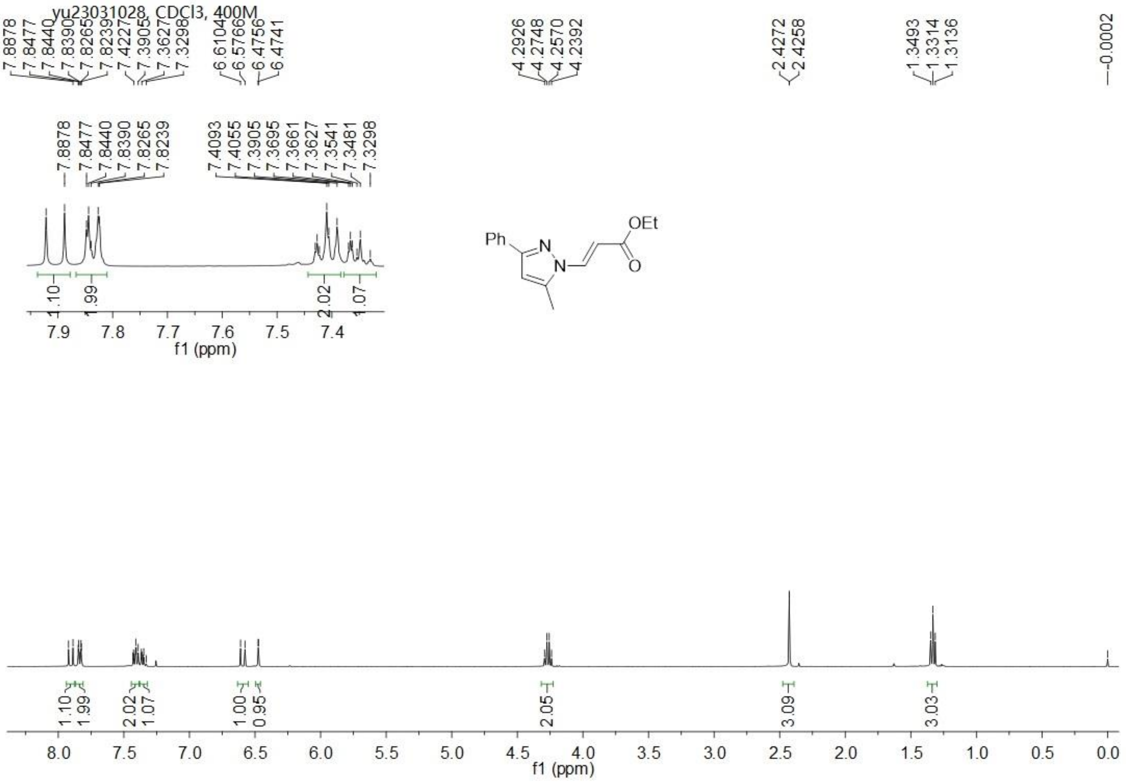

^{1}H NMR of **5i’**

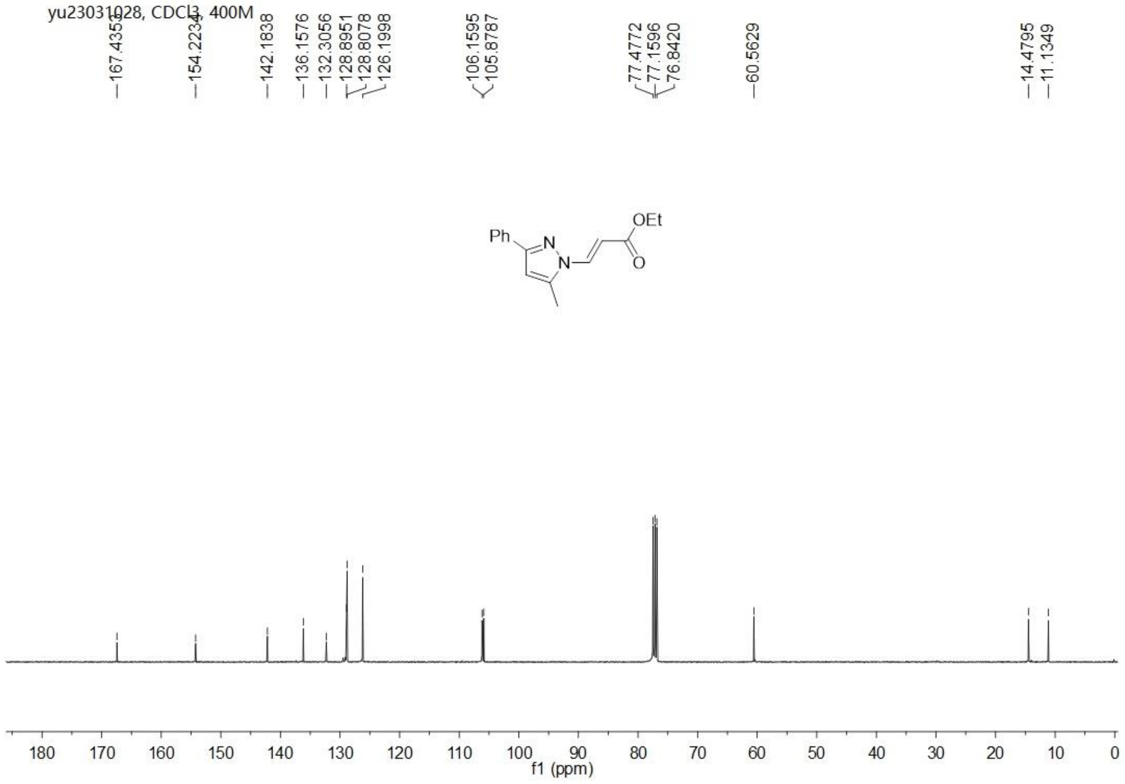

^{13}C NMR of **5i’**

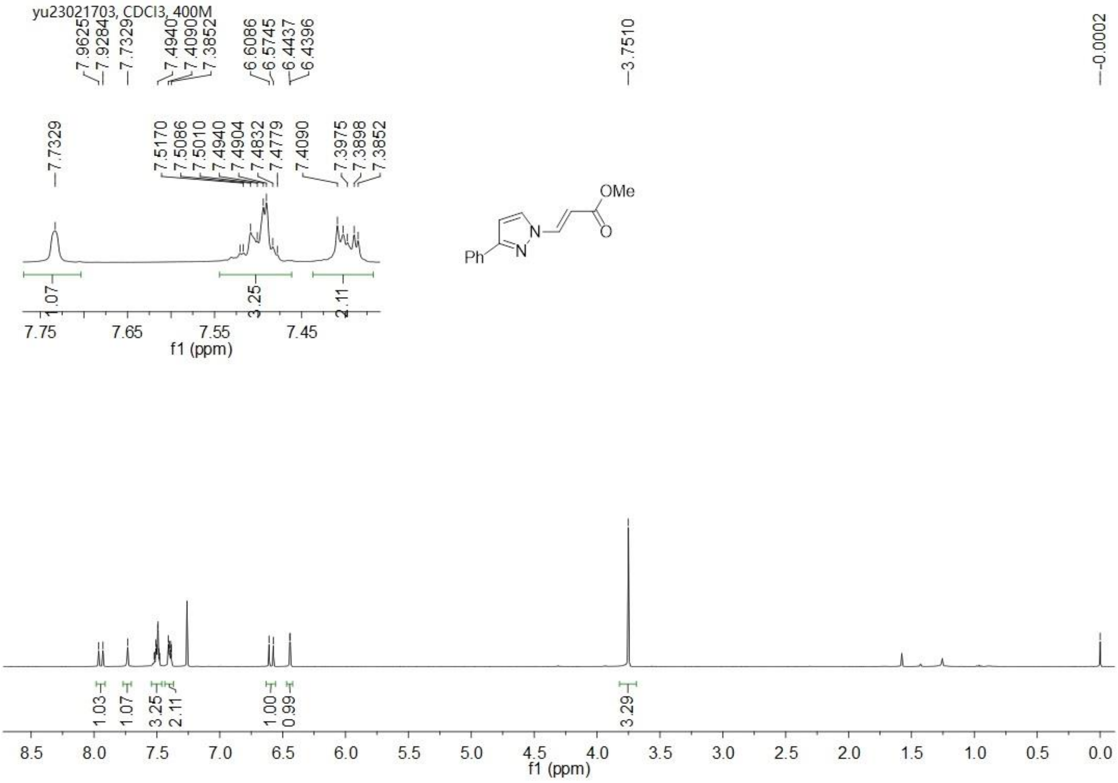

^{1}H NMR of **5j**

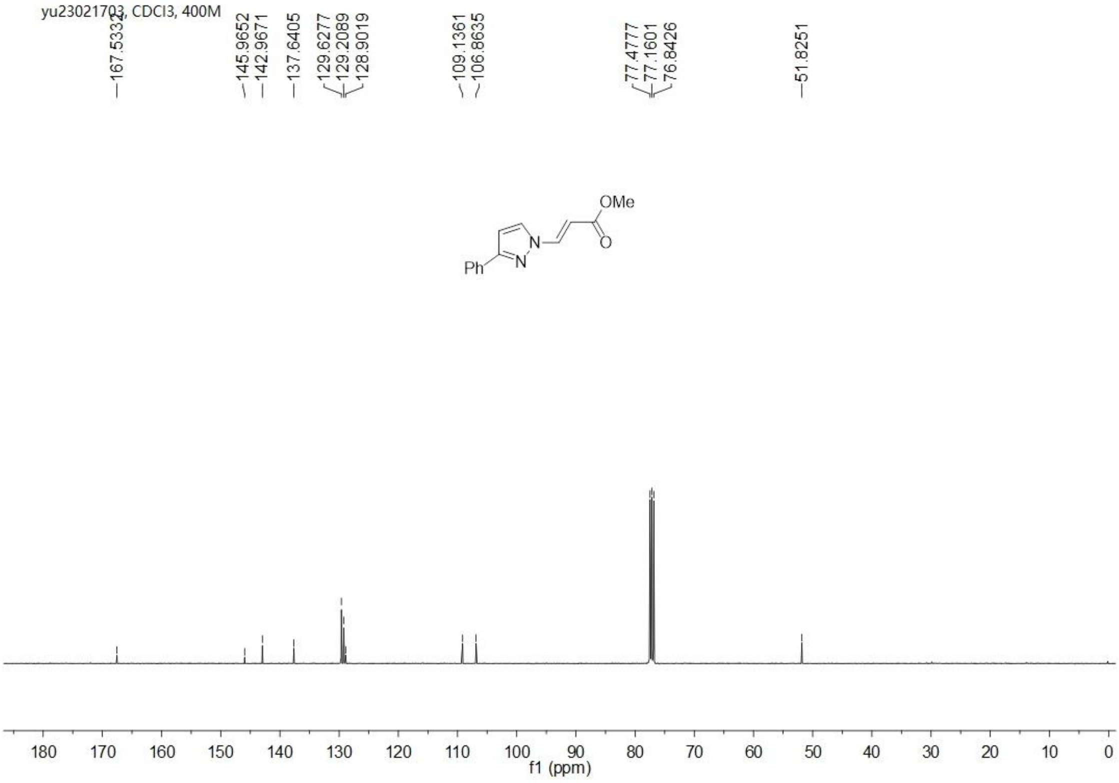

^{13}C NMR of **5j**

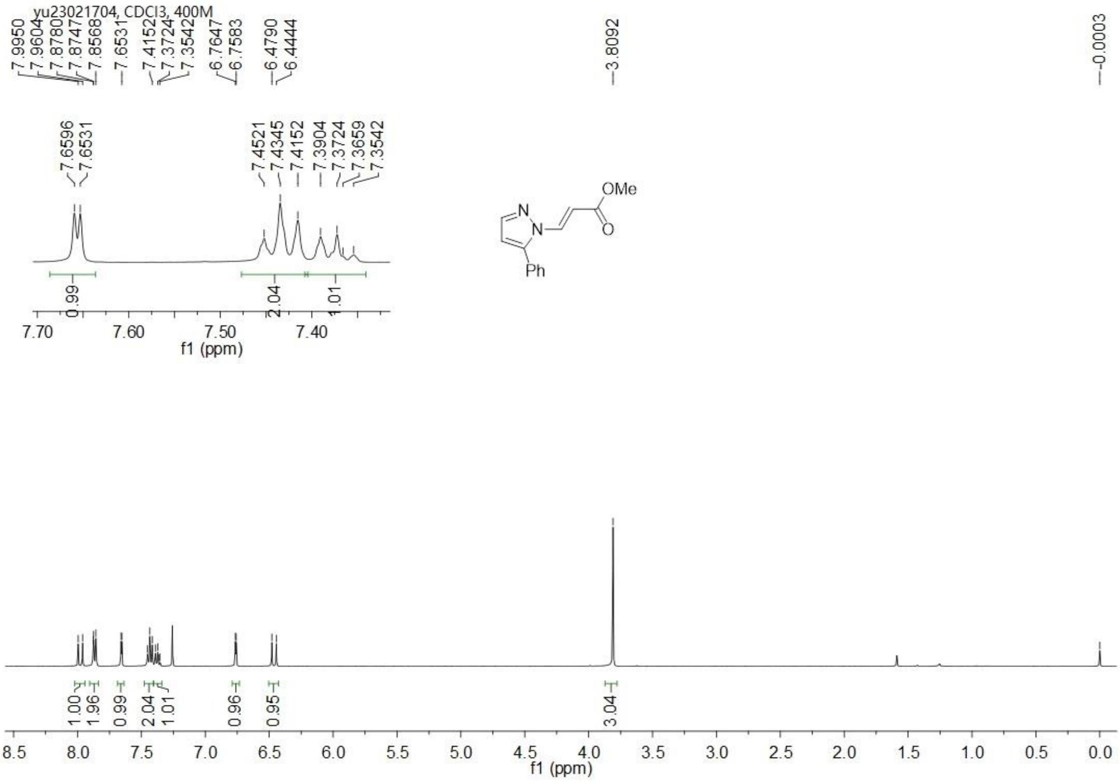

^{1}H NMR of **5j'**

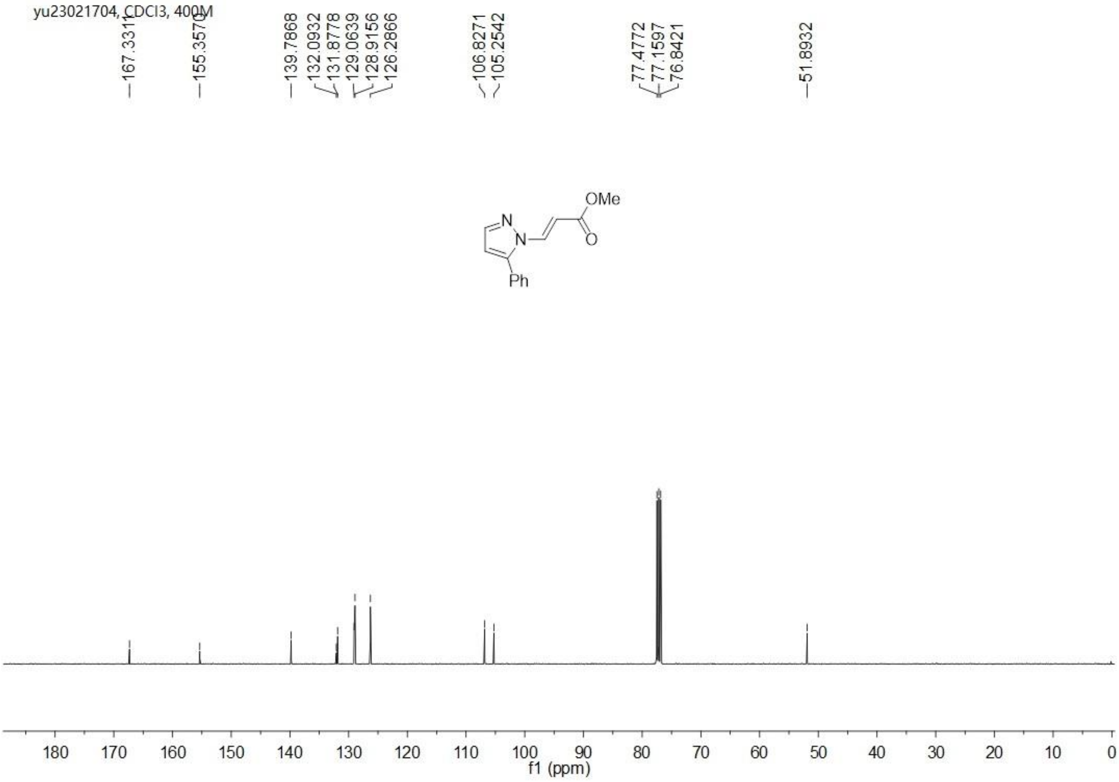

^{13}C NMR of **5j'**

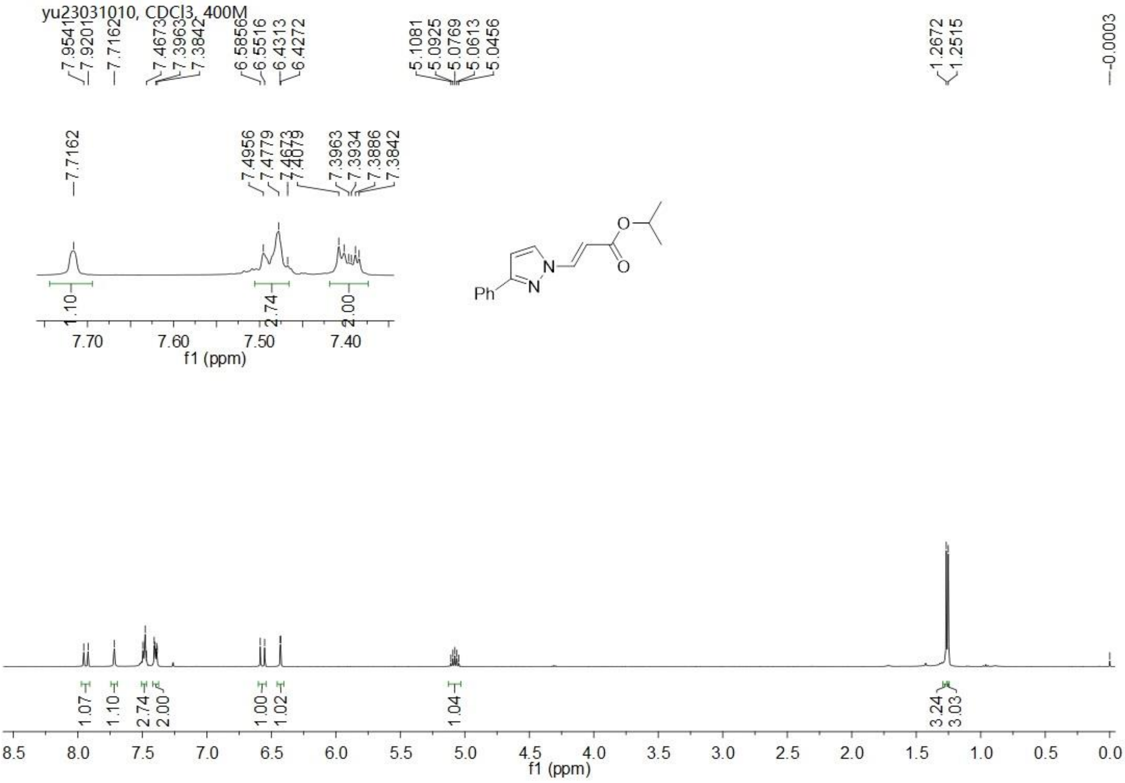

^{1}H NMR of **5k**

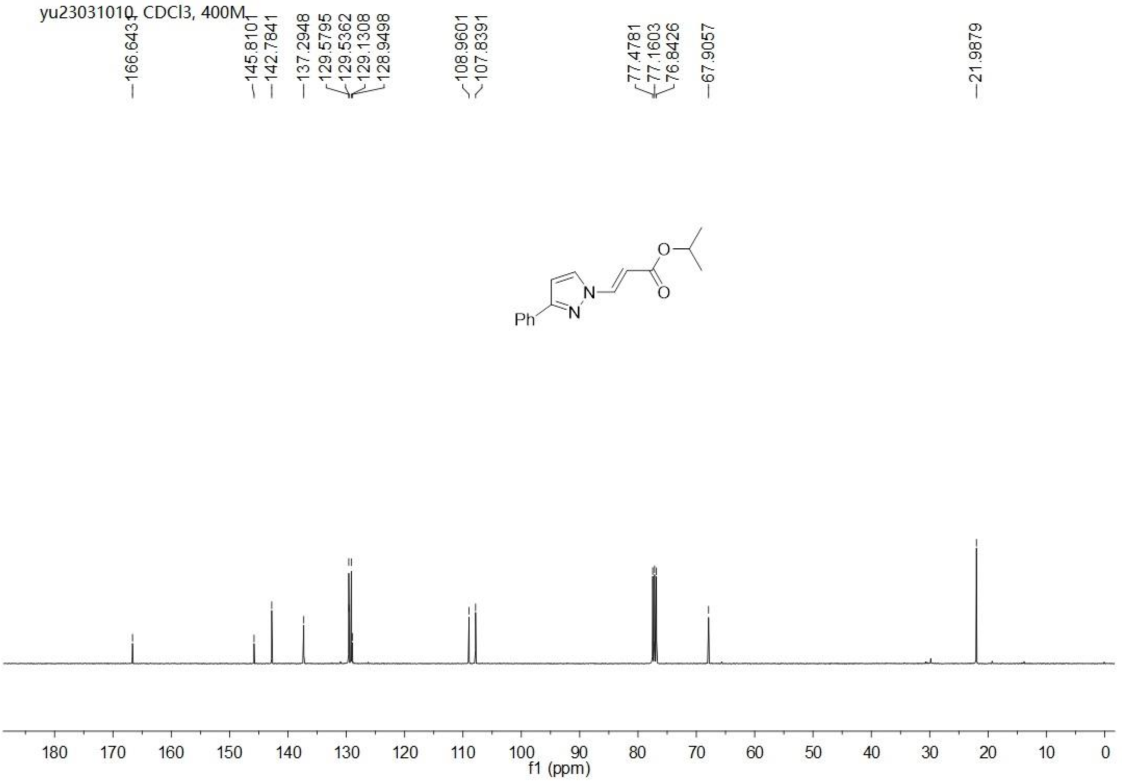

^{13}C NMR of **5k**

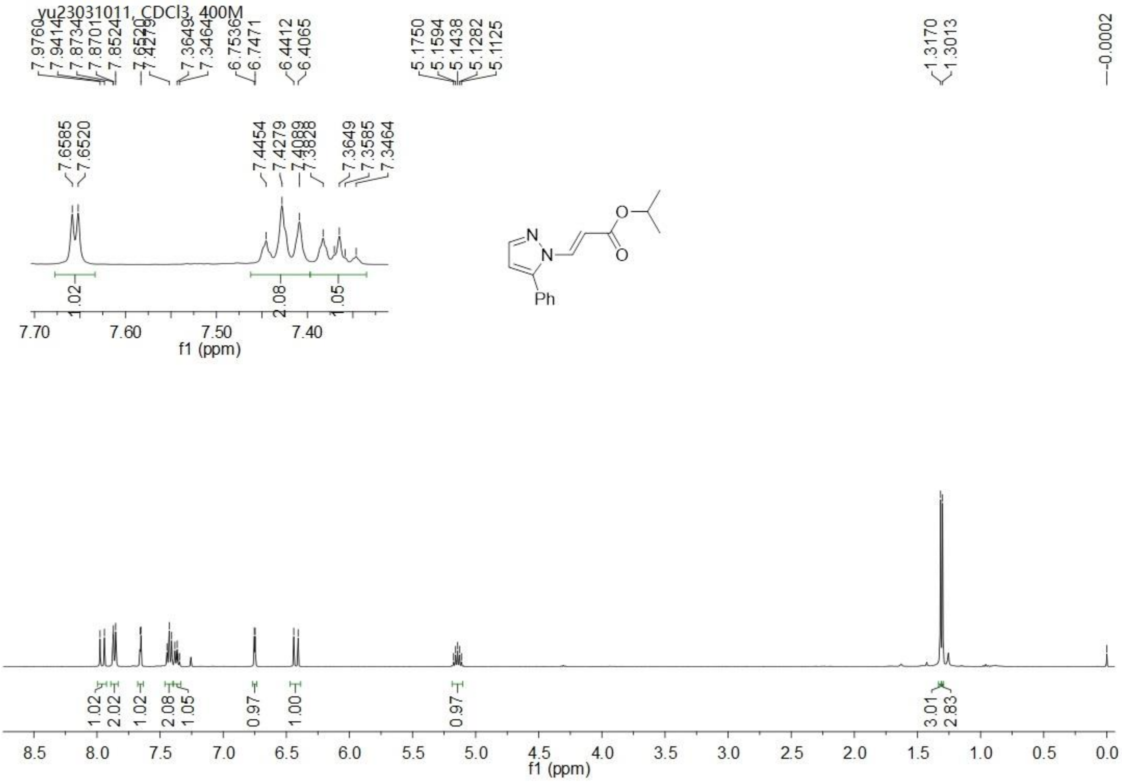

^{1}H NMR of **5k’**

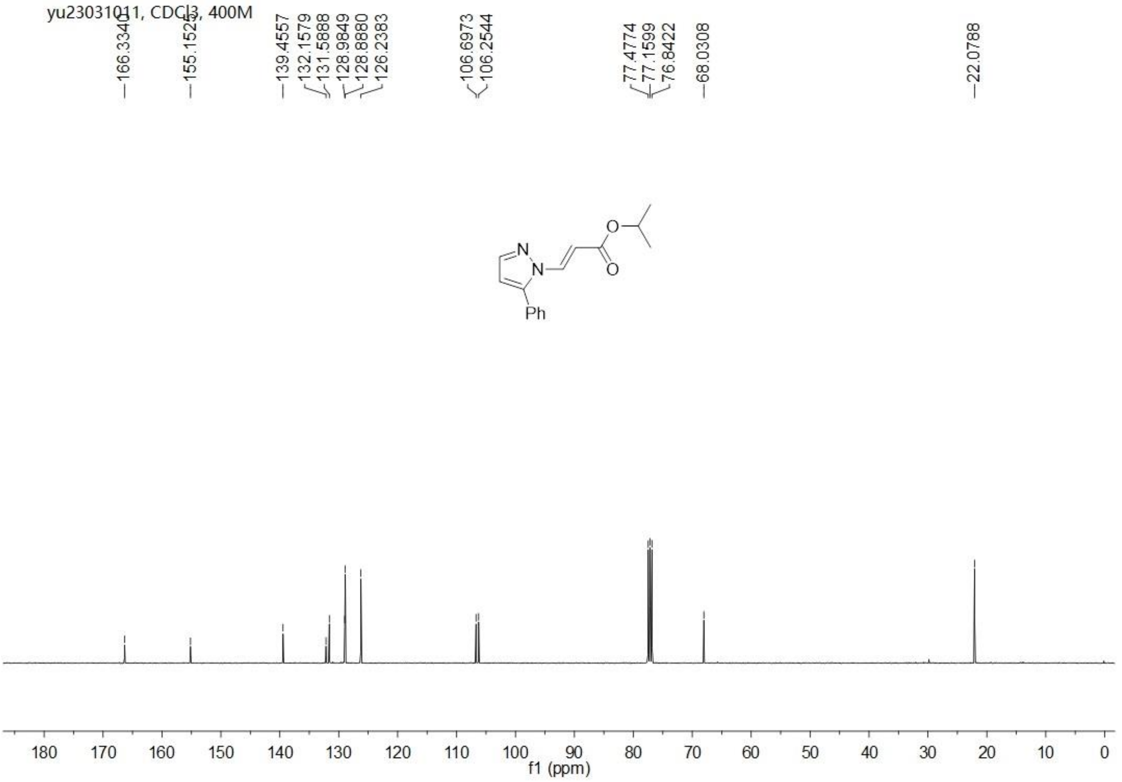

^{13}C NMR of **5k’**

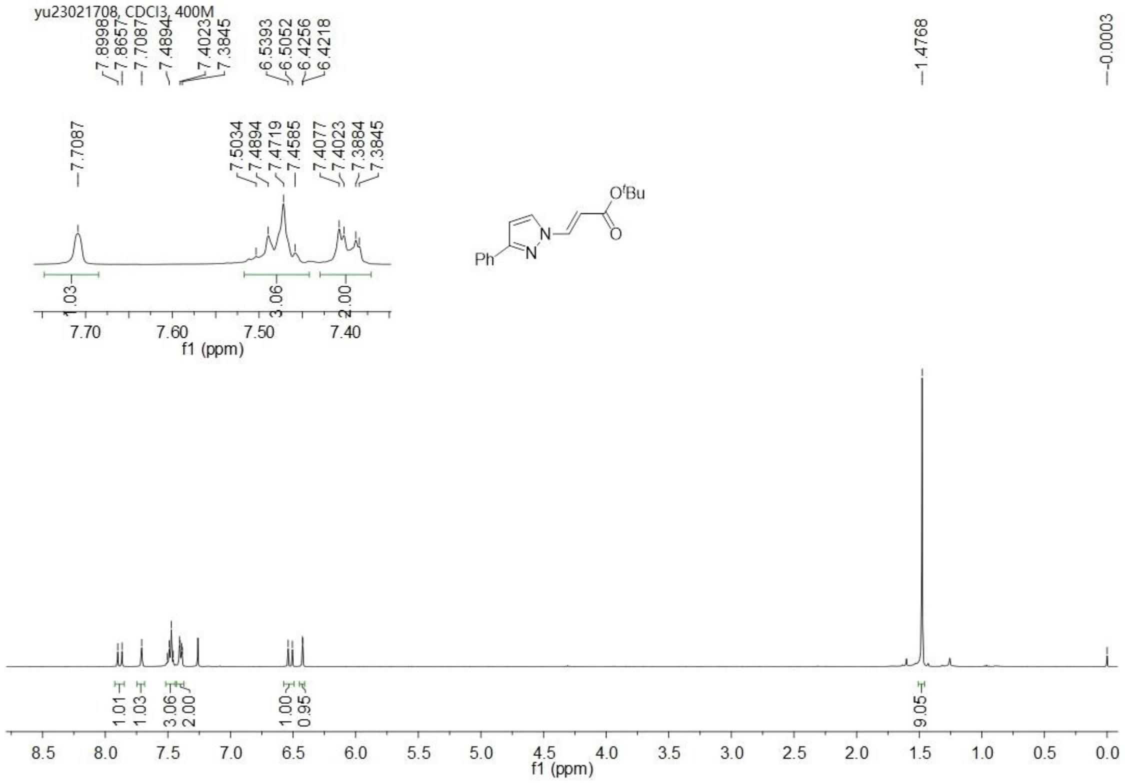

^{1}H NMR of **5l**

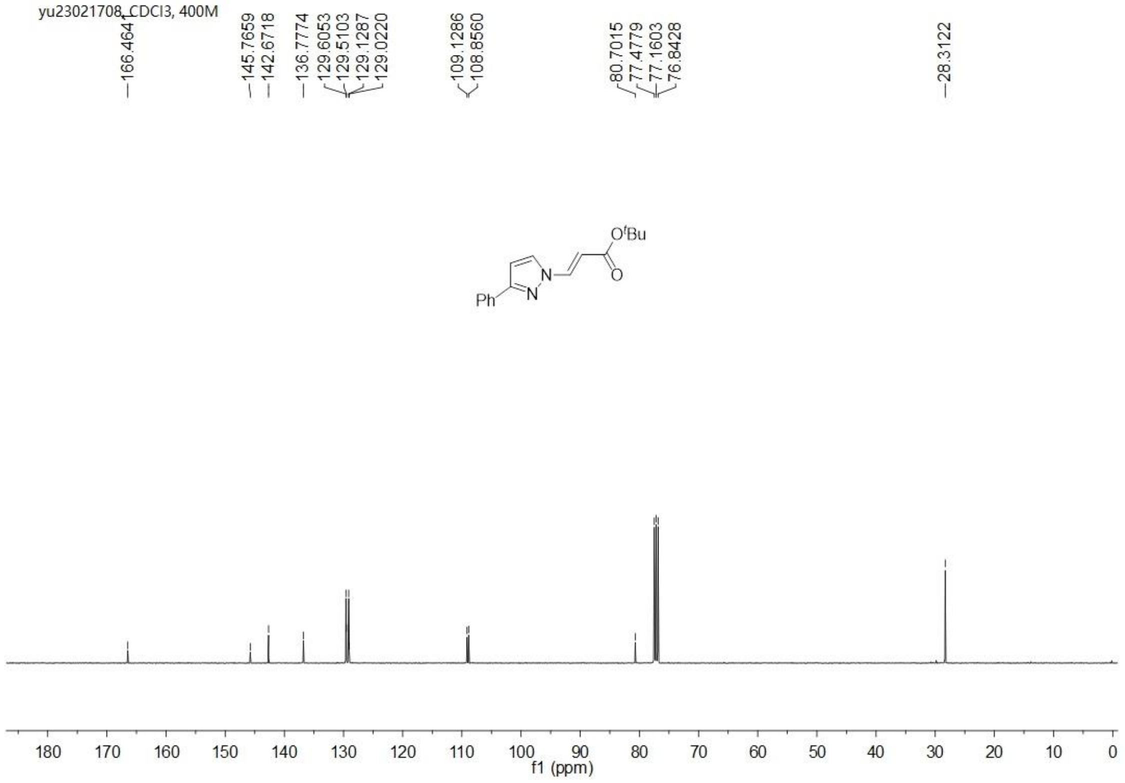

^{13}C NMR of **5l**

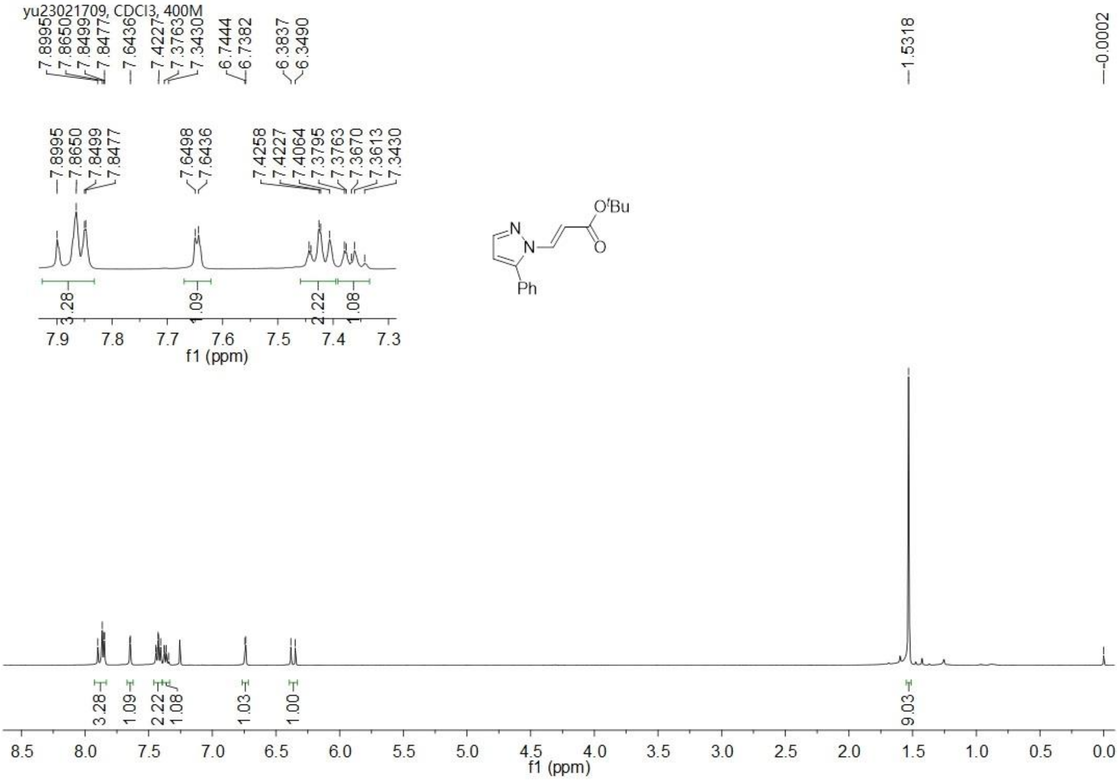

^{1}H NMR of **5l’**

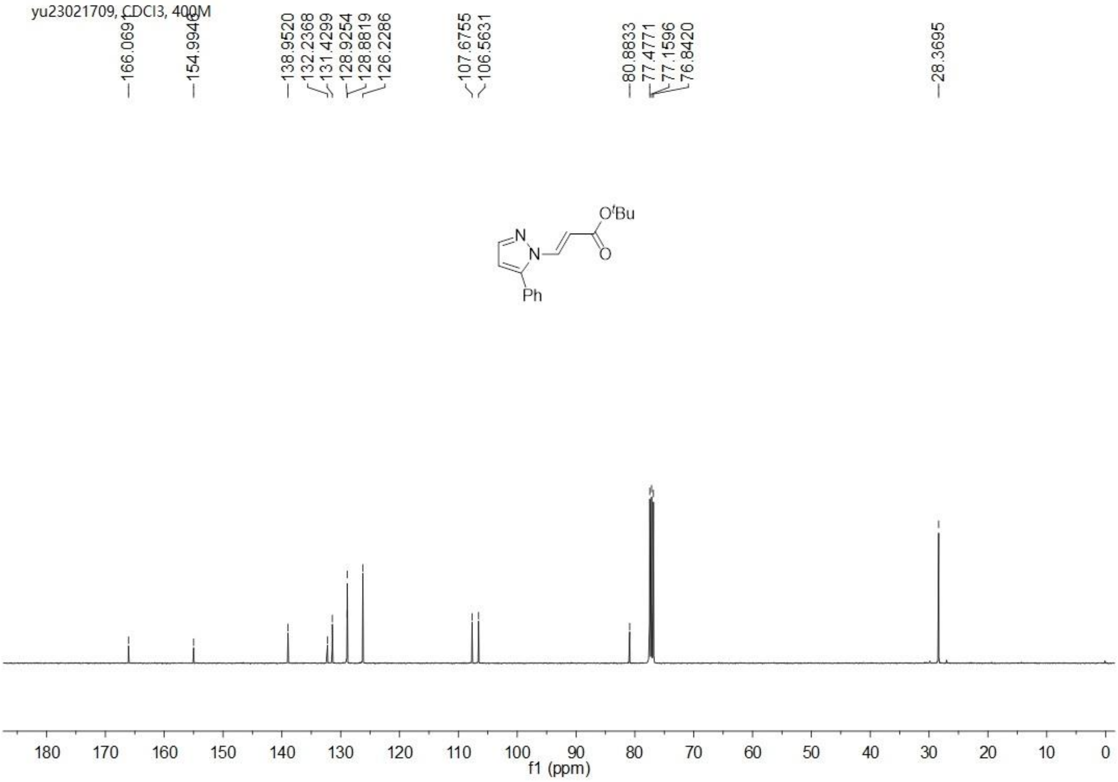

^{13}C NMR of **5l’**

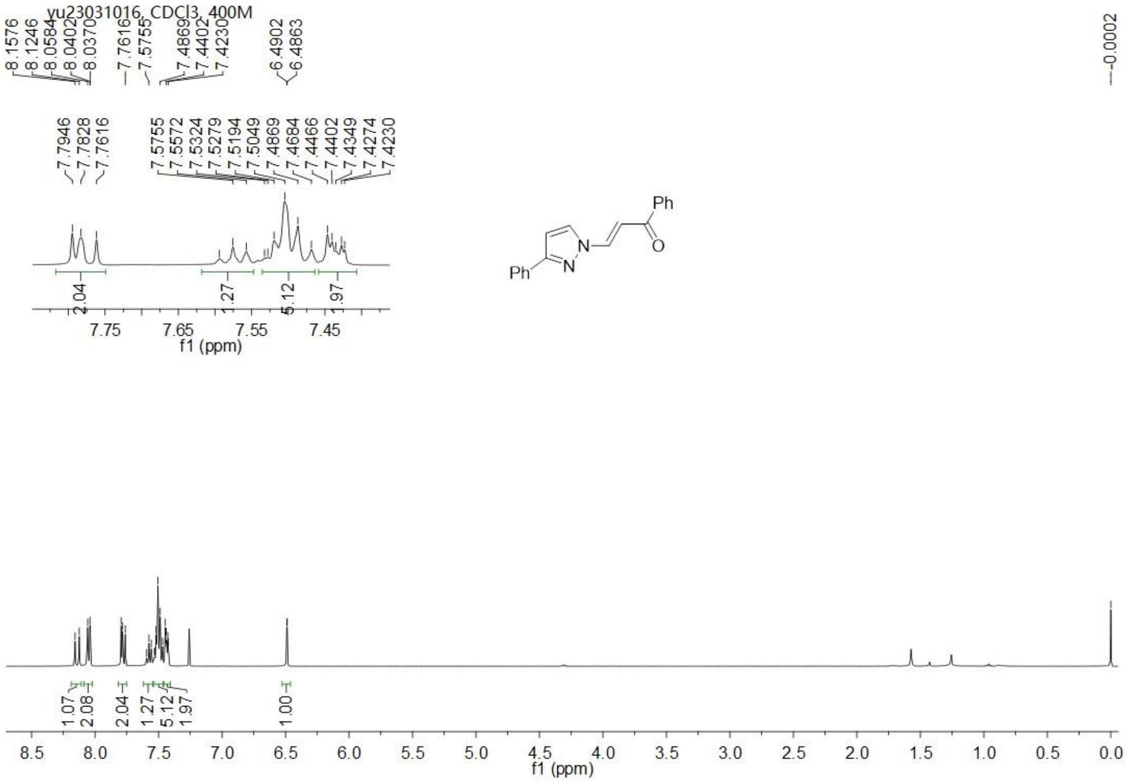

^{1}H NMR of **5m**

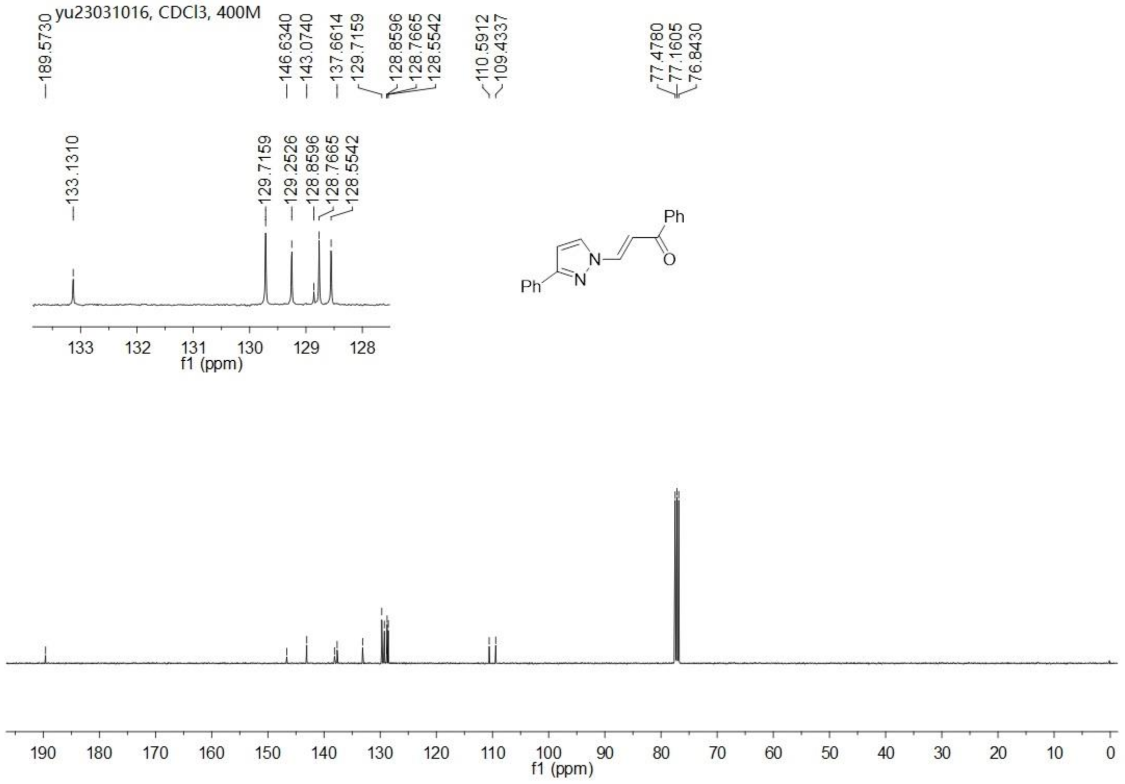

^{13}C NMR of **5m**

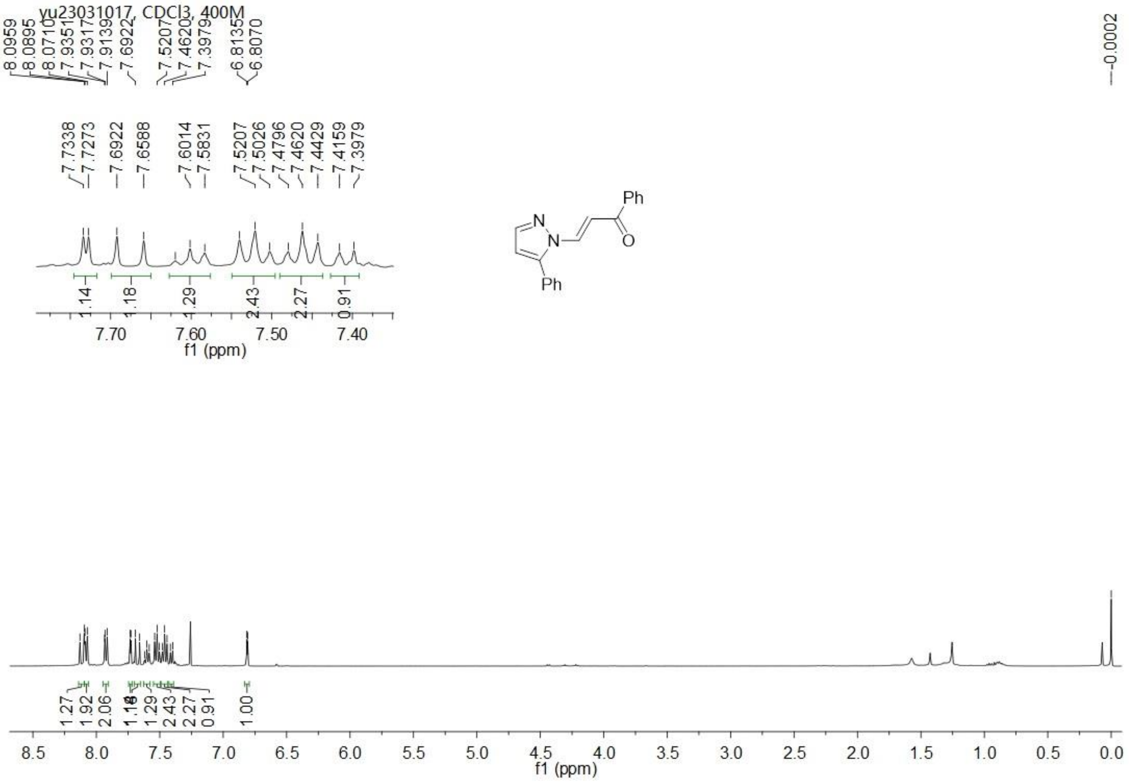

^{1}H NMR of **5m’**

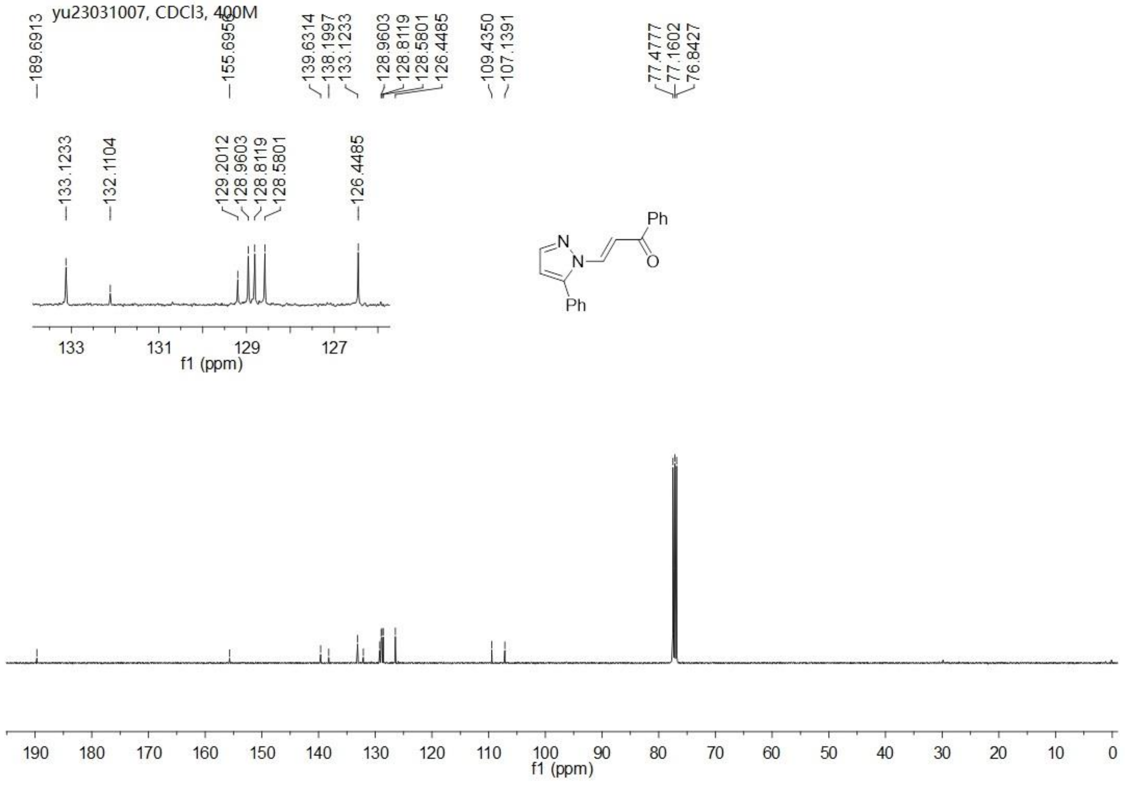

^{13}C NMR of **5m’**

Copies of ^{1}H NMR and ^{13}C NMR spectra of compounds 6

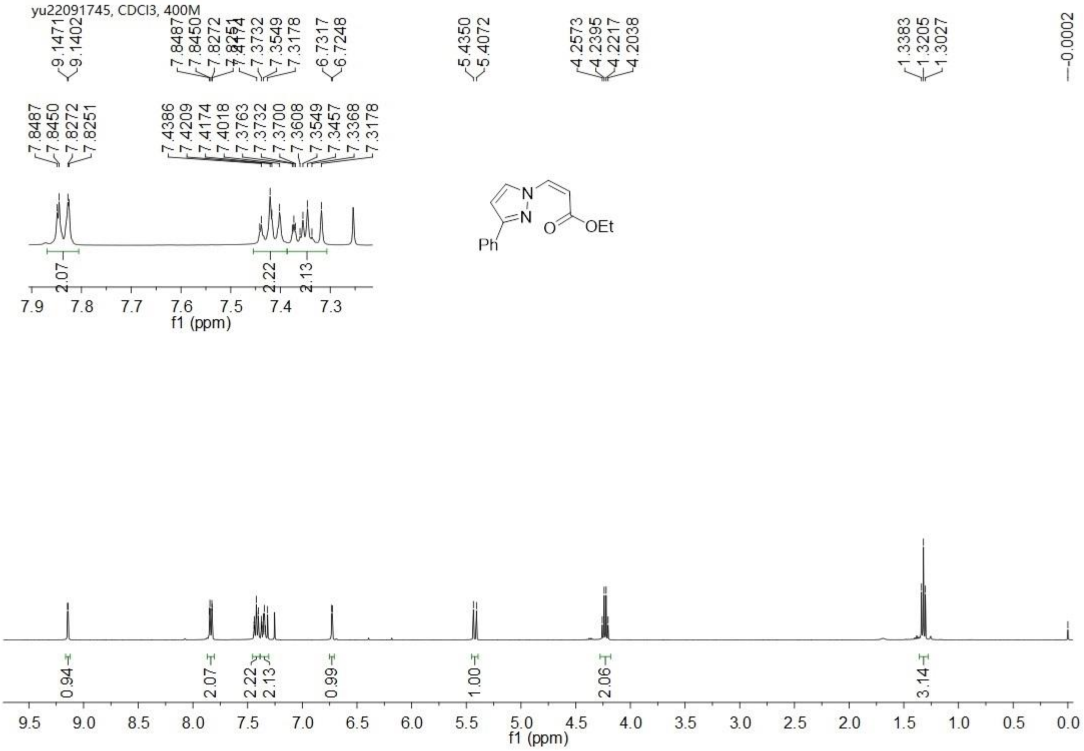

^{1}H NMR of **6a**

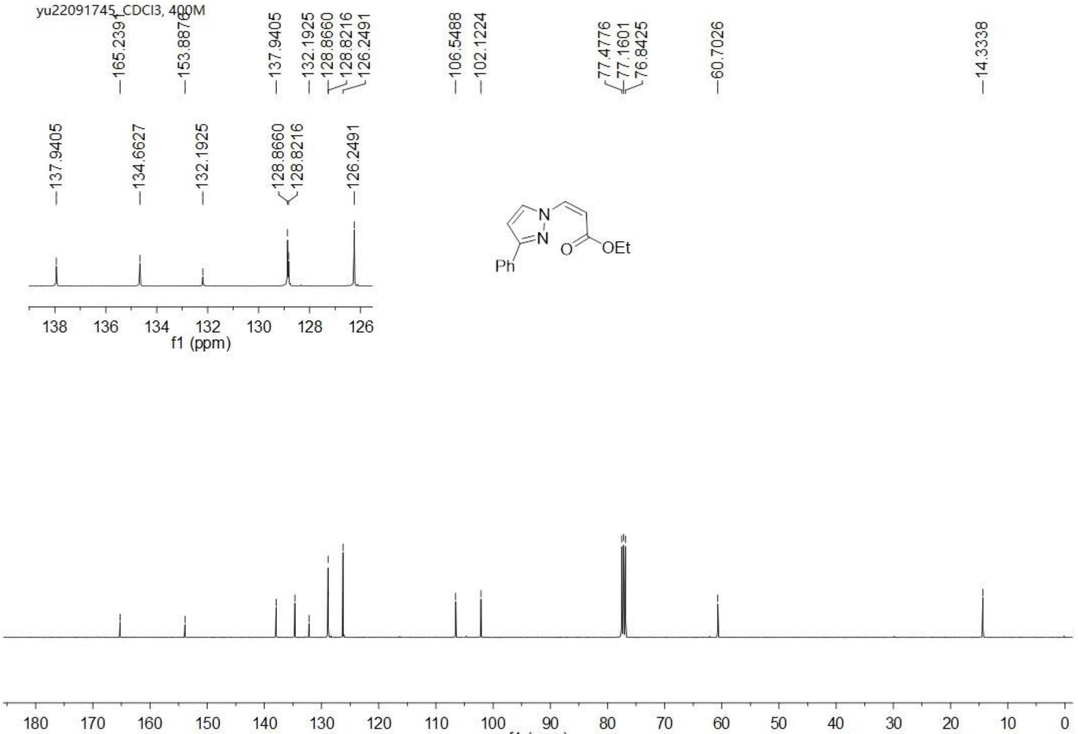

^{13}C NMR of **6a**

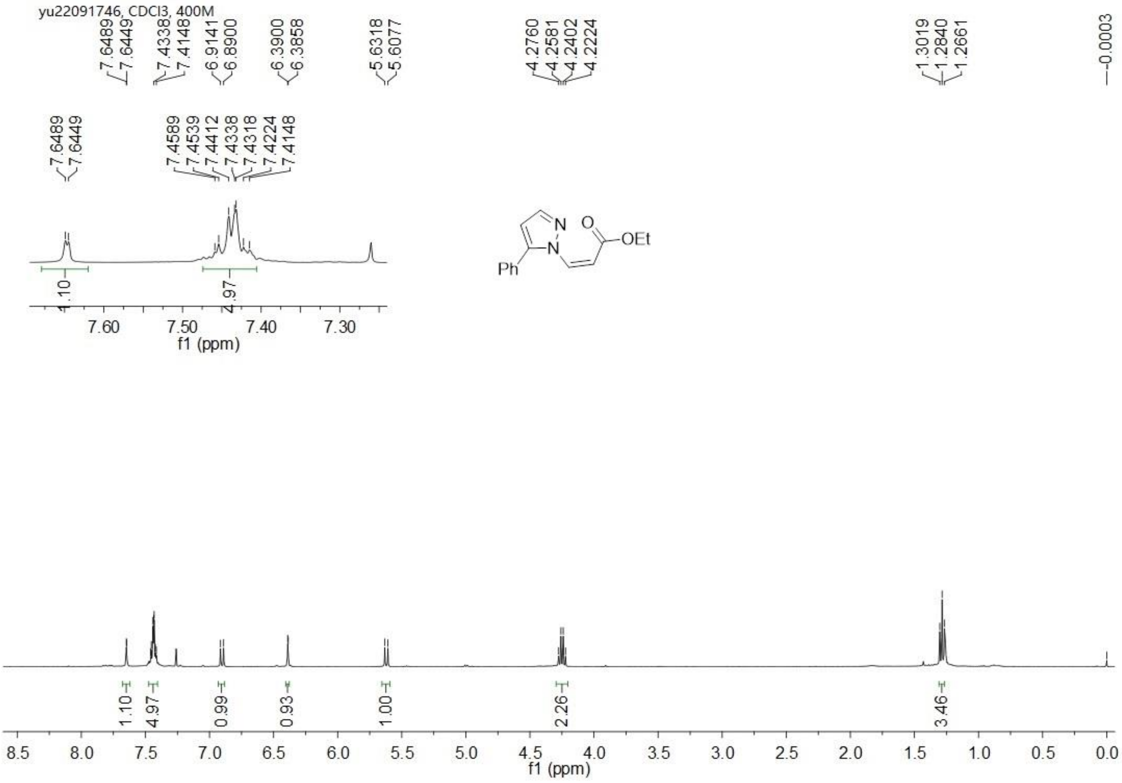

^{1}H NMR of **6a’**

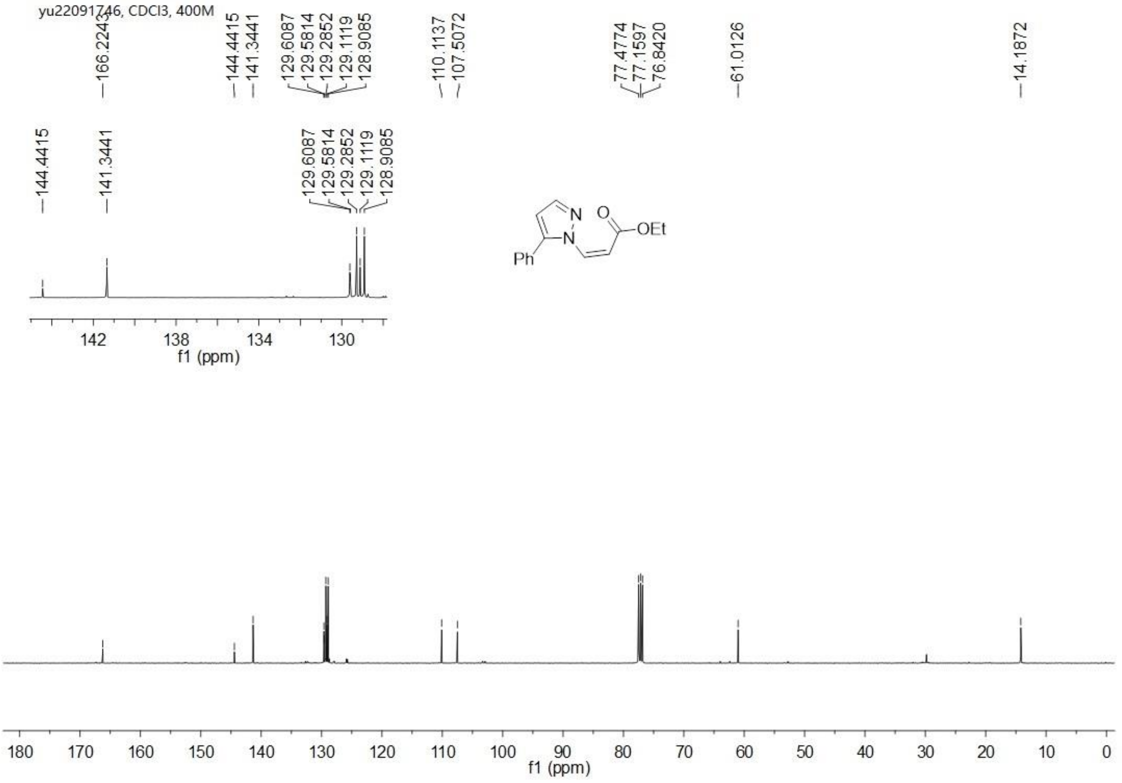

^{13}C NMR of **6a’**

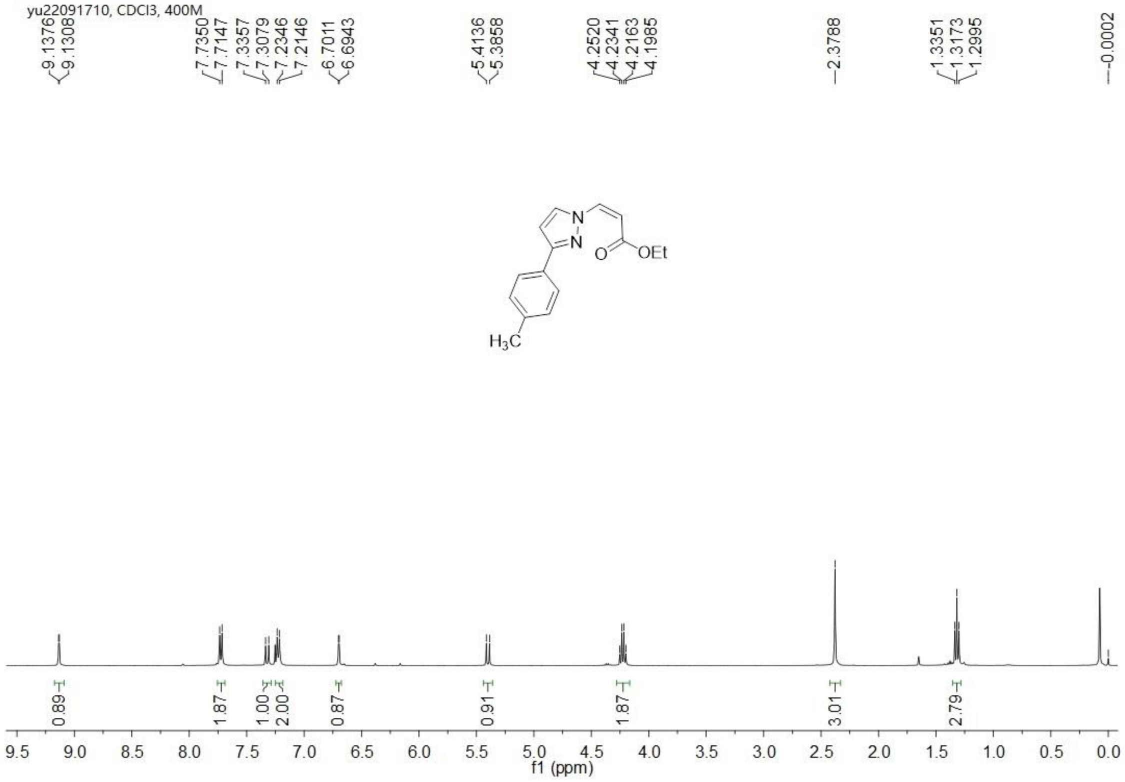

^{1}H NMR of **6b**

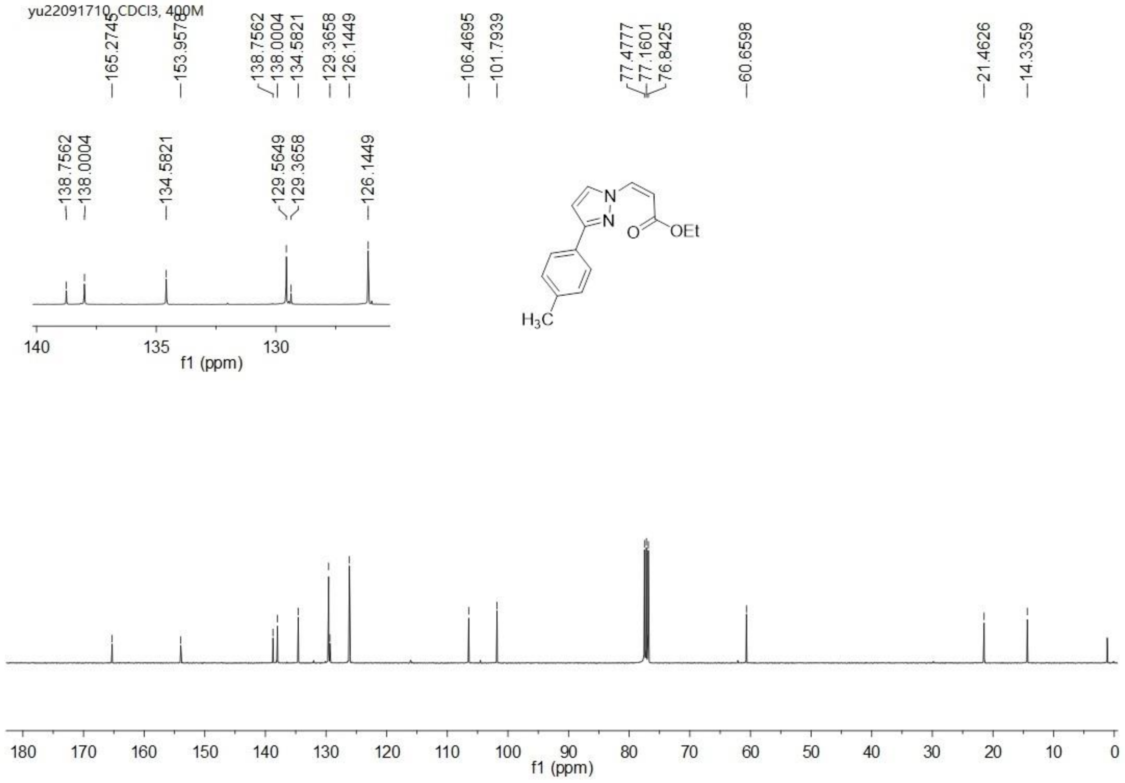

^{13}C NMR of **6b**

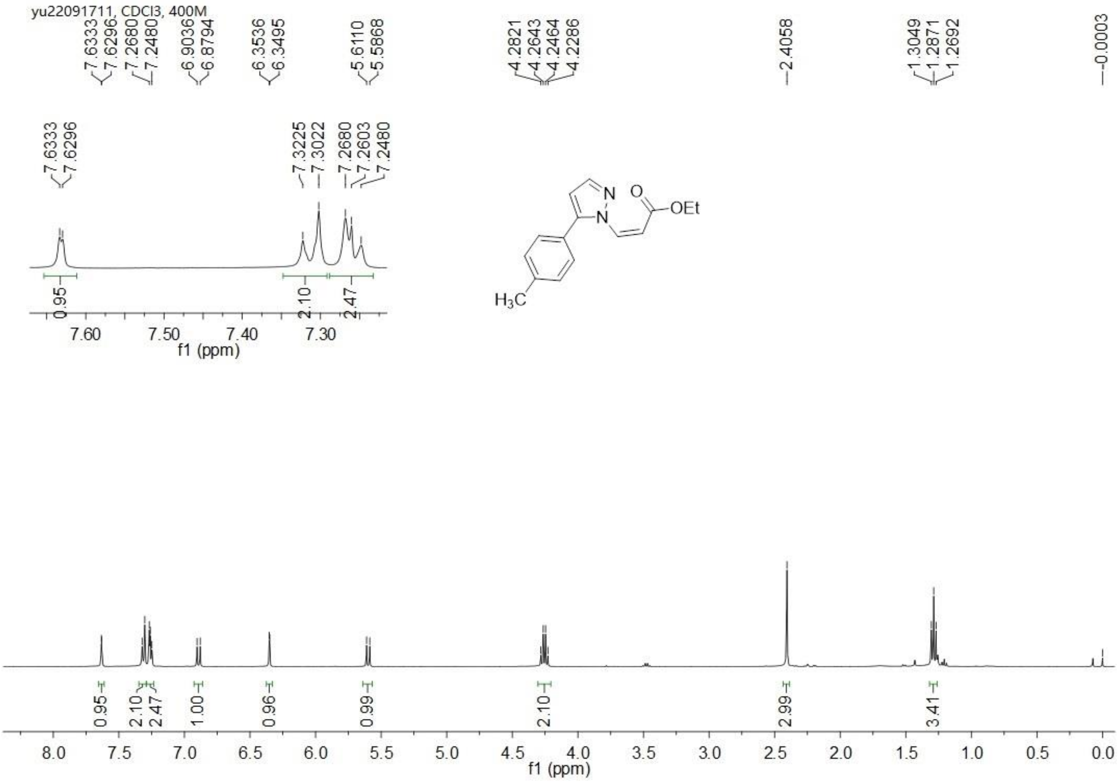

^{1}H NMR of **6b’**

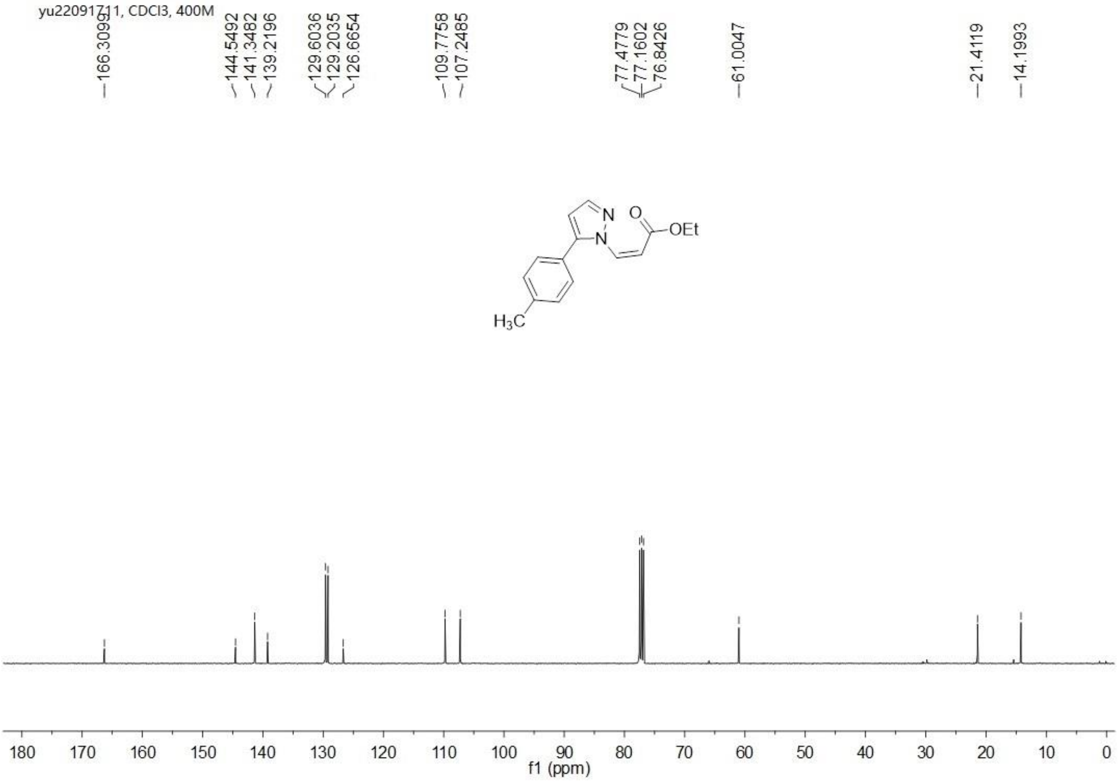

^{13}C NMR of **6b’**

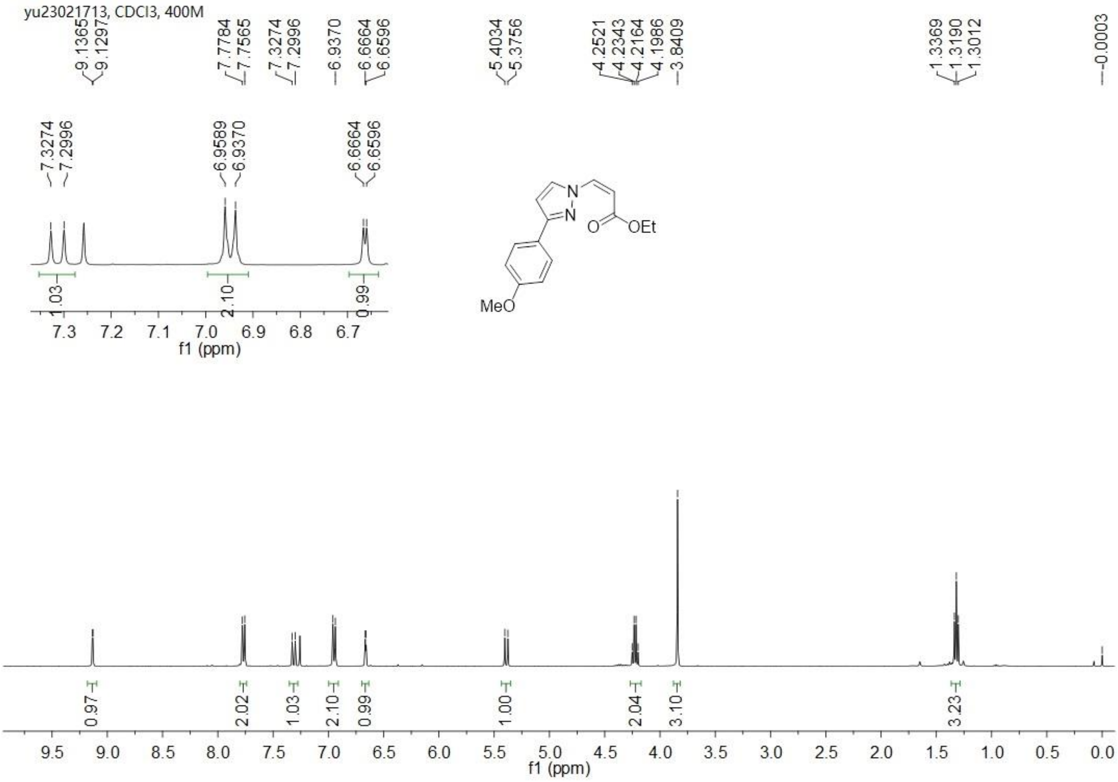

^{1}H NMR of **6c**

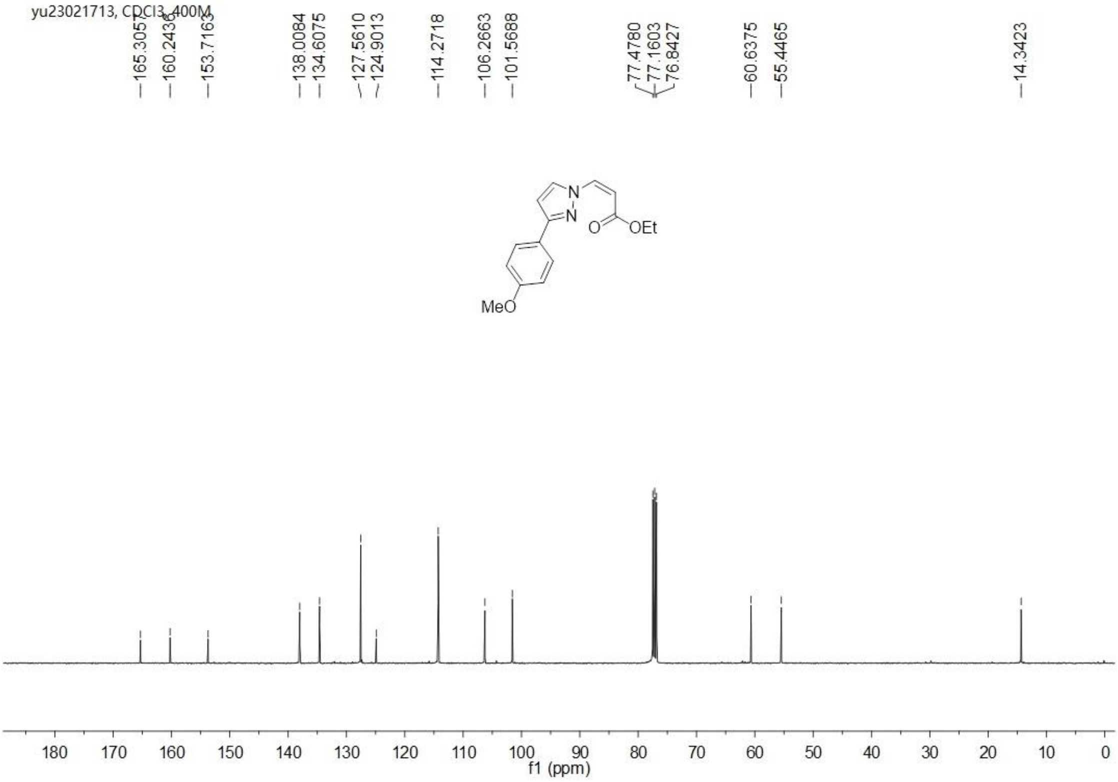

^{13}C NMR of **6c**

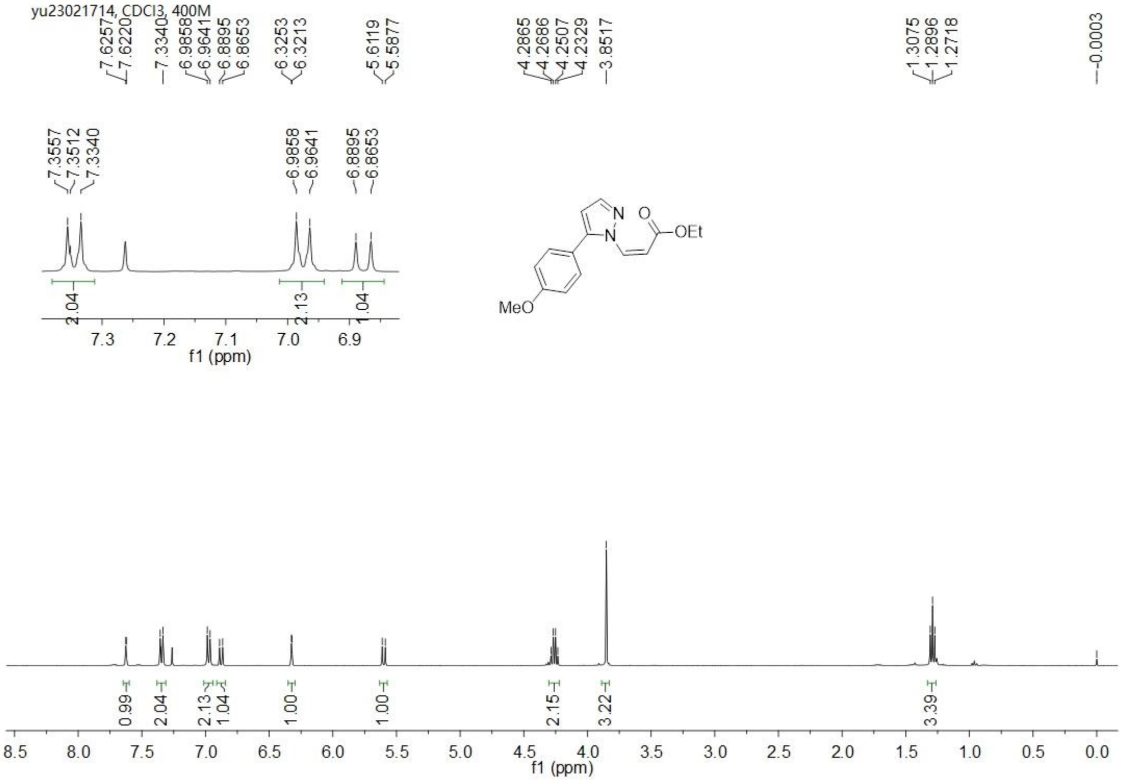

^{1}H NMR of **6c’**

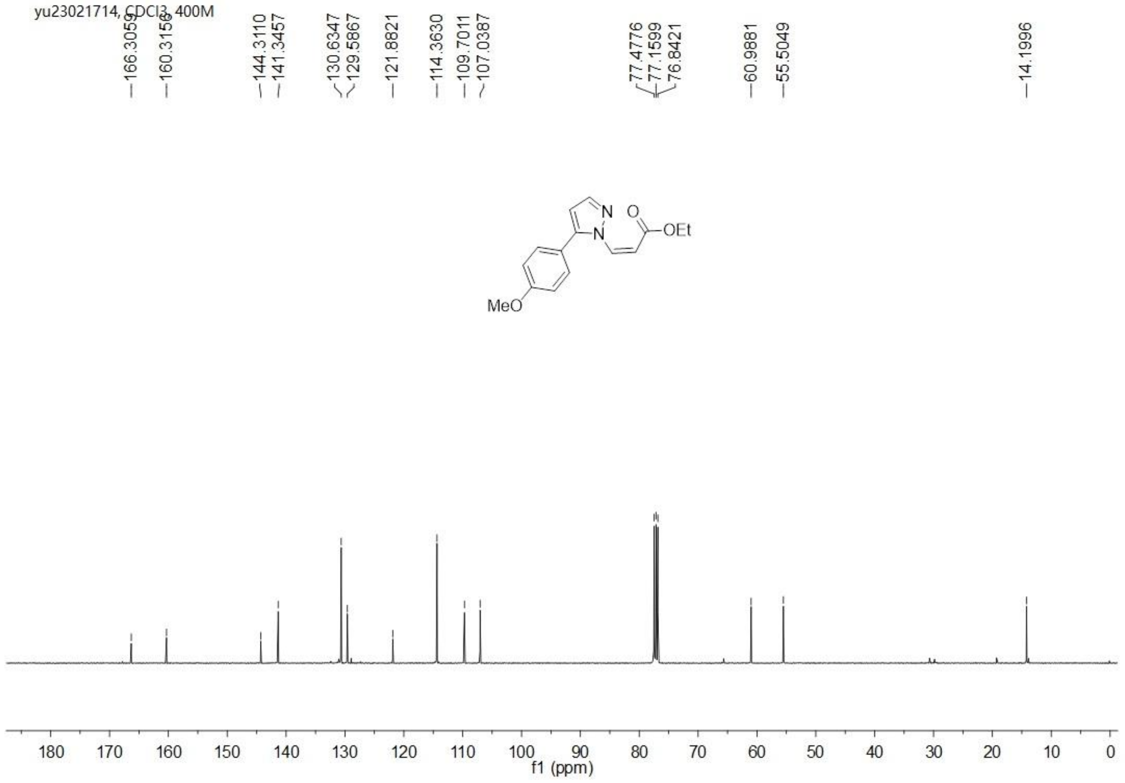

^{13}C NMR of **6c’**

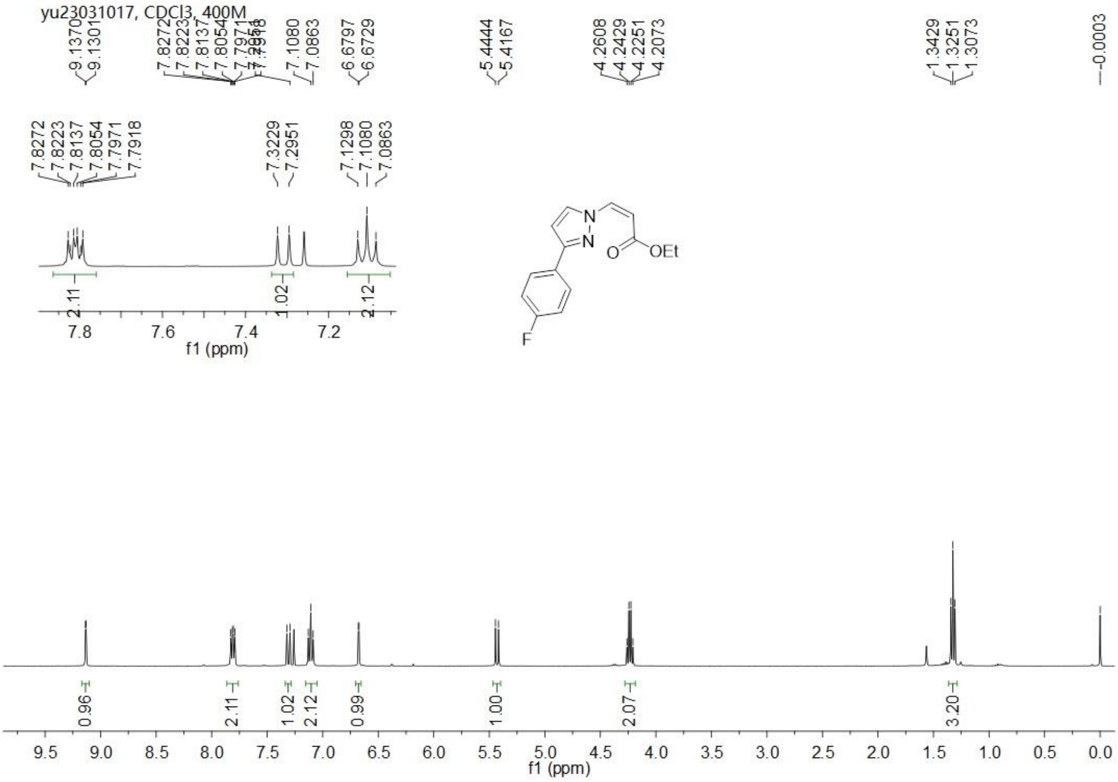

^{1}H NMR of **6d**

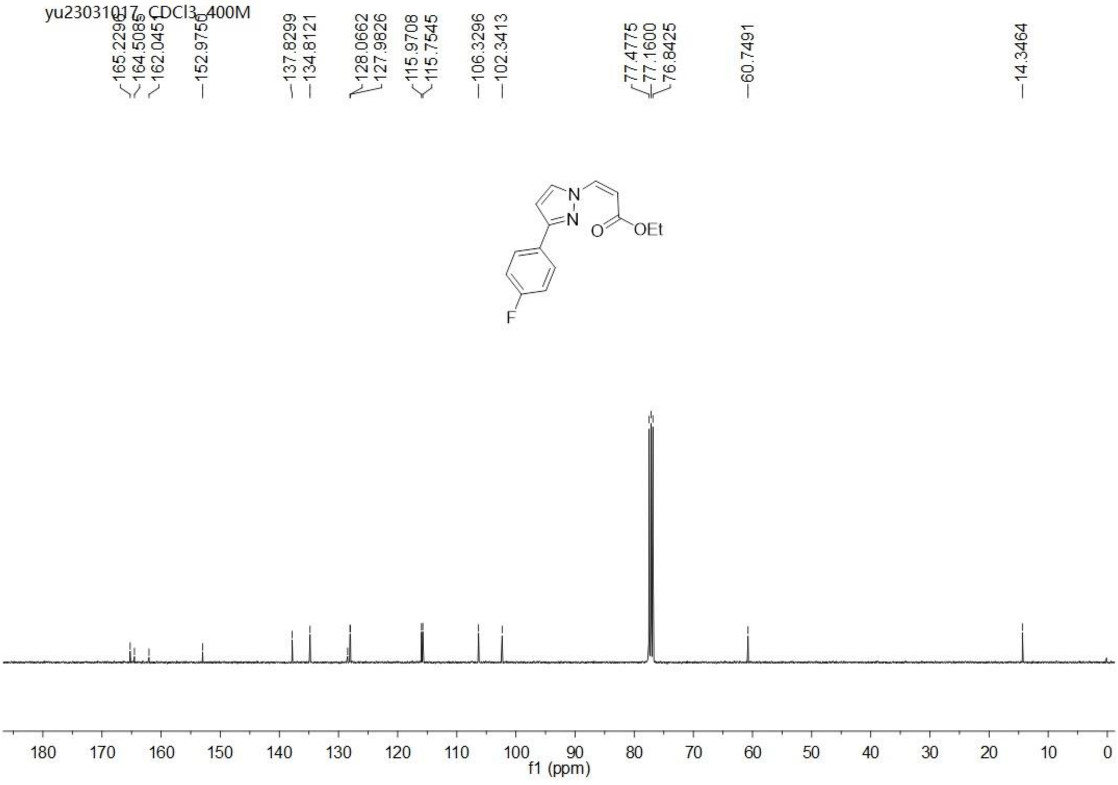

^{13}C NMR of **6d**

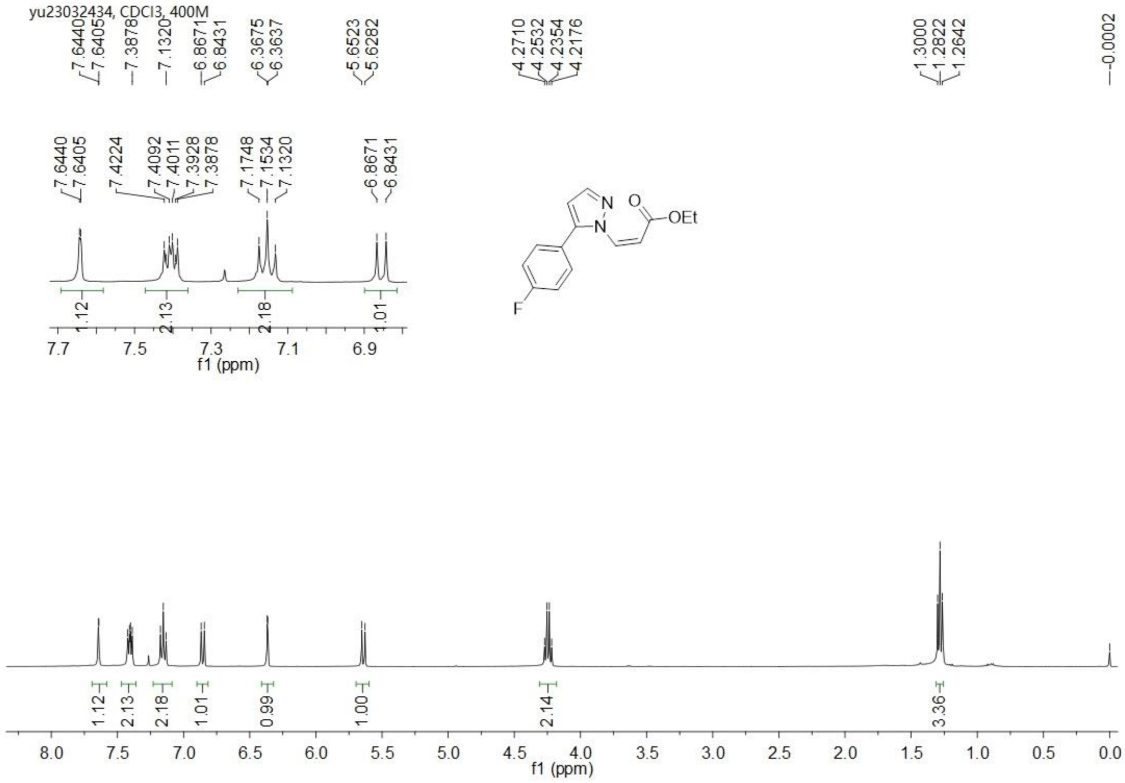

^{1}H NMR of **6d’**

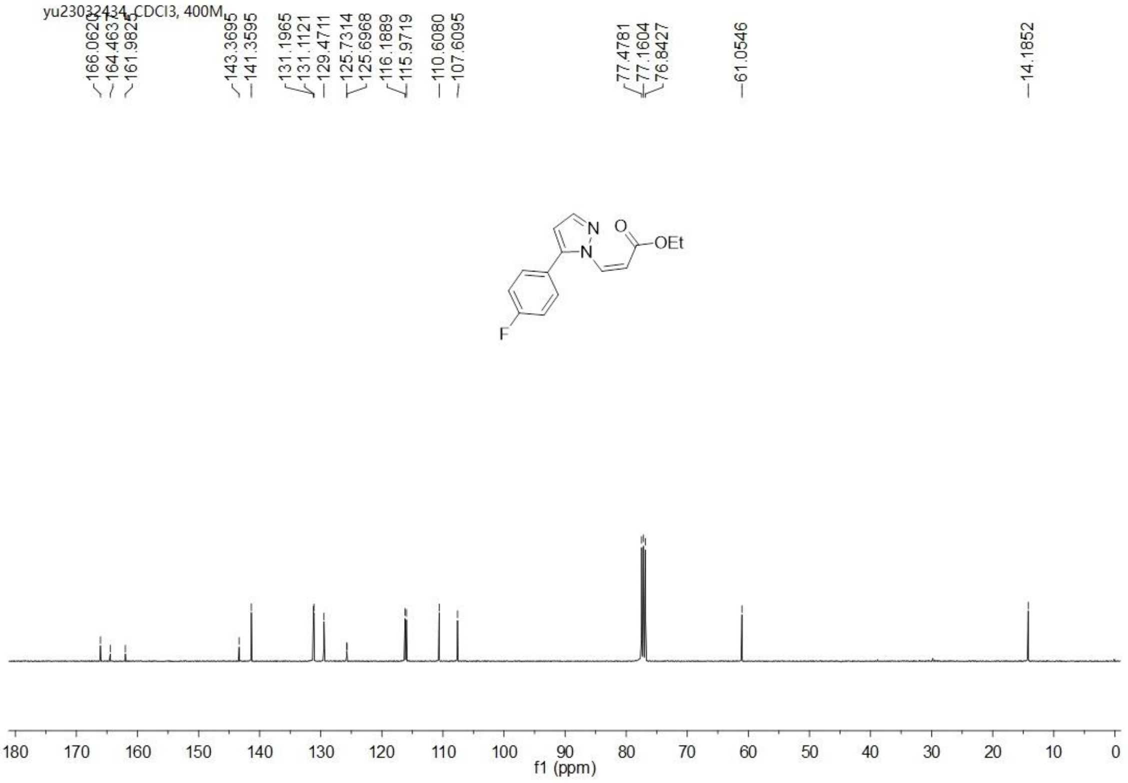

^{13}C NMR of **6d’**

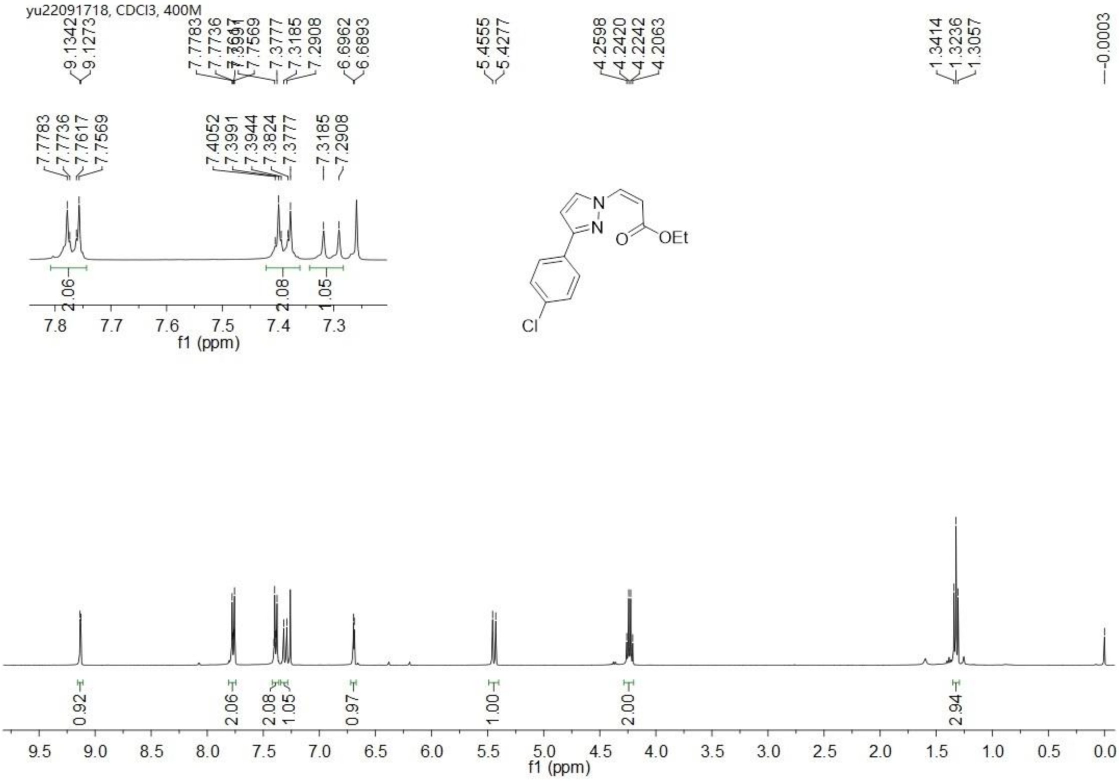

^{1}H NMR of **6e**

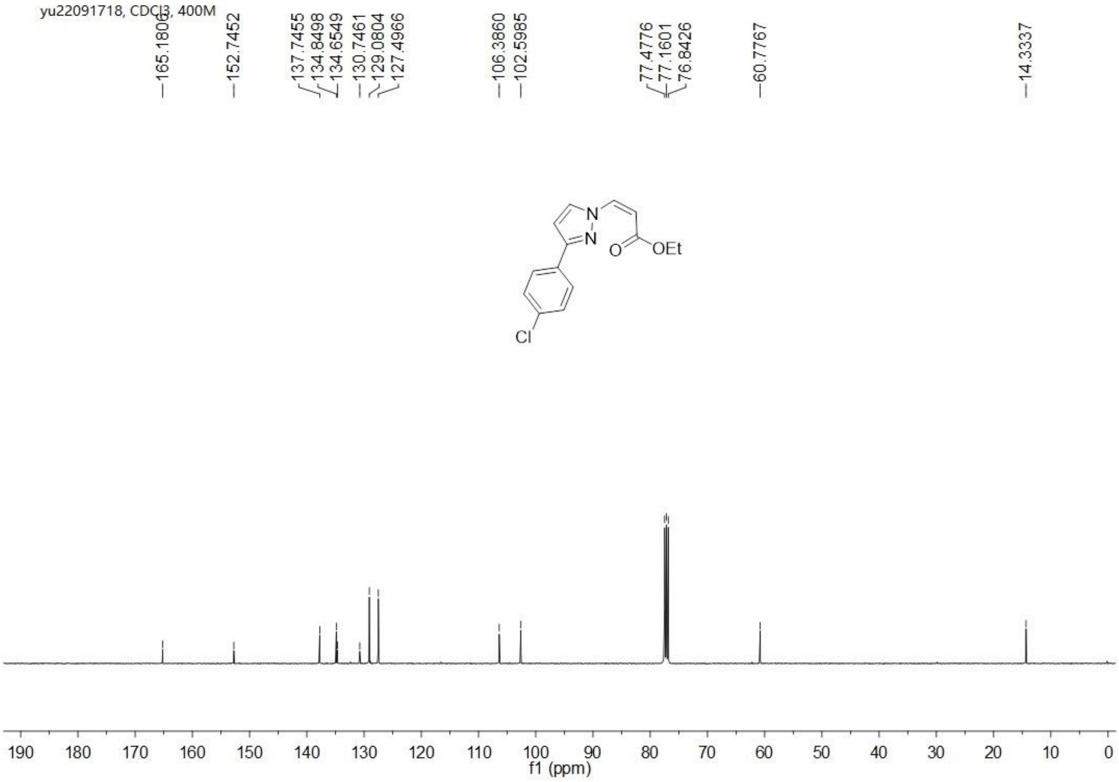

^{13}C NMR of **6e**

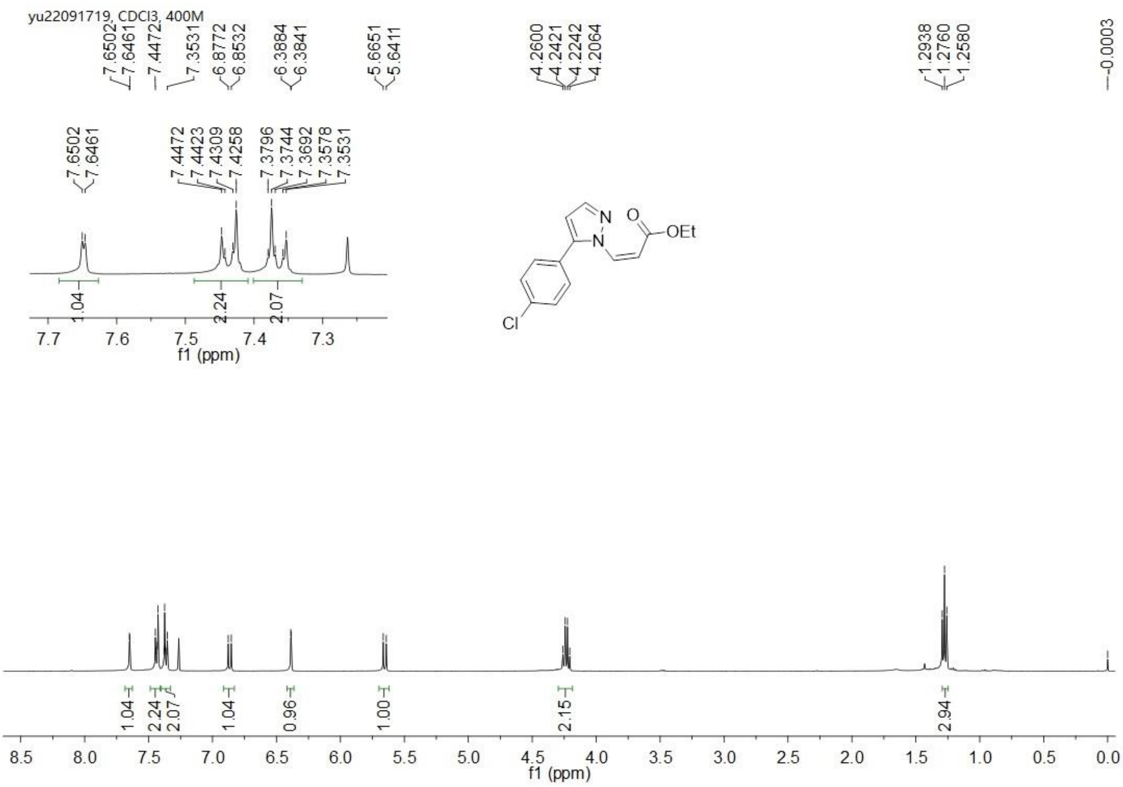

^{1}H NMR of **6e'**

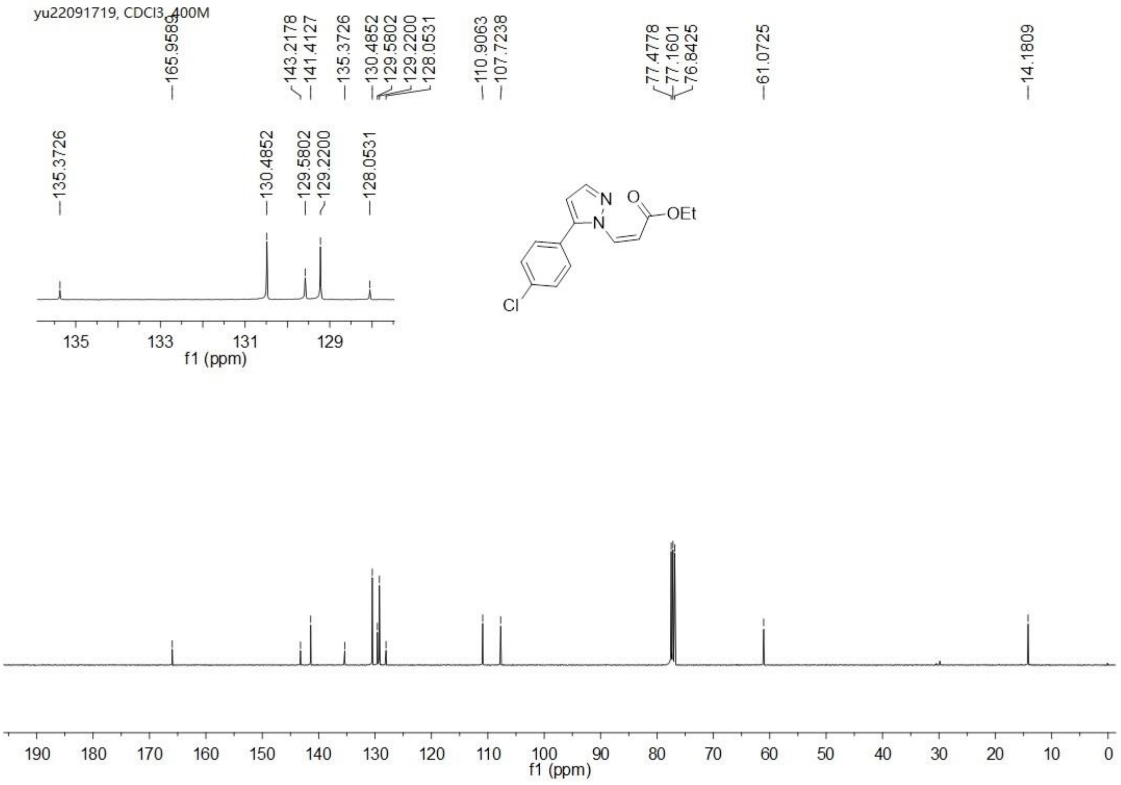

^{13}C NMR of **6e'**

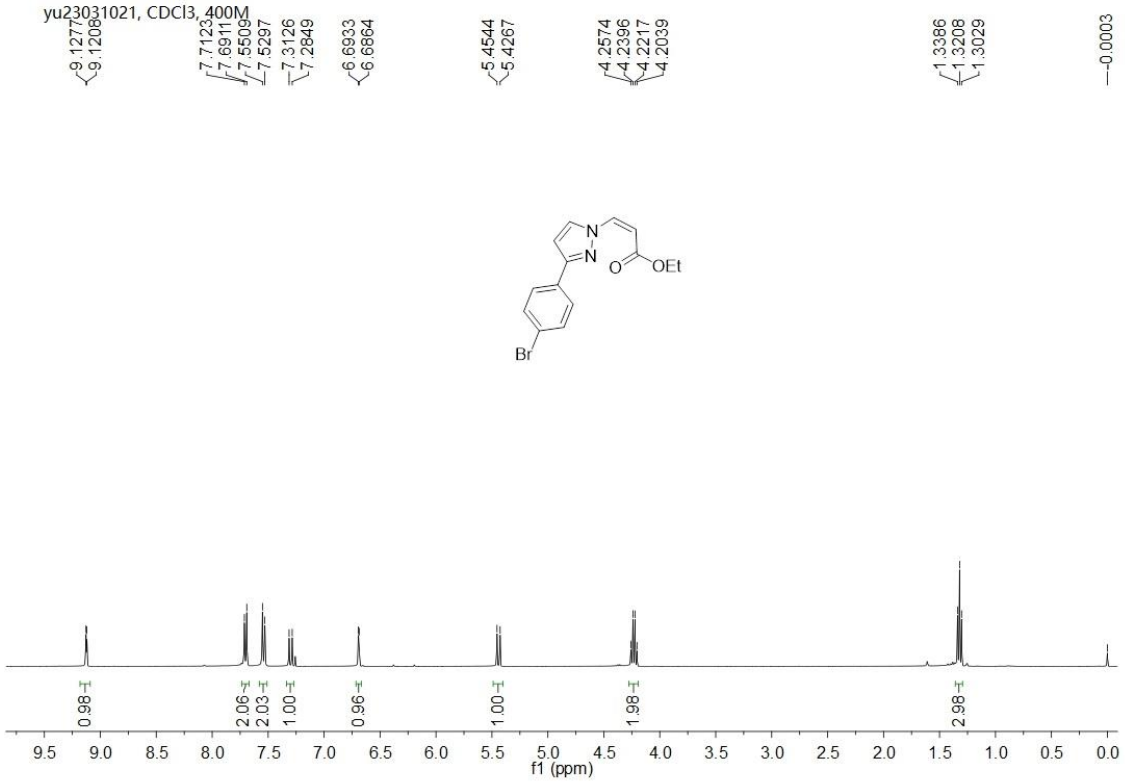

^{1}H NMR of **6f**

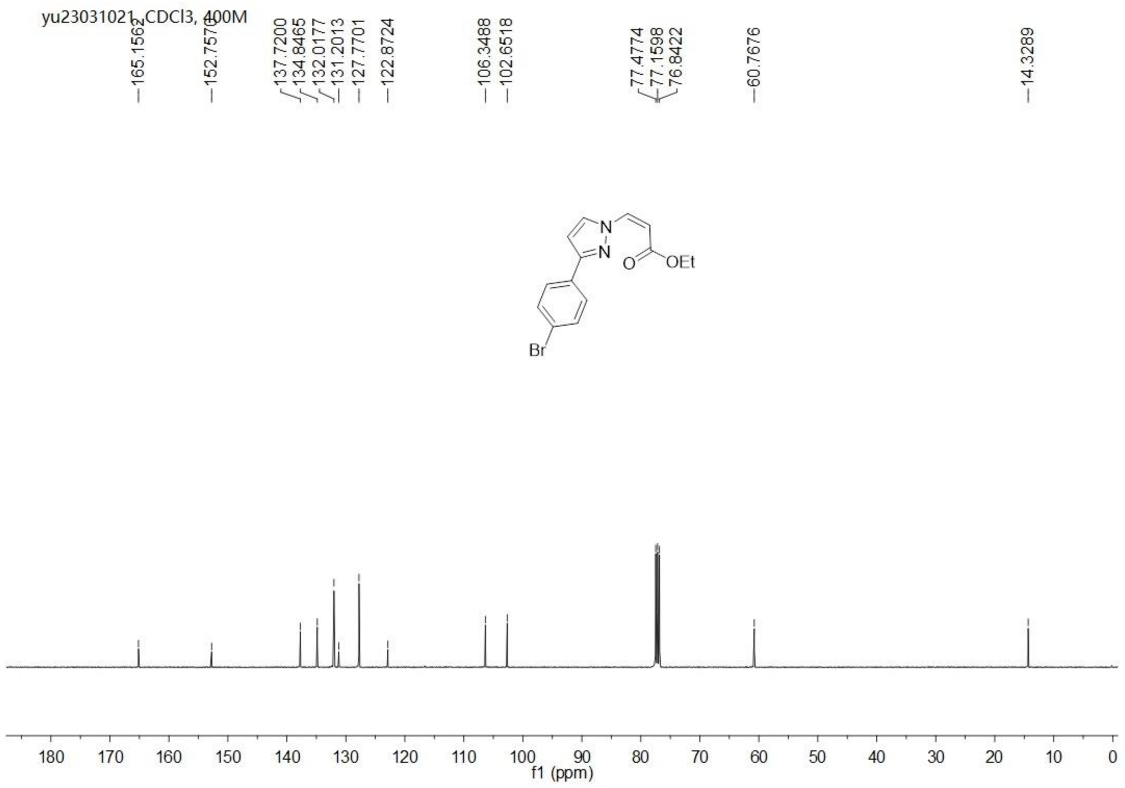

^{13}C NMR of **6f**

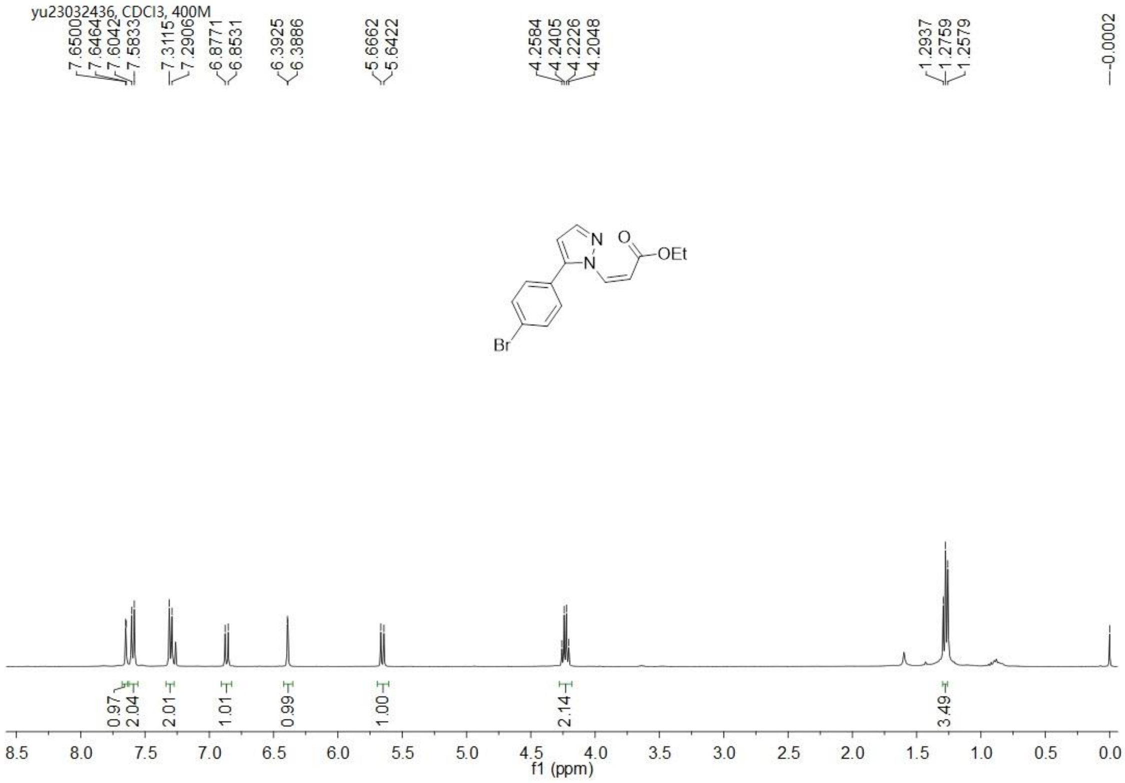

^{1}H NMR of **6f’**

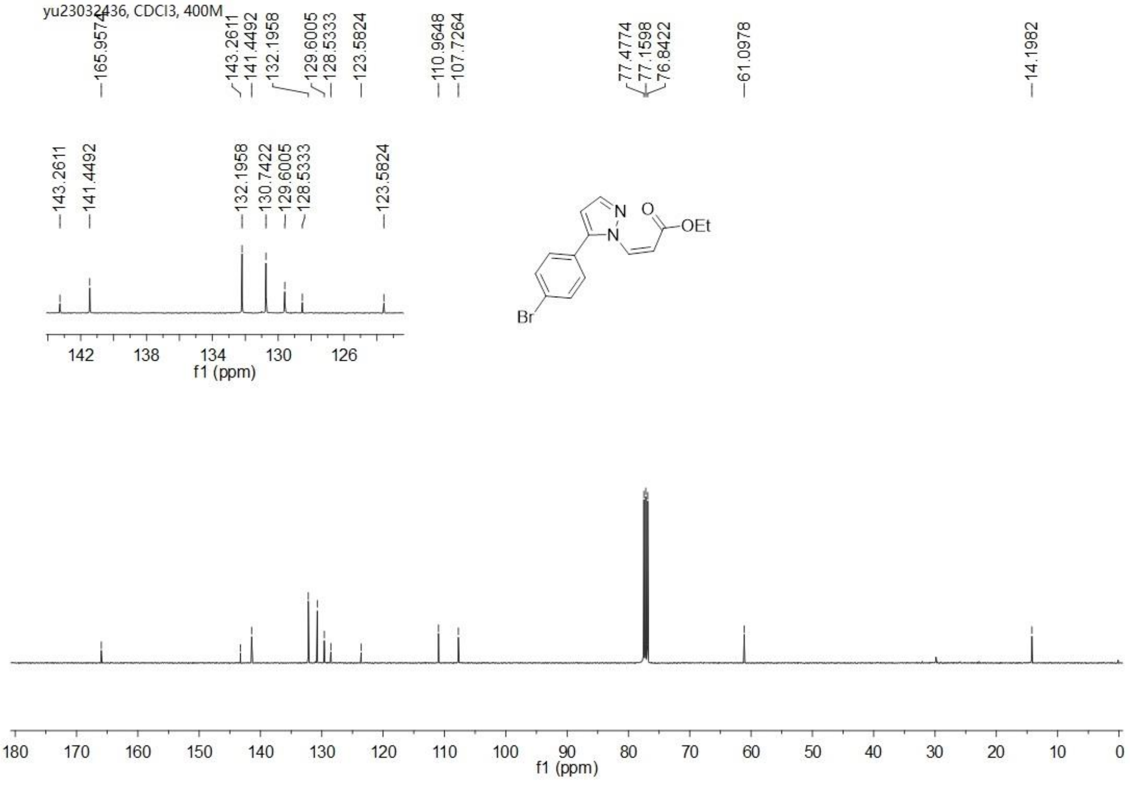

^{13}C NMR of **6f’**

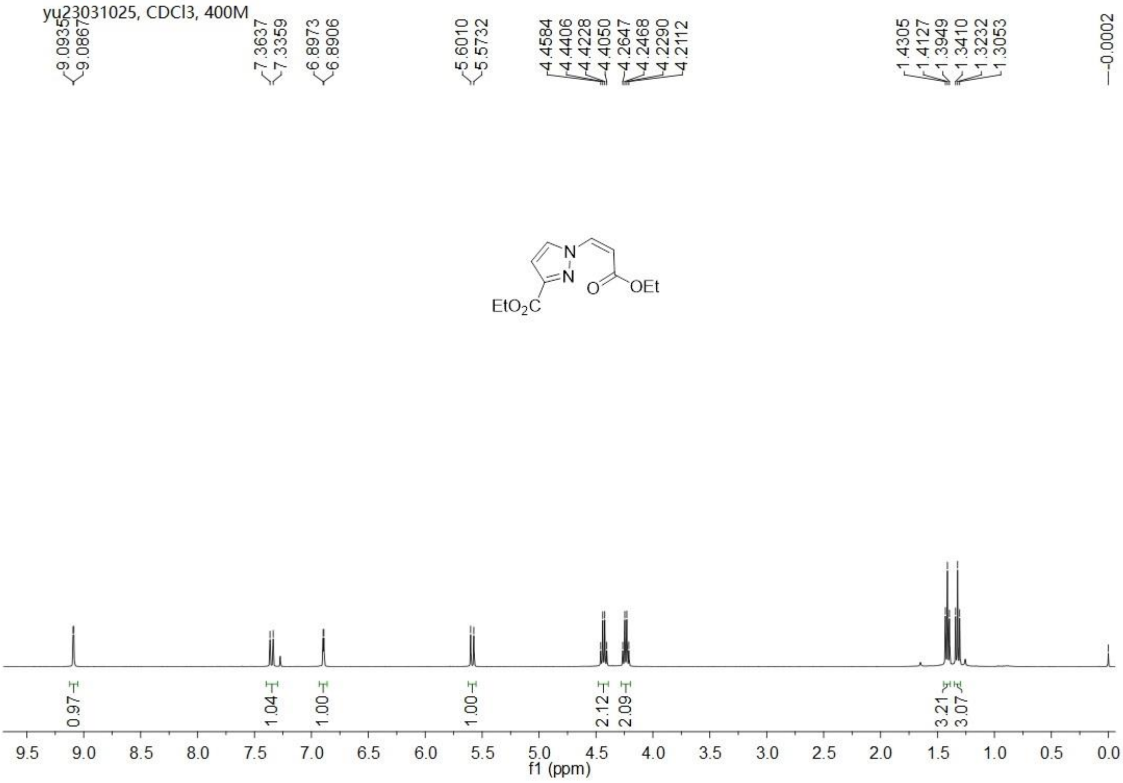

^{1}H NMR of **6g**

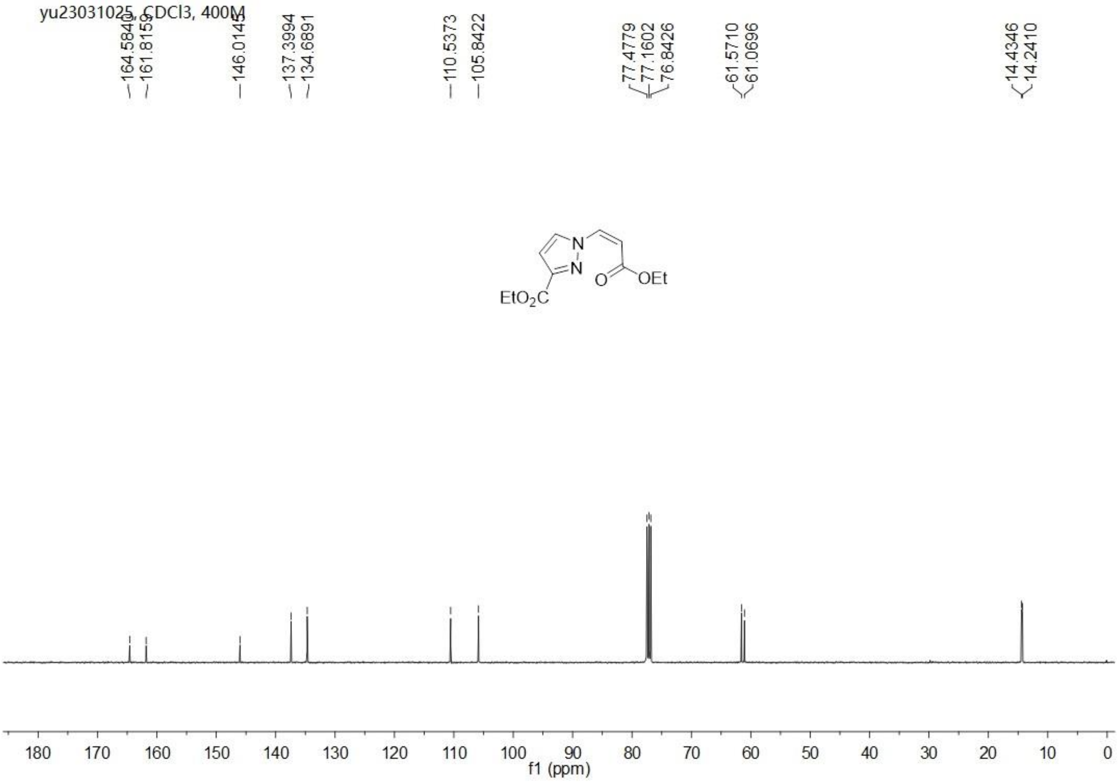

^{13}C NMR of **6g**

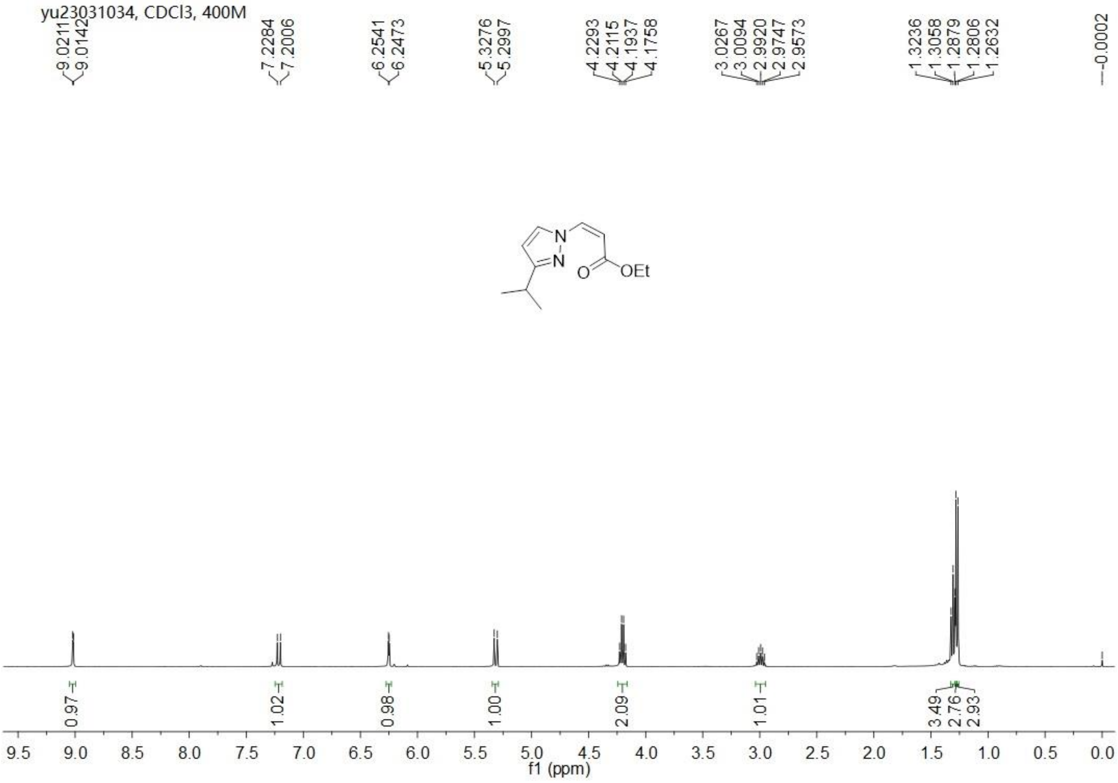

^{1}H NMR of **6h**

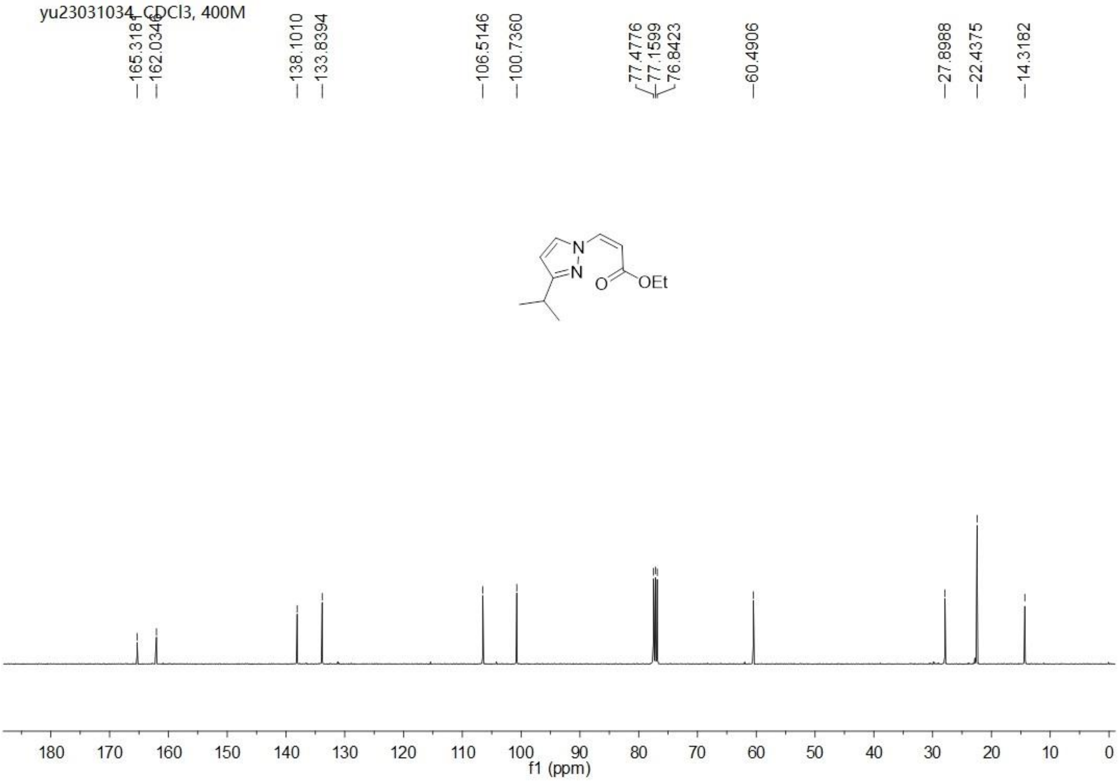

^{13}C NMR of **6h**

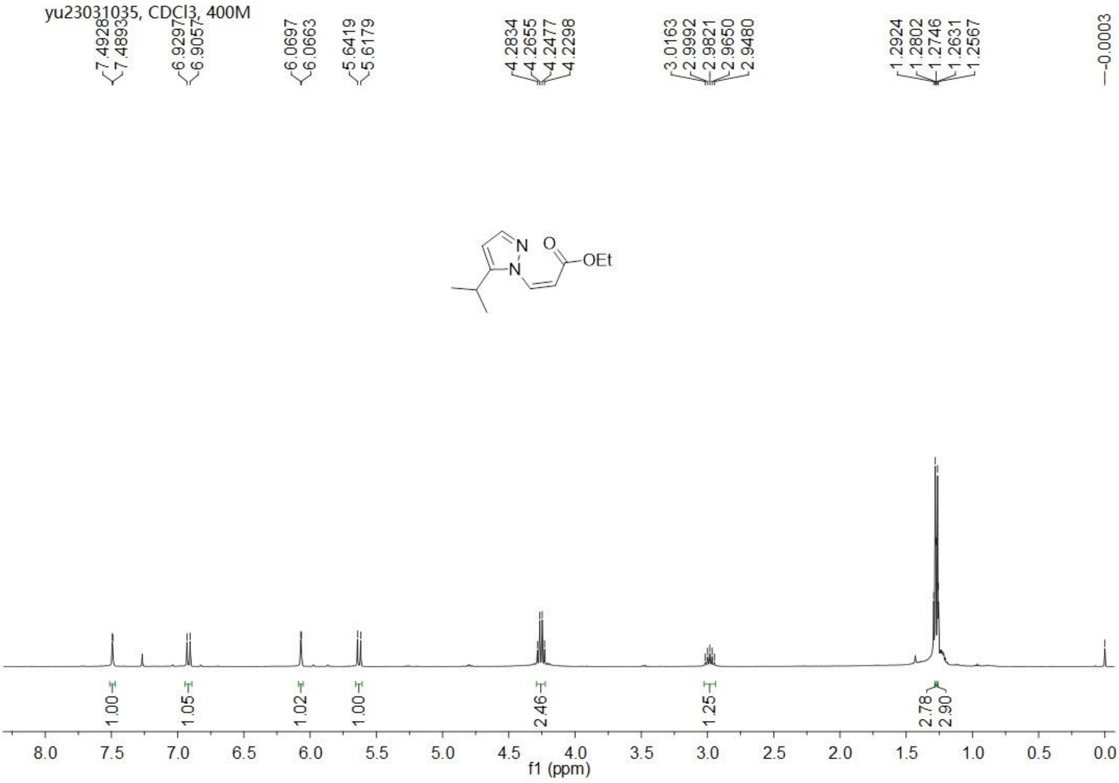

^{1}H NMR of **6h’**

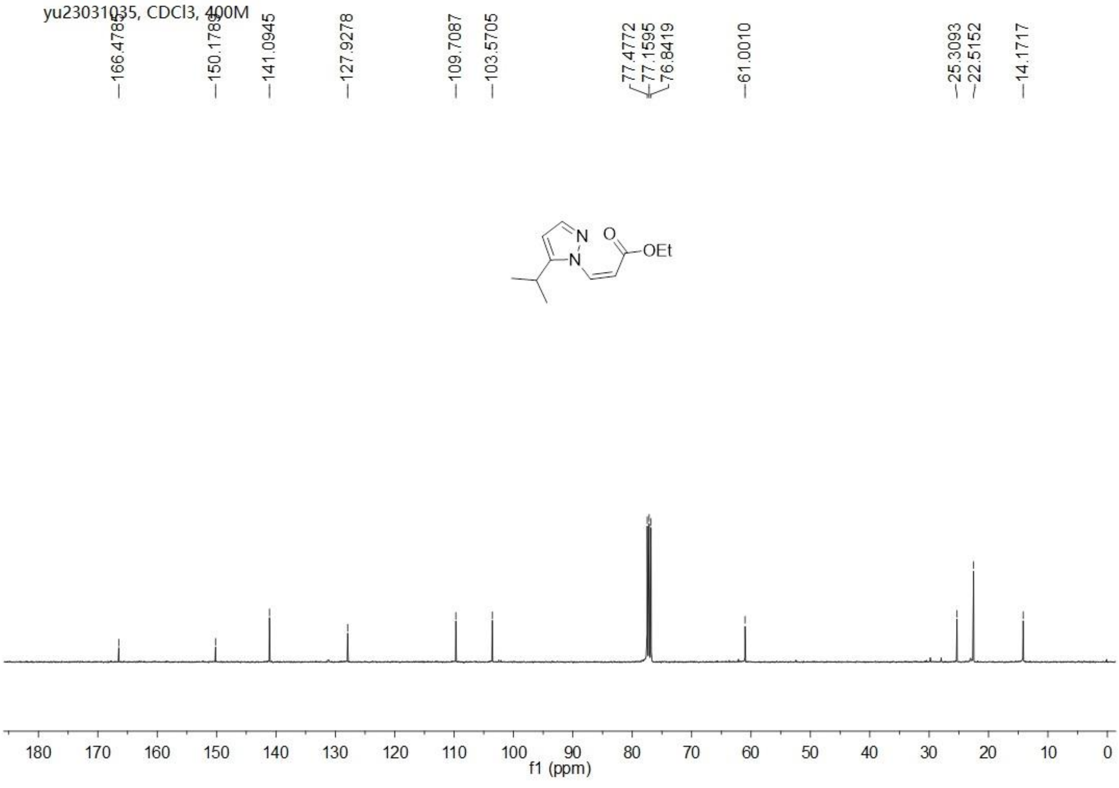

^{13}C NMR of **6h’**

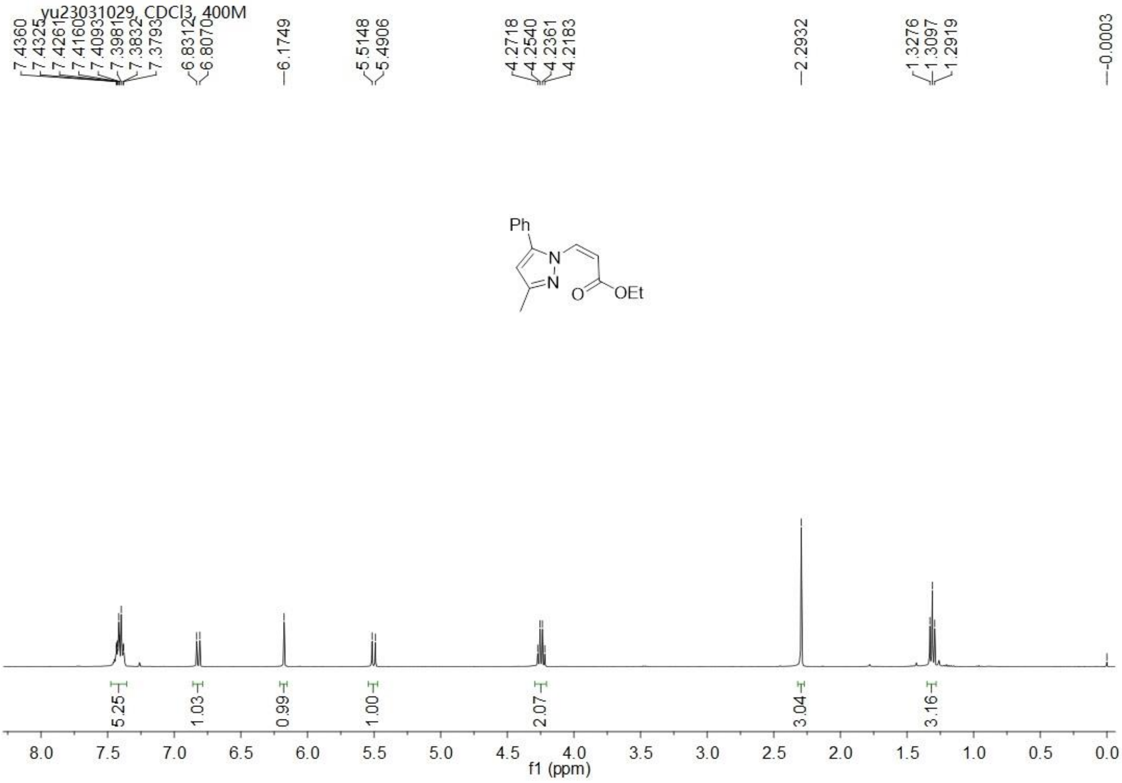

^{1}H NMR of **6i**

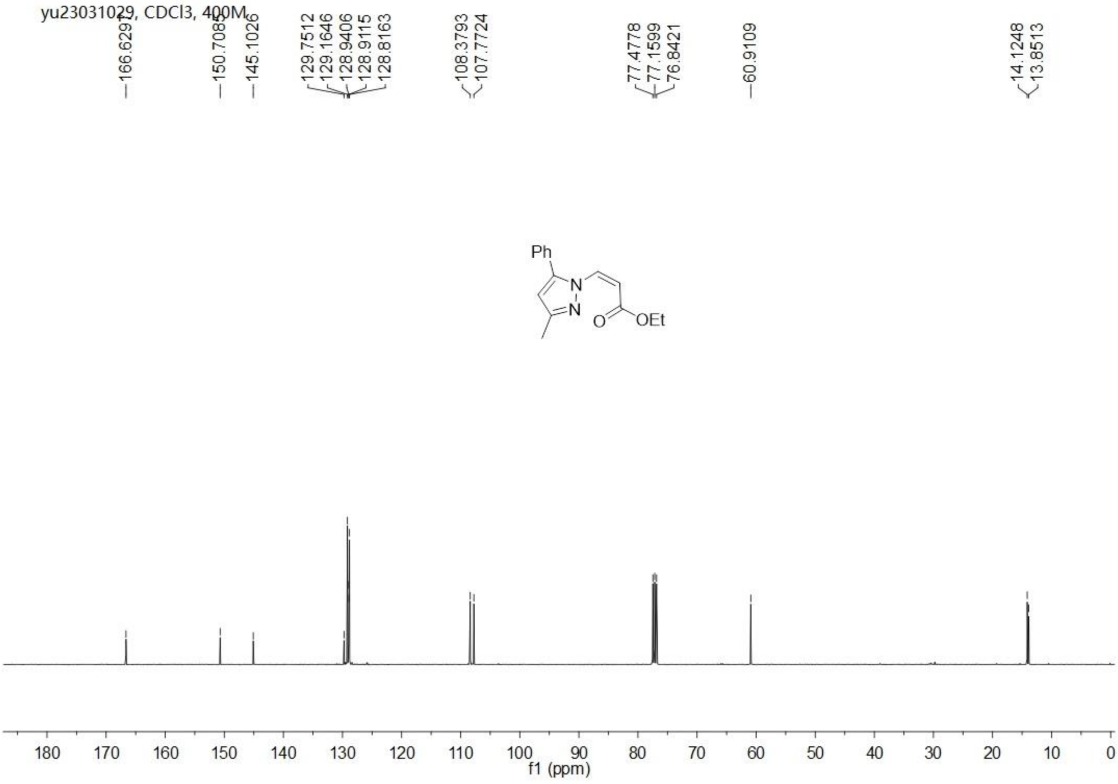

^{13}C NMR of **6i**

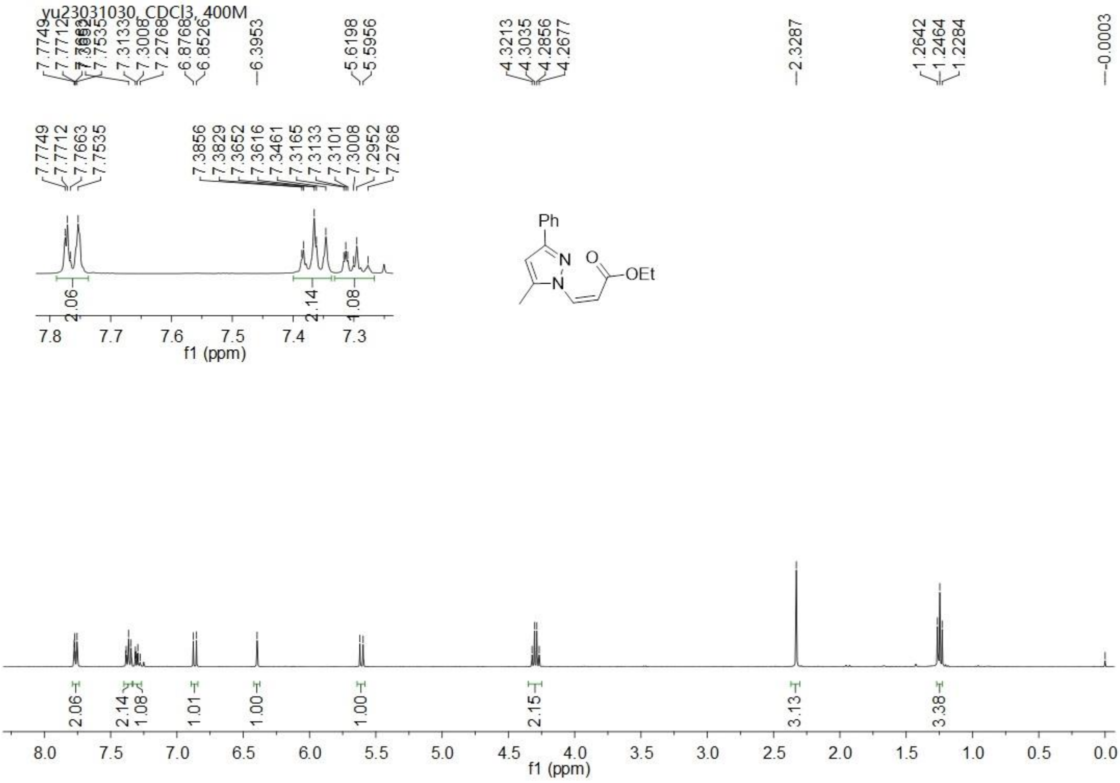

^{1}H NMR of **6i’**

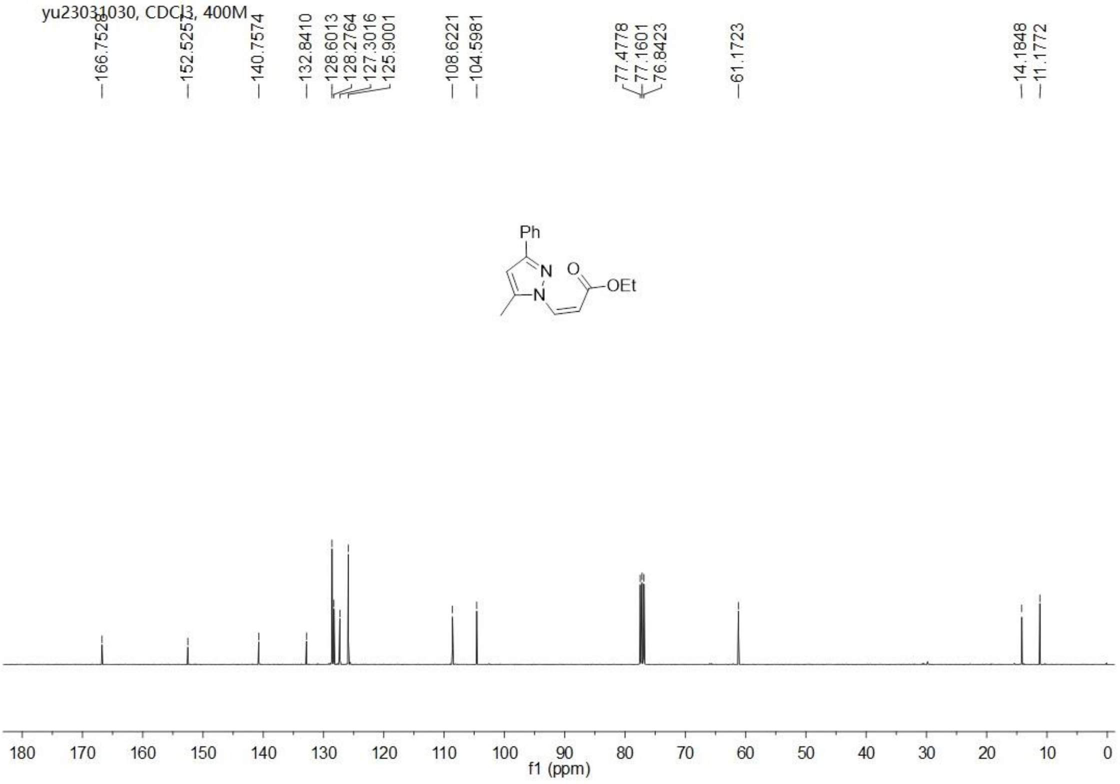

^{13}C NMR of **6i’**

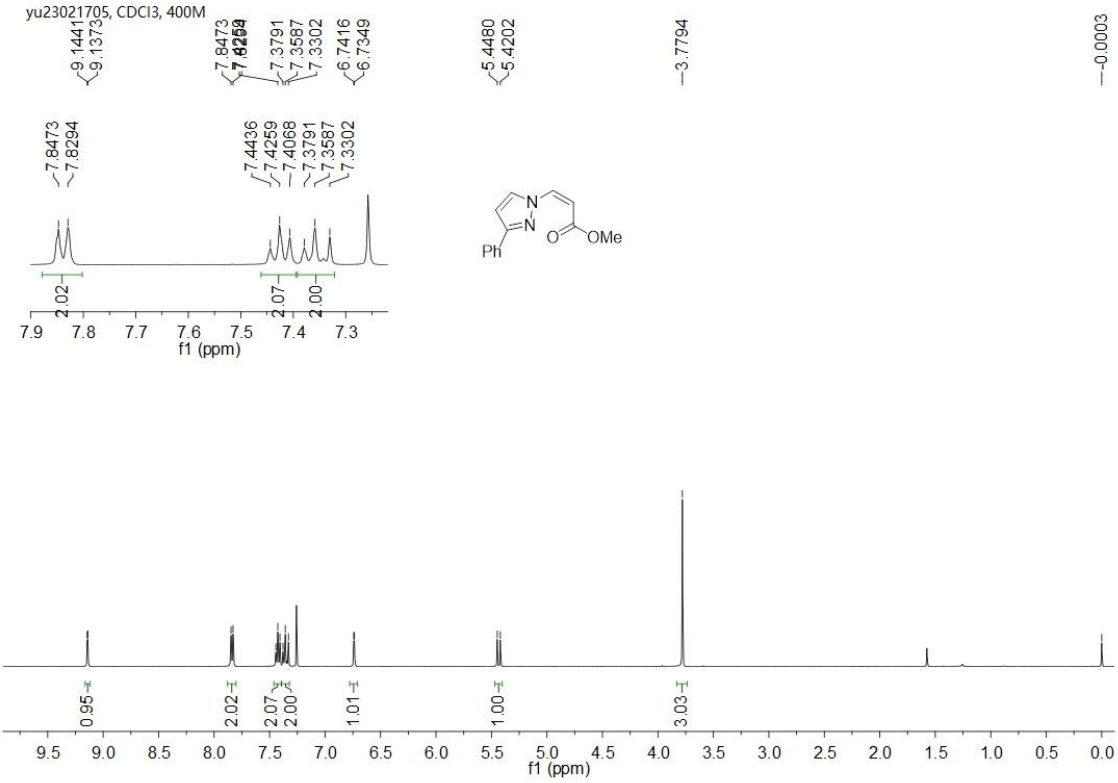

^{1}H NMR of **6j**

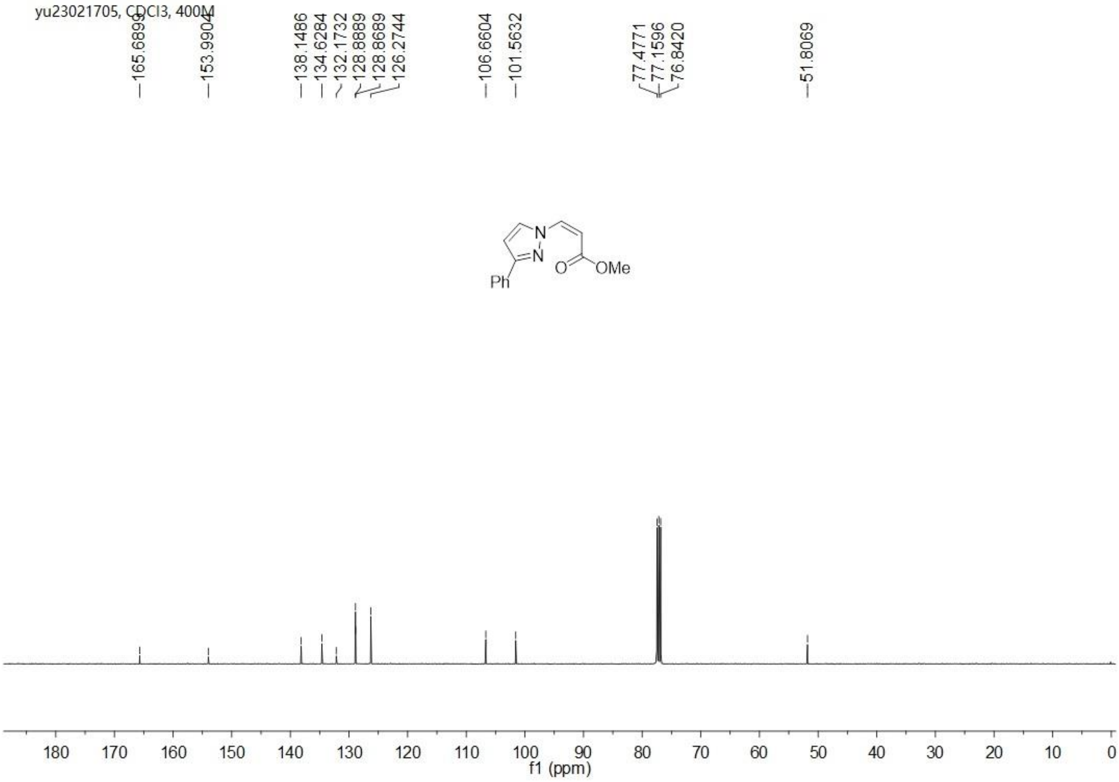

^{13}C NMR of **6j**

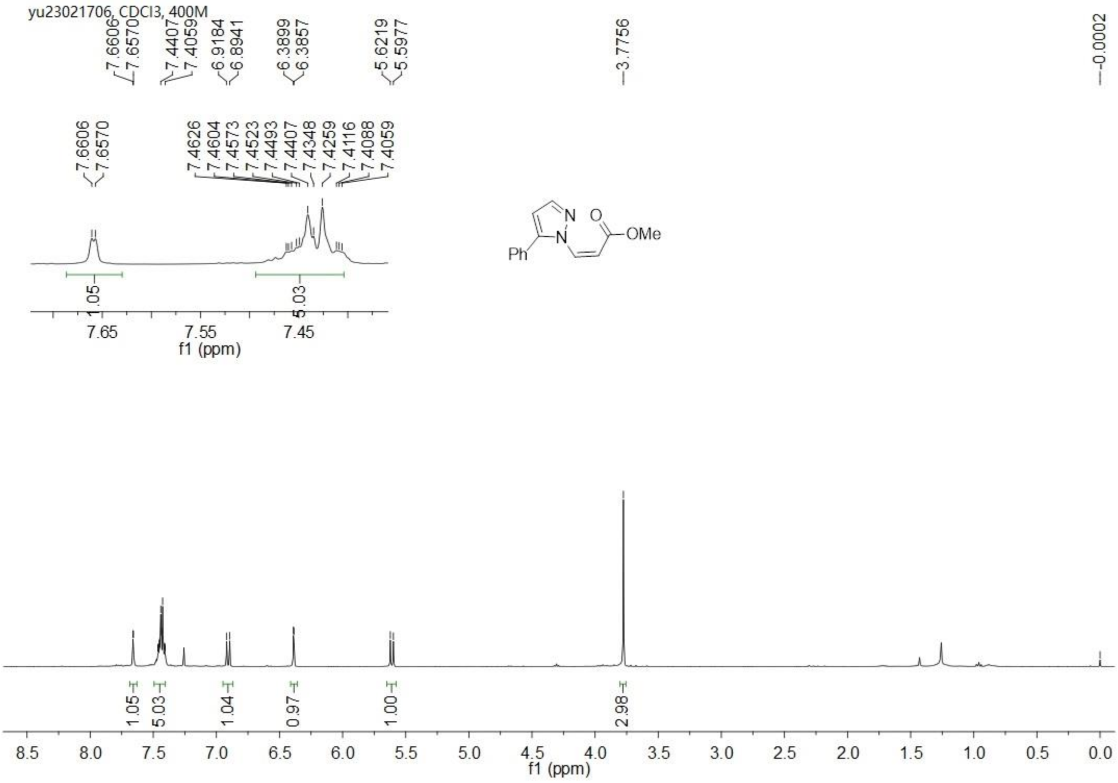

^{1}H NMR of **6j'**

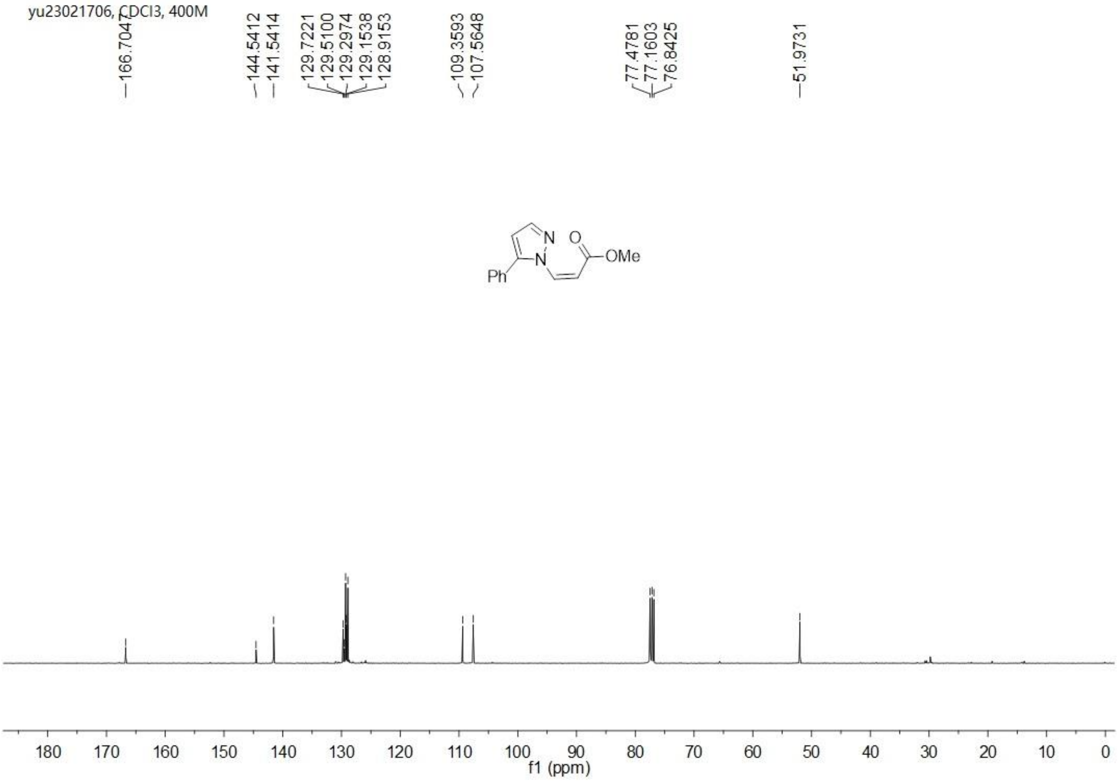

^{13}C NMR of **6j'**

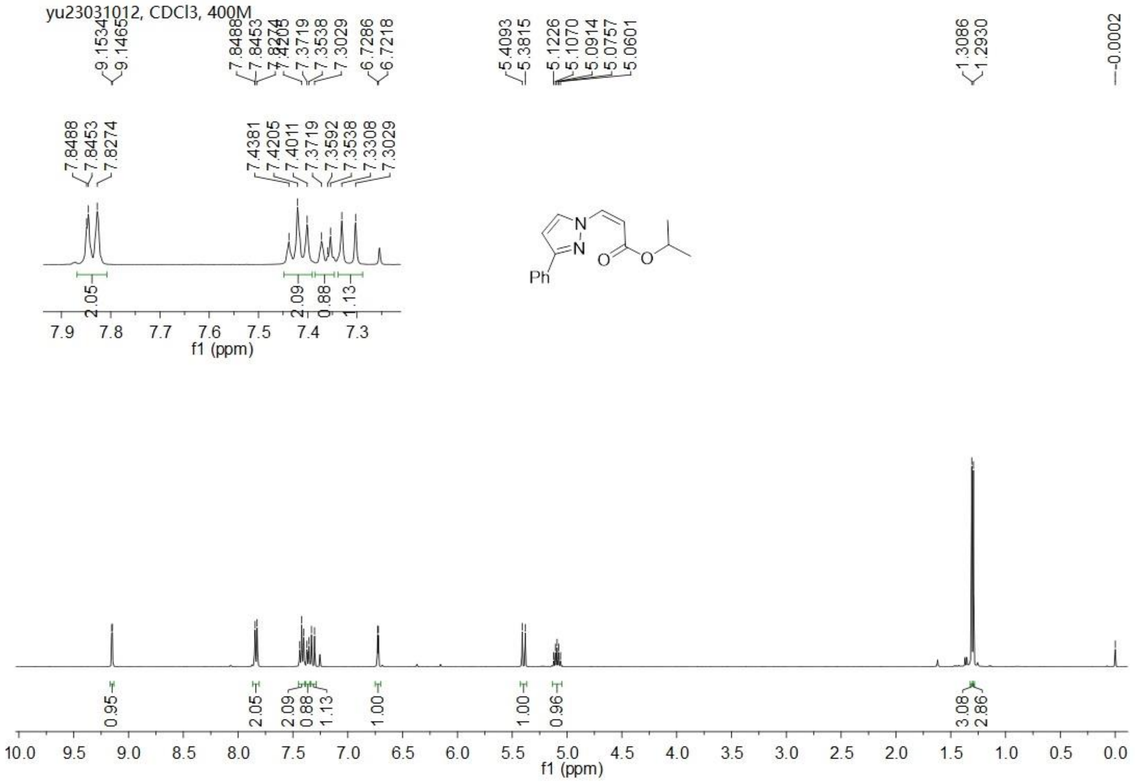

^{1}H NMR of **6k**

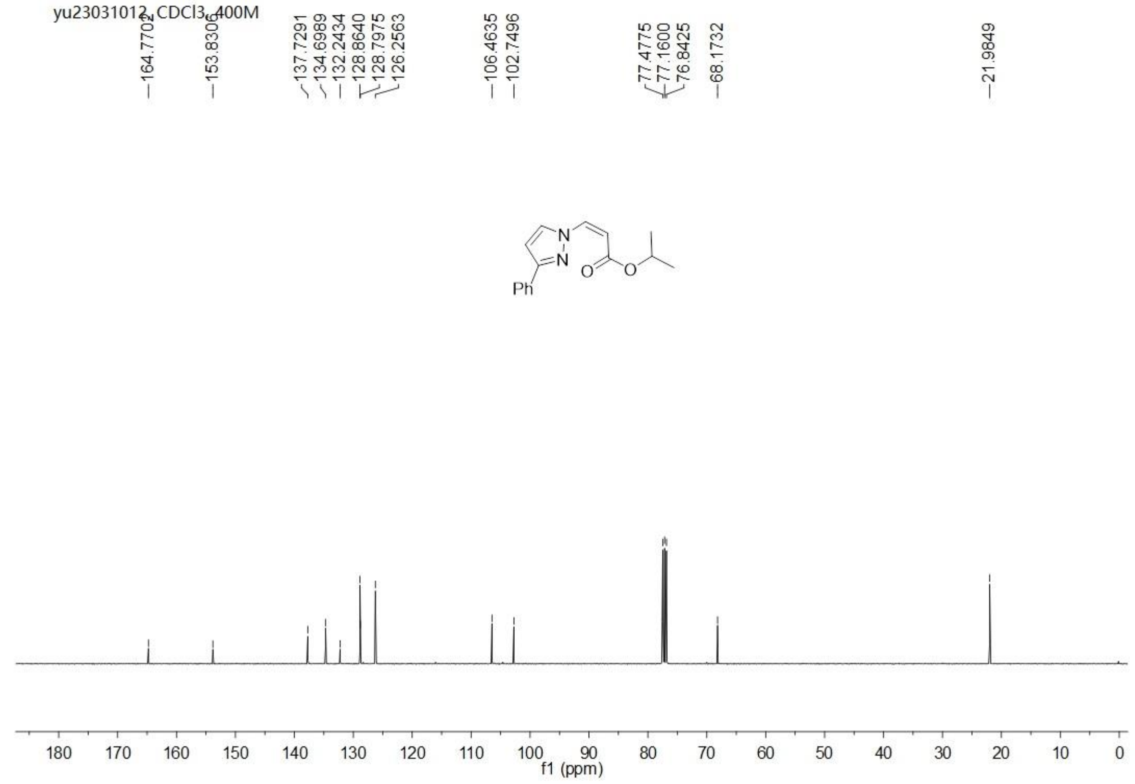

^{13}C NMR of **6k**

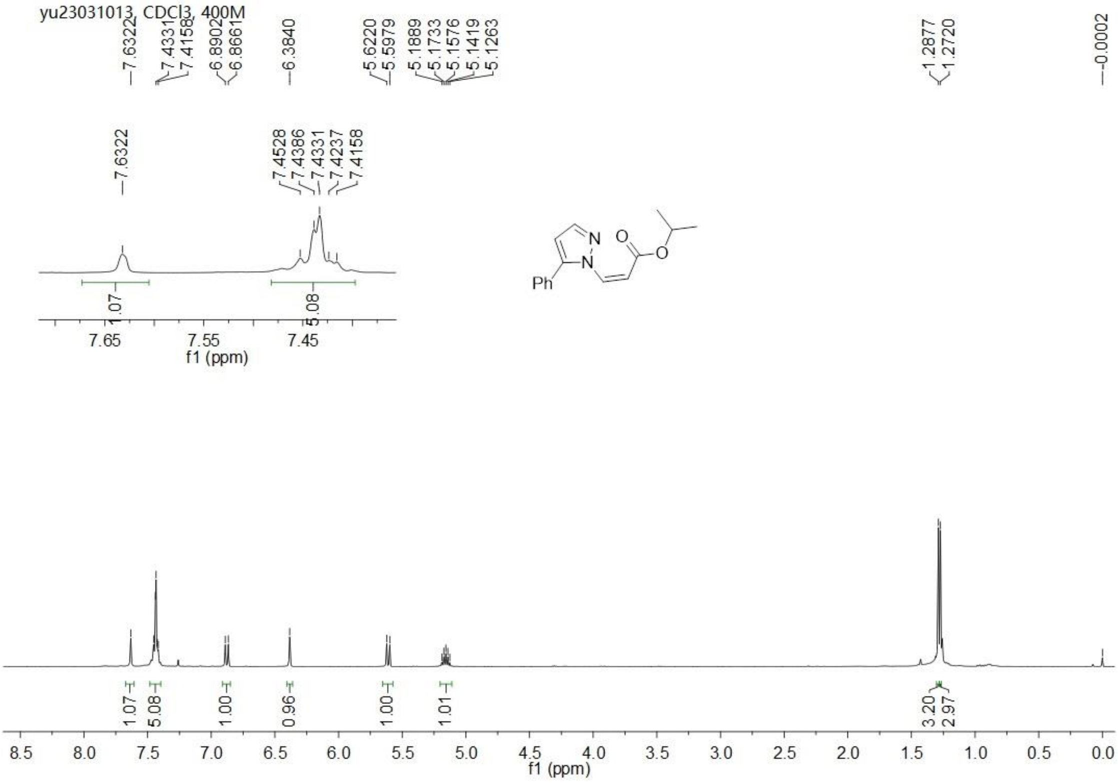

^{1}H NMR of **6k’**

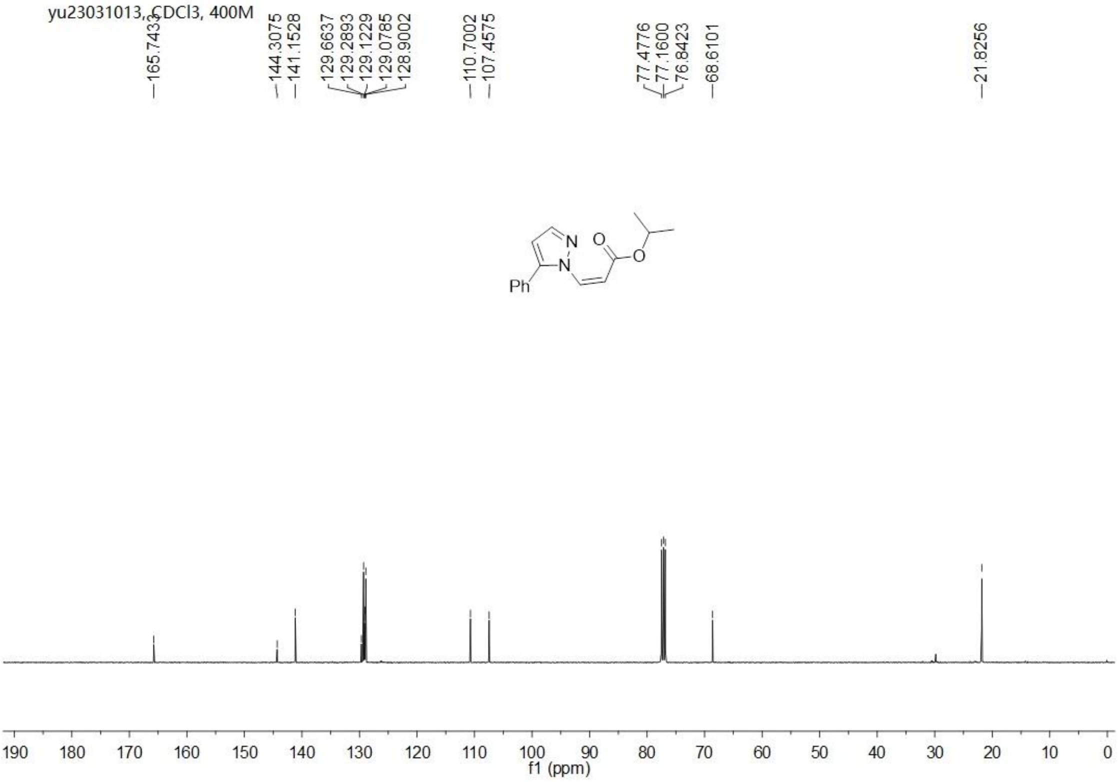

^{13}C NMR of **6k’**

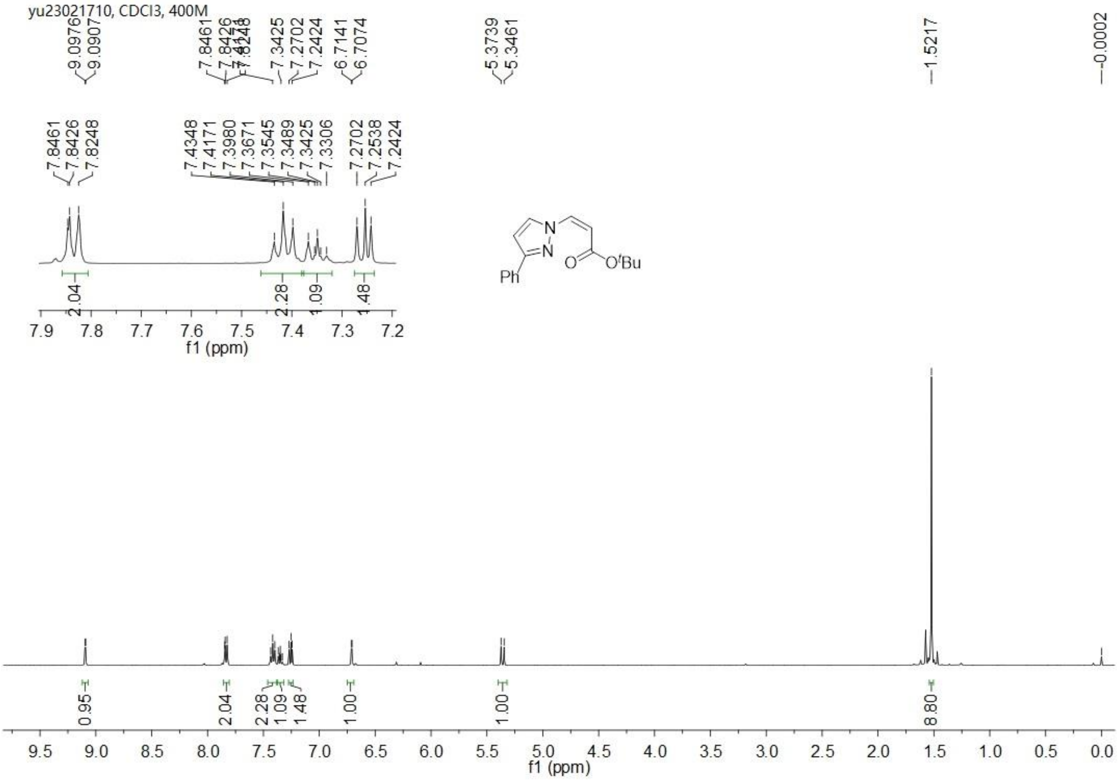

^{1}H NMR of **6l**

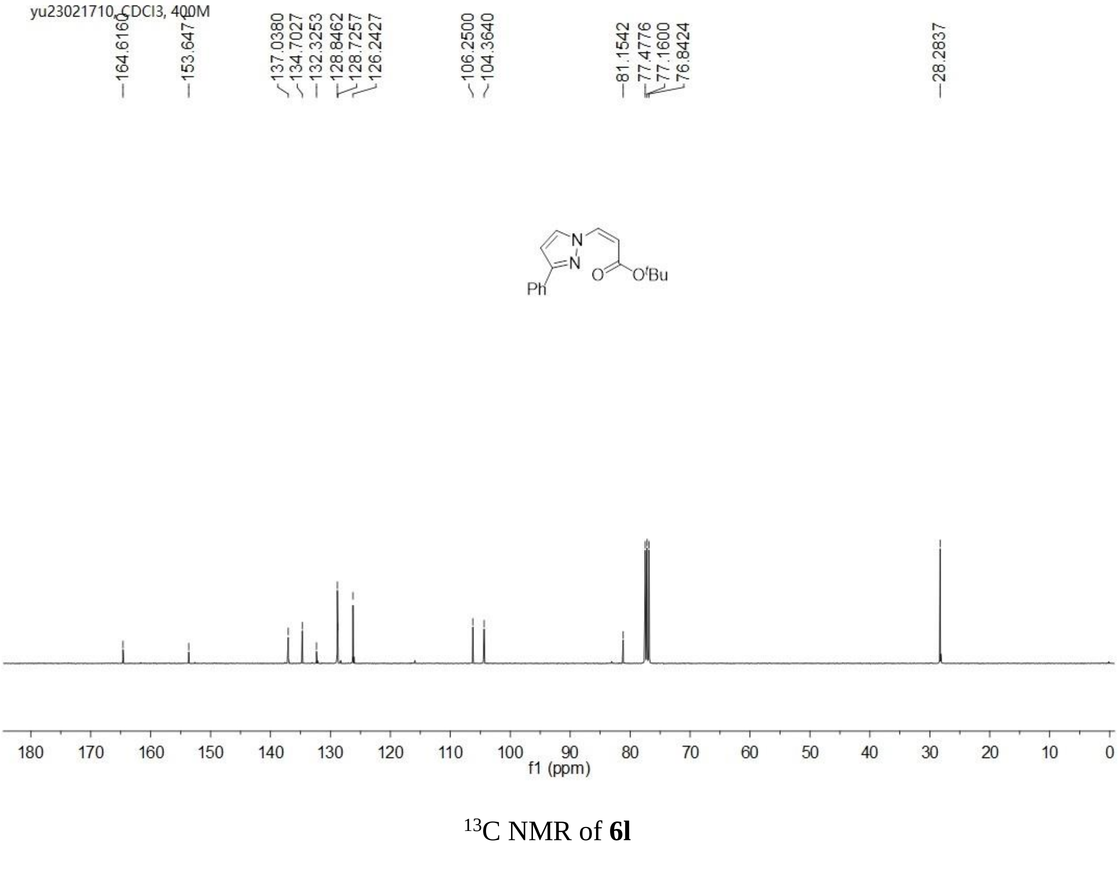

^{13}C NMR of **6l**

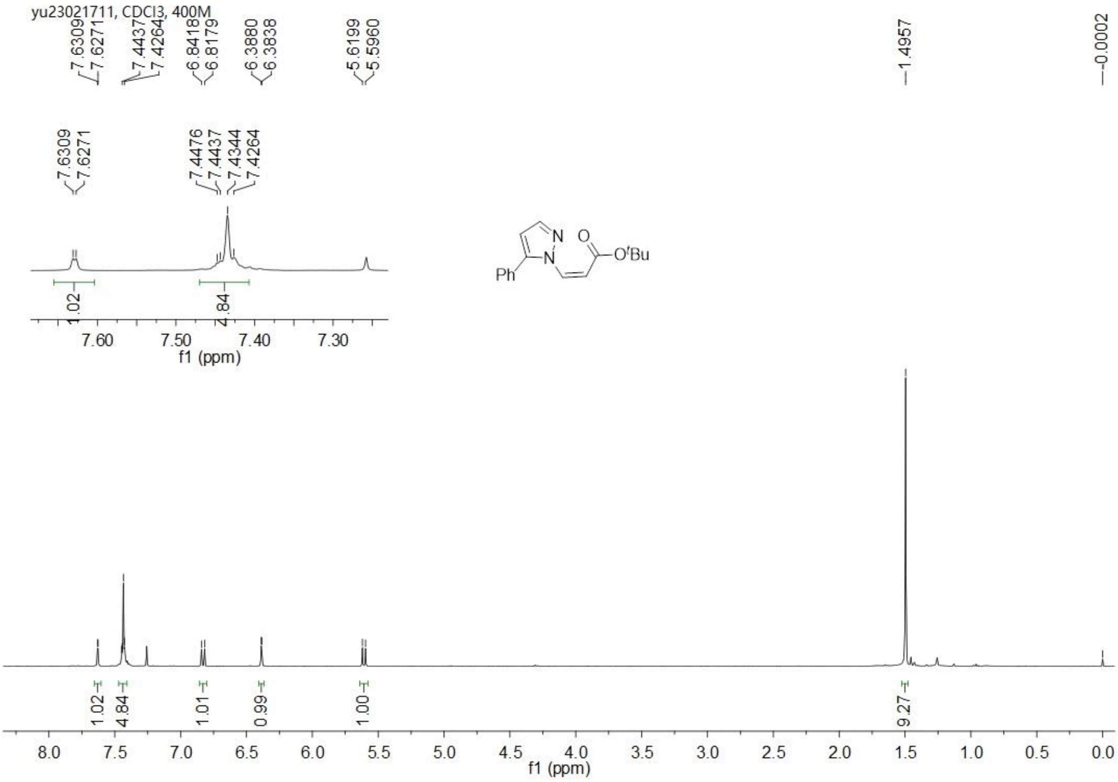

^{1}H NMR of **6l’**

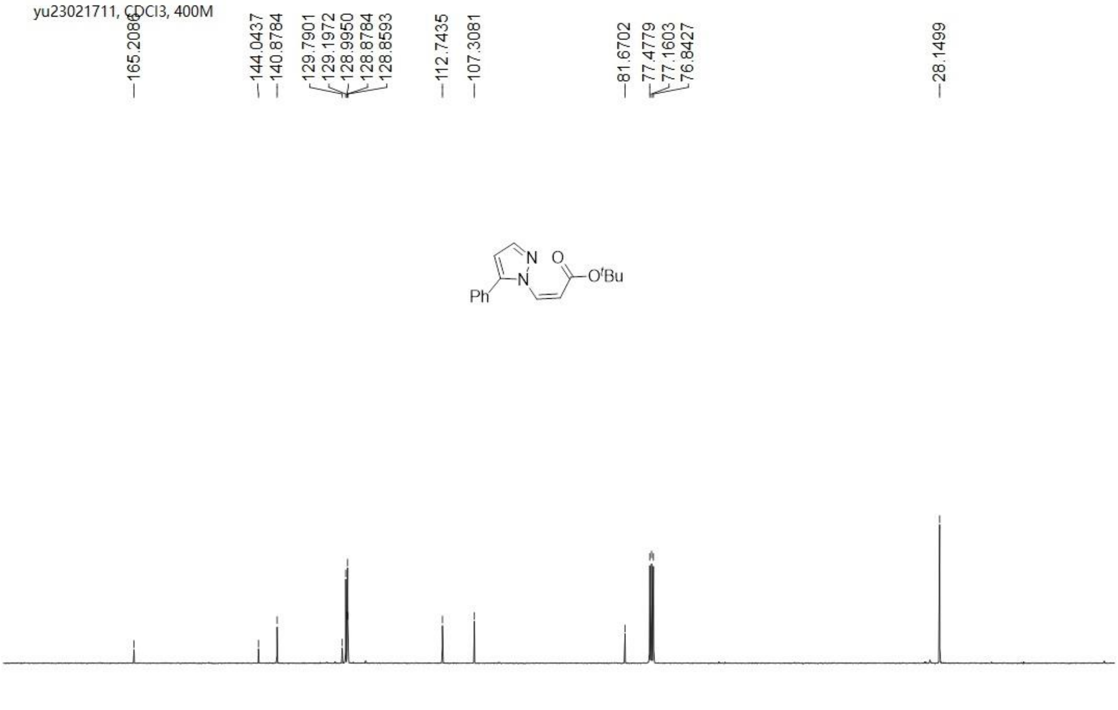

^{13}C NMR of **6l’**

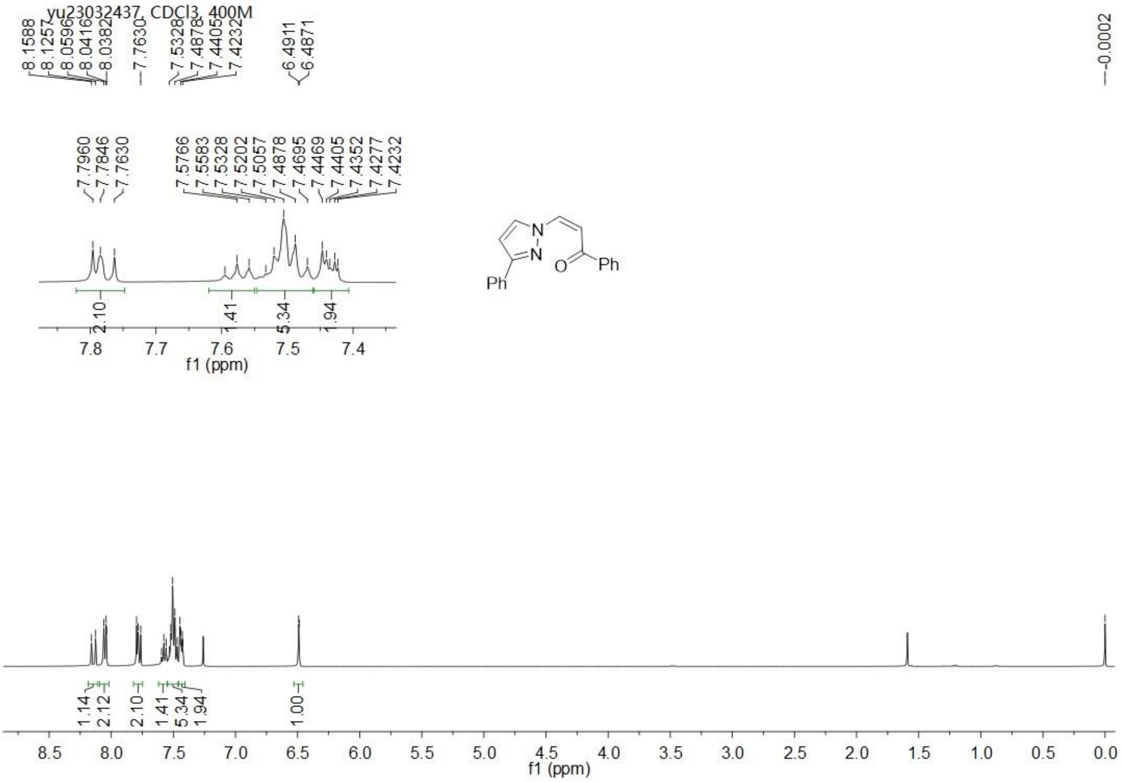

^{1}H NMR of **6m**

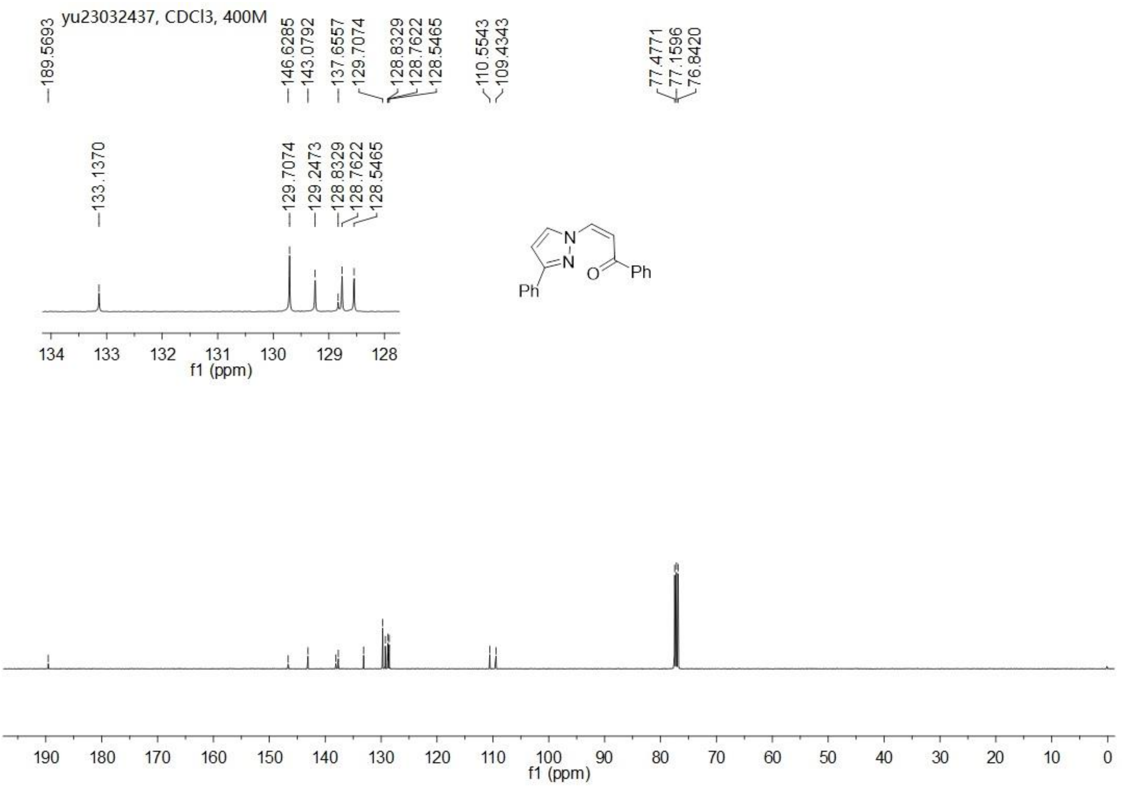

^{13}C NMR of **6m**

VI. Crystal Data of **3l**, **4o**, **5c**, **5k’**, **6c**

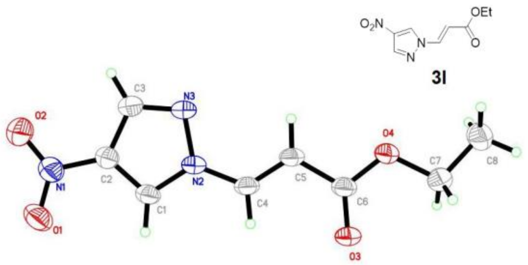

Crystal data of **3l**

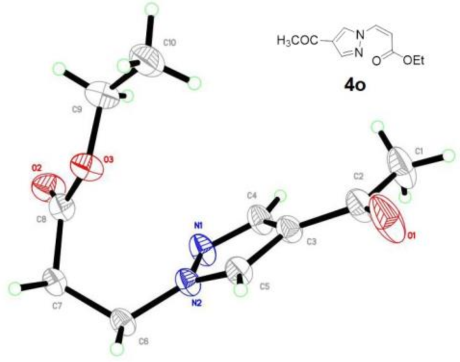

Crystal data of **4o**

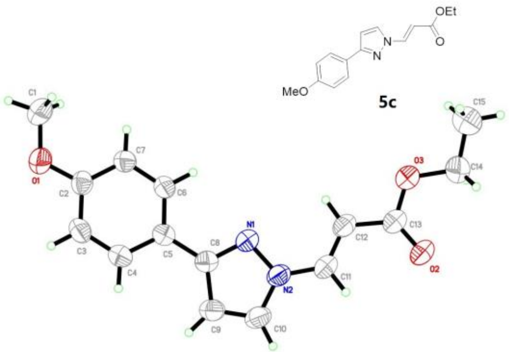

Crystal data of **5c**

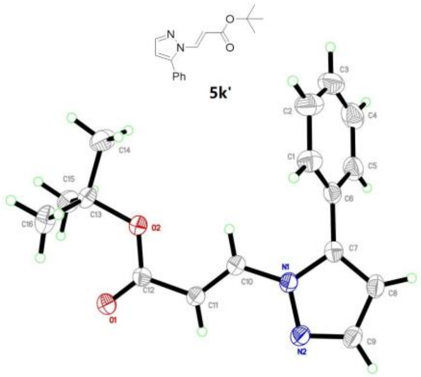

Crystal data of **5k’**

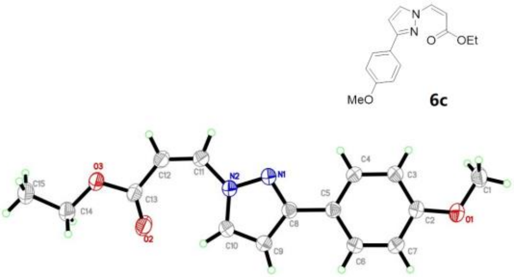

Crystal data of **6c**
